# Evolutionary assessment of *SQUAMOSA PROMOTER BINDING PROTEIN-LIKE* genes in citrus relatives with a specific focus on flowering

**DOI:** 10.1186/s43897-023-00061-4

**Published:** 2023-07-20

**Authors:** Yawei Li, Shuting Wang, Prakash Babu Adhikari, Bing Liu, Shengjun Liu, Yue Huang, Gang Hu, Michitaka Notaguchi, Qiang Xu

**Affiliations:** 1 National Key Laboratory for Germplasm Innovation and Utilization for Fruit and Vegetable Horticultural Crops, Huazhong Agricultural University, Wuhan, 430000 China; 2 Bioscience and Biotechnology Center, Nagoya University, Furo-cho, Chikusa-ku, Nagoya, 464-8601 Japan; 3 Hubei Hongshan Laboratory, Wuhan, 430070 China

**Keywords:** SPL, Gene Family, Phylogeny, Phase Transition, Early Flowering

## Abstract

Phase transition and floral induction in citrus requires several years of juvenility after germination. Such a long period of juvenility has been a major hindrance to its genetic improvement program. Studies have shown that miR156 along with its downstream genes SQUAMOSA PROMOTER BINDING PROTEINS (SBP) and SBP-LIKE (SPL) mediate the phase transition and floral induction process in plants. Our current study has systematically analyzed *SPL*s in 15 different citrus-related species, systematically annotated them based on their close homology to their respective Arabidopsis orthologs, and confirmed the functional attributes of the selected members in floral precocity. The majority of the species harbored 15 *SPL*s. Their *cis*-element assessment suggested the involvement of the *SPL*s in diverse developmental and physiological processes in response to different biotic and abiotic cues. Among all, *SPL5*, *SPL9*, and *SPL11* stood out as consistently differentially expressed *SPL*s in the adult and young tissues of different citrus-related species. Independent overexpression of their *F. hindsii* orthologs (*FhSPL5*, *FhSPL9*, and *FhSPL11*) brought an enhanced expression of endogenous *FLOWERING LOCUS T* leading to the significantly precocious flowering in transgenic Arabidopsis lines. Future study of the genes in the citrus plant itself is expected to conclude the assessments made in the current study.

**Graphical Abstract:**

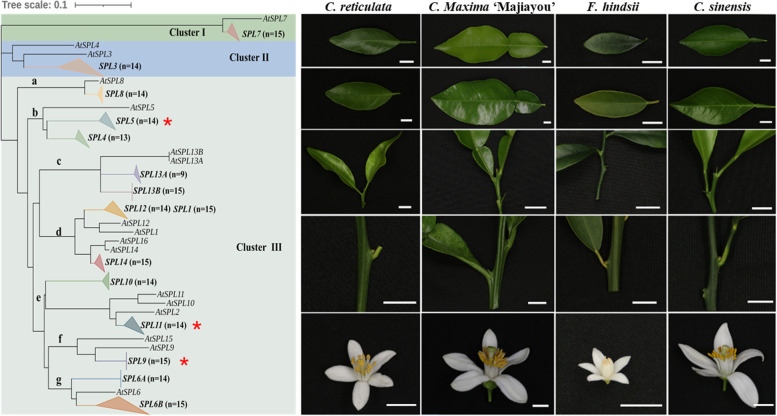

**Supplementary Information:**

The online version contains supplementary material available at 10.1186/s43897-023-00061-4.

## Core

By systematically identifying the members of the SPL family and comparing their expression levels in in the adult and young tissues of different citrus-related species, it was found that SPL5, SPL9, and SPL11 play an important role in the regulation of childhood in citrus. Independent overexpression of their *F. hindsii* orthologs (FhSPL5, FhSPL9, and FhSPL11) brought an enhanced expression of endogenous FT leading to the significantly precocious flowering in transgenic Arabidopsis lines.

## Gene & accession numbers

Most of their genomes are included in the local Citrus Pan-genome to Breeding database (CPBD; http://citrus.hzau.edu.cn/). Some genomic data has not been published, please contact the author if necessary. Sequence data from this article can be found in the database of the National Center for Biotechnology Information (NCBI) under the accession numbers: FhSPL5(OR234301), FhSPL9 (OR234302), FhSPL11 (OR234303), CsActin (Cs1g_pb000860), AtFT (NM_001334207.1), AtAcin (NM_112764.4).

## Introduction

Juvenile citrus plants are characterized by thorniness and vigorous growth unlike their frequently flowering adult counterparts (Furr et al. 1947; Hendry 1982). As compared to the annual herbaceous plants, the very first floral induction (after phase transition) in citrus-like perennial plants take a relatively much longer time after germination. While some citrus species start flowering at age as early as 4-6 months after germination in a tropical climate, others spend 4-15 years of juvenility before flowering (Table 1). The long juvenility of the majority of citrus species has been a serious hindrance to rapid yield gain and breeding cycle. Hence, several attempts have been made and practiced to circumvent the issue since long (Furr et al. 1947; Moss 1969; Goldschmidt et al. 1985; Endo et al. 2005; Soares et al. 2020). However, there has not been a significant breakthrough in reducing juvenility in the species yet. It still necessitates better know-how on the molecular and physiological processes behind phase transition and floral induction.

**Table 1:** Number of SPLs discovered in citrus members and relatives

**Species**	**Ploidy**	**Juvenility**	**Number of SBP-box gene**	**SPLs w/o miR156-target sites**
*Aegle marmelos*	2	>12 years	15	1, 7, 8, 12, 14
*Murraya paniculata*	2	2-3 years	14	1, 7, 8, 12, 14
*Atalantia buxifoliata*	2	2-3 years	15	1, 7, 8, 12, 14
*Clausena lansium*	2	2-3 years	15	1, 7, 8, 12, 14
*Citropsis gilletiama*	2	> 3 years	15	1, 7, 8, 12, 14
*Poncirus trifoliata*	2	4-5 years	15	1, 7, 8, 12, 14
*Fortunella hindsii*	2	4-6 months	15	1, 7, 8, 12, 14
*Citrus mangshannensis*	2	8-10 years	15	1, 7, 8, 12, 14
*Citrus ichangensis*	2	8-10 years	15	1, 7, 8, 12, 14
*Citrus sinensis*	2	4-6 years	15	1, 7, 8, 12, 14
*Citrus reticulata*	2	4-6 years	15	1, 7, 8, 12, 14
*Citrus hongheensis*	2	10-12 years	15	1, 7, 8, 12, 14
*Citrus maxima* ‘Majiayou’	2	6-8 years	15	1, 7, 8, 12, 14
*Citrus maxima* ‘Zipi’	2	6-8 years	12	1, 7, 8, 12, 14
*Citrus medica*	2	4-5 years	15	1, 7, 8, 12, 14

Flowering in higher plants like citrus and Arabidopsis is tightly regulated by the interplay of diverse molecular players. Often multiple pathways (vernalization, autonomous, photoperiod, hormone, and age pathways) converge during the floral induction (Teotia and Tang 2015; Kim 2020). Compared to others, age-dependent floral induction is fail-safe in plants as it occurs even in non-inductive conditions. However, it essentially depends on the de-repression of *miR156*-regulated *SQUAMOSA PROMOTER BINDING-LIKE* (*SPL*) genes (Wu et al. 2009). Such change is apparently threshold dependent as the progressive decrease in miR156 transcript abundance in the subsequent leaves facilitates leaf morphogenesis (He et al. 2018). *miR156* and one of its target *SPL* member, *SPL9* oppositely regulate the age-dependent activation of SUPPRESSOR OF OVEREXPRESSION OF CONSTANS 1 (*SOC1*), a crucial MADS-box floral activator in Arabidopsis (Wang et al. 2009). An orthologous transgenic study with *C. sinensis* derived *SOC1* showed that its overexpression in Arabidopsis leads to precocious flowering and delayed senescence of the flowers in the plants (Tan and Swain 2007), indicating the functional conservation of *CsSOC1*. Whether *CsSPL9* too is involved in its regulation in citrus was yet to be unraveled.

Apart from their role in age-dependent flowering, citrus studies have shown the involvement of *miR156* in the positive regulation of somatic embryogenesis (Wu et al. 2011; Wu et al. 2015; Long et al. 2018), cellular starch accumulation (Liu et al. 2017), potential involvement in male sterility (Fang et al. 2016), etc. However, the detailed mechanism behind how *miR156*-*SPL* module shapes such phenotypes is yet to be elucidated. Moreover, even though there had been some studies on individual citrus species, they lack consensus on gene nomenclature.

In the current study, we have carried out a systematic assessment of *SPL*s from 15 representative citrus-related species (Aurantioideae family members) that included the earliest flowering *Fortunella hindsii* to late flowering citrus wild relative *Aegle marmelos*. Our study showed that *SPL*s are highly conserved among citrus members in their number and basic sequence features. Among 15 *SPL*s discovered in the majority of species, *SPL7/8* and *SPL3/4/5* were found to be the putatively most distant and the most recently evolved members respectively. We further assessed and confirmed the potential of *F. hindsii* derived *SPL*s (*FhSPL5*, *FhSPL9*, and *FhSPL11*) in floral precocity *via* orthologous overexpression in Arabidopsis.

## Results

### Citrus *SPL*s exhibit highly conserved sequence features

*SPL*s were retrieved from respective genomes of 15 citrus-related species, most of which are included in the local Citrus Pan-genome to Breeding Database (CPBD; http://citrus.hzau.edu.cn/) (Liu et al. 2022). Unlike Arabidopsis, which harbors 17 *SPL*s (including two identical copies of *AtSPL13*s), most of the citrus-related members (13 out of 15 species) appear to harbor 15 *SPL*s (Tables 1, S1). Interestingly, a manual homology search revealed that 14 *SPL* harboring *M. paniculata* too harbors the genomic region for its missing SPL (putative *MpSPL4*). However, it constitutes multiple deleterious point mutations within its putative exons. We analyzed the target sites of miR156 in the SPL gene family taking an earlier study by Liu *et al.* (2017) as a reference (Figure S1, Table S2). The results showed that except for *SPL1*, *SPL7*, *SPL8*, *SPL12*, and *SPL14*, all other *SPL*s harbor putative miR156 target sites (Table 1).

**Fig. 1:**
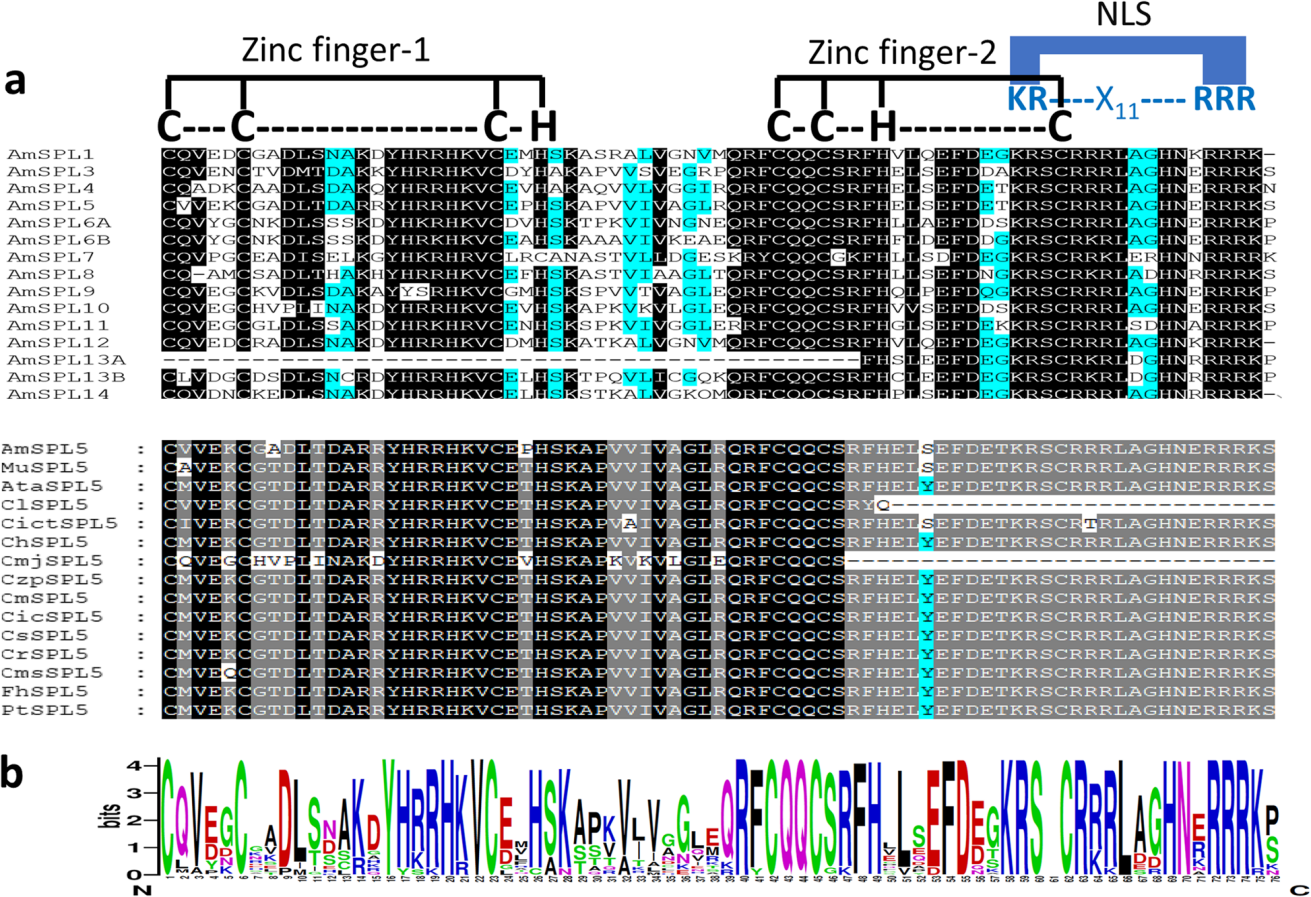
SBP domain-specific motifs are conserved in citrus-derived SPLs. **a** Conserved SBP domains in all SPL types derived from a representative member *Aegle marmelos*. The domain-specific conserved motifs are denoted on the top. **b** A higher degree of overall sequence conservation within a particular SPL type (SPL5 as a representative member) derived from the citrus relatives

Based on the studies in Arabidopsis (Guo et al. 2008; Xing et al. 2010), we divided the *miR156*-targeted citrus *SPL*s into two distinct subgroups- members with smaller peptides- represented by *SPL3* (*SPL3*, *SPL4,* and *SPL5*), and those with larger peptides- represented by *SPL9* (*SPL2*, *SPL6A*, *SPL6B*, *AtSPL9*, *SPL10*, *SPL11*, *SPL13A*, *SPL13B,* and *SPL15*). All SPL proteins harbored the 79 amino acid long signature domain comprising two zinc finger binding motifs (C3H or C4 and C2HC) and a bipartite nuclear localization signal (KR-X11-RRR/K) overlapped with the latter zinc finger (Figs. 1 and S2). Overall, sequences of each SPL-types were highly identical among most of the assessed species except for *A. marmelos*, the most distant relative taken in the study. Notably, *SPL8* and *SPL10* were located tandemly in a tail-to-tail fashion with just ~7 kb intermediary region in all species.

### *SPLs* exhibit distinct evolutionary patterns among citrus relatives

**Fig. 2:**
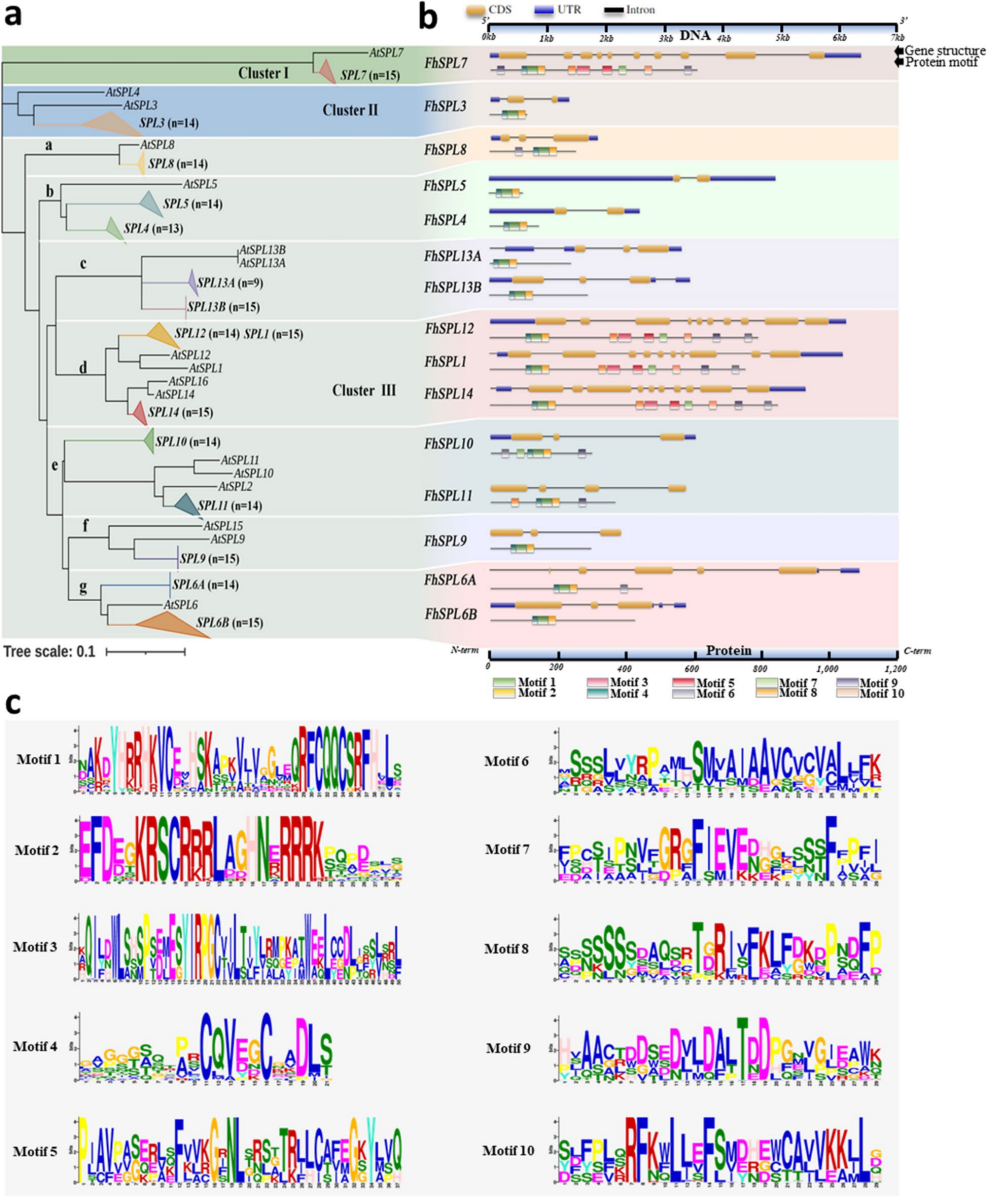
Evolutionary relationship, gene structure, and protein motif assessment of SPLs. **a** Neighbor-Joining phylogeny of the SBP domains of citrus-related species and Arabidopsis (1000 bootstrap, Poisson correction method). The area of each compressed sub-cluster is relative to the diversity of the compressed sequences. *n* represents the number of species harboring the respective *SPL* genes. **b** Gene structures (upper) and conserved protein motifs (lower) of each SPL type from a representative species *F. hindsii*. Blue squares, yellow squares, and black lines of the gene structure represent UTRs, exons, and introns respectively; each colored box in the SPL proteins represents a motif, and its size corresponds to the motif length. **c** Sequence information of the most conserved 10 protein motifs among the SPLs in b

The phylogenetic tree was developed from the SBP domains of the citrus-related species and Arabidopsis. Based on their clustering, we divided the *SPL*s into three groups- Cluster-I (constitutes *SPL7*s), Cluster-II (constitutes citrus *SPL3*s), and Cluster-III (constitutes all remaining *SPL*s) (Fig. 2a). Interestingly, some *SPL* orthologs have specifically been duplicated and diversified among citrus members as compared to their Arabidopsis counterparts which include *SPL4*/*SPL5* (close orthologs of *AtSPL5*), *SPL6A*/*SPL6B* (close orthologs of *AtSPL6*), and *SPL13A*/*SPL13B* (close orthologs of fully identical *AtSPL13A*/*AtSPL13B*) (Fig. 2a). Some *SPL* members showed reduced duplication among citrus-related species, which include *SPL3* (close ortholog of *AtSPL3*/*AtSPL4*), *SPL14* (close ortholog of *AtSPL14*/*AtSPL16*), *SPL11* (close ortholog of *AtSPL2*/*AtSPL10*/*AtSPL11*), and *SPL9* (close ortholog of *AtSPL9*/*AtSPL15*) (Fig. 2a). Among all, one *SPL* member, *SPL10*, appeared to be unique to the citrus-related species which was not clustered with any of the *AtSPL*s indicating its unique evolution at least among the citrus-related members. *SPL1* and *SPL12* were clustered together instead of clustering with their respective *AtSPL* orthologs suggesting for the independent duplication/diversification among *citrus*-related species and *Arabidopsis*. Interestingly, two *SPL* members, *SPL7* and *SPL8* were accompanied by their respective *AtSPL*s indicating for their evolutionary and functional conservation in *Arabidopsis* and *citrus* relatives.

### Gene *structure* and motif conservation assessment of the *SPL*s

To ascertain whether the phylogenetic relationship was also by the gene structure and protein motifs similarities, we assessed such features in all *SPL*s. As expected, the closely clustered *SPL*s, exhibited similar gene (exon/intron) structures and conserved motifs (Figs. 2b, S3, and S4). Of 15 *SPL*s, the *SPL1/SPL7/SPL12/SPL14* are the longest *SPL*s in both gene and peptide length. Among the most conserved 10 motifs predicted, motif4+motif1+motif2 encompassed the SBP domain, which was conserved in all SPLs as expected (Fig. 2b and c). Additionally, a C-terminally located motif-6 constitutes part of the protein sequence putatively involved in the transmembrane binding of the SPL1s, SPL7s, SPL12s, and SPL14s. It is notable that SPL6A too harbors motif-6, even though no transmembrane helix was predicted in it (Figs. 2b, c, and S5). While it is very plausible that the other six conserved motifs (motif3, motif5, motif7, motif8, motif9、motif10) play role in the proper functioning of the SPLs harboring them (all SPLs in our study), their functional relevance at this point is yet unclear.

### Cis-*regulatory* element (CRE) assessment

**Fig. 3:**
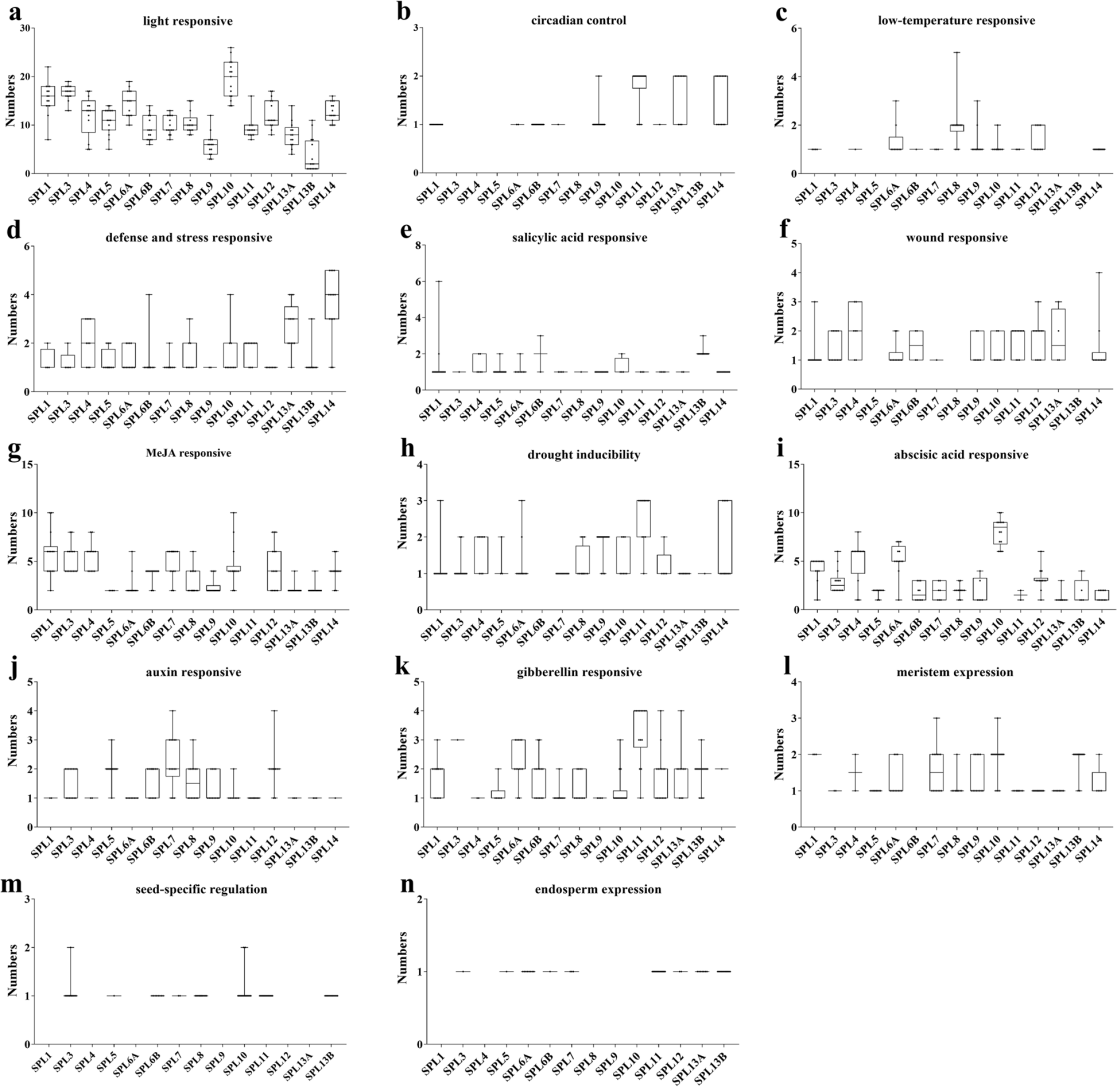
The number of cis-acting elements predicted on the promoter of SPL genes in 15 citrus-related species

SPL members are involved in various development processes. To have a general overview of their nature of expression, we fed the 2.0 kb regions upstream of the translation start site of each *SPL* to the PlantCARE database and retrieved potential transcription factor binding *cis*-element within. They were later visualized using TBtools (Figure S6) and boxplots (Fig. 3). Citrus *SPL* promoters harbor *cis*-elements responsive to light, methyl jasmonate, abscisic acid, auxin, salicylic acid, wound, drought, defense, gibberellin, low temperature, circadian rhythm, and other response factors. Among the predicted *cis*-elements those responsive to light were the most prevalent in all *SPL* promoters with *SPL10* and *SPL13* promoters harboring them at the highest and lowest number respectively (Fig. 3a). Interestingly, among all, *SPL11* and *SPL13* promoters harbor a relatively higher number (2) of circadian-associated *cis*-elements as compared to others (0-1) (Fig. 3b). Additionally, most of the *SPL* promoters harbor just a single temperature-responsive element except for that of *SPL8*. Promoters of *SPL3/5/13A/13B* promoters, on the other hand, constitute none (Fig. 3c).

Interestingly, the *SPL* promoters harbored very few *cis*-elements potentially responsive to either defense/stress, salicylic acid (SA), wound, methyl jasmonate (MeJA), and/or drought (Fig. 3d-h). While the majority of the *SPL* promoters harbored single auxin and gibberellin responsive elements, they constitute a relatively higher number of abscisic acid (ABA) responsive elements with the *SPL10* promoters harboring them in the highest number (Fig. 3i-k).Most of the *SPL* promoters harbor just a single *cis*-element each associated with the meristem, endosperm, and seed-specific regulation. However, *SPL6B* promoters lacked any meristem regulation related; *SPL1/4/8/9/10/14* lacked any endosperm regulation related, and *SPL1/4/6A/9/12/13A/14* lacked any seed-specific regulation related *cis*-elements (Fig. 3l-n). The functional relevance of the *cis*-regulatory elements in their expression is yet to be explored in citrus.

### Expression profiling of the *SPL* orthologs in citrus members

**Fig. 4:**
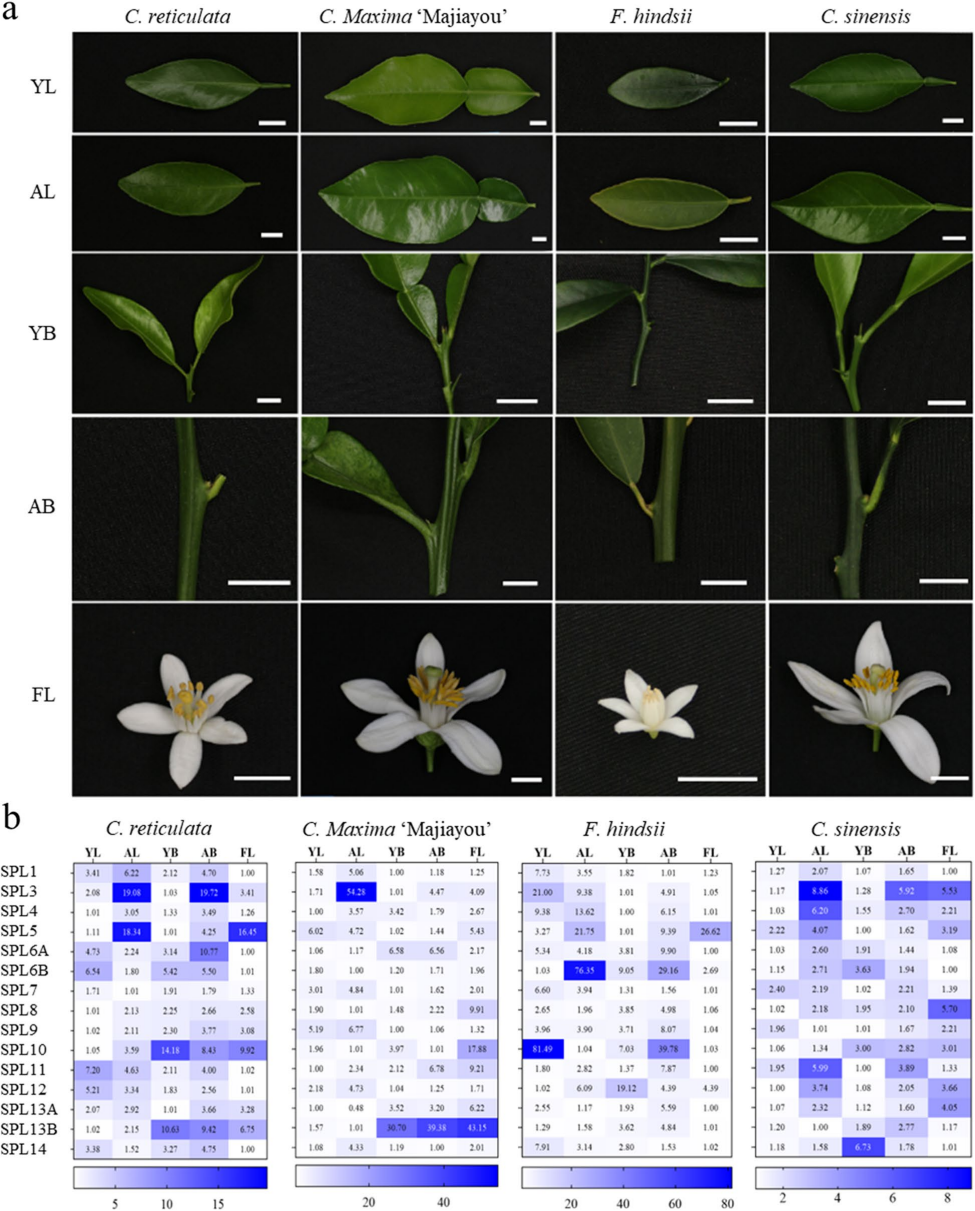
*SPL* orthologs exhibit species-specific expression variation. **a** Different organizations. the bars represent 1cm. **b** Gene expression detection. YL, juvenile leaf; AL, adult leaf; YB, juvenile bud; AB, adult bud; FL, fully open flower; The darker the blue, the higher the expression level. Values represent relative expression levels. Three biological replicates were conducted for each group of experiments, and the expression level of *Actin* was used as an internal control

To assess which of the *SPL*s exhibit uniquely different expression patterns in young and adult tissues (buds and leaves) as well as in flowers of four representative members of the cultivated citrus species, namely *C. reticulata*, *C. maxima* ‘Majiayou’, *F. hindsii*, and *C. sinensis*, and we detected the expression level of *SPLs* gene through Real-time quantitative PCR. Some of the representative *SPL*s relatively highly expressed in adult leaves as compared to young leaves include *C. sinensis* (*CsSPL3*, *CsSPL4*, *CsSPL11,* and *CsSPL12*), *Fortunella hindsii* (*FhSPL5*, *FhSPL6A,* and *FhSPL12*), *C. maxima* ‘Majiayou’ (*CmjSPL1*, *CmjSPL3*, *CmjSPL4,* and *CmjSPL14*) and *C. reticulata* (*CrSPL3*, *CrSPL4,* and *CrSPL10*). Notably, *FhSPL6B* and *CmjSPL14* exhibited 74- and 31 times higher expression respectively in adult leaves as compared to their young counterparts. Similarly, the *SPL*s exhibiting relatively higher expression at the adult buds as compared to the young ones include *C. sinensis* (*CsSPL3* and *CsSPL11*), *F. hindsii* (*FhSPL3, FhSPL4, FhSPL5, FhSPL6B, FhSPL10* and *FhSPL11*), *C. maxima* ‘Majiayou’ (*CmjSPL3* and *CmjSPL11*) and *C. reticulata* (*CrSPL3*, *CrSPL5*, *CrSPL6A,* and *CrSPL13A*). *CrSPL3*, in particular, was up-regulated 19 times higher in the adult buds as compared to the young ones. Furthermore, *SPL5* was relatively highly expressed in the flower tissues of four species (Fig. 4).

Since the change in meristem identity is crucial to flowering in plants, we opted to screen the *SPL*s exhibiting relatively higher expression in adult buds as compared to their young counterparts.

While several *SPL* genes were up-regulated in buds of different citrus members, *SPL3* and *SPL11* were up-regulated in all four species, and *SPL4*, *SPL5*, and *SPL9* were up-regulated in all but Majiayou. Adult leaves, on the other hand, exhibited a higher level of *SPL4* expression in all four species. Overall, *SPL*s of *F. hindsii* exhibited the widest, those of *C. sinensis* exhibited the narrowest, and those of the remaining two species exhibited intermediary expression variations (closer to *C. sinensis*) (Fig. 4). Among all, we selected *SPL5* (exhibiting higher expression in adult leaves of the majority of the species), *SPL9* (exhibiting higher expression in adult buds of the majority of the species), and *SPL11* (exhibiting higher expression in adult buds of all species) from Kumquat to assess their florigenic potential.

### *Independent* overexpression of *FhSPL5*, *FhSPL9* and *FhSPL11* induced precocious flowering in Arabidopsis

**Fig. 5:**
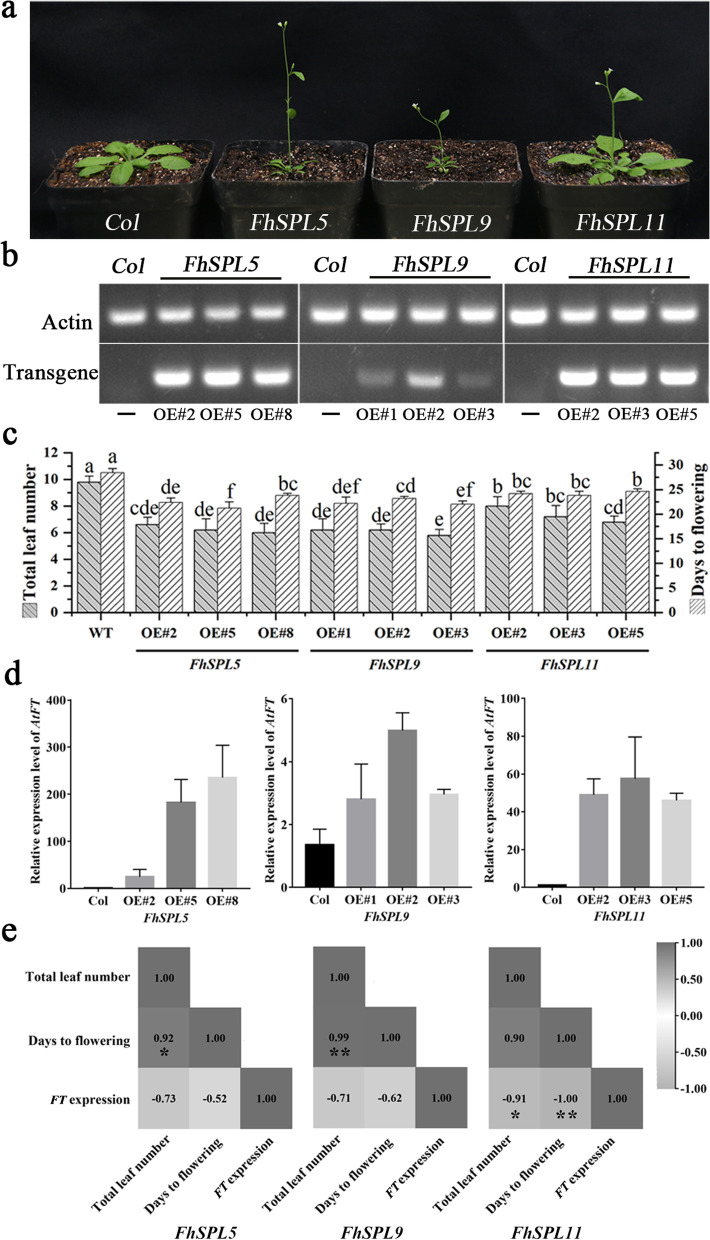
Citrus *SPL*s exhibit cross-species functional conservation. **a** Representative images of WT and respective transgenic lines of *FhSPL5*, *FHSPL9*, and *FhSPL11* at 3 weeks after germination. **b** Confirmation of the ectopic *FhSPL*-overexpressing transgenic Arabidopsis lines; **c** Quantitative *FT* expression analysis showed that all *FhSPL* transgenic lines exhibited its higher expression with the *FhSPL5* transformants showing the highest and that of *FhSPL9* showing the lowest. **d** All *SPL*-transgenic lines flowered at a significantly short time as compared to WT and the transgenic lines constituted a significantly lesser number of rosette leaves. The groups (or lines) with the same letter above bars were not statistically significantly different (Student’s T-test*, p* ≤ 0.05). **e** Correlation of endogenous *FT* expression (*ΔΔC*_*t*_-value) to leaf number and flowering time in the transgenic *Arabidopsis thaliana* (single-tailed T-test; *p ≤ 0.05*)

The expression of plant *SPL* is generally regulated by upstream *miR156*. Therefore, we predicted its target site within the sequence of SPL genes of *F. hindsii*. The results showed that FhmiR156 targeted the 5’-UTR region of *FhSPL5*(Fh4g16520) and the CDS region of *FhSPL9*(Fh6g17020) and *FhSPL11*(Fh7g25680) (Table S3). We constructed independent overexpression vectors of *FhSPL5*, *FhSPL9*, and *FhSPL11* using respective CDS (Table S4) after synonymous mutations of miR156 target sites of *FhSPL9* and *FhSPL11*. Three independent positive transgenic lines of each gene were selected and grown to their T3 generation through subsequent progeny selection in the selection media (Fig. 5a). The transgenic lines were confirmed by RT-qPCR of ectopically expressed *FhSPL*s (Fig. 5b).

The results showed that the average flowering time of the wild type was about 28.4 days after sowing, and the average number of rosette leaves was 9.8 (Fig. 5c). The transgenic lines overexpressing *FhSPL5* had a significantly reduced flowering time of 21.2-23.8 days and a rosette leaf number of 6.0-6.2 (Fig. 5c). Similar observations were made for the overexpression lines of *FhSPL9* (22.0-23.2 days of flowering time; 5.8-6.2 number of rosette leaves) and *FhSPL11* (23.8-24.6 days of flowering time and 6.8-8.0 number of rosette leaves) (Fig. 5c). We further confirmed that the overexpression of *FhSPL*s led to the upregulation of endogenous *AtFT*(), a crucial gene involved in floral induction, and assessed its correlation with the precocious flowering in the transgenic Arabidopsis lines. Even though all transgenic lines exhibited a negative correlation of *FT* expression to days-to-flowering and total leaf number, it was statistically significant only for the *FhSPL11* transgenic lines (Fig. 5d). The data showed a relatively stronger flowering promotion effect of *SPL5* and a very similar effect of *SPL9* as compared to *SPL11* in Arabidopsis. An earlier study by Shalom et al. (2015) with the *C. clementina* derived *SPL5* (annotated as *CiSPL5* in the study) comes in agreement with our observation. Gene sequence analysis revealed that SPL5 is a protein composed of only 131 amino acids. In combination with the expression data (Fig. 4), it is very likely that *SPL5* plays a positive role in shortening juvenility and/or promoting flowering in citrus.

## Discussion

### Characterization of SPL family in citrus-related species

A total of 221 *SPL* genes have been identified among 15 citrus-related species in this study. Gene duplication followed by their diversification and/or neofunctionalization has often been regarded as the driver behind *SPL* multiplication in plants (Guo et al. 2008; Ren et al. 2022). Our assessment showed that certain *SPL*s (*SPL4/5*, *SPL13A/13B*, and *SPL6A/6B*) have been specifically multiplied and diversified among citrus-related species as compared to those in Arabidopsis (*AtSPL5*, *AtSPL13*, and *AtSPL6* respectively) suggesting for their functional diversification among citrus related species. However, citrus members harbor a relatively lesser number of *SPL*s (15) as compared to Arabidopsis (17). The infrequent reproductive cycle caused by gametic sterility, apomixis, and vegetative propagation in combination with their long juvenility in citrus has been suggested to be part of the reason (Xu et al. 2013; Zeng et al. 2019). Some citrus *SPL*s shared relatively higher sequence similarities and were tightly clustered with each other (*SPL1*/*SPL12*, *SPL6A*/*SPL6B*, and *SPL13A*/*SPL13B*), to which earlier study proposed for their segmental duplication during the evolutionary process (Zeng et al. 2019). The study further argued for the occurrence of a more recent whole genome duplication event, which was not found in citrus, as a likely contributor behind the relatively higher levels of expansion of *SPL* numbers in plants like Arabidopsis (17), poplar (30), apple (30), etc. Our study has shown that the *SPL* number has been fixed among the citrus-related species, although one wild relative, *M. paniculata*, had a loss of functional *SPL14* ortholog.

The phylogenetic tree developed from the SBP domains of the respective SPLs showed that the members with relatively similar gene structure and conserved motif patterns had been clustered together, which was partly corroborated by the studies of Song et al. (2021), Zeng et al. (2019) Shalom et al. (2015), and Liu et al. (2017) on independent citrus species. We additionally found that four of the miR156 non-targeted SPLs (SPL1, SPL7, SPL12, and SPL14) harbor transmembrane domains near their C-termini. While the studies in orthologous species suggest their crucial role in SPL localization and/or function (Stone et al. 2005; Chao et al. 2017; Yang et al. 2022), their functional relevance in citrus species itself has not been reported yet.

### *Evolutionary* diversification and functional relevance of citrus SPL genes

In Arabidopsis, 12 out of 17 *SPL*s are targeted by miR156 (Cardon et al. 1999; Rhoades et al. 2002; Xu et al. 2016). Our assessment showed that such a feature is conserved among the assessed member species as well. Like in Arabidopsis, except for *SPL1*, *SPL7*, *SPL8*, *SPL12*, and *SPL14*, all other *SPL*s harbor miR156-recognition sites in citrus-related species (Table 1). This comes in agreement with earlier reports on *C. clementina* (Shalom et al. 2015; Zeng et al. 2019), *C. sinensis* (Liu et al. 2017). *F. hindsii* (Long et al. 2018), etc. Furthermore, except for the *SPL8*, all other miR156 non-targeted SPLs are the ones with the highest exon number (10) and the longest gene/peptide size.

### SPLs lacking miR156 target site

Each citrus-related member harbored a uniquely single *SPL* ortholog, which clustered with *AtSPL7* (Fig. 2a). Studies have reported the involvement of this miR156 target site lacking *SPL* in copper deficiency response, which depends on the cleavage of its C-terminally located transmembrane domain followed by its nuclear transport (Garcia-Molina et al. 2014; Schulten et al. 2019) suggesting for similar fate and role of *SPL7* in citrus members.miR156 target site lacking *SPL8* citrus orthologs were clustered uniquely with the *AtSPL8*. Studies in Arabidopsis vaguely indicate that it may play a positive role in the pollen-specific brassinosteroid signaling during anther sac formation and pollen development in coordination with *miR156*-regulated *SPL*s (Unte et al. 2003; Xing et al. 2013). Whether citrus orthologs are involved in a similar role is yet unclear. Interestingly, a study in *C. clementina* showed that its ortholog is upregulated in the buds upon de-fruiting (Shalom et al. 2015). Heavy fruit load is known to inhibit flowering, the removal of which promotes the process in the subsequent season (Muñoz-Fambuena et al. 2012). A More recent study by Zeng et al. (2019) showed that the *SPL8* ortholog in *C. clementina* (annotated as *CclSBP7*) uniquely transcribed three splice variants (*α*, *β*, and *γ*), overexpression of which brought precocious flowering in Arabidopsis by elevating the expression of *FT*, *FRUITFULL* (*FUL*), *APETALA1* (*AP1*), and *LEAFY* (*LFY*). Additionally, the transgenic lines produced relatively shorter siliques (Cao et al. 2019; Zeng et al. 2019). An earlier study in Arabidopsis reported that *AtSPL8* plays a localized tissue-specific role in response to gibberellin (GA) signaling (Zhang et al. 2007). Hence, it requires further assessment of the florigenic potential of the *miR156* non-targeted *SPL8* in citrus.

Additional miR156 non-targeted members, *SPL1* and *SPL12* citrus orthologs were clustered together- close to *AtSPL1*/*AtSPL12*. Like their Arabidopsis orthologs, all but *CrSPL12* harbored putative transmembrane binding domain near respective C-termini. An Arabidopsis study reported their involvement in thermotolerance at the reproductive stage (Chao et al. 2017). Furthermore, a study in rice showed that its *SPL12* ortholog, *OsSPL6* is crucial for the suppression of ER stress conditions thereby avoiding cell death in developing panicles (Wang et al. 2018). An earlier study in *C. clementina* by Zeng et al. (2019) also suggested their (annotated as *CclSBP5* and *CclSBP14* in the study) involvement during drought stress response and floral induction in citrus based on their expression profile. A study in *Tamarix chinensis* also suggests the involvement of the *SPL1/12* as well as *SPL14* orthologs in coping with salt stress, as the study showed their elevated expression of salt stress (Wang et al. 2019). *SPL14* orthologs were clustered close to *AtSPL14/16* in our study. Its positive involvement in juvenile phase extension and sensitivity to the fungal toxin fumonisin B1 had been reported in Arabidopsis (Stone et al. 2005). Based on their expression profile change upon draught treatment, Zeng et al. (2019) suggested the involvement of *SPL14* (annotated as *CclSBP12* in the study) in the revival of vegetative growth in *C. clementina*. It requires further assessment to conclude the potential involvement in stress response and juvenility in citrus.

### SPLs harboring miR156 target sites

miR156 harboring *SPL*s are attributed to their involvement in plant development in an age-dependent manner and are of particular interest to flowering-related studies. Among the 10 miR156-targeted citrus *SPL*s, *SPL10* was the only sub-clade that was not clustered with any Arabidopsis orthologs. An orthologous transgenic study of the *C. clementina* derived *SPL10* (annotated as *CclSBP6* in the study) showed that its ectopic overexpression significantly delays flowering time in Arabidopsis plants due to the reduced expression of *FT* and *SPL2*/*3*/*4*/*5*/*9*. The overexpression lines additionally exhibited dwarf growth, slender leaves, smaller flowers, shorter siliques, and longer root phenotypes under long-day conditions. Due to the similarity of the transgenic plants to the miR156 overexpression lines, the study suggested the potential of *SPL10* being not targeted by miR156 (Zeng et al. 2019). It requires additional study in citrus to conclude the regulation of this uniquely citrus-specific *SPL* and its potential role in citrus juvenility.

*SPL13*, which is present in two identical copies in Arabidopsis and is reportedly involved in the negative regulation of the transition from cotyledonary to vegetative leaf stage (Martin et al. 2010), is present in pair (*SPL13A* and *SPL13B*) in citrus. A study in Arabidopsis showed that its overexpression leads to the stunted growth and formation of distorted branches, while its silencing delays flowering (Gao et al. 2018). Tomato plants transformed with its *SPL13* ortholog exhibit precocious flowering (Cui et al. 2020). A study in kumquat reported that overexpression of csi-miR156a or independent knock-down of *SPL13B* and *SPL5* (annotated as *CsSPL14* and *CsSPL3* respectively in the study) significantly enhanced the somatic embryogenesis competence of its callus (Long et al. 2018). Their more recent study suggested the reduced starch accumulation led by the overexpression of starch biosynthesis gene repressors, *TOE1.1* and *TOE1.2* in the miR156 repressed citrus calli as one of the key factors behind their lower SE efficiency (Feng et al. 2022). Their finding came close to an earlier study by Liu et al. (2017), which reported increased starch content in the miR156 overexpressed citrus calli with a higher degree of downregulation of *SPL10* and *SPL13B* (annotated as *CsSPL2* and *CsSPL14* in the study). These studies link the decline in embryogenetic potential with maturity often observed in plants (Isah 2016). Our expression assessment showed that both versions of *SPL13* exhibit their higher expression at the buds in all of the species except for *C. sinensis*. However, the transcripts of *SPL13A* were much higher in the adult buds while that of *APL13B* were almost at the similar level in both young and adult buds suggesting for their functional differences at the apical meristems. It requires further study to conclude their potential role in SAM fate determination.

Citrus-related species additionally harbor *SPL6* in pairs (*SPL6A* and *SPL6B*). Studies in other species suggest their involvement in defense-related gene activation *in planta* (Padmanabhan et al. 2013). A study in *C. sinensis* showed that the expression of all *SPL*s decreases with time after infection with *Diaporthe citri*. However, *SPL6B* (annotated as *CsSBP6* in the study) exhibits a relatively lesser degree of decrease (Song et al. 2021). It is also notable that unlike herbaceous plants like Arabidopsis and rice, tree species like citrus, apple, poplar, hybrid cherry tree (*Prunus* × *yedoensis*), etc. have the duplication and diversification of *SPL6* orthologs (Li et al. 2013; Zeng et al. 2019; Gao et al. 2022). While it is plausible, it requires further assessment to elaborate on the potential involvement of these *SPL*s in pathogen defense/resistance and the mechanism behind it.

A study in apple reported that *MdSPL2* (close ortholog of *CsSPL13B*) and *MdSPL33* (close ortholog of *CsSPL9*) promote anthocyanin accumulation in fruit peel by hindering the suppressive effect of miR156 mediated by lncRNAs (MLNC3.2 and MLNC4.6) (Yang et al. 2019). Contrastingly, a transgenic study in poplar showed that the miR156 overexpressing lines exhibits reduced abundances of *SPL8/11/12/17/28/29* and higher accumulation of anthocyanin in its shoot (Wang et al. 2020). Earlier independent studies in Arabidopsis (Gou et al. 2011) also reported the negative regulation of anthocyanin accumulation by a miR156 targeted *AtSPL9* in its stem. A study in blueberry showed similarly reduced expression of its six miR156 targeted *SPL*s in ripe whole fruit. The abundance of the SPLs would have otherwise repressed the expression of *DFR*, a gene responsible for anthocyanin biosynthesis (Li et al. 2021). Whether *SPL*-dependent anthocyanin accumulation is species-dependent or tissue-specific is not fully clear yet. Moreover, the anthocyanin content of many citrus fruit changes with their maturity both inside and out. It is likely but needs substantial evidence to conclude its association with the *SPL* expression in the plants.

Studies in Arabidopsis suggest the involvement of *SPL3*, *SPL4*, and *SPL5* in integrating age pathway to the photoperiod and GA signals to promote flowering (Jung et al. 2012; Jung et al. 2016). They are involved in floral meristem promotion (Xu et al. 2016). They redundantly interact with FD and facilitates/enhances its binding to the promoters of *AP1*, *LFY*, and *FUL* promoters, which gradually increase with plant age (Jung et al. 2016). A study in *C. clementina* showed that fruit load represses the expression of some *SPL*s and the removal of fruits leads to their increased expression at buds. Those *SPL*s include *SPL5* (annotated as *CiSPL5* in the study), *SPL10,* and *SPL8* (Shalom et al. 2015). *SPL9*, on the other hand, plays role in integrating GA-signaling into the age pathway to promote shoot maturation (Schwarz et al. 2008), juvenile-to-adult phase transition (Yu et al. 2012; Hyun et al. 2016), axillary bud formation (Zhang et al. 2020), and flowering (Wang et al. 2009). The *SPL11* orthologs, *AtSPL2/10/11* reportedly regulate shoot maturation and proper development of the lateral organs in the reproductive phase in Arabidopsis. They additionally suppress root regeneration with age by binding to *AP2/ERF* promoters (Ye et al. 2020) and are partly involved in phase transition by regulating *FUL* (Shikata et al. 2009). A recent study in *C. clementina* showed that SPL11 (annotated as CiSPL11 in the study) positively regulates the expression of *CiKN6*, a citrus homolog of *KNOTTED1-LIKE HOMEOBOX* (*KNOX*) family genes, by binding to its promoter. Furthermore, the study showed that CiKN6 complexes with CiKN1, which in turn suppresses the expression of *miR164a* by binding to its promoter and modulates leaf development in citrus (Zeng et al. 2022). In the current study, based on their relatively unique tissue expression pattern, we chose *SPL5*, *SPL9*, and *SPL11* orthologs from Kumquat for their functional assessment.

### *Confirmation* of the florigenic potential of selected *SPL*s

An earlier study by Zhu et al. (2019) showed that *FhSPL5*, *FhSPL8*, *FhSPL10*, and *FhSPL3* (annotated as *FhSPL1*, *FhSPL7*, *FhSPL8*, and *FhSPL9* in their study) exhibited expression correlation with the flowering related genes and suggested for their functional redundancies in floral induction. It should be noted that Zhu et al. (2019) reported higher number of *FhSPL* members earlier, most likely due to the less refined genome data used in the study. In the current study, we assessed the expression profiles of the *SPL*s in young and adult leaves and buds as well as flowers of four different species. Based on our recent assessment, we selected *FhSPL5* (expressed in flower buds at a relatively higher degree), *FhSPL9*, and *FhSPL11* (both expressed relatively highly in adult buds) for their florigenic potential assessment. Their independent overexpression led to significantly precocious flowering in Arabidopsis confirming their promotional role in floral induction (Fig. 5). An earlier study by Shalom et al. (2015) with the *C. clementina* derived *SPL5* (annotated as *CiSPL5* in the study) comes in agreement with our observation. Their study additionally reported that the overexpression of *CiSPL5* with an intact miR156 target site brought only a slight change to the flowering time. Our study showed that, among the three, *FhSPL5* overexpression led to the highest endogenous FT expression in plants. The floral precocity effect of *FhSPL5* is likely more direct than those of *FhSPL9* and *FhSPL11* since their Arabidopsis orthologs are reportedly involved in the regulation of flowering time, phase change/axillary bud formation, and shoot maturity respectively (Shikata et al. 2009; Hyun et al. 2016; Jung et al. 2016). However, it requires further experimental evidence in citrus to conclude as such for the citrus orthologs.

### *Conclusion* and perspective

Flowering in a majority of citrus species requires a long period of juvenility after germination, which has remained to be a serious hindrance to researchers and farmers alike. The phase transition process in a plant is often linked to the reciprocal change in the abundance of miR156 and miR172. Several studies have shown that such a process is coordinated by the miR156 targeted *SPL*s. Our current study has methodically assessed and freshly annotated such *SPL*s from 15 different citrus-related species. We confirmed that most of them harbor a full set of 15 *SPL* members. *Cis*-regulatory element assessment of the *SPL* promoters suggested the involvement of the gene in the diverse developmental and physiological process *via* their responsive expression to hormones, defense/stress, wound, light/temperature, etc. We found that the expression profile of *SPL*s among different species varies significantly. Interestingly *FhSPL5* showed higher expression at the flower buds and *FhSPL9/FhSPL11* exhibited higher expression at adult buds (compared to young) of all species assessed. Their independent overexpression in Arabidopsis brought precocious flowering by upregulating the endogenous *FT* expression. Our report is the first to document such an occurrence with the ectopic expression of citrus-derived *SPL9*/*SPL11*. Being a woody species, the observations made on the Arabidopsis could still be suggestive for the citrus species. Future study on the species is expected to conclude the observations and predictions made in the current study.

## Materials and methods

### Plant materials

Seeds were collected from the mature fruits of *C. reticulata*, *C. maxima* ‘Majiayou’, *F. hindsii*, and *C. sinensis*. They were incubated at 28℃ for a week to accelerate the germination and moved to the greenhouse for growth until next spring. Samples were collected when the seedlings were about 5 months old. The leaves were sampled from the fully unfolded leaves near the top (juvenile leaf, YL), and the buds were sampled from the top buds and three additional buds below (juvenile bud, YB). The adult bud (AB) was sampled at the stage before physiological differentiation, adult leaf (AL) was sampled from the fully unfolded leaf near the bud, and the flower (FL) was sampled of the fully open flower. Three biological replicates are selected for each group of samples. All citrus varieties came from the germplasm resource nursery at Huazhong Agricultural University (Wuhan, China). For the *SPL* expression assessment, the leaves, buds, and flowers of adult trees, as well as the leaves and buds of young trees, were collected. They were frozen in liquid nitrogen after sampling and stored at - 80 ℃ until use.

### Identification of SPL gene family members

A total of 15 citrus-related species (2n) were selected as the representative Aurantioideae members for the study- *Aegle marmelos*, *Murraya paniculate*, *Atalantia buxifolia*, *Clausena lansium*, *Citropsis gilletiana*, *Poncirus trifoliata*, *Fortunella hindsii*, *Citrus mangshannensis*, *Citrus ichangensis*, *Citrus sinensis*, *Citrus reticulata* ‘Pokan’, *Citrus hongheensis*, *Citrus maxima* ‘Majiayou’, *Citrus maxima* ‘Zipi’, and *Citrus medica*). Most of their genomes are included in the local Citrus Pan-genome to Breeding database (CPBD; http://citrus.hzau.edu.cn/). The hidden Markov model (HMM) and Basic Local Alignment Search Tool (BLAST) were used to screen the SPL gene in their respective genomes. The protein family database (Pfam) derived SBP domain profile PF03110, was used during the process (Finn et al. 2014). Arabidopsis*-*derived amino acid and nucleotide sequences of its 16 *SPL* genes were retrieved from the TAIR database (https://www.arabidopsis.org/). Homologous citrus *SPL*s were assessed with blastp. The *SPL* number was determined based on the results of HMM and blastp followed by their nomenclature based on the closest Arabidopsis *SPL*s.

### Phylogenetic Analysis

To study the evolutionary relationship between SPLs of different *citrus* varieties and members of the SPL gene family in Arabidopsis, we took their respective SBP domain to construct the phylogenetic trees. Multiple alignment was carried out using ClusterW in MEGA7.0, and an evolution tree was prepared using the Neighbor-Joining (NJ) method with default parameters (bootstrap = 1000). The iTOL was used (https://itol.embl.de/) for the curation of the developed phylogenetic tree.

### Analysis of gene structure, motif, and cis-acting elements

The gene structure (exon and intron composition) of *SPL* genes among 15 citrus-related species was assessed using GSDS 2.0 (http://gsds.gao-lab.org/) (Hu et al. 2014). Respective protein motifs were predicted using the MEME Suite (https://meme-suite.org/meme/index.html) with default parameters (for a maximum of 10 motifs) (Bailey et al. 2015). The gene structure and motif data were visualized using TBtools (Chen et al. 2020). To compare and analyze the *cis*-acting elements of the *SPL* promoters, 2.0 kb long promoter sequences of respective *SPL*s were retrieved, and the *cis-*elements were predicted using PlantCARE (http://bioinformatics.psb.ugent.be/webtools/plantcare/html/) (Lescot et al. 2002). GraphPad Prism 9 was used to prepare the boxplots of major *cis*-elements.

### RNA extraction and real-time quantitative PCR

The earlier protocol described by He et al. (2022) was used for total RNA extraction from the plant samples, their reverse transcription, and real-time quantitative PCR. Actin was used as an internal control and each sample was subjected to three repeats. The primers used for the quantitative PCR have been provided in Supplementary Table S5.

### Transgenic plant regeneration and analysis

According to the prediction made for the miR156 target site, *FhSPL5* harbor it at its 5’-UTR, while *FhSPL9* and *FhSPL11* harbor such site within their CDS region (Table S4, Figure S7). After synonymous mutations of *FhSPL9* and *FhSPL11* miR156-target sites, respective CDS were incorporated into pK7WG2D overexpression vector. The plasmid was transformed into *Agrobacterium* strain GV3101, which was then used for the transformation of *Arabidopsis* (Col-0) using the floral dip method as described by reference (He et al. 2022). T3 transgenic plants were generated *via* antibiotic resistance screening. *Arabidopsis* flowering time and rosette leaf number were used for the statistical analysis as described by reference (Zeng et al. 2019).

### Supplementary Information

**Additional file 1: **The online version contains supplementary material available at (web address will be provided by the publisher). **Supplementary Fig. S1.** Prediction of target sites for miR156 in SPL. **Supplementary Fig. S2.** SPL conserved domain sequence alignment. **Supplementary Fig. S3.** Gene structure. **Supplementary Fig. S4.** SPL-motifs prediction. **Supplementary Fig. S5.** Nuclear localization prediction. **Supplementary Fig. S6.** Cis-acting element pred by PlantCARE + TBTOOLS. **Supplementary Fig. S7.** FhSPL9 and FhSPL11 Mutation Sites. **Supplementary Table S1.** The characteristics of identified SPL genes in Citrus. **Supplementary Table S2.** Table S2. Prediction of miR156 and SPL target sequences. **Supplementary Table S3.** Prediction of miR156 target genes in *Fortunella hindsii. ***Supplementary Table S4.** Vector construction and quantitative primers for gene expression detection. **Supplementary Table S5.** Quantitative PCR primers for SPL genes of *Fortunella hindsii*, *Citrus sinensis*, *Citrus reticulata* ‘Pokan’ and *Citrus maxima*‘Majia’.

## Data Availability

The materials are available.

## Abbreviations

Ab: *Atalantia buxifoliata*
AB: Adult bud
ABA: Abscisic acid
AL: Adult leaf
Am: *Aegle marmelos*
AP1: Apetala 1
At: Arabidopsis;
BLAST: basic local alignment search tool
Cg: *Citropsis gilletiama*
Ch: *Citrus hongheensis*
Cic: *Citrus ichangensis*
Cl: *Clausena lansium*
Cm: *Citrus medica*
Cmj: *Citrus maxima* ‘Majiayou’
Cms: *Citrus mangshannensis*
CO: Constans
Col-0: Arabidopsis
CPBD: Citrus Pan-genome to Breeding Database
CRE: Cis-regulatory element
Cs: *Citrus sinensis*
Cr: *Citrus reticulate*
Czp: *Citrus maxima* ‘Zipi’
Fh: *Fortunella hindsii*
FL: Fully open flower
FT: Flowering locus T
FUL: Fruitfull
GA: Gibberellin
HMM: Hidden Markov model
KNOX: Knotted1-like homeobox
LFY: Leafy
MeJA: Methyl jasmonate
Mp: *Murraya paniculate*
NJ: Neighbor-Joining
OE: Overexpression
Pfam: Protein family database
Pt: *Poncirus trifoliate*
SA: Salicylic acid
SOC1: Suppressor of overexpression of constans 1
SPL: Squamosa promoter binding proteins (SBP) and SBP-like
YB: Juvenile bud
YL: Juvenile leaf
