# Supplementary material for: Evolutionary assessment of SQUAMOSA PROMOTER BINDING PROTEIN-LIKE genes in citrus relatives with a specific focus on flowering
Source: Mol Hortic. 2023 Jul 20;3:13. doi: 10.1186/s43897-023-00061-4 (PMC10515035; doi:10.1186/s43897-023-00061-4)
Supplement: Supplementary file 1 — Additional file 1: The online version contains supplementary material available at (web address will be provided by the publisher). Supplementary Fig. S1. Prediction of target sites for miR156 in SPL. Supplementary Fig. S2. SPL conserved domain sequence alignment. Supplementary Fig. S3. Gene structure. Supplementary Fig. S4. SPL-motifs prediction. Supplementary Fig. S5. Nuclear localization prediction. Supplementary Fig. S6. Cis-acting element pred by PlantCARE + TBTOOLS. Supplementary Fig. S7. FhSPL9 and FhSPL11 Mutation Sites. Supplementary Table S1. The characteristics of identified SPL genes in Citrus. Supplementary Table S2. Table S2. Prediction of miR156 and SPL target sequences. Supplementary Table S3. Prediction of miR156 target genes in Fortunella hindsii. Supplementary Table S4. Vector construction and quantitative primers for gene expression detection. Supplementary Table S5. Quantitative PCR primers for SPL genes of Fortunella hindsii, Citrus sinensis, Citrus reticulata ‘Pokan’ and Citrus maxima‘Majia’. [file 43897_2023_61_MOESM1_ESM.zip › Figure S2 SPL conserved domain sequence alignment.pdf]

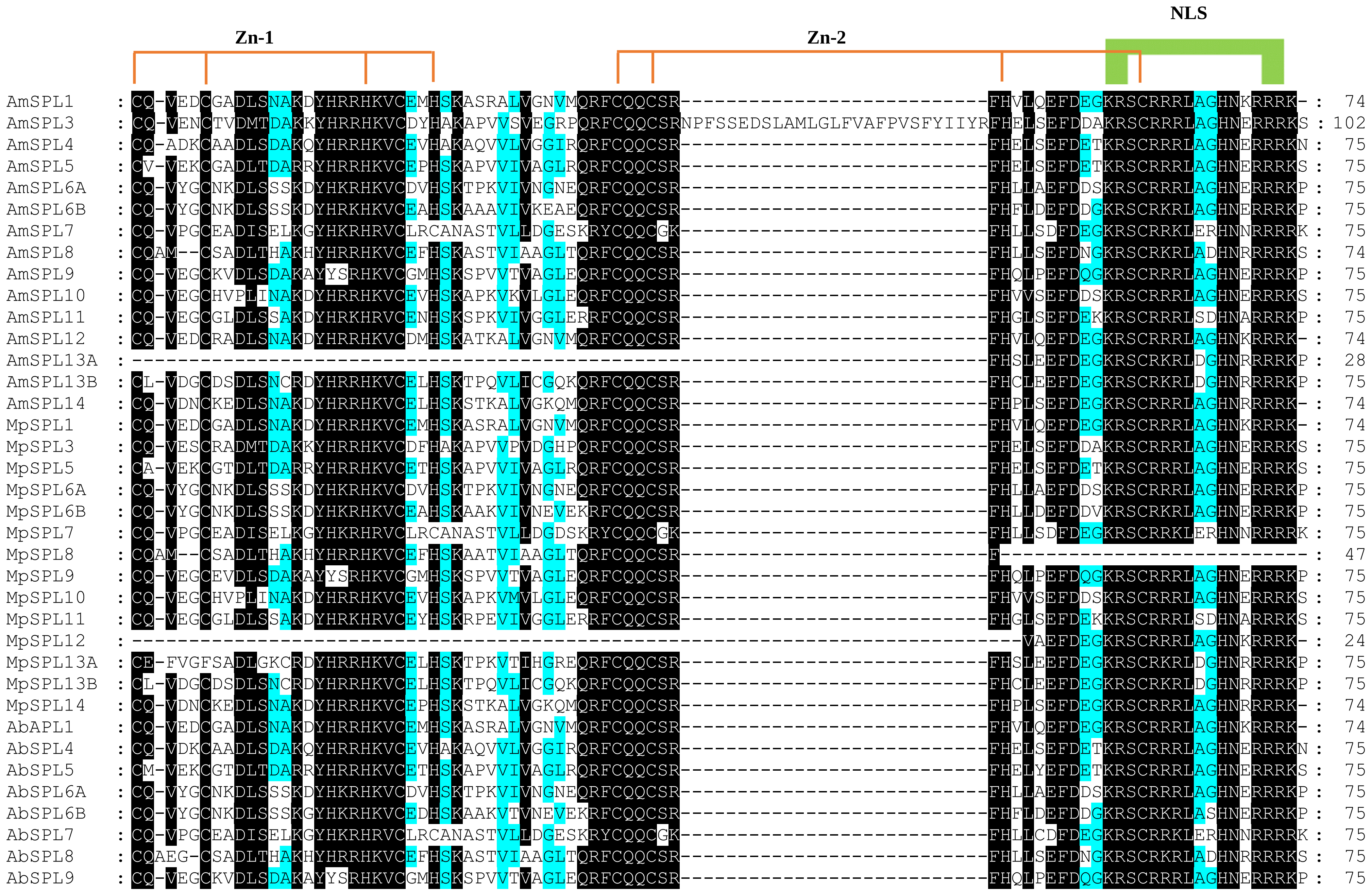

NLS
Zn-1
Zn-2
AmSPL1 : CQ-VEDCGADLSNAKDYHRRHKVCEMHSKASRALVGNVMQRFCQQCSR---------------------------FHVLQEFDEGKRSCRRRLAGHNKRRRK- : 74
AmSPL3 : CQ-VENCTVDMTDAKKYHRRHKVCDYHAKAPVVSVEGRPQRFCQQCSRNPFSSEDSLAMLGLFVAFPVSFYIIYRFHELSEFDDAKRSCRRRLAGHNERRRKS : 102
AmSPL4 : CQ-ADKCAADLSDAKQYHRRHKVCEVHAKAQVVLVGGIRQRFCQQCSR---------------------------FHELSEFDETKRSCRRRLAGHNERRRKN : 75
AmSPL5 : CV-VEKCGADLTDARRYHRRHKVCEPHSKAPVVIVAGLRQRFCQQCSR---------------------------FHELSEFDETKRSCRRRLAGHNERRRKS : 75
AmSPL6A : CQ-VYGCNKDLSSSKDYHKRHKVCDVHSKTPKVIVNGNEQRFCQQCSR---------------------------FHLLAEFDDSKRSCRRRLAGHNERRRKP : 75
AmSPL6B : CQ-VYGCNKDLSSSKDYHRKHKVCEAHSKAAAVIVKEAEQRFCQQCSR---------------------------FHFLDEFDDGKRSCRKRLAGHNERRRKP : 75
AmSPL7 : CQ-VPGCEADISELKGYHKRHRVCLRCANASTVLLDGESKRYCQQCGK---------------------------FHLLSDFDEGKRSCRRKLERHNNRRRRK : 75
AmSPL8 : CQAM--CSADLTHAKHYHRRHKVCEFHSKASTVIAAGLTQRFCQQCSR---------------------------FHLLSEFDNGKRSCRKRLADHNRRRRKS : 74
AmSPL9 : CQ-VEGCKVDLSDAKAYYSRHKVCGMHSKSPVVTVAGLEQRFCQQCSR---------------------------FHQLPEFDQGKRSCRRRLAGHNERRRKP : 75
AmSPL10 : CQ-VEGCHVPLINAKDYHRRHKVCEVHSKAPKVKVLGLEQRFCQQCSR---------------------------FHVVSEFDDSKRSCRRRLAGHNERRRKS : 75
AmSPL11 : CQ-VEGCGLDLSSAKDYHRKHRVCENHSKSPKVIVGGLERRFCQQCSR---------------------------FHGLSEFDEKKRSCRRRLSDHNARRRKP : 75
AmSPL12 : CQ-VEDCRADLSNAKDYHRRHKVCDMHSKATKALVGNVMQRFCQQCSR---------------------------FHVLQEFDEGKRSCRRRLAGHNKRRRK- : 74
AmSPL13A : ---FHSLEEFDEGKRSCRKRLDGHNRRRRKP : 28
AmSPL13B : CL-VDGCDSDLSNCRDYHRRHKVCELHSKTPQVLICGQKQRFCQQCSR---------------------------FHCLEEFDEGKRSCRKRLDGHNRRRRKP : 75
AmSPL14 : CQ-VDNCKEDLSNAKDYHRRHKVCELHSKSTKALVGKQMQRFCQQCSR---------------------------FHPLSEFDEGKRSCRRRLAGHNRRRRK- : 74
MpSPL1 : CQ-VEDCGADLSNAKDYHRRHKVCEMHSKASRALVGNVMQRFCQQCSR---------------------------FHVLQEFDEGKRSCRRRLAGHNKRRRK- : 74
MpSPL3 : CQ-VESCRADMTDAKKYHRRHKVCDFHAKAPVVPVDGHPQRFCQQCSR---------------------------FHELSEFDDAKRSCRRRLAGHNERRRKS : 75
MpSPL5 : CA-VEKCGTDLTDARRYHRRHKVCETHSKAPVVIVAGLRQRFCQQCSR---------------------------FHELSEFDETKRSCRRRLAGHNERRRKS : 75
MpSPL6A : CQ-VYGCNKDLSSSKDYHKRHKVCDVHSKTPKVIVNGNEQRFCQQCSR---------------------------FHLLAEFDDSKRSCRRRLAGHNERRRKP : 75
MpSPL6B : CQ-VYGCNKDLSSSKDYHKRHKVCEAHSKAAKVIVNEVEKRFCQQCSR---------------------------FHLLDEFDDVKRSCRKRLAGHNERRRKP : 75
MpSPL7 : CQ-VPGCEADISELKGYHKRHRVCLRCANASTVLLDGDSKRYCQQCGK---------------------------FHLLSDFDEGKRSCRRKLERHNNRRRRK : 75
MpSPL8 : CQAM--CSADLTHAKHYHRRHKVCEFHSKAATVIAAGLTQRFCQQCSR---------------------------F--------------------------- : 47
MpSPL9 : CQ-VEGCEVDLSDAKAYYSRHKVCGMHSKSPVVTVAGLEQRFCQQCSR---------------------------FHQLPEFDQGKRSCRRRLAGHNERRRKP : 75
MpSPL10 : CQ-VEGCHVPLINAKDYHRRHKVCEVHSKAPKVMVLGLEQRFCQQCSR---------------------------FHVVSEFDDSKRSCRRRLAGHNERRRKS : 75
MpSPL11 : CQ-VEGCGLDLSSAKDYHRKHRVCEYHSKRPEVIVGGLERRFCQQCSR---------------------------FHGLSEFDEKKRSCRRRLSDHNARRRKS : 75
MpSPL12 : --VAEFDEGKRSCRRRLAGHNKRRRK- : 24
MpSPL13A : CE-FVGFSADLGKCRDYHRRHKVCELHSKTPKVTIHGREQRFCQQCSR---------------------------FHSLEEFDEGKRSCRKRLDGHNRRRRKP : 75
MpSPL13B : CL-VDGCDSDLSNCRDYHRRHKVCELHSKTPQVLICGQKQRFCQQCSR---------------------------FHCLEEFDEGKRSCRKRLDGHNRRRRKP : 75
MpSPL14 : CQ-VDNCKEDLSNAKDYHRRHKVCEPHSKSTKALVGKQMQRFCQQCSR---------------------------FHPLSEFDEGKRSCRRRLAGHNRRRRK- : 74
AbAPL1 : CQ-VEDCGADLSNAKDYHRRHKVCEMHSKASRALVGNVMQRFCQQCSR---------------------------FHVLQEFDEGKRSCRRRLAGHNKRRRK- : 74
AbSPL4 : CQ-VDKCAADLSDAKQYHRRHKVCEVHAKAQVVLVGGIRQRFCQQCSR---------------------------FHELSEFDETKRSCRRRLAGHNERRRKN : 75
AbSPL5 : CM-VEKCGTDLTDARRYHRRHKVCETHSKAPVVIVAGLRQRFCQQCSR---------------------------FHELYEFDETKRSCRRRLAGHNERRKS : 75
AbSPL6A : CQ-VYGCNKDLSSSKDYHKRHKVCDVHSKTPKVIVNGNEQRFCQQCSR---------------------------FHLLAEFDDSKRSCRRRLAGHNERRRKP : 75
AbSPL6B : CQ-VYGCNKDLSSSKGYHKRHKVCEDHSKAAKVTVNEVEKRFCQQCSR---------------------------FHFLDEFDDGKRSCRKRLASHNERRRKP : 75
AbSPL7 : CQ-VPGCEADISELKGYHKRHRVCLRCANASTVLLDGESKRYCQQCGK---------------------------FHLLCDFDEGKRSCRRKLERHNNRRRRK : 75
AbSPL8 : CQAEG-CSADLTHAKHYHRRHKVCEFHSKASTVIAAGLTQRFCQQCSR---------------------------FHLLSEFDNGKRSCRKRLADHNRRRRKS : 75
AbSPL9 : CQ-VEGCKVDLSDAKAYYSRHKVCGMHSKSPVVTVAGLEQRFCQQCSR---------------------------FHQLPEFDQGKRSCRRRLAGHNERRRKP : 75

```
AbSPL10   : CQ-VEGCHVPLINAKDYHRRHKVCEVHSKAPKVKVLGLEQRFCQQCSR---------------------------FHVVSEFDDSKRSCRRRLAGHNERRRKS :  75
AbSPL11   : CQ-VEGCGLDLSSAKDYHRKHRVCENHSKSPKVIVGGLERRFCQQCSR---------------------------FHGLSEFDEKKRSCRRRLSDHNARRRKS :  75
AbSPL12   : CQ-VEDCRADLSNAKDYHRRHKVCDMHSKATKALVGNVMQRFCQQCSR---------------------------FHVLQEFDEGKRSCRRRLAGHNKRRRK- :  74
AbSPL13A  : CL-VDGCTADLGKCRDYHRRHKVCELHSKTPKVTIHGREQRFCQQCSR---------------------------FHSLEEFDEGKRSCRKRLDGHNRRRRKP :  75
AbSPL13B  : CL-VDGCDSDLSNCRDYHRRHKVCELHSKTPQVLICGQKQRFCQQCSR---------------------------FHCLEEFDEGKRSCRKRLDGHNRRRRKP :  75
AbSPL14   : CQ-VDNCKEDLSNAKDYHRRHKVCELHSKSTKALVGKQMQRFCQQCSR---------------------------FHPLSEFDEGKRSCRRRLAGHNRRRRK- :  74
ClSPL1    : CQ-VEDCGADLSNAKDYHRRHKVCEMHSKASRALVGNVMQRFCQQCSR---------------------------FHVLQEFDEGKRSCRRRLAGHNKRRRK- :  74
ClSPL3    : CQ-VENCRADMTDAKKYHRRHKVCDFHAKAPVVPIAGLPQRFCQQCSR------------------------------------------------------- :  47
ClSPL4    : CQ-GDKCAADLSDAKQYHRRHKVCEVHAKAQVVLVGGIRQRFCQQCSR---------------------------FHELLEFDETKRSCRRRLAGHNERRRKN :  75
ClSPL5    : CV-VEKCGTDLTDARRYHRRHKVCETHSKAPVVIVAGLRQRFCQQCSR---------------------------YQ-------------------------- :  49
ClSPL6A   : CQ-VYGCNKDLSSSKDYHKRHKVCDVHSKTPKVIVNGNEQRFCQQCSR---------------------------FHLLAEFDDSKRSCRRRLAGHNERRRKP :  75
ClSPL6B   : CQ-VYGCNKDLSSSKDYHKRHKVCEAHSKAAKVVVDQFEQRFCQQCSR---------------------------FHFLDEFDDGKRSCRKRLAGHNERRRKP :  75
ClSPL7    : CQ-VPGCEADISELKGYHKRHRVCLRCANASTVLLDGESKRYCQQCGK---------------------------FHLLSDFDEGKRSCRRKLERHNNRRRRK :  75
ClSPL8    : CQAM--CSADLTHAKHYHRRHKVCEFHSKASTVIAAGLTQRFCQQCSR---------------------------FHLLSEFDNGKRSCRKRLADHNRRRRKS :  74
ClSPL9    : CQ-VEGCKVDLSDAKAYYSRHKVCGMHSKSPVVTVAGLEQRFCQQCSR---------------------------FHQLPEFDQGKRSCRRRLAGHNERRRKP :  75
ClSPL10   : CQ-VEGCHVPLINAKDYHRRHKVCEVHSKAPKVMVLGLEQRFCQQCSR---------------------------FHVVSEFDDSKRSCRRRLAGHNERRRKS :  75
ClSPL11   : CQ-VEGCGLDLSSAKDYHRKHRVCENHSKSPKVIVGGLERRFCQQCSR---------------------------FHGLSEFDEKKRSCRRRLSDHNARRRKS :  75
ClSPL12   : CQ-VEDCRADLSNAKDYHRRHKVCDMHSKATKALVGNITQRFCQQCSR---------------------------FHVLQEFDEGKRSCRRRLAGHNKRRRK- :  74
ClSPL13A  : CL-VDGCTADLGNCRDYHRRHKVCELHSKTPKVTIHGREQRFCQQCSR---------------------------FHSLEEFDEGKRSCRKRLDGHNRRRRKP :  75
ClSPL13B  : CL-VDGCDSDLSNCRDYHRRHKVCELHSKTPQVLICGQKQRFCQQCSR---------------------------FHCLEEFDEGKRSCRKRLDGHNRRRRKP :  75
ClSPL14   : CQ-VDNCKEDLSNAKDYHRRHKVCELHSKSTKALVGKQMQRFCQQCSR---------------------------FHPLSEFDEGKRSCRRRLAGHNRRRRK- :  74
CgSPL1    : CQ-VEDCGADLSNAKDYHRRHKVCEMHSKSSRALVGNVMQRFCQQCSR---------------------------FHVLQEFDEGKRSCRRRLAGHNKRRRK- :  74
CgSPL3    : CQ-VENCRADMTDAGKYHRRHKVCDSHAKAPVVRVEGLAQRFCQQCSK---------------------------FHELSEFDDAKRSCRRRLAGHNERRRKS :  75
CgSPL4    : CQ-VDKCAADLSDAKQYHRRHKVCEVHAKAQVVLVGGIRQRFCQQCSR---------------------------CHELSEFDETKRSCRRRLAGHNERRRKN :  75
CgSPL5    : CICVERCGTDLTDARRYHRRHKVCETHSKAPVAIVAGLRQRFCQQCSR---------------------------FHELSEFDETKRSCRTRLAGHNERRRKS :  76
CgSPL6A   : CQ-VYGCNKDLSSSKDYHKRHKVCDVHSKTPKVIVNGNEQRFCQQCSR---------------------------FHLLAEFDDSKRSCRRRLAGHNERRRKP :  75
CgSPL6B   : CQ-VYGCNKDLSSSKDYHKRHKVCEAHSKAAKVIVKEVEQRFCQQCSR---------------------------FHFLDEFDDGKRSCRKRLAGHNERRRKP :  75
CgSPL7    : CQ-VPGCEADISELKGYHKRHRVCLRCANASTVLLDGESKRYCQQCGK---------------------------FHLLSDFDEGKRSCRRKLERHNNRRRRK :  75
CgSPL8    : CQAM--CSADLTHAKHYHRRHKVCEFHSKASTVIAAGLTQRFCQQCSR---------------------------FHLLSEFDNGKRSCRKRLADHNRRRRKS :  74
CgSPL9    : CQ-VEGCKVDLSDAKAYYSRHKVCGMHSKSPVVTVAGLEQRFCQQCSR---------------------------FHQLPEFDQGKRSCRRRLAGHNERRRKP :  75
CgSPL10   : CQ-VEGCHVPLINAKDYHRRHKVCEVHSKAPKVKVLGLEQRFCQQCSR---------------------------FHVVSEFDDSKRSCRRRLAGHNERRRKS :  75
CgSPL11   : CQ-VEGCGLDLSSAKDYHRKHRVCENHSKSPKVIVGGLERRFCQQCSR---------------------------FHGLSEFDEKKRSCRRRLSDHNARRRKS :  75
CgSPL12   : CQ-VEDCRADLSNAKDYHRRHKVCDMHSKATKALVGNVMQRFCQQCSR---------------------------FHVLQEFDEGKRSCRRRLAGHNKRRRK- :  74
CgSPL13A  : CL-VDGCTADLGKCRDYHRRHKVCELHSKTPKVTIHGREQRFCQQCSR---------------------------FHSLEEFDEGKRSCRKRLDGHNRRRRKP :  75
CgSPL13B  : CL-VDGCDSDLSNCRDYHRRHKVCELHSKTPQVLICGQKQRFCQQCSR---------------------------FHCLEEFDEGKRSCRKRLDGHNRRRRKP :  75
CgSPL14   : CQ-VDNCKEDLSNAKDYHRRHKVCELHSKSTKALVGKQMQRFCQQCSR---------------------------FHPLSEFDEGKRSCRRRLAGHNRRRRK- :  74
ChSPL1    : CQ-VEDCGADLSNAKDYHRRHKVCEMHSKASRALVGNVMQRFCQQCSR---------------------------FHVLQEFDEGKRSCRRRLAGHNKRRRK- :  74
ChSPL3    : CQ-VENCRADMTDAKKYHRRHKVCDFHAKAPVVRVEGLAQRFCQQCSR---------------------------FHELSEFDDTKRSCRRRLAGHNERRRKS :  75
ChSPL4    : CQ-VDKCAADLSDAKQYHRRHKVCEVHAKAQVVLMGGIRQRFCQQCSR---------------------------FHELSEFDETKRSCRRRLAGHNERRRKN :  75
ChSPL5    : CM-VEKCGTDLTDARRYHRRHKVCETHSKAPVVIVAGLRQRFCQQCSR---------------------------FHELYEFDETKRSCRRRLAGHNERRRKS :  75
ChSPL6A   : CQ-VYGCNKDLSSSKDYHKRHKVCDVHSKTPKVIVNGNEQRFCQQCSR---------------------------FHLLAEFDDSKRSCRRRLAGHNERRRKP :  75
ChSPL6B   : CQ-VYGCNKDLSSSKDYHKRHKVCEAHSKAAKVIVNEIEQRFCQQCSR---------------------------FHFLDEFDDGKRSCRKRLAGHNERRRKP :  75
ChSPL7    : CQ-VPGCEADISELKGYHKRHRVCLRCANASTVLLDGESKRYCQQCGK---------------------------FHLLSDFDEGKRSCRRKLERHNNRRRRK :  75
```

```
ChSPL8    : CQAM--CSADLTHAKHYHRRHKVCEFHSKASTVIAAGLTQRFCQQCSR---------------------------FHLLSEFDNGKRSCRKRLADHNRRRRKS :  74
ChSPL9    : CQ-VEGCKVDLSDAKAYYSRHKVCGMHSKSPVVTVAGLEQRFCQQCSR---------------------------FHQLPEFDQGKRSCRRRLAGHNERRRKP :  75
ChSPL10   : CQ-VEGCHVPLINAKDYHRRHKVCEVHSKAPKVKVLGLEQRFCQQCSR---------------------------FHVVSEFDDSKRSCRRRLAGHNERRRKS :  75
ChSPL11   : CQ-VEGCGLDLSSAKDYHRKHRVCENHSKSPKVIVGGLERRFCQQCSR---------------------------FHGLSEFDEKKRSCRRRLSDHNARRRKS :  75
ChSPL12   : CQ-VEDCRADLSNAKDYHRRHKVCDMHSKATKALVGNVMQRFCQQCSR---------------------------FHVLQEFDEGKRSCRRRLAGHNKRRRK- :  74
ChSPL13A  : ----------------------------------------------AK---------------------------FHSLEEFDEGKRSCRKRLDGHNRRRRKP :  30
ChSPL13B  : CL-VDGCDSDLSNCRDYHRRHKVCELHSKTPQVLICGQKQRFCQQCSR---------------------------FHCLEEFDEGKRSCRKRLDGHNRRRRKP :  75
ChSPL14   : CQ-VDNCKEDLSNAKDYHRRHKVCELHSKSTKALVGKQMQRFCQQCSR---------------------------FHPLSEFDEGKRSCRRRLAGHNRRRRK- :  74
CmjSPL1   : CQ-VEDCGADLSNAKDYHRRHKVCEMHSKASRALVGNVMQRFCQQCSR---------------------------FHVLQEFDEGKRSCRRRLAGHNKRRRK- :  74
CmjSPL3   : CQ-VENCRADMTDAKKYHRRHKVCDFHAKAPVVRVEGLAQRFCQQCSR---------------------------FHELSEFDDTKRSCRRRLAGHNERRRKS :  75
CmjSPL4   : CQ-VDKCAADLSDAKQYHRRHKVCEVHAKAQVVLMGGIRQRFCQQCSR---------------------------FHELSEFDETKRSCRRRLAGHNERRRKN :  75
CmjSPL5   : CM-VEKCGTDLTDARRYHRRHKVCETHSKAPVVIVAGLRQRFCQQCSR---------------------------FHELYEFDETKRSCRRRLAGHNERRRKS :  75
CmjSPL6A  : CQ-VYGCNKDLSSSKDYHKRHKVCDVHSKTPKVIVNGNEQRFCQQCSR---------------------------FHLLAEFDDSKRSCRRRLAGHNERRRKP :  75
CmjSPL6B  : CQ-VYGCNKDLSSSKDYHKRHKVCEAHSKAAKVIVNEIEQRFCQQCSR---------------------------FHFLDEFDDGKRSCRKRLAGHNERRRKP :  75
CmjSPL7   : CQ-VPGCEADISELKGYHKRHRVCLRCANASTVLLDGESKRYCQQCGK---------------------------FHLLSDFDEGKRSCRRKLERHNNRRRRK :  75
CmjSPL8   : CQAM--CSADLTHAKHYHRRHKVCEFHSKASTVIAAGLTQRFCQQCSR---------------------------FHLLSEFDNGKRSCRKRLADHNRRRRKS :  74
CmjSPL9   : CQ-VEGCKVDLSDAKAYYSRHKVCGMHSKSPVVTVAGLEQRFCQQCSR---------------------------FHQLPEFDQGKRSCRRRLAGHNERRRKP :  75
CmjSPL10  : CQ-VEGCHVPLINAKDYHRRHKVCEVHSKAPKVKVLGLEQRFCQQCS-------------------------------------------------------- :  46
CmjSPL11  : CQ-VEGCGLDLSSAKDYHRKHRVCENHSKSPKVIVGGLERRFCQQCSR---------------------------FHGLSDFDEKKRSCRRRLSDHNARRRKS :  75
CmjSPL12  : CQ-VEDCRADLSNAKDYHRRHKVCDMHSKATKALVGNVMQRFCQQCSR---------------------------FHVLQEFDEGKRSCRRRLAGHNKRRRK- :  74
CmjSPL13A : ----------------------------------------------AK---------------------------FHSLEEFDEGKRSCRKRLDGHNRRRRKP :  30
CmjSPL13B : CL-VDGCDSDLSNCRDYHRRHKVCELHSKTPQVLICGQKQRFCQQCSR---------------------------FHCLEEFDEGKRSCRKRLDGHNRRRRKP :  75
CmjSPL14  : CQ-VDNCKEDLSNAKDYHRRHKVCELHSKSTKALVGKQMQRFCQQCSR---------------------------FHPLSEFDEGKRSCRRRLAGHNRRRRK- :  74
CzpSPL1   : CQ-VEDCGADLSNAKDYHRRHKVCEMHSKASRALVGNVMQRFCQQCSR---------------------------FHVLQEFDEGKRSCRRRLAGHNKRRRK- :  74
CzpSPL3   : CQ-VENCRADMTDAKKYHRRHKVCDFHAKAPVVRVEGLAQRFCQQCSR---------------------------FHELSEFDDTKRSCRRRLAGHNERRRKS :  75
CzpSPL5   : CM-VEKCGTDLTDARRYHRRHKVCETHSKAPVVIVAGLRQRFCQQCSR---------------------------FHELYEFDETKRSCRRRLAGHNERRRKS :  75
CzpSPL6B  : CQ-VYGCNKDLSSSKDYHKRHKVCEAHSKAAKVIVNEIEQRFCQQCSR---------------------------FHFLDEFDDGKRSCRKRLAGHNERRRKP :  75
CzpSPL7   : CQ-VPGCEADISELKGYHKRHRVCLRCANASTVLLDGESKRYCQQCGK---------------------------FHLLSDFDEGKRSCRRKLERHNNRRRRK :  75
CzpSPL8   : CQAM--CSADLTHAKHYHRRHKVCEFHSKASTVIAAGLTQRFCQQCSR---------------------------FHLLSEFDNGKRSCRKRLADHNRRRRKS :  74
CzpSPL9   : CQ-VEGCKVDLSDAKAYYSRHKVCGMHSKSPVVTVAGLEQRFCQQCSR---------------------------FHQLPEFDQGKRSCRRRLAGHNERRRKP :  75
CzpSPL10  : CQ-VEGCHVPLINAKDYHRRHKVCEVHSKAPKVKVLGLEQRFCQQCSR---------------------------FHVVSEFDDSKRSCRRRLAGHNERRRKS :  75
CzpSPL12  : CQ-VEDCRADLSNAKDYHRRHKVCDMHSKATKALVGNVMQRFCQQCSR---------------------------FHVLQEFDEGKRSCRRRLAGHNKRRRK- :  74
CzpSPL13B : CL-VDGCDSDLSNCRDYHRRHKVCELHSKTPQVLICGQKQRFCQQCSR---------------------------FHCLEEFDEGKRSCRKRLDGHNRRRRKP :  75
CzpSPL14  : CQ-VDNCKEDLSNAKDYHRRHKVCELHSKSTKALVGKQMQRFCQQCSR---------------------------FHPLSEFDEGKRSCRRRLAGHNRRRRK- :  74
CmSPL1    : CQ-VEDCGADLSNAKDYHRRHKVCEMHSKASRALVGNVMQRFCQQCSR---------------------------FHVLQEFDEGKRSCRRRLAGHNKRRRK- :  74
CmSPL3    : CQ-VENCRADMTDAKKYHRRHKVCDFHAKAPVVRVEGLAQRFCQQCSR---------------------------FHELSEFDDTKRSCRRRLAGHNERRRKS :  75
CmSPL4    : CQ-VDKCAADLSDAKQYHRRHKVCEVHAKAQVVLMGGIRQRFCQQCSR---------------------------FHELSEFDETKRSCRRRLAGHNERRRKN :  75
CmSPL5    : CM-VEKCGTDLTDARRYHRRHKVCETHSKAPVVIVAGLRQRFCQQCSR---------------------------FHELYEFDETKRSCRRRLAGHNERRRKS :  75
CmSPL6A   : CQ-VYGCNKDLSSSKDYHKRHKVCDVHSKTPKVIVNGNEQRFCQQCSR---------------------------FHLLAEFDDSKRSCRRRLAGHNERRRKP :  75
CmSPL6B   : CQ-VYGCNKDLSSSKDYHKRHKVCEAHSKAAKVIVNEIEQRFCQQCSR---------------------------FHFLDEFDDGKRSCRKRLAGHNERRRKP :  75
CmSPL7    : CQ-VPGCEVDISELKGYHKRHRVCLRCANASTVLLDGESKRYCQQCGK---------------------------FHLLSDFDEGKRSCRRKLERHNNRRRRK :  75
CmSPL8    : CQAM--CSADLTHAKHYHRRHKVCEFHSKASTVIAAGLTQRFCQQCSR---------------------------FHLLSEFDNGKRSCRKRLADHNRRRRKS :  74
CmSPL9    : CQ-VEGCKVDLSDAKAYYSRHKVCGMHSKSPVVTVAGLEQRFCQQCSR---------------------------FHQLPEFDQGKRSCRRRLAGHNERRRKP :  75
```

```
CmSPL10   : CQ-VEGCHVPLINAKDYHRRHKVCEVHSKAPKVKVLGLEQRFCQQCSR---------------------------FHVVSEFDDSKRSCRRRLAGHNERRRKS :  75
CmSPL11   : CQ-VEGCGLDLSSAKDYHRKHRVCENHSKSPKVIVGGLERRFCQQCSR---------------------------FHGLSEFDEKKRSCRRRLFDHNARRRKS :  75
CmSPL12   : CQ-VEDCRADLSNAKDYHRRHKVCDMHSKATKALVGNVMQRFCQQCSR---------------------------FHVLQEFDEGKRSCRRRLAGHNKRRRK- :  74
CmSPL13B  : CL-VDGCDSDLSNCRDYHRRHKVCELHSKTPQVLICGQKQRFCQQCSR---------------------------FHCLEEFDEGKRSCRKRLDGHNRRRRKP :  75
CmSPL14   : CQ-VDNCKEDLSNAKDYHRRHKVCELHSKSTKALVGKQMQRFCQQCSR---------------------------FHPLSEFDEGKRSCRRRLAGHNRRRRK- :  74
CicSPL1   : CQ-VEDCGADLSNAKDYHRRHKVCEMHSKASRALVGNVMQRFCQQCSR---------------------------FHVLQEFDEGKRSCRRRLAGHNKRRRK- :  74
CicSPL3   : CQ-VENCRADMTDAKKYHRRHKVCDFHAKAPVVRVEGLAQRFCQQCSR---------------------------FHELSEFDDAKRSCRRRLAGHNERRRKS :  75
CicSPL4   : CQ-VDKCAADLSDAKQYHRRHKVCEVHAKAQVVLMGGIRQRFCQQCSR---------------------------FHELSEFDETKRSCRRRLAGHNERRRKN :  75
CicSPL5   : CM-VEKCGTDLTDARRYHRRHKVCETHSKAPVVIVAGLRQRFCQQCSR---------------------------FHELYEFDETKRSCRRRLAGHNERRRKS :  75
CicSPL6A  : CQ-VYGCNKDLSSSKDYHKRHKVCDVHSKTPKVIVNGNEQRFCQQCSR---------------------------FHLLAEFDDSKRSCRRRLAGHNERRRKP :  75
CicSPL6B  : CQ-VYGCNKDLSSSKDYHKRHKVCEAHSKAAKVIVNEIEQRFCQQCSR---------------------------FHFLDEFDDGKRSCRKRLAGHNERRRKP :  75
CicSPL7   : CQ-VPGCEADISELKGYHKRHRVCLRCANASTVLLDGESKRYCQQCGK---------------------------FHLLSDFDEGKRSCRRKLERHNNRRRRK :  75
CicSPL8   : CQAM--CSADLTHAKHYHRRHKVCEFHSKASTVIAAGLTQRFCQQCSR---------------------------FHLLSEFDNGKRSCRKRLADHNRRRRKS :  74
CicSPL9   : CQ-VEGCKVDLSDAKAYYSRHKVCGMHSKSPVVTVAGLEQRFCQQCSR---------------------------FHQLPEFDQGKRSCRRRLAGHNERRRKP :  75
CicSPL10  : CQ-VEGCHVPLINAKDYHRRHKVCEVHSKAPKVKVLGLEQRFCQQCSR---------------------------FHVVSEFDDSKRSCRRRLAGHNERRRKS :  75
CicSPL11  : CQ-VEGCGLDLSSAKDYHRKHRVCENHSKSPKVIVGGLERRFCQQCSR---------------------------FHGLSEFDEKKRSCRRRLSDHNARRRKS :  75
CicSPL12  : CQ-VEDCRADLSNAKDYHRRHKVCDMHSKATKALVGNVMQRFCQQCSR---------------------------FHVLQEFDEGKRSCRRRLAGHNKRRRK- :  74
CicSPL13A : ---------------------------------------------------------------------------FHSLEEFDEGKRSCRKRLDGHNRRRRKP :  28
CicSPL13B : CL-VDGCDSDLSNCRDYHRRHKVCELHSKTPQVLICGQKQRFCQQCSR---------------------------FHCLEEFDEGKRSCRKRLDGHNRRRRKP :  75
CicSPL14  : CQ-VDNCKEDLSNAKDYHRRHKVCELHSKSIKALVGKQMQRFCQQCSR---------------------------FHPLSEFDEGKRSCRRRLAGHNRRRRK- :  74
CsSPL1    : CQ-VEDCGADLSNAKDYHRRHKVCEMHSKASRALVGNVMQRFCQQCSR---------------------------FHVLQEFDEGKRSCRRRLAGHNKRRRK- :  74
CsSPL3    : CQ-VENCRADMTDAKKYHRRHKVCDFHAKAPVVRVEGLAQRFCQQCSR---------------------------FHELSEFDDTKRSCRRRLAGHNERRRKS :  75
CsSPL4    : CQ-VDKCAADLSDAKQYHRRHKVCEVHAKAQVVLMGGMRQRFCQQCSR---------------------------FHELSEFDETKRSCRRRLAGHNERRRKN :  75
CsSPL5    : CM-VEKCGTDLTDARRYHRRHKVCETHSKAPVVIVAGLRQRFCQQCSR---------------------------FHELYEFDETKRSCRRRLAGHNERRRKS :  75
CsSPL6A   : CQ-VYGCNKDLSSSKDYHKRHKVCDVHSKTPKVIVNGNEQRFCQQCSR---------------------------FHLLAEFDDSKRSCRRRLAGHNERRRKP :  75
CsSPL6B   : CQ-VYGCNKDLSSSKDYHKRHKVCEAHSKAAKVIVNEIEQRFCQQCSR---------------------------FHFLDEFDDGKRSCRKRLAGHNERRRKP :  75
CsSPL7    : CQ-VPGCEADISELKGYHKRHRVCLRCANASTVLLDGESKRYCQQCGK---------------------------FHLLSDFDEGKRSCRRKLERHNNRRRRK :  75
CsSPL8    : CQAM--CSADLTHAKHYHRRHKVCEFHSKASTVIAAGLTQRFCQQCSR---------------------------FHLLSEFDNGKRSCRKRLADHNRRRRKS :  74
CsSPL9    : CQ-VEGCKVDLSDAKAYYSRHKVCGMHSKSPVVTVAGLEQRFCQQCSR---------------------------FHQLPEFDQGKRSCRRRLAGHNERRRKP :  75
CsSPL10   : CQ-VEGCHVPLINAKDYHRRHKVCEVHSKAPKVKVLGLEQRFCQQCSR---------------------------FHVVSEFDDSKRSCRRRLAGHNERRRKS :  75
CsSPL11   : CQ-VEGCGLDLSSAKDYHRKHRVCENHSKSPKVIVGGLERRFCQQCSR---------------------------FHGLSEFDEKKRSCRRRLSDHNARRRKS :  75
CsSPL12   : CQ-VEDCRADLSNAKDYHRRHKVCDMHSKATKALVGNVMQRFCQQCSR---------------------------FHVLQEFDEGKRSCRRRLAGHNKRRRK- :  74
CsSPL13A  : CL-VDGCTADLGKCRDYHRRHKVCELHSKTPKVTIHGREQRFCQQCSR---------------------------FHSLEEFDEGKRSCRKRLDGHNRRRRKP :  75
CsSPL13B  : CL-VDGCDSDLSNCRDYHRRHKVCELHSKTPQVLICGQKQRFCQQCSR---------------------------FHCLEEFDEGKRSCRKRLDGHNRRRRKP :  75
CsSPL14   : CQ-VDNCKEDLSNAKDYHRRHKVCELHSKSTKALVGKQMQRFCQQCSR---------------------------FHPLSEFDEGKRSCRRRLAGHNRRRRK- :  74
CrSPL1    : CQ-VEDCGADLSNAKDYHRRHKVCEMHSKASRALVGNVMQRFCQQCSR---------------------------FHVLQEFDEGKRSCRRRLAGHNKRRRK- :  74
CrSPL3    : CQ-VENCRADMTDAKKYHRRHKVCDFHAKAPVVRVEGLAQRFCQQCSR---------------------------FHELSEFDDTKRSCRRRLAGHNERRRKS :  75
CrSPL4    : CQ-VDKCAADLSDAKQYHRRHKVCEVHAKAQVVLMGGMRQRFCQQCSR---------------------------FHELSEFDETKRSCRRRLAGHNERRRKN :  75
CrSPL5    : CM-VEKCGTDLTDARRYHRRHKVCETHSKAPVVIVAGLRQRFCQQCSR---------------------------FHELYEFDETKRSCRRRLAGHNERRRKS :  75
CrSPL6A   : CQ-VYGCNKDLSSSKDYHKRHKVCDVHSKTPKVIVNGNEQRFCQQCSR---------------------------FHLLAEFDDSKRSCRRRLAGHNERRRKP :  75
CrSPL6B   : CQ-VYGCNKDLSSSKDYHKRHKVCEAHSKAAKVIVNEIEQRFCQQCSR---------------------------FHFLDEFDDGKRSCRKRLAGHNERRRKP :  75
CrSPL7    : CQ-VPGCEADISELKGYHKRHRVCLRCANASTVLLDGESKRYCQQCGK---------------------------FHLLSDFDEGKRSCRRKLERHNNRRRRK :  75
CrSPL8    : CQAM--CSADLTHAKHYHRRHKVCEFHSKASTVIAAGLTQRFCQQCSR---------------------------FHLLSEFDNGKRSCRKRLADHNRRRRKS :  74
```

```
CrSPL9    : CQ-VEGCKVDLSDAKAYYSRHKVCGMHSKSPVVTVAGLEQRFCQQCSR---------------------------FHQLPEFDQGKRSCRRRLAGHNERRRKP :  75
CrSPL10   : CQ-VEGCHVPLINAKDYHRRHKVCEVHSKAPKVKVLGLEQRFCQQCSR---------------------------FHVVSEFDDSKRSCRRRLAGHNERRRKS :  75
CrSPL11   : CQ-VEGCGLDLSSAKDYHRKHRVCENHSKSPKVIVGGLERRFCQQCSR---------------------------FHGLSEFDEKKRSCRRRLSDHNARRRKS :  75
CrSPL12   : CQ-VEDCRADLSNAKDYHRRHKVCDMHSKATKALVGNVMQRFCQQCSR---------------------------FHVLQEFDEGKRSCRRRLAGHNKRRRK- :  74
CrSPL13A  : CL-VDGCTADLGKCRDYHRRHKVCELHSKTPKVTIHGREQRFCQQCSR---------------------------FHSLEEFDEGKRSCRKRLDGHNRRRRKP :  75
CrSPL13B  : CL-VDGCDSDLSNCRDYHRRHKVCELHSKTPQVLICGQKQRFCQQCSR---------------------------FHCLEEFDEGKRSCRKRLDGHNRRRRKP :  75
CrSPL14   : CQ-VDNCKEDLSNAKDYHRRHKVCELHSKSTKALVGKQMQRFCQQCSR---------------------------FHPLSEFDEGKRSCRRRLAGHNRRRRK- :  74
CmsSPL1   : CQ-VEDCGADLSNAKDYHRRHKVCEMHSKASRALVGNVMQRFCQQCSR---------------------------FHVLQEFDEGKRSCRRRLAGHNKRRRK- :  74
CmsSPL3   : CQ-VENCRADMTDAKKYHRRHKVCDFHAKAPVVRVEGLAQRFCQQCSR---------------------------FHELSEFDDAKRSCRRRLAGHNERRRKS :  75
CmsSPL4   : CQ-VDKCAADLSDAKQYHRRHKVCEVHAKAQVVLMGGIRQRFCQQCSR---------------------------FHELSEFDETKRSCRRRLAGHNERRRKN :  75
CmsSPL5   : CM-VEQCGTDLTDARRYHRRHKVCETHSKAPVVIVAGLRQRFCQQCSR---------------------------FHELYEFDETKRSCRRRLAGHNERRRKS :  75
CmsSPL6A  : CQ-VYGCNKDLSSSKDYHKRHKVCDVHSKTPKVIVNGNEQRFCQQCSR---------------------------FHLLAEFDDSKRSCRRRLAGHNERRRKP :  75
CmsSPL6B  : CQ-VYGCNKDLSSSKDYHKRHKVCEAHSKAAKVIVNEIEQRFCQQCSR---------------------------FHFLDEFDDGKRSCRKRLAGHNERRRKP :  75
CmsSPL7   : CQ-VPGCEADISELKGYHKRHRVCLRCANASTVLLDGESKRYCQQCGK---------------------------FHLLSDFDEGKRSCRRKLERHNNRRRRK :  75
CmsSPL8   : CQAM--CSADLTHAKHYHRRHKVCEFHSKASTVIAAGLTQRFCQQCSR---------------------------FHLLSEFDNGKRSCRKRLADHNRRRRKS :  74
CmsSPL9   : CQ-VEGCKVDLSDAKAYYSRHKVCGMHSKSPVVTVAGLEQRFCQQCSR---------------------------FHQLPEFDQGKRSCRRRLAGHNERRRKP :  75
CmsSPL10  : CQ-VEECHVPLINAKDYHRRHKVCEVHSKAPKVKVLGLEQRFCQQCSR---------------------------FHVVSEFDDSKRSCRRRLAGHNERRRKS :  75
CmsSPL11  : CQ-VEGCGLDLSSAKDYHRKHRVCEYHSKSPKVIVGGLERRFCQQCSR---------------------------FHGLSEFDEKKRSCRRRLSDHNARRRKS :  75
CmsSPL12  : CQ-VEDCRADLSNAKDYHRRHKVCDMHSKATKALVGNVMQRFCQQCSR---------------------------FHVLQEFDEGKRSCRRRLAGHNKRRRK- :  74
CmsSPL13A : ---------DLGKCRDYHRRHKVCELHSKTPKVTIHGREQRFCQQCSR---------------------------FHSLEEFDEGKRSCRKRLDGHNRRRRKP :  67
CmsSPL13B : CL-VDGCDSDLSNCRDYHRRHKVCELHSKTPQVLICGQKQRFCQQCSR---------------------------FHCLEEFDEGKRSCRKRLDGHNRRRRKP :  75
CmsSPL14  : CQ-VDNCKEDLSNAKDYHRRHKVCELHSKSTKALVGKQMQRFCQQCSR---------------------------FHPLSEFDEGKRSCRRRLAGHNRRRRK- :  74
FhSPL1    : CQ-VEDCGADLSNAKDYHRRHKVCEMHSKASRALVGNVMQRFCQQCSR---------------------------FHVLQEFDEGKRSCRRRLAGHNKRRRK- :  74
FhSPL3    : CQ-VENCRADMTDAKKYHRRHKVCDFHAKAPVVRVEGLAQRFCQQCSR---------------------------FHELSEFDDAKRSCRRRLAGHNERRRKS :  75
FhSPL4    : CQ-VDKCAADLSDAKQYHRRHKVCEVHAKAQVVLMGGMRQRFCQQCSR---------------------------FHELSEFDETKRSCRRRLAGHNERRRKN :  75
FhSPL5    : CM-VEKCGTDLTDARRYHRRHKVCETHSKAPVVIVAGLRQRFCQQCSR---------------------------FHELYEFDETKRSCRRRLAGHNERRRKS :  75
FhSPL6A   : CQ-VYGCNKDLSSSKDYHKRHKVCDVHSKTPKVIVNGNEQRFCQQCSR---------------------------FHLLAEFDDSKRSCRRRLAGHNERRRKP :  75
FhSPL6B   : CQ-VYGCNKDLSSSKDYHKRHKVCEAHSKAAKVVVNEIEQRFCQQCSR---------------------------FHFLDEFDDGKRSCRKRLAGHNERRRKP :  75
FhSPL7    : CQ-VPGCEADISELKGYHKRHRVCLRCANASTVLLDGESKRYCQQCGK---------------------------FHLLSDFDEGKRSCRRKLERHNNRRRRK :  75
FhSPL8    : CQAM--CSADLTHAKHYHRRHKVCEFHSKASTVIAAGLTQRFCQQCSR---------------------------FHLLSEFDNGKRSCRKRLADHNRRRRKS :  74
FhSPL9    : CQ-VEGCKVDLSDAKAYYSRHKVCGMHSKSPVVTVAGLEQRFCQQCSR---------------------------FHQLPEFDQGKRSCRRRLAGHNERRRKP :  75
FhSPL10   : CQ-VEGCHVPLINAKDYHRRHKVCEVHSKAPKVKVLGLEQRFCQQCSR---------------------------FHVVSEFDDSKRSCRRRLAGHNERRRKS :  75
FhSPL11   : CQ-VEGCGLDLSSAKDYHRKHRVCENHSKSPKVIVGGLERRFCQQCSR---------------------------FHGLSEFDEKKRSCRRRLSDHNARRRKS :  75
FhSPL12   : CQ-VEDCRADLSNAKDYHRRHKVCDMHSKATKALVGNVMQRFCQQCSR---------------------------FHALQEFDEGKRSCRRRLAGHNKRRRK- :  74
FhSPL13A  : CL-VDGCTADLGKCRDYHRRHKVCELHSKTPKVTIHGREQRFCQQCSR---------------------------FHSLEEFDEGKRSCRKRLDGHNRRRRKP :  75
FhSPL13B  : CL-VDGCDSDLSNCRDYHRRHKVCELHSKTPQVLICGQKQRFCQQCSR---------------------------FHCLEEFDEGKRSCRKRLDGHNRRRRKP :  75
FhSPL14   : CQ-VDNCKEDLSNAKDYHRRHKVCELHSKSTKALVGKQMQRFCQQCSR---------------------------FHPLSEFDEGKRSCRRRLAGHNRRRRK- :  74
PtSPL1    : CQ-VEDCGADLSNAKDYHRRHKVCEMHSKASRALVGNVMQRFCQQCSR---------------------------FHVLQEFDEGKRSCRRRLAGHNKRRRK- :  74
PtSPL3    : CQ-VENCRADMTDAKKYHRRHKVCDFHAKAPVVRVEGLAQRFCQQCSR---------------------------FHELSEFDDTKRSCRRRLAGHNERRRKS :  75
PtSPL4    : CQ-VDKCAADLSDAKQYHRRHKVCEVHAKAQVVLMGGIRQRFCQQCSR---------------------------FHELSEFDETKRSCRRRLAGHNERRRKN :  75
PtSPL5    : CM-VEKCGTDLTDARRYHRRHKVCETHSKAPVVIVAGLRQRFCQQCSR---------------------------FHELYEFDETKRSCRRRLAGHNERRRKS :  75
PtSPL6A   : CQ-VYGCNKDLSSSKDYHKRHKVCDVHSKTPKVIVNGNEQRFCQQCSR---------------------------FHLLAEFDDSKRSCRRRLAGHNERRRKP :  75
PtSPL6B   : CQ-VYGCNKDLSSSKDYHKRHRVCEAHSKEAKVIVNEIEQRFCQQCSR---------------------------FHFLDEFDDGKRSCRKRLAGHNERRRKP :  75
```

```
PtSPL7    : CQ-VPGCEADISELKGYHKRHRVCLRCANASTVLLDGESKRYCQQCGK---------------------------FHLLSDFDEGKRSCRRKLERHNNRRRRK :  75
PtSPL8    : CQAM--CSADLTHAKHYHRRHKVCEFHSKAATVIAAGLTQRFCQQCSR---------------------------FHLLSEFDNGKRSCRKRLADHNRRRRKS :  74
PtSPL9    : CQ-VEGCKVDLSDAKAYYSRHKVCGMHSKSPVVTVAGLEQRFCQQCSR---------------------------FHQLPEFDQGKRSCRRRLAGHNERRRKP :  75
PtSPL10   : CQ-VEGCHVPLFNAKDYHRRHKVCEVHSKAPKVKVLGLEQRFCQQCSR---------------------------FHVVSEFDDSKRSCRRRLAGHNERRRKS :  75
PtSPL11   : CQ-VGGCGLDLSSAKDYHRKHRVCENHSKSPKVIVGGLERRFCQQCSR---------------------------FHGLSEFDEQKRSCRRRLSDHNARRRKS :  75
PtSPL12   : CQ-VEDCRADLSNAKDYHRRHKVCDMHSKATKALVGNVMQRFCQQCSR---------------------------FHVLQEFDEGKRSCRRRLAGHNKRRRK- :  74
PtSPL13A  : CL-VDGCTADLGKCRDYHRRHKVCELHSKTPKVTIHGREQRFCQQCSR---------------------------FHSLEEFDEGKRSCRKRLDGHNRRRRKP :  75
PtSPL13B  : CL-VDGCDSDLSNCRDYHRRHKVCELHSKTPQVLICGQKQRFCQQCSR---------------------------FHCLEEFDEGKRSCRKRLDGHNRRRRKP :  75
PtSPL14   : CQ-VDNCKEDLSNAKDYHRRHKVCELHSKSTKALVGKQMQRFCQQCSR---------------------------FHPLSEFDEGKRSCRRRLAGHNRRRRK- :  74
```
