# Supplementary material for: Evolutionary assessment of SQUAMOSA PROMOTER BINDING PROTEIN-LIKE genes in citrus relatives with a specific focus on flowering
Source: Mol Hortic. 2023 Jul 20;3:13. doi: 10.1186/s43897-023-00061-4 (PMC10515035; doi:10.1186/s43897-023-00061-4)
Supplement: Supplementary file 1 — Additional file 1: The online version contains supplementary material available at (web address will be provided by the publisher). Supplementary Fig. S1. Prediction of target sites for miR156 in SPL. Supplementary Fig. S2. SPL conserved domain sequence alignment. Supplementary Fig. S3. Gene structure. Supplementary Fig. S4. SPL-motifs prediction. Supplementary Fig. S5. Nuclear localization prediction. Supplementary Fig. S6. Cis-acting element pred by PlantCARE + TBTOOLS. Supplementary Fig. S7. FhSPL9 and FhSPL11 Mutation Sites. Supplementary Table S1. The characteristics of identified SPL genes in Citrus. Supplementary Table S2. Table S2. Prediction of miR156 and SPL target sequences. Supplementary Table S3. Prediction of miR156 target genes in Fortunella hindsii. Supplementary Table S4. Vector construction and quantitative primers for gene expression detection. Supplementary Table S5. Quantitative PCR primers for SPL genes of Fortunella hindsii, Citrus sinensis, Citrus reticulata ‘Pokan’ and Citrus maxima‘Majia’. [file 43897_2023_61_MOESM1_ESM.zip › Figure S5 Nuclear localization prediction.pdf]

Please cite this paper if NLStradamus was useful for your studies:
Nguyen Ba AN, Pogoutse A, Provart N, Moses AM. **NLStradamus: a simple Hidden Markov Model for nuclear localization signal prediction.** *BMC Bioinformatics.* 2009 Jun 29;**10(1)**:202.

The pubmed entry for this paper is found **here**.

- Program may resolve in an error if a large .fasta file is sent for prediction when images or graphics are desired. **Please only use the table display when using large predictions. (>100 sequences)**
- A standalone of the program is available **here**.
- Notice: NLStradamus is not a predictor of nuclear proteins; although better than random, it is a very poor classifier of nuclear proteins. Comparative approaches should be used for this problem. NLStradamus predicts NLSs in nuclear proteins that are transported by the import machinery of the cell.
- Click **here** for general help.

Prediction Request

Sequence Text

```
[illegible]
PLWQNHSQDPPPDRYLQCSTAGTGFSGPGIPCGGCFTGVADSNCALSLLSNQPWGSKNPTPGHGVGDLMHAHTKSVT
QPVSPHGAAINQYPNMSWGFKGNAAGSSSHQMAPQLGLAPTSVPINSQFSGELESSQQSRQYMKLQHSRDYDDSNQQ
IHWSL*
>CmSPL9
MELGSDYLAESGGGSGSGSRSGSSSAEPSLNGLKFGKKIYFEDVGTAGAPFPGSGSSSGSGSGSGRKVRGGGGGMVT
SGQQPPRCQVEGCKVDLSDAKAYYSRHKVCGMHSKSPVVTVAGLEQRFCQQCSRFHQLPEFDQGKRSCRRRLAGHNE
RRRKPTSGPFLGTRYGRLSSSVIENSSQGGGFLIDFSAYQMVGGRDGWPVTSVSKQVSGNQTTVTARHLPQPLWQNH
SQDPPPDRYLQCSTAGTGFSGPGIPCGGCFTGVADSNCALSLLSNQPWGSKNQTPGHGVGDLMHAHTKSVTQPVSPR
GAAINQYPNMPWGFKGNEAGSSSHQLAPQLGLAPTSVPINSQFSGELESSQQSRQYMKLQHSRDYDDSNQQIHWSL*
>AmSPL10
MEIGGNIVIREDTGNSNNNNSNNLAWENMWALNNHSRFDLGNSSSLYATAEVTNTTHASEGNAAHALMFPHSQSLYA
GDGSNLHPDPHLMCLKLGKRHYFEDATATSIGDQGFSMVKKGKPYYAFASAGGGGGGVGPSSSSPVLVTSTATVPR
CQVEGCHVPLINAKDYHRRHKVCEVHSKAPKVKVLGLEQRFCQQCSRFHVVSEFDDSKRSCRRRLAGHNERRRKSSH
DSLSRNSSQVNELMTGRFPFPLPRGRALSLLSSKAESWVSSSDLSSRCSAALRELIAENRAAIVTRHLILDGRDWHL
[illegible]
```

Accepts :
- 1 protein sequence
- Multiple sequences in FASTA format.

File Upload: Choose File AtSPL and all…inital).fasta

Accepts .fasta, .txt, and soon .gz, .bz2 and .zip.

HMM state emission and transition frequencies.

Pre-loaded models: 2 state HMM static | 2 state HMM dynamic | 4 state HMM static

Those are the models based on our characterized sequences.

Prediction Type

Viterbi ☑

Posterior ☑ — 0.6 Prediction Cutoff (]0,1])

Prediction Display

Table ☑ — A table that you can export with the start and stop positions of predicted NLS.

Image ☑ — An image with the NLS boxed over a line representing the protein sequence.

Graphic ☑ — Only applicable to posterior probability. A graphical representation of its distribution.

Predict

Scroll down for predictions.

Predictions for AtSPL7

Viterbi Path

Viterbi Path

Posterior @ 0.6: 199 - RRKLERHNNRRKRKPV - 214

Posterior @ 0.6

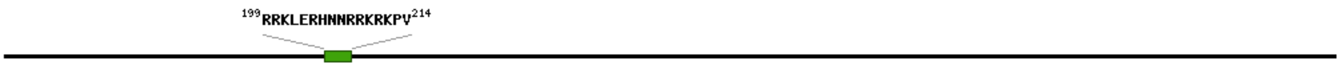

Posterior

Graphic. Threshold Marked @ 0.6

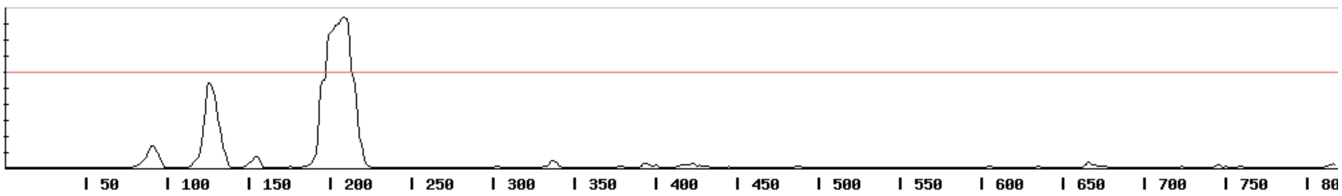

Predictions for AmSPL7

Viterbi Path

Viterbi Path

Posterior @ 0.6

197 - RRKLERHNNRRRRK - 210

Posterior @ 0.6

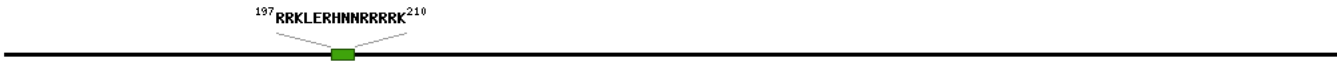

Posterior Graphic. Threshold Marked @ 0.6

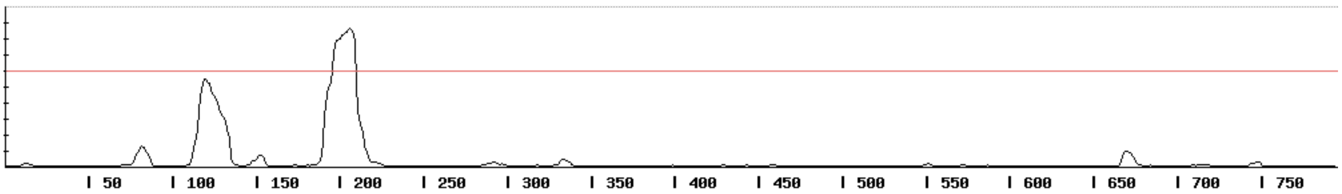

Predictions for MuSPL7

Viterbi Path

Viterbi Path

Posterior @ 0.6

192 - RRKLERHNNRRRRKS - 206

Posterior @ 0.6

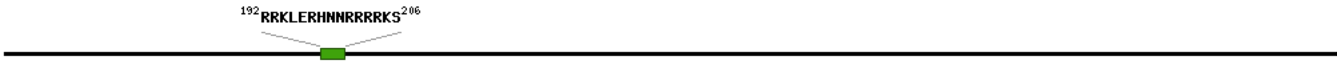

Posterior Graphic. Threshold Marked @ 0.6

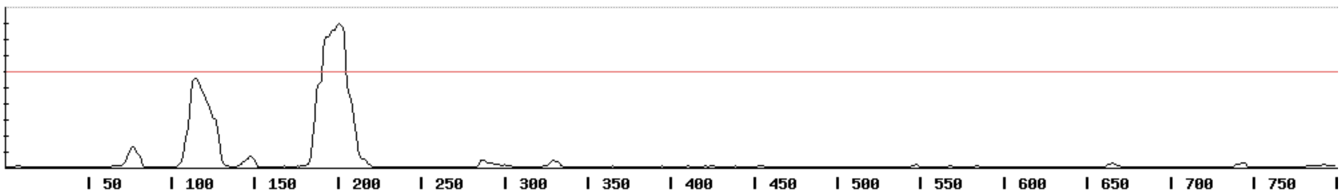

Predictions for AbSPL7

Viterbi Path

Viterbi Path

Posterior @ 0.6	192 - RRKLERHNNRRRRK - 205
Posterior @ 0.6	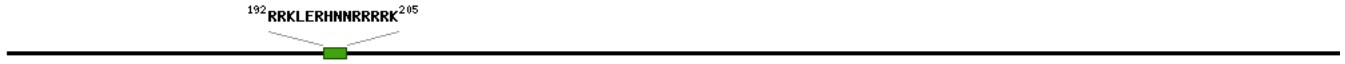
Posterior Graphic. Threshold Marked @ 0.6	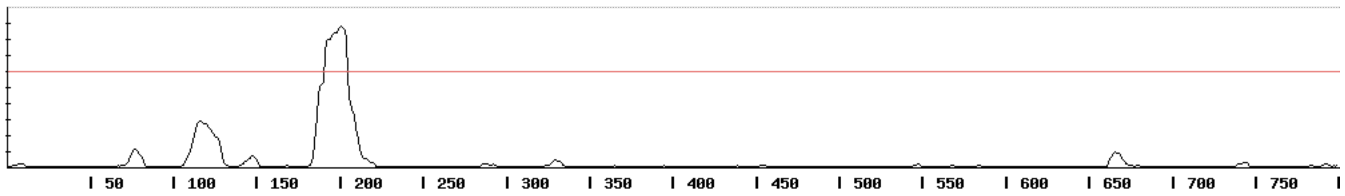

Predictions for ClSPL7

Viterbi Path	
Viterbi Path	
Posterior @ 0.6	192 - RRKLERHNNRRRRK - 205
Posterior @ 0.6	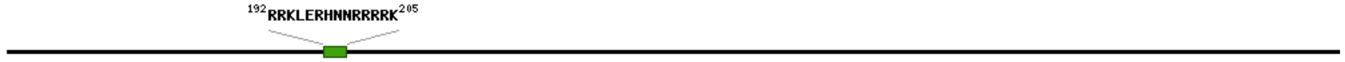
Posterior Graphic. Threshold Marked @ 0.6	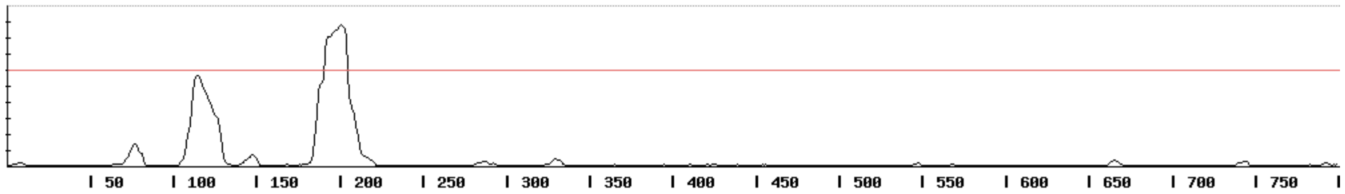

Predictions for CitSPL7

Viterbi Path	
Viterbi Path	
Posterior @ 0.6	192 - RRKLERHNNRRRRK - 205
Posterior @ 0.6	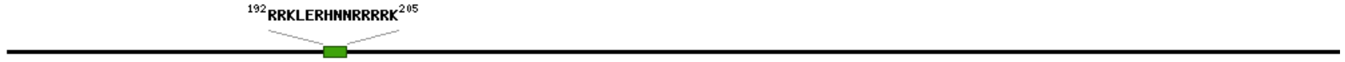
Posterior Graphic. Threshold Marked @ 0.6	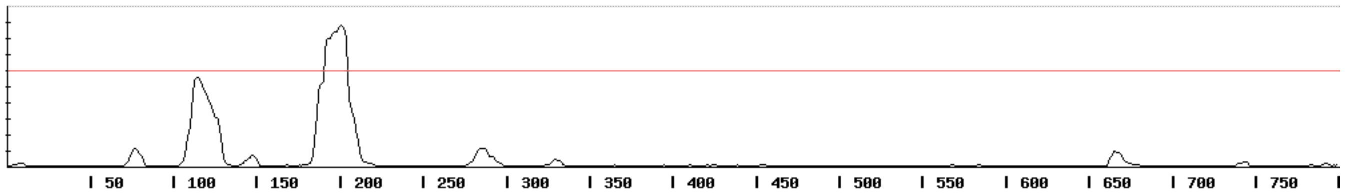

Predictions for PtSPL7

Viterbi Path	
Viterbi Path	
Posterior @ 0.6	192 - RRKLERHNNRRRRK - 205
Posterior @ 0.6	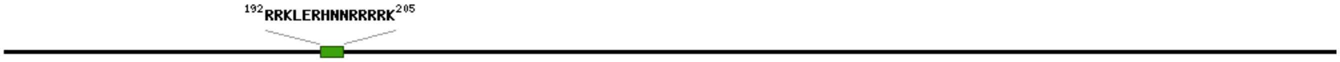
Posterior Graphic. Threshold Marked @ 0.6	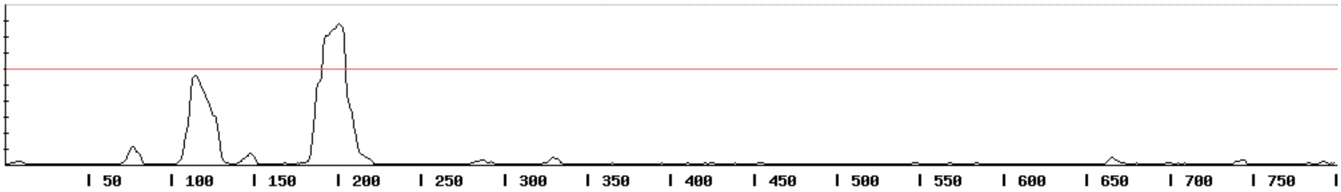

Predictions for FhSPL7

Viterbi Path	
Viterbi Path	
Posterior @ 0.6	192 - RRKLERHNNRRRRKS - 206
Posterior @ 0.6	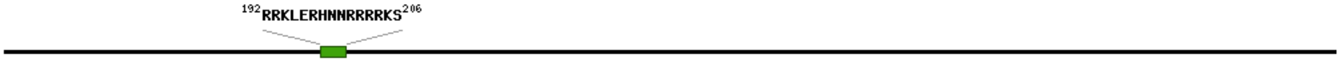
Posterior Graphic. Threshold Marked @ 0.6	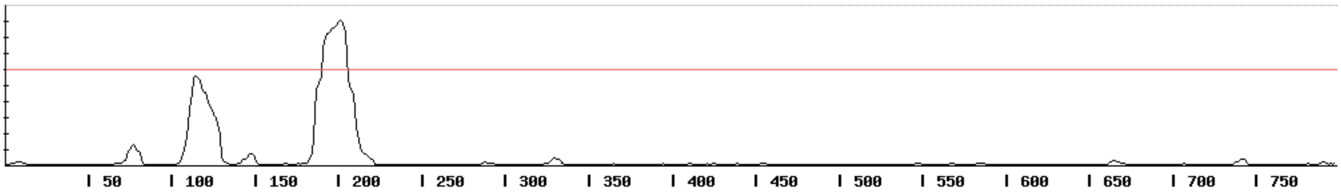

Predictions for CmsSPL7

Viterbi Path	
Viterbi Path	
Posterior @ 0.6	192 - RRKLERHNNRRRRK - 205
Posterior @ 0.6	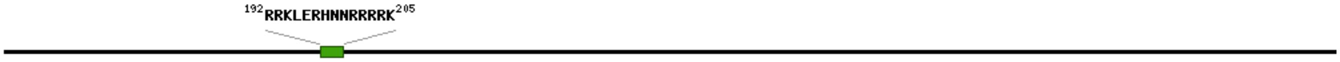
Posterior Graphic. Threshold	

Marked @ 0.6

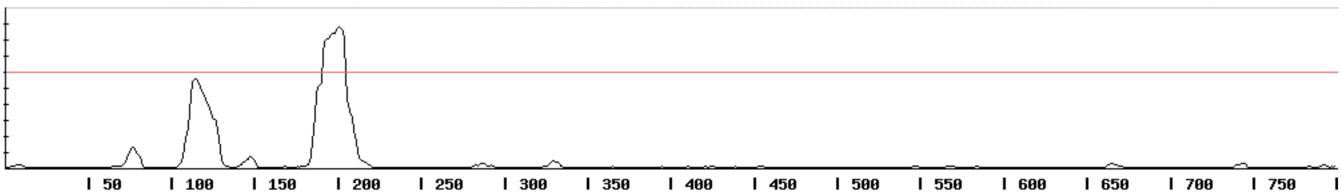

Predictions for CicSPL7

Viterbi Path

Viterbi Path

Posterior @ 0.6

192 - RRKLERHNNRRRRKS - 206

Posterior @ 0.6

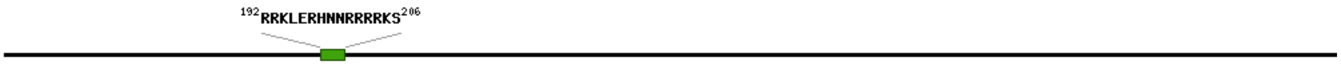

Posterior Graphic. Threshold Marked @ 0.6

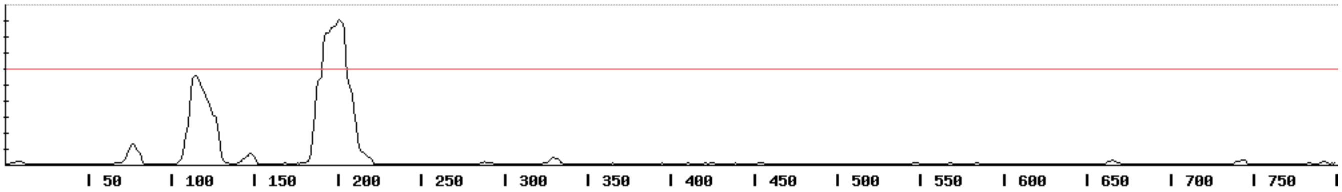

Predictions for CsSPL7

Viterbi Path

Viterbi Path

Posterior @ 0.6

192 - RRKLERHNNRRRRK - 205

Posterior @ 0.6

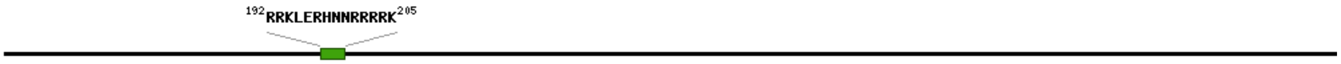

Posterior Graphic. Threshold Marked @ 0.6

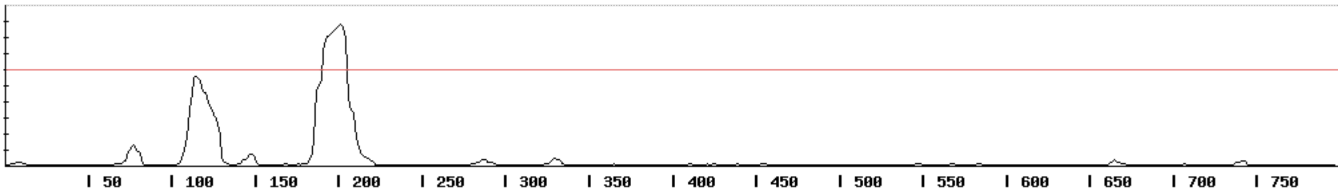

Predictions for CrSPL7

Viterbi Path

Viterbi Path

Posterior @ 0.6 | 192 - RRKLERHNNRRRRK - 205

Posterior @ 0.6

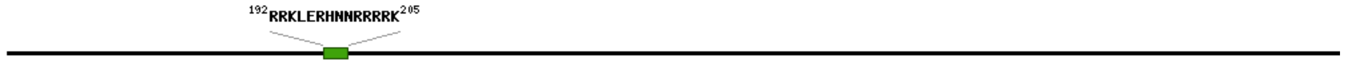

Posterior Graphic. Threshold Marked @ 0.6

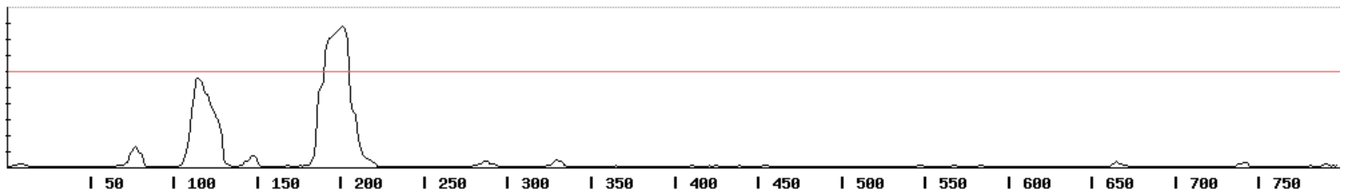

Predictions for ChSPL7

Viterbi Path

Viterbi Path

Posterior @ 0.6 | 192 - RRKLERHNNRRRRK - 205

Posterior @ 0.6

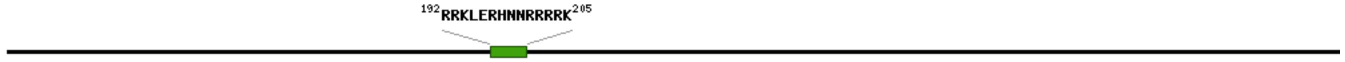

Posterior Graphic. Threshold Marked @ 0.6

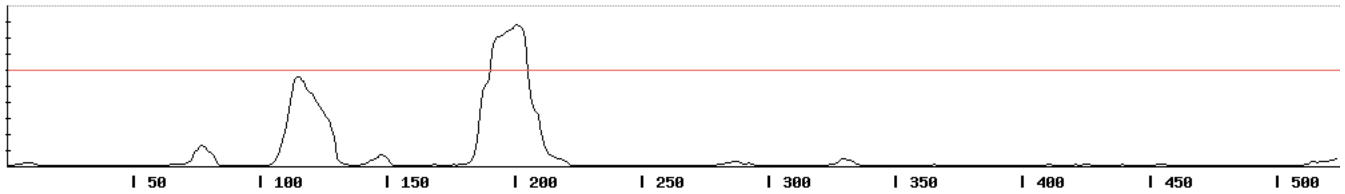

Predictions for CmjSPL7

Viterbi Path

Viterbi Path

Posterior @ 0.6 | 192 - RRKLERHNNRRRRK - 205

Posterior @ 0.6

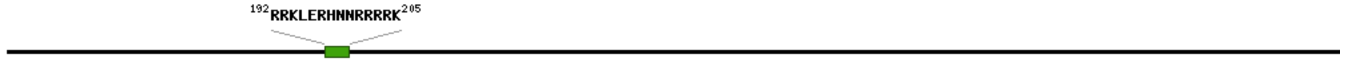

Posterior Graphic. Threshold Marked @ 0.6

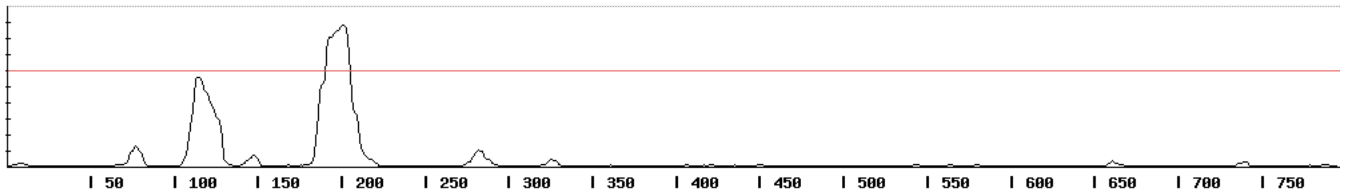

Predictions for CzpSPL7

Viterbi Path

Viterbi Path

Posterior @ 0.6

192 - RRKLERHNNRRRRK - 205

Posterior @ 0.6

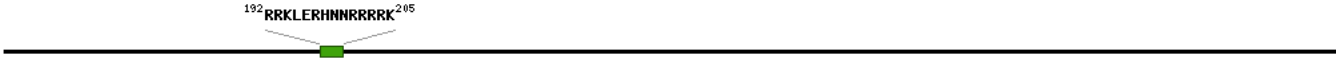

Posterior Graphic. Threshold Marked @ 0.6

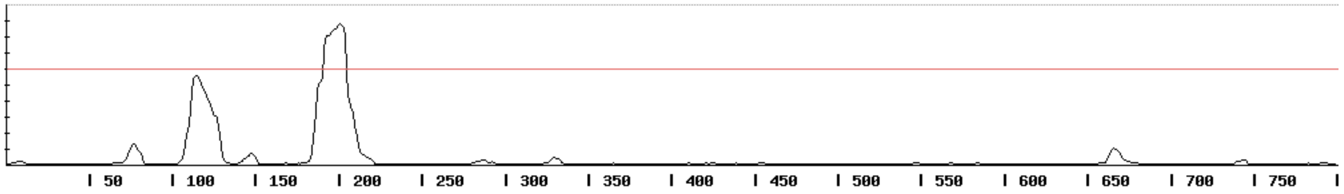

Predictions for CmSPL7

Viterbi Path

Viterbi Path

Posterior @ 0.6

192 - RRKLERHNNRRRRK - 205

Posterior @ 0.6

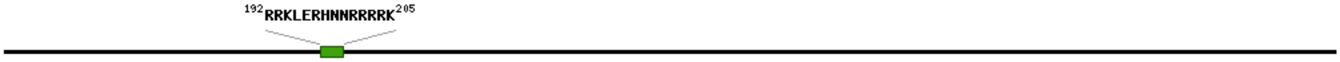

Posterior Graphic. Threshold Marked @ 0.6

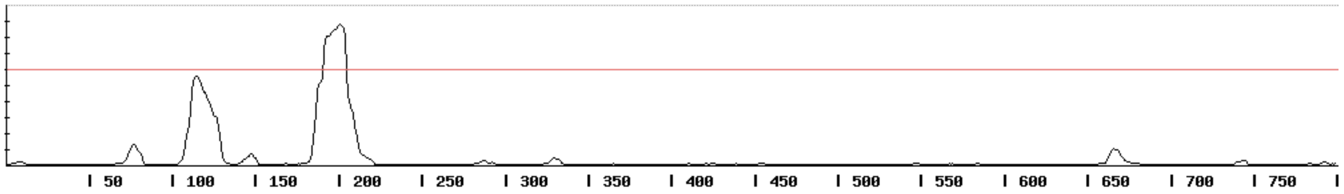

Predictions for AtSPL8

Viterbi Path

Viterbi Path

Posterior @ 0.6

Posterior @ 0.6

Posterior Graphic. Threshold

Marked @ 0.6

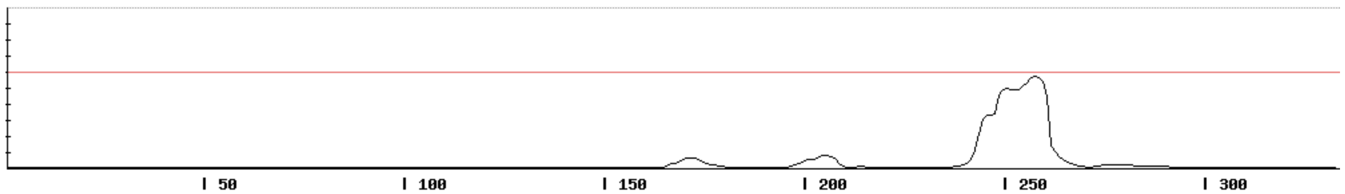

Predictions for AmSPL8

Viterbi Path	
Viterbi Path	
Posterior @ 0.6	235 - HNRRRR - 240
Posterior @ 0.6	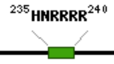
Posterior Graphic. Threshold Marked @ 0.6	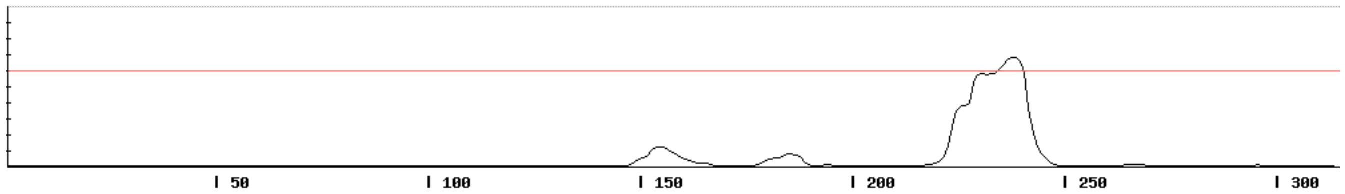

Predictions for MuSPL8

Viterbi Path	
Viterbi Path	
Posterior @ 0.6	
Posterior @ 0.6	
Posterior Graphic. Threshold Marked @ 0.6	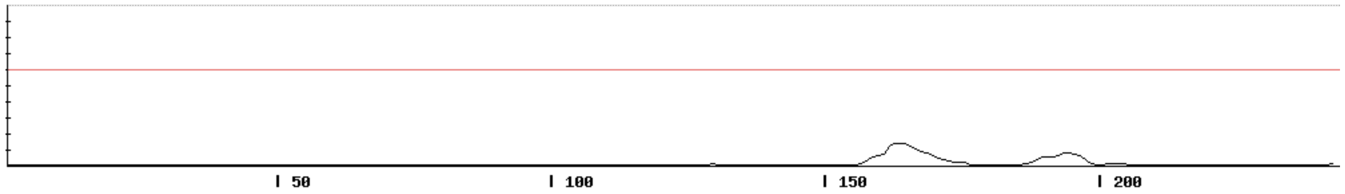

Predictions for AbSPL8

Viterbi Path	
Viterbi Path	

Posterior @ 0.6

136 - HNRRRR - 141

Posterior @ 0.6

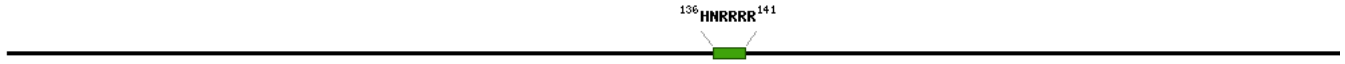

Posterior Graphic. Threshold Marked @ 0.6

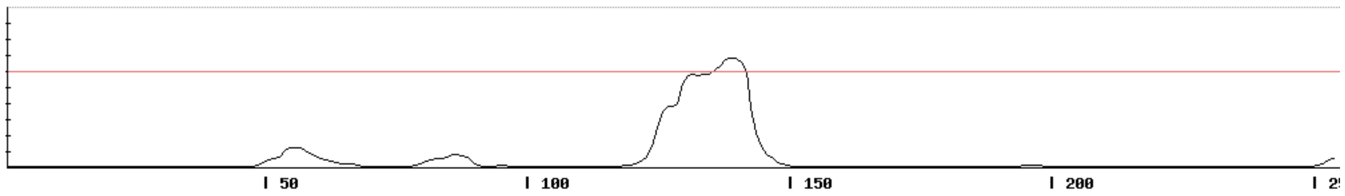

Predictions for ClSPL8

Viterbi Path

Viterbi Path

Posterior @ 0.6

241 - NRRRR - 245

Posterior @ 0.6

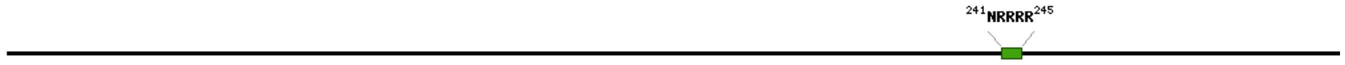

Posterior Graphic. Threshold Marked @ 0.6

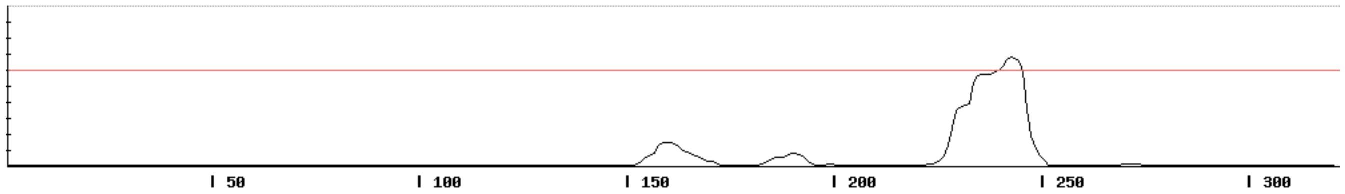

Predictions for CitSPL8

Viterbi Path

Viterbi Path

Posterior @ 0.6

249 - HNRRRR - 254

Posterior @ 0.6

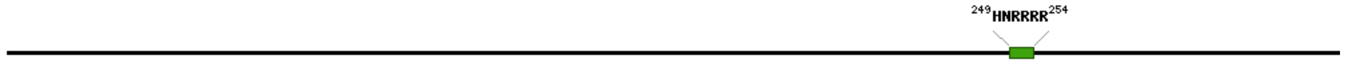

Posterior Graphic. Threshold Marked @ 0.6

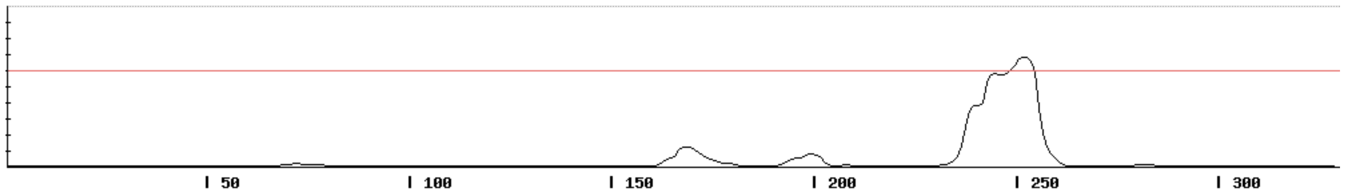

Predictions for PtSPL8

Viterbi Path	
Viterbi Path	
Posterior @ 0.6	252 - HNRRRR - 257
Posterior @ 0.6	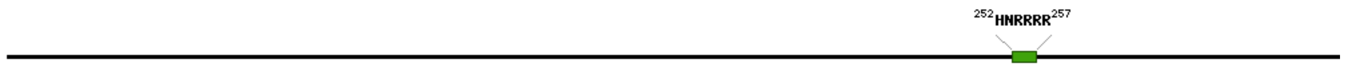
Posterior Graphic. Threshold Marked @ 0.6	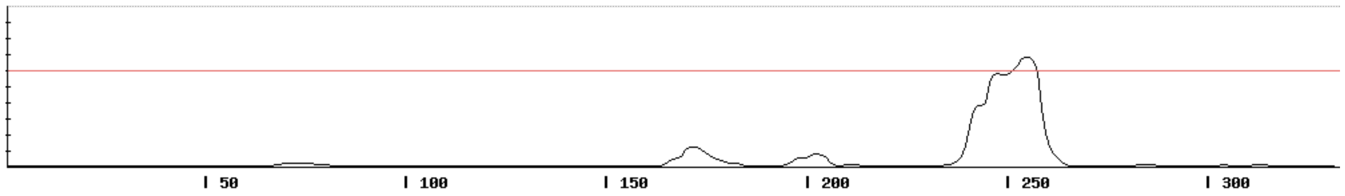

Predictions for FhSPL8

Viterbi Path	
Viterbi Path	
Posterior @ 0.6	248 - HNRRRR - 253
Posterior @ 0.6	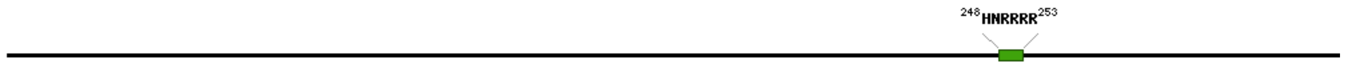
Posterior Graphic. Threshold Marked @ 0.6	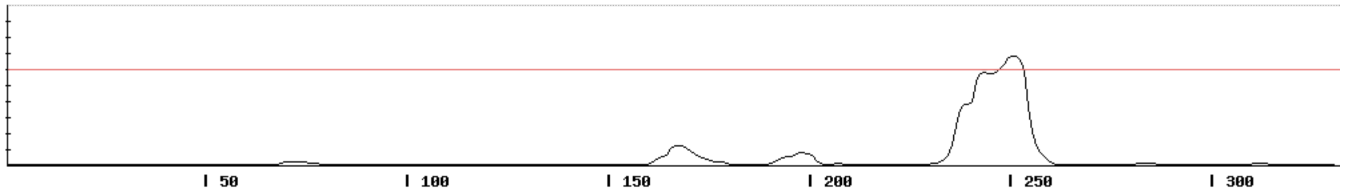

Predictions for CmsSPL8

Viterbi Path	
Viterbi Path	
Posterior @ 0.6	249 - HNRRRR - 254
Posterior @ 0.6	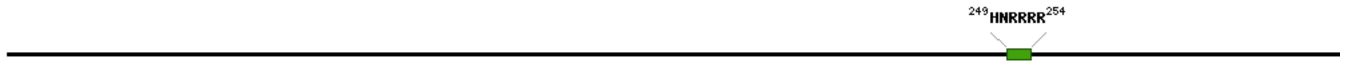
Posterior Graphic. Threshold	

Marked
@ 0.6

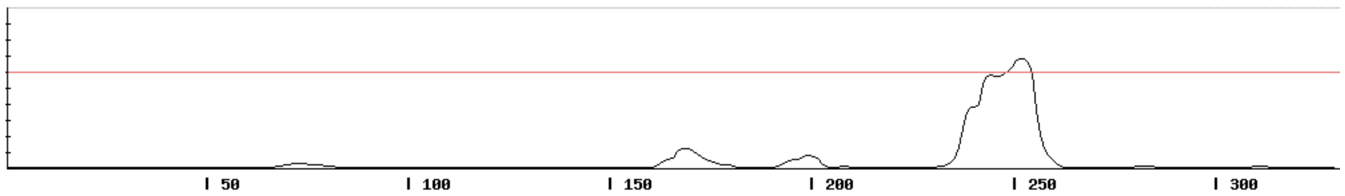

Predictions for CicSPL8

Viterbi Path

Viterbi Path

Posterior @ 0.6

250 - HNRRRR - 255

Posterior @ 0.6

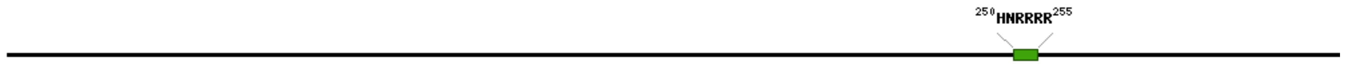

Posterior Graphic. Threshold Marked @ 0.6

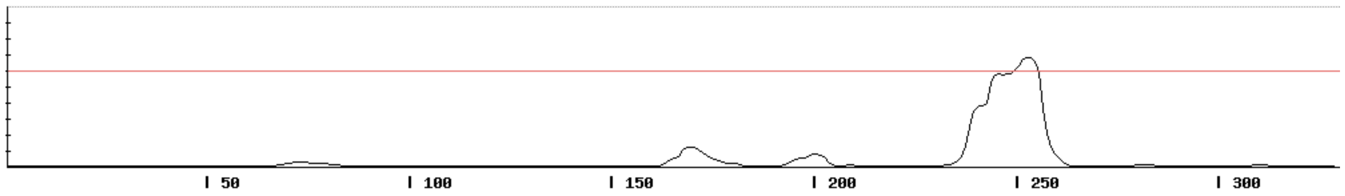

Predictions for CsSPL8

Viterbi Path

Viterbi Path

Posterior @ 0.6

249 - HNRRRR - 254

Posterior @ 0.6

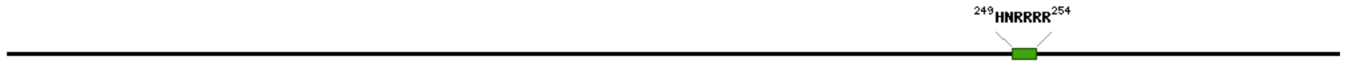

Posterior Graphic. Threshold Marked @ 0.6

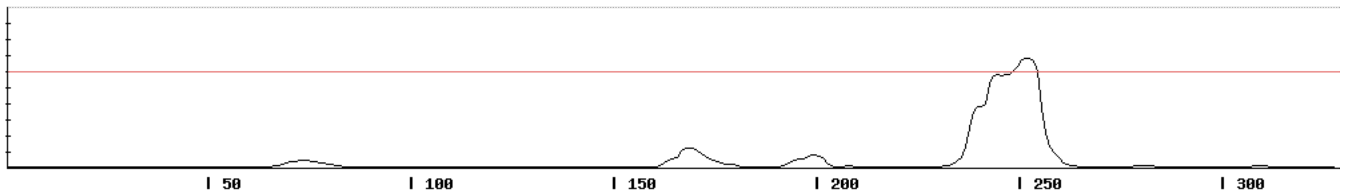

Predictions for CrSPL8

Viterbi Path

Viterbi Path

Posterior @ 0.6

Posterior @ 0.6

Posterior Graphic. Threshold Marked @ 0.6

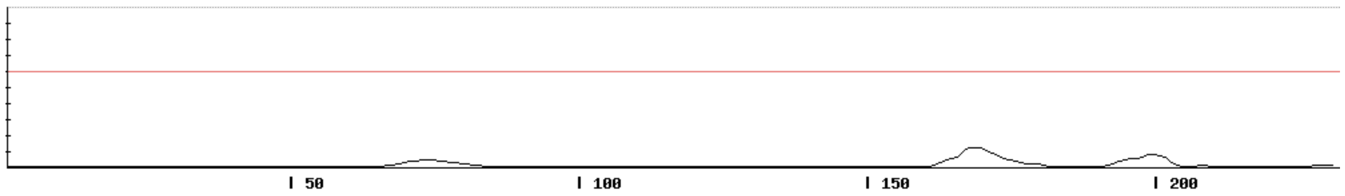

Predictions for ChSPL8

Viterbi Path

Viterbi Path

Posterior @ 0.6

136 - HNRRRR - 141

Posterior @ 0.6

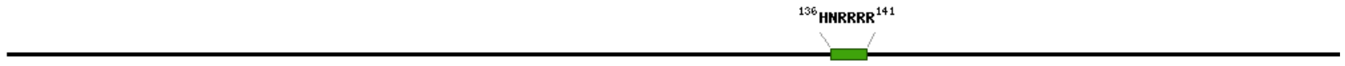

Posterior Graphic. Threshold Marked @ 0.6

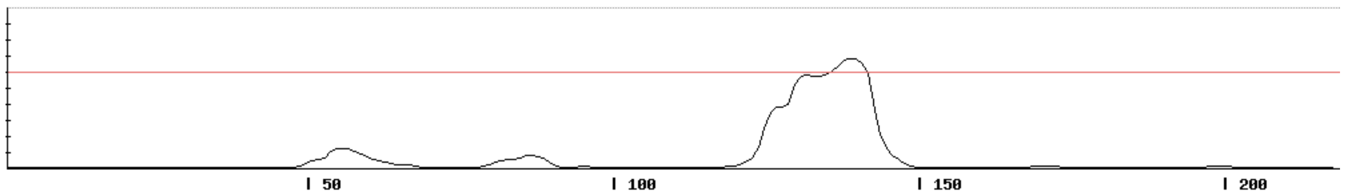

Predictions for CmjSPL8

Viterbi Path

Viterbi Path

Posterior @ 0.6

249 - HNRRRR - 254

Posterior @ 0.6

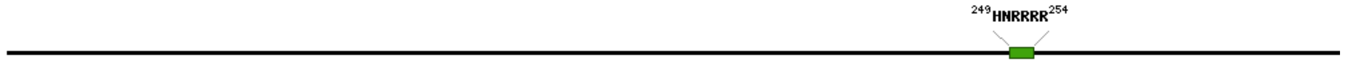

Posterior Graphic. Threshold Marked @ 0.6

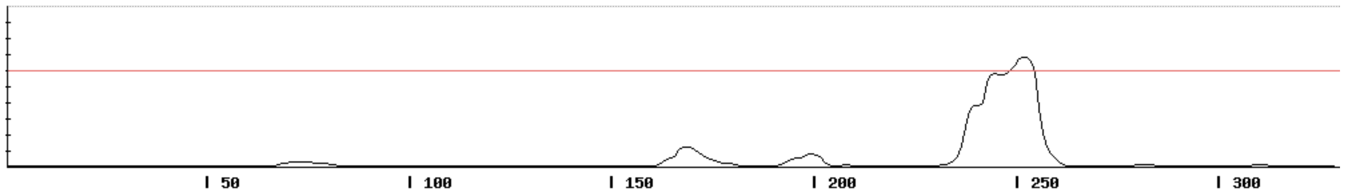

Predictions for CzpSPL8

Viterbi Path	
Viterbi Path	
Posterior @ 0.6	247 - HNRRRR - 252
Posterior @ 0.6	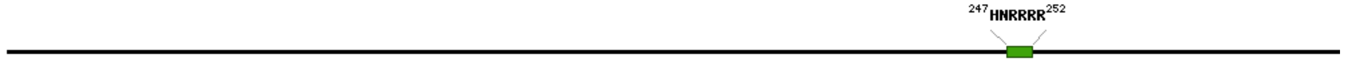
Posterior Graphic. Threshold Marked @ 0.6	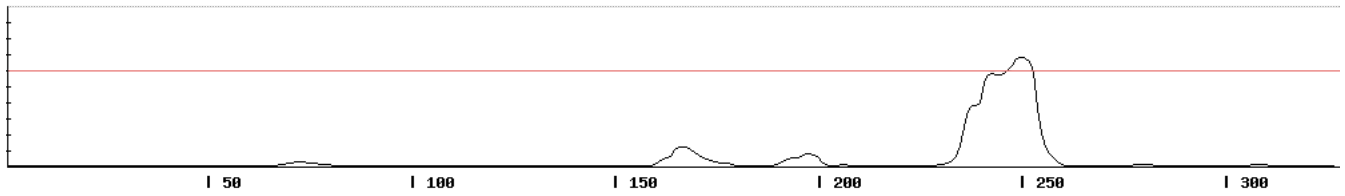

Predictions for CmSPL8

Viterbi Path	
Viterbi Path	
Posterior @ 0.6	249 - HNRRRR - 254
Posterior @ 0.6	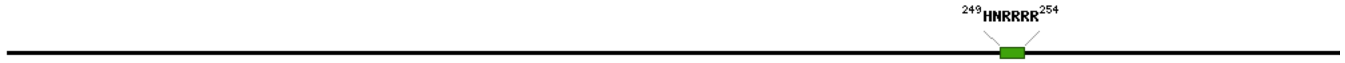
Posterior Graphic. Threshold Marked @ 0.6	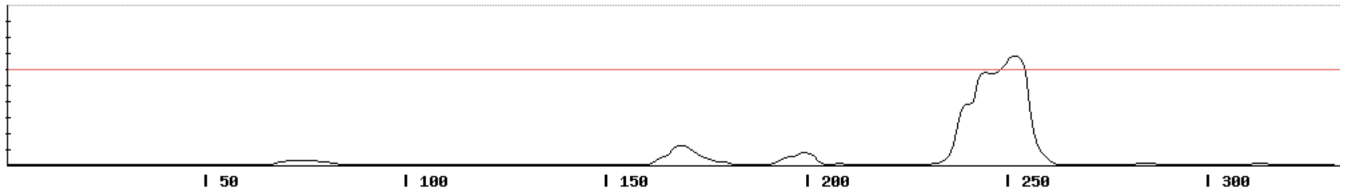

Predictions for AtSPL11

Viterbi Path	
Viterbi Path	
Posterior @ 0.6	236 - RKRLSHHNARRRK - 248
Posterior @ 0.6	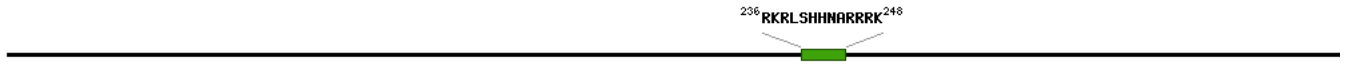
Posterior Graphic. Threshold	

Marked
@ 0.6

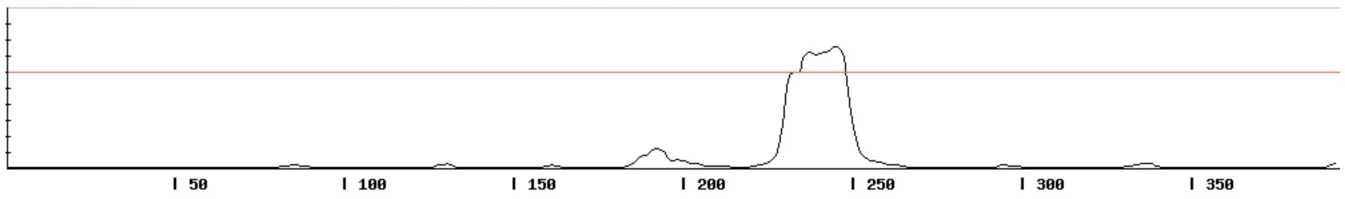

Predictions for AmSPL11

Viterbi Path	
Viterbi Path	
Posterior @ 0.6	255 - ARRRK - 259
Posterior @ 0.6	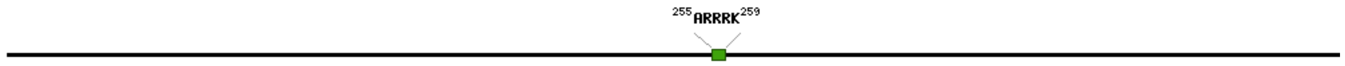
Posterior Graphic. Threshold Marked @ 0.6	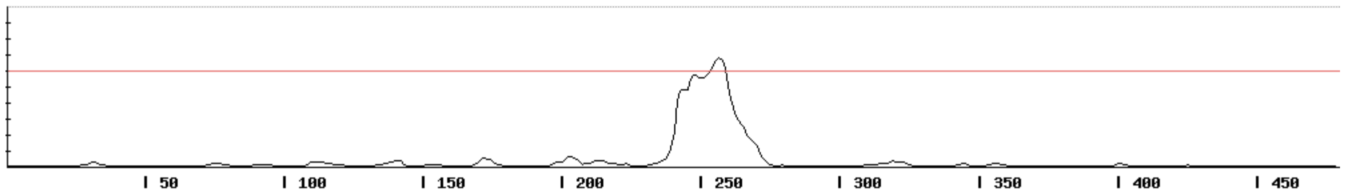

Predictions for MuSPL11

Viterbi Path	
Viterbi Path	
Posterior @ 0.6	
Posterior @ 0.6	
Posterior Graphic. Threshold Marked @ 0.6	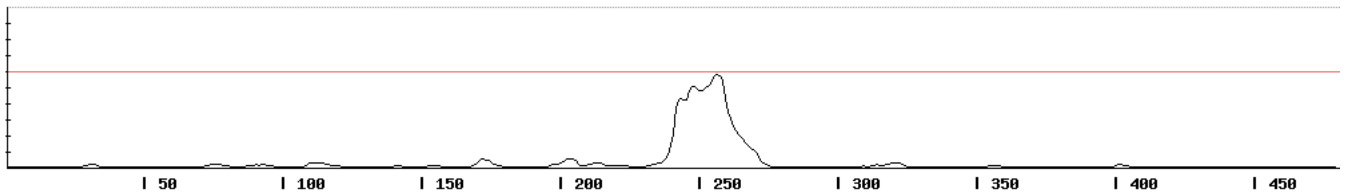

Predictions for AbSPL11

Viterbi Path	
Viterbi Path	

Posterior
@ 0.6

Posterior
@ 0.6

Posterior
Graphic.
Threshold
Marked
@ 0.6

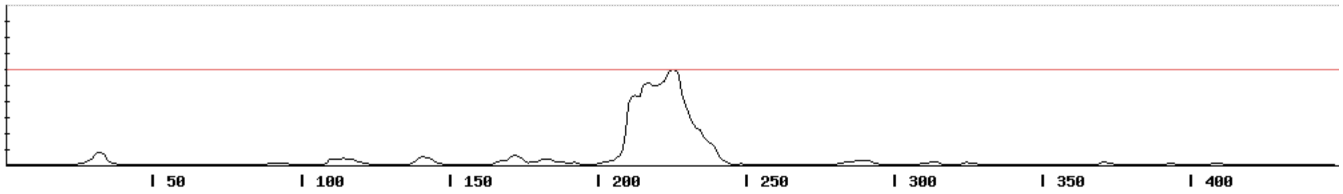

Predictions for ClSPL11

Viterbi
Path

Viterbi
Path

Posterior
@ 0.6

Posterior
@ 0.6

Posterior
Graphic.
Threshold
Marked
@ 0.6

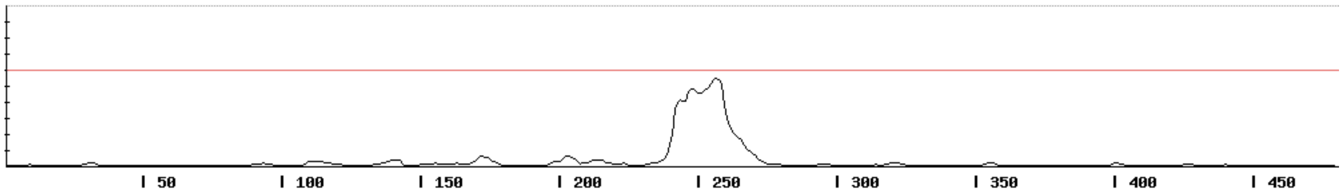

Predictions for CitSPL11

Viterbi
Path

Viterbi
Path

Posterior
@ 0.6

Posterior
@ 0.6

Posterior
Graphic.
Threshold
Marked
@ 0.6

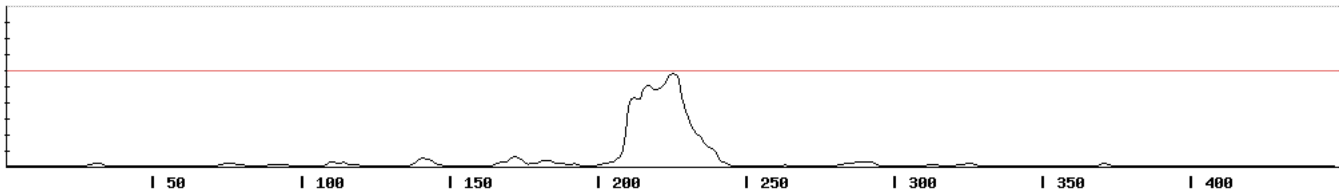

Predictions for PtSPL11

Viterbi Path

Viterbi Path

Posterior @ 0.6

Posterior @ 0.6

Posterior Graphic. Threshold Marked @ 0.6

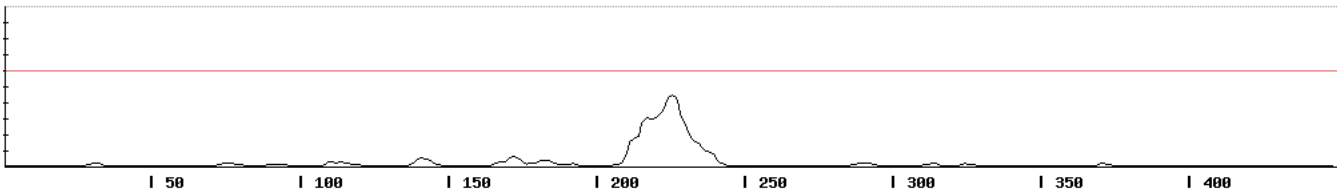

Predictions for FhSPL11

Viterbi Path

Viterbi Path

Posterior @ 0.6

Posterior @ 0.6

Posterior Graphic. Threshold Marked @ 0.6

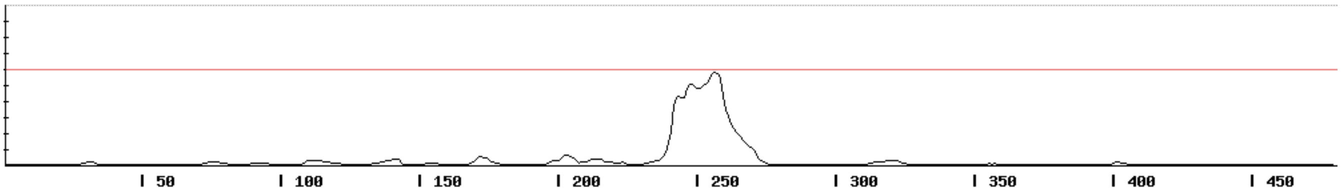

Predictions for CmsSPL11

Viterbi Path

Viterbi Path

Posterior @ 0.6

Posterior @ 0.6

Posterior Graphic. Threshold

Marked
@ 0.6

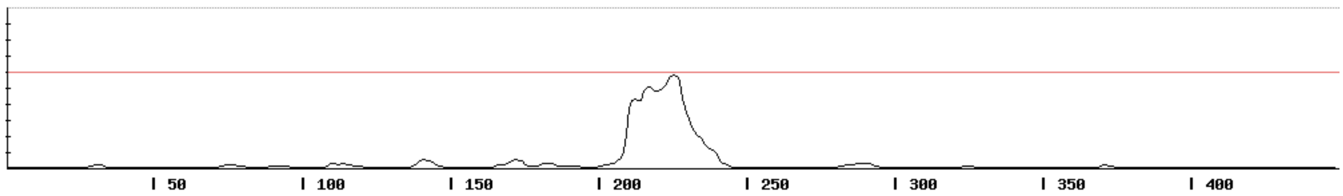

Predictions for CicSPL11

Viterbi
Path

Viterbi
Path

Posterior
@ 0.6

Posterior
@ 0.6

Posterior
Graphic.
Threshold
Marked
@ 0.6

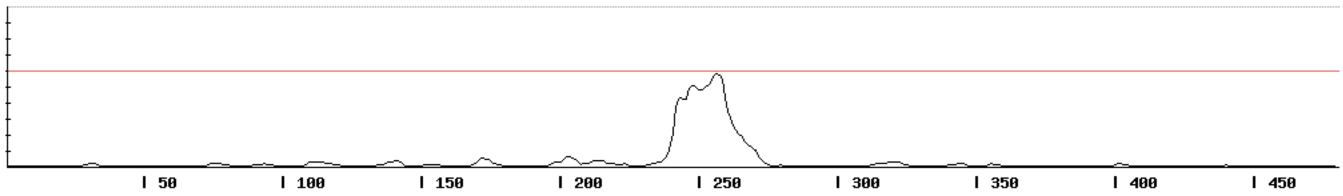

Predictions for CsSPL11

Viterbi
Path

Viterbi
Path

Posterior
@ 0.6

Posterior
@ 0.6

Posterior
Graphic.
Threshold
Marked
@ 0.6

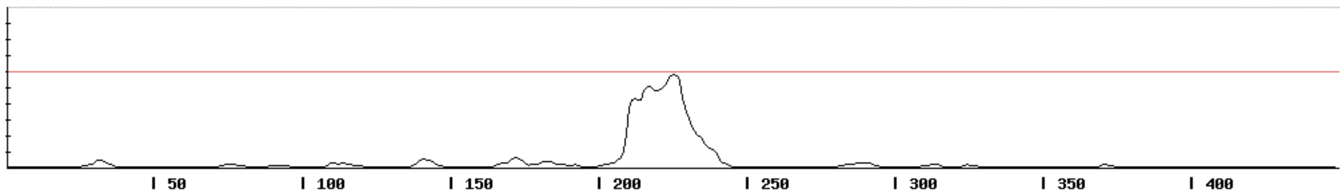

Predictions for CrSPL11

Viterbi
Path

Viterbi
Path

Posterior
@ 0.6

Posterior
@ 0.6

Posterior
Graphic.
Threshold
Marked
@ 0.6

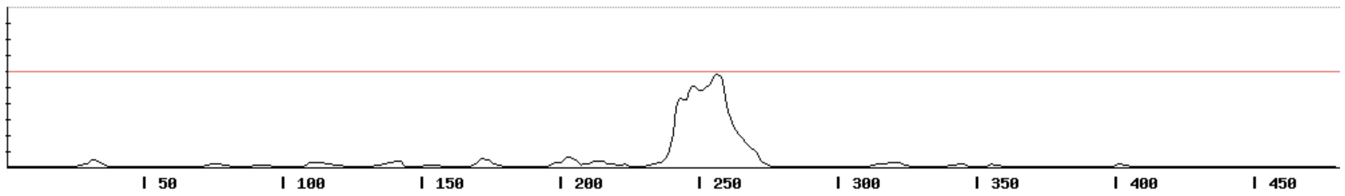

Predictions for ChSPL11

Viterbi
Path

Viterbi
Path

Posterior
@ 0.6

Posterior
@ 0.6

Posterior
Graphic.
Threshold
Marked
@ 0.6

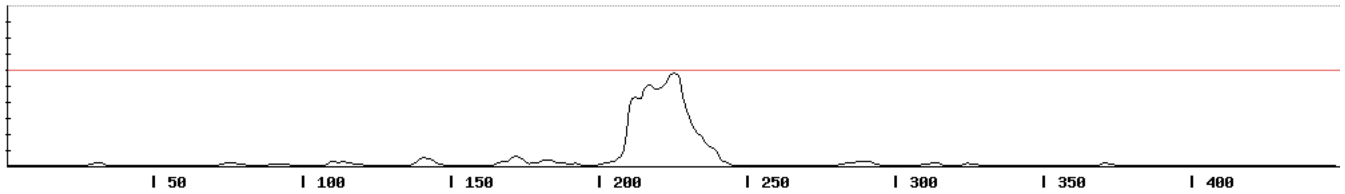

Predictions for CmjSPL11

Viterbi
Path

Viterbi
Path

Posterior
@ 0.6

Posterior
@ 0.6

Posterior
Graphic.
Threshold
Marked
@ 0.6

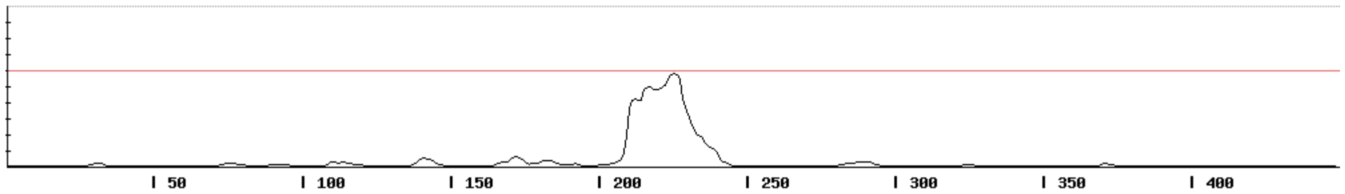

Predictions for CmSPL11

Viterbi Path

Viterbi Path

Posterior @ 0.6

Posterior @ 0.6

Posterior Graphic. Threshold Marked @ 0.6

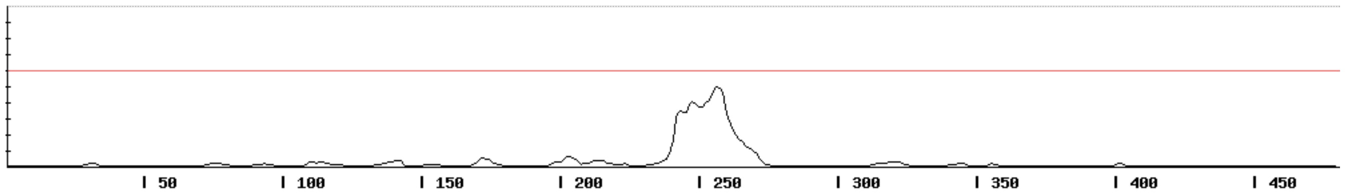

Predictions for AtSPL2

Viterbi Path

Viterbi Path

Posterior @ 0.6

231 - RRLSDHNARRRKP - 243

Posterior @ 0.6

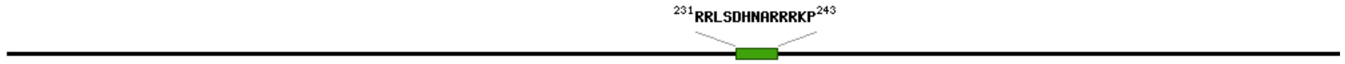

Posterior Graphic. Threshold Marked @ 0.6

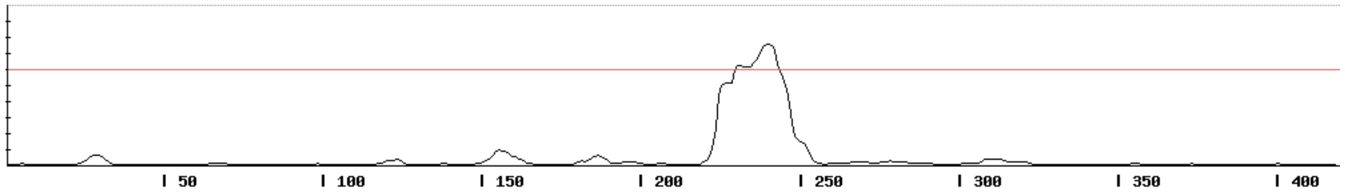

Predictions for AtSPL3

Viterbi Path

Viterbi Path

Posterior @ 0.6

Posterior @ 0.6

Posterior Graphic. Threshold

Marked @ 0.6

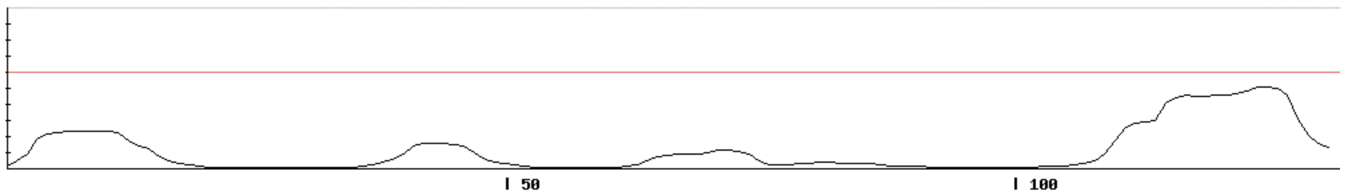

Predictions for AmSPL3

Viterbi Path

Viterbi Path

Posterior @ 0.6

37 - IKKKGKR - 43

Posterior @ 0.6

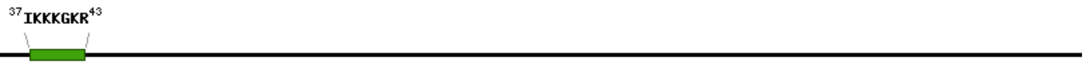

Posterior Graphic. Threshold Marked @ 0.6

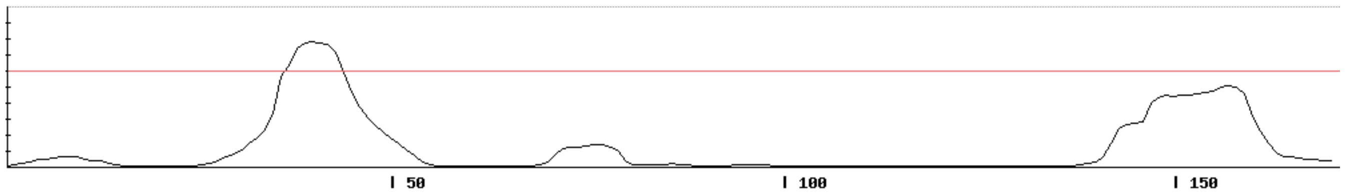

Predictions for MuSPL3

Viterbi Path

Viterbi Path

Posterior @ 0.6

36 - IKKKGKR - 42

Posterior @ 0.6

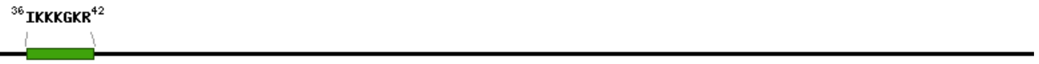

Posterior Graphic. Threshold Marked @ 0.6

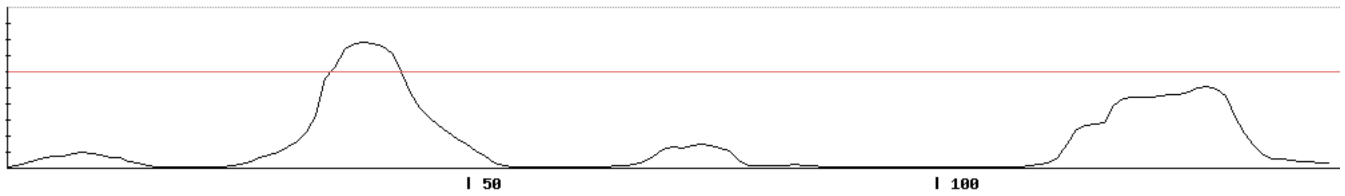

Predictions for AbSPL3

Viterbi Path

Viterbi Path

Posterior @ 0.6 — 38 - KKKGK - 42

Posterior @ 0.6

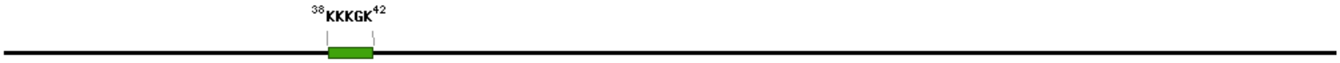

Posterior Graphic. Threshold Marked @ 0.6

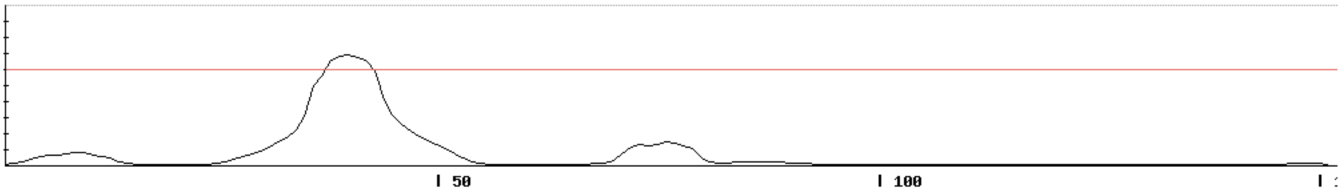

Predictions for ClSPL3

Viterbi Path

Viterbi Path

Posterior @ 0.6 — 33 - KKKGKR - 38

Posterior @ 0.6

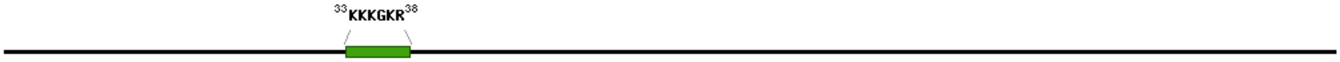

Posterior Graphic. Threshold Marked @ 0.6

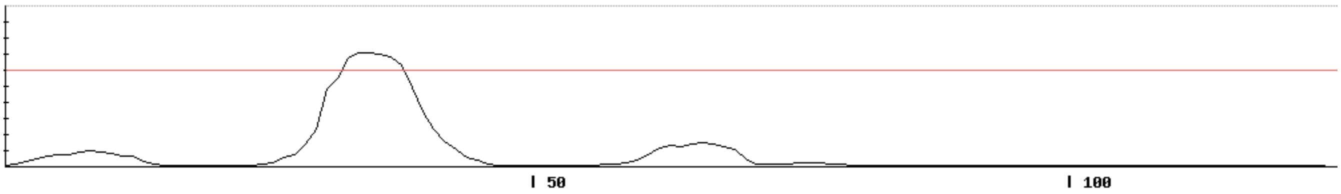

Predictions for CitSPL3

Viterbi Path

Viterbi Path

Posterior @ 0.6 — 37 - IKKKGKR - 43

Posterior @ 0.6

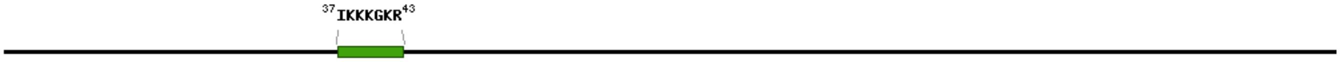

Posterior Graphic. Threshold Marked @ 0.6

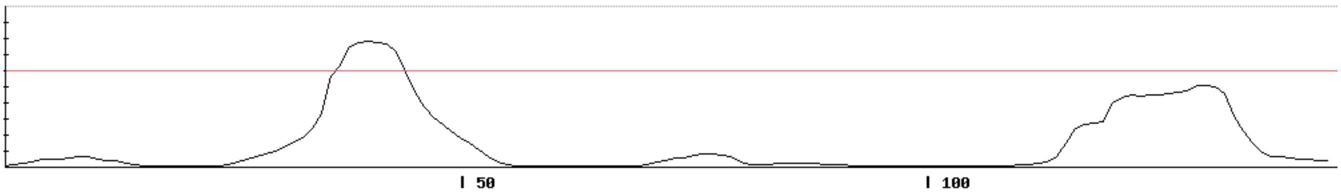

Predictions for PtSPL3

Viterbi Path

Viterbi Path

Posterior @ 0.6

39 - KKKGRR - 44

Posterior @ 0.6

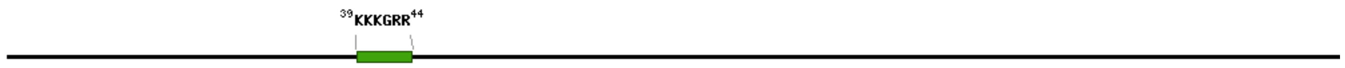

Posterior Graphic. Threshold Marked @ 0.6

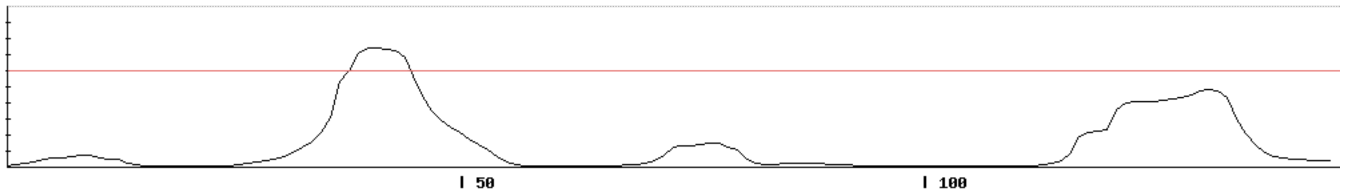

Predictions for FhSPL3

Viterbi Path

Viterbi Path

Posterior @ 0.6

37 - IKKKGKR - 43

Posterior @ 0.6

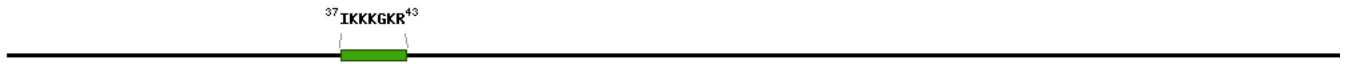

Posterior Graphic. Threshold Marked @ 0.6

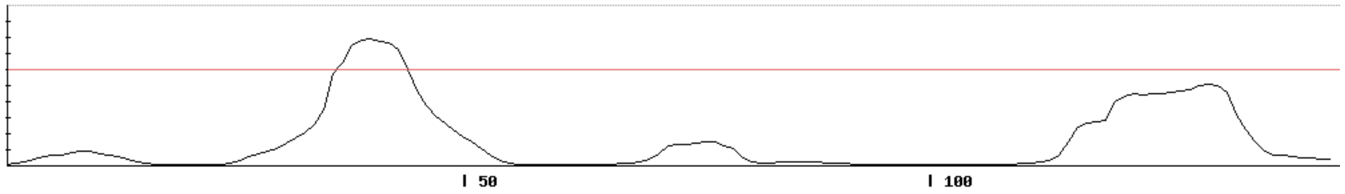

Predictions for CmsSPL3

Viterbi Path

Viterbi Path

Posterior @ 0.6

37 - IKKKGKR - 43

Posterior @ 0.6

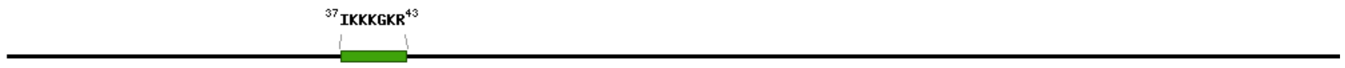

Posterior Graphic. Threshold

Marked
@ 0.6

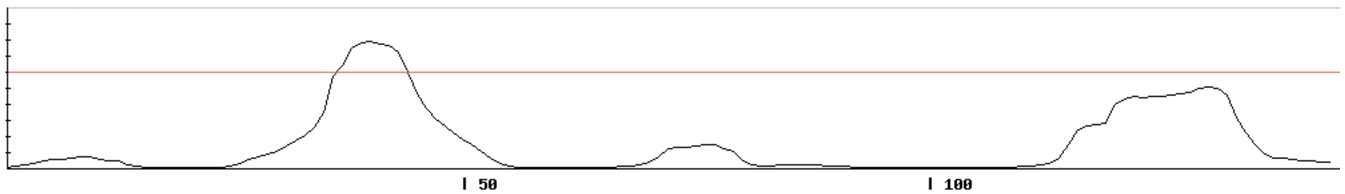

Predictions for CicSPL3

Viterbi Path

Viterbi Path

Posterior @ 0.6: 37 - IKKKGKR - 43

Posterior @ 0.6

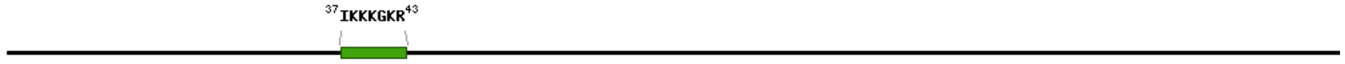

Posterior Graphic. Threshold Marked @ 0.6

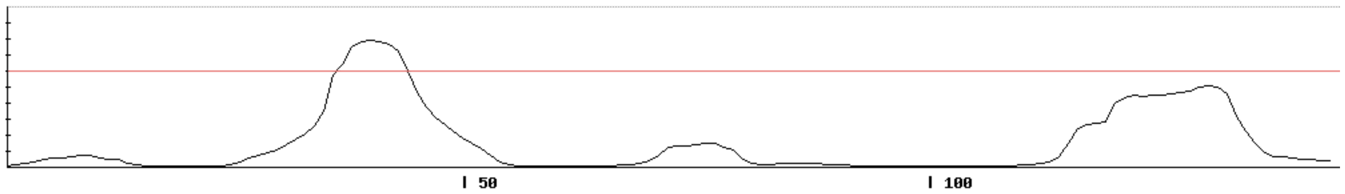

Predictions for CsSPL3

Viterbi Path

Viterbi Path

Posterior @ 0.6: 89 - IKKKGKRG - 96

Posterior @ 0.6

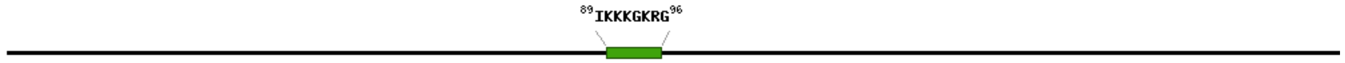

Posterior Graphic. Threshold Marked @ 0.6

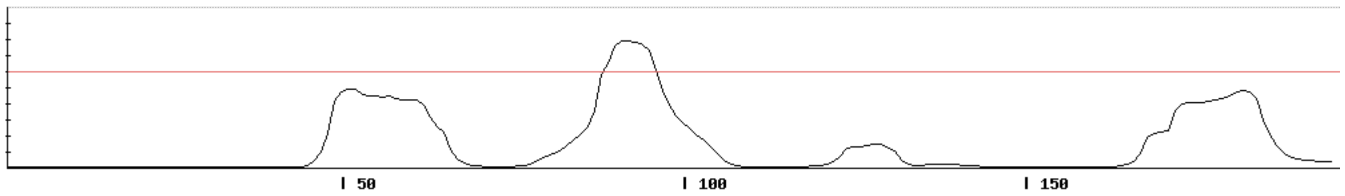

Predictions for CrSPL3

Viterbi Path

Viterbi Path

Posterior @ 0.6 | 37 - IKKKGKR - 43

Posterior @ 0.6

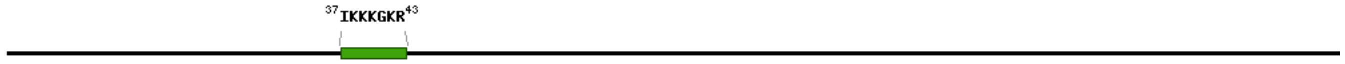

Posterior Graphic. Threshold Marked @ 0.6

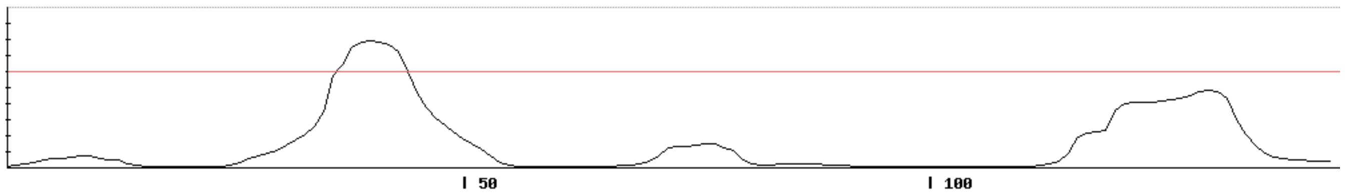

Predictions for ChSPL3

Viterbi Path

Viterbi Path

Posterior @ 0.6 | 36 - KVKKKGKRRS - 45

Posterior @ 0.6

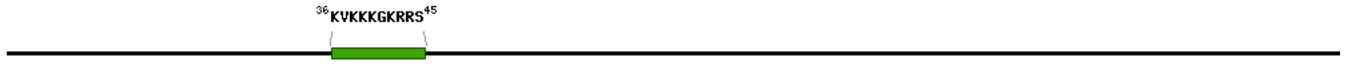

Posterior Graphic. Threshold Marked @ 0.6

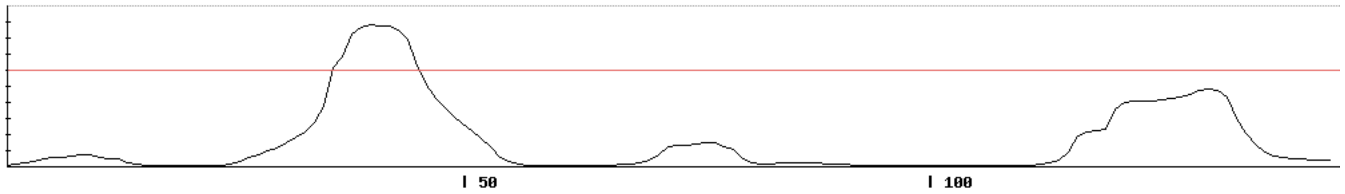

Predictions for CmjSPL3

Viterbi Path

Viterbi Path

Posterior @ 0.6 | 89 - IKKKGKRG - 96

Posterior @ 0.6

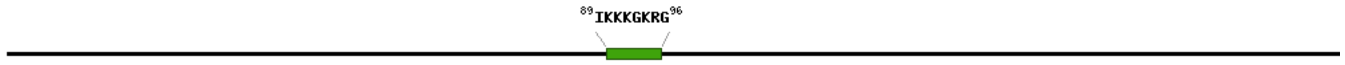

Posterior Graphic. Threshold Marked @ 0.6

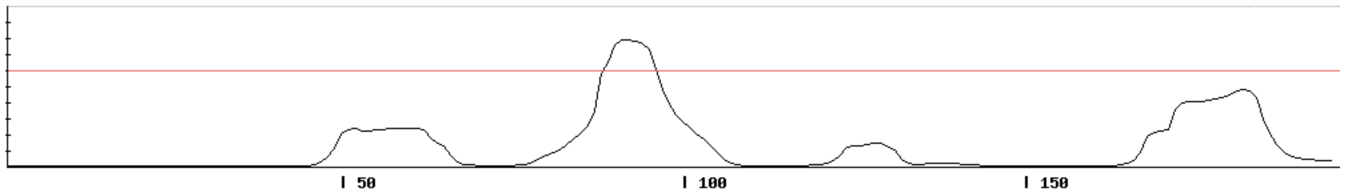

Predictions for CzpSPL3

Viterbi Path

Viterbi Path

Posterior @ 0.6 — 89 - IKKKGKRG - 96

Posterior @ 0.6

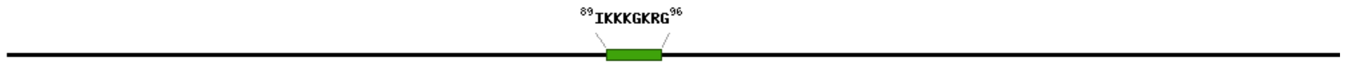

Posterior Graphic. Threshold Marked @ 0.6

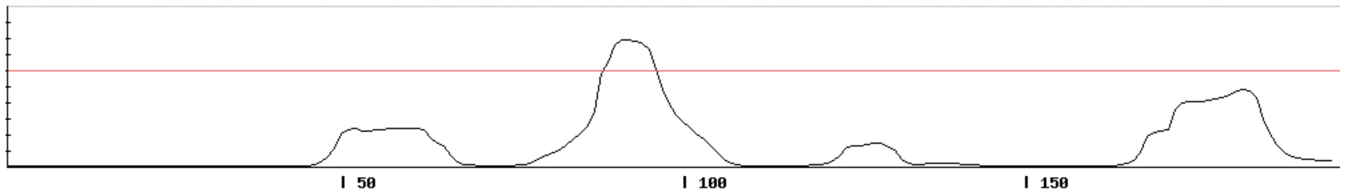

Predictions for CmSPL3

Viterbi Path

Viterbi Path

Posterior @ 0.6 — 37 - IKKKGKR - 43

Posterior @ 0.6

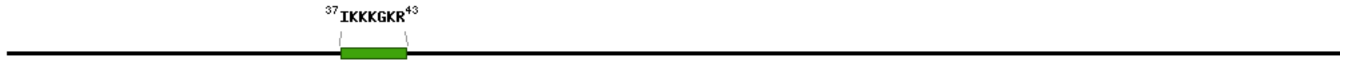

Posterior Graphic. Threshold Marked @ 0.6

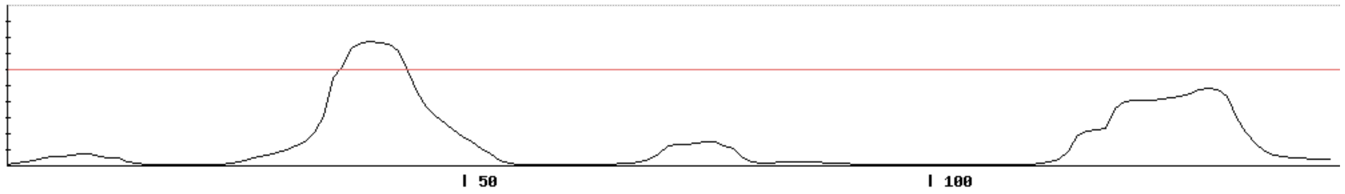

Predictions for AtSPL4

Viterbi Path

Viterbi Path

Posterior @ 0.6

Posterior @ 0.6

Posterior Graphic. Threshold

Marked
@ 0.6

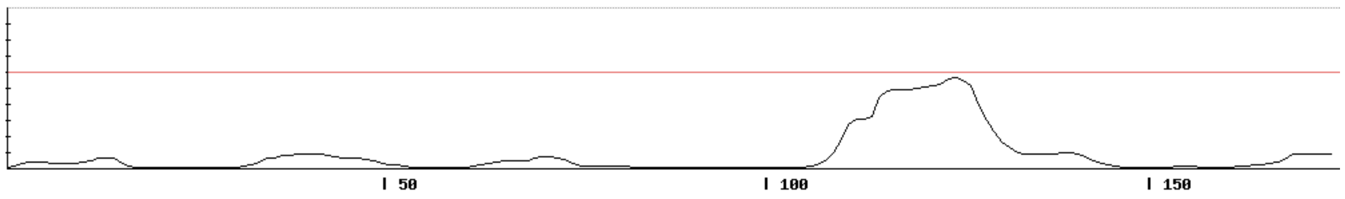

Predictions for AtSPL5

Viterbi
Path

Viterbi
Path

Posterior
@ 0.6

Posterior
@ 0.6

Posterior
Graphic.
Threshold
Marked
@ 0.6

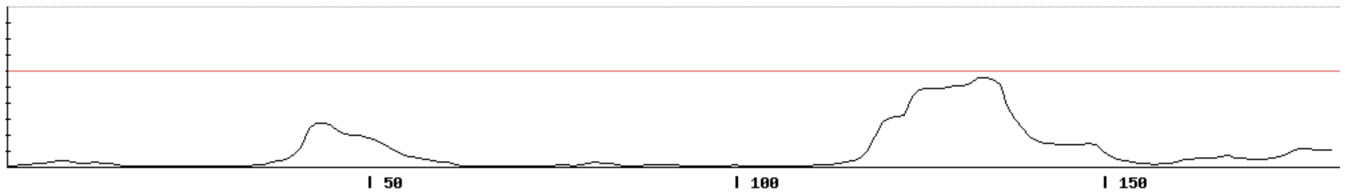

Predictions for AmSPL5

Viterbi
Path

Viterbi
Path

Posterior
@ 0.6

Posterior
@ 0.6

Posterior
Graphic.
Threshold
Marked
@ 0.6

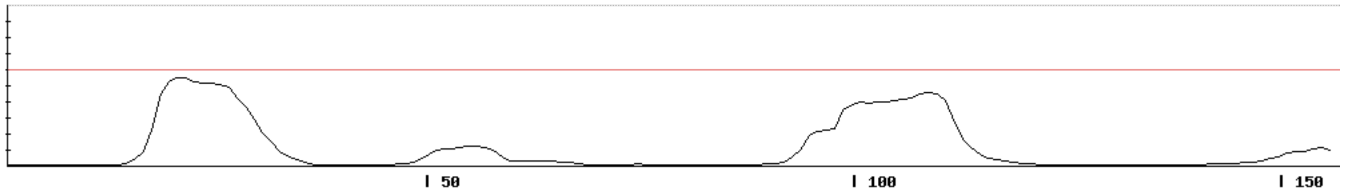

Predictions for MuSPL5

Viterbi
Path

Viterbi
Path

Posterior
@ 0.6

Posterior
@ 0.6

Posterior
Graphic.
Threshold
Marked
@ 0.6

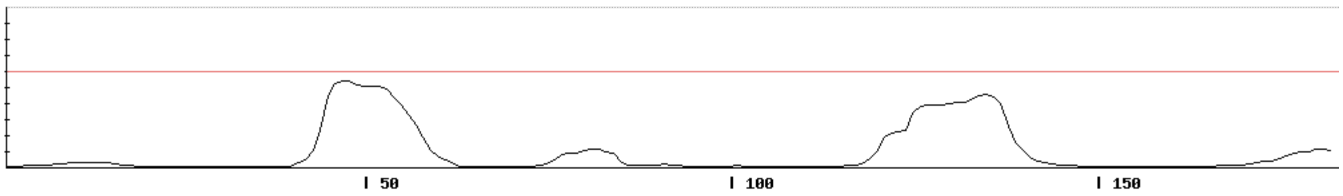

Predictions for AbSPL5

Viterbi
Path

Viterbi
Path

Posterior
@ 0.6

Posterior
@ 0.6

Posterior
Graphic.
Threshold
Marked
@ 0.6

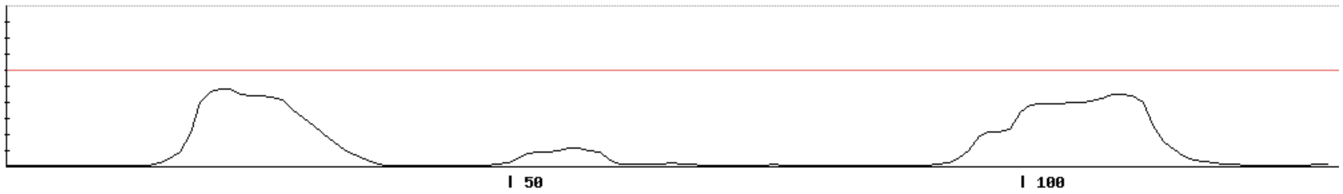

Predictions for ClSPL5

Viterbi
Path

Viterbi
Path

Posterior
@ 0.6

Posterior
@ 0.6

Posterior
Graphic.
Threshold
Marked
@ 0.6

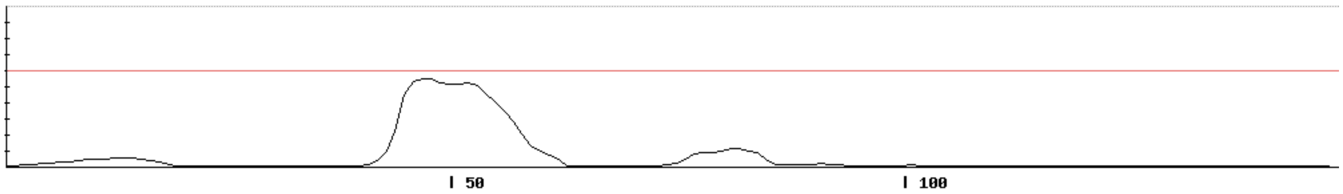

Predictions for CitSPL5

Viterbi Path

Viterbi Path

Posterior @ 0.6

Posterior @ 0.6

Posterior Graphic. Threshold Marked @ 0.6

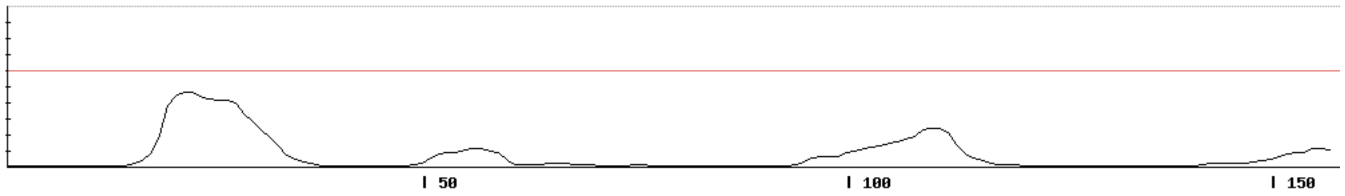

Predictions for PtSPL5

Viterbi Path

Viterbi Path

Posterior @ 0.6

Posterior @ 0.6

Posterior Graphic. Threshold Marked @ 0.6

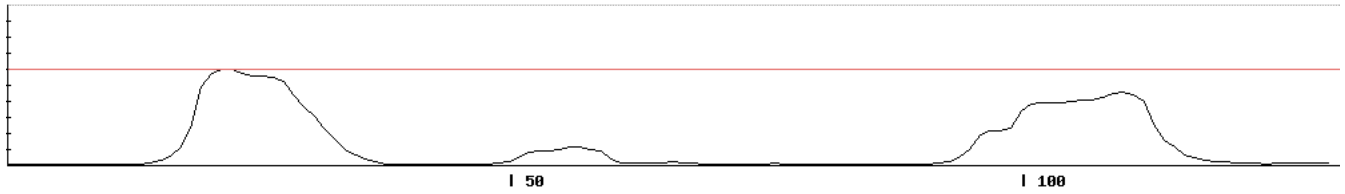

Predictions for FhSPL5

Viterbi Path

Viterbi Path

Posterior @ 0.6

21 - KKKANGR - 27

Posterior @ 0.6

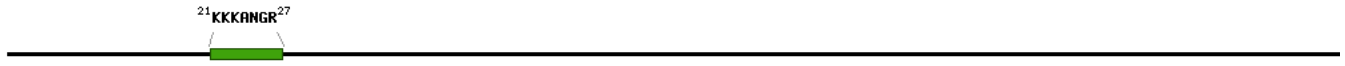

Posterior Graphic. Threshold

Marked
@ 0.6

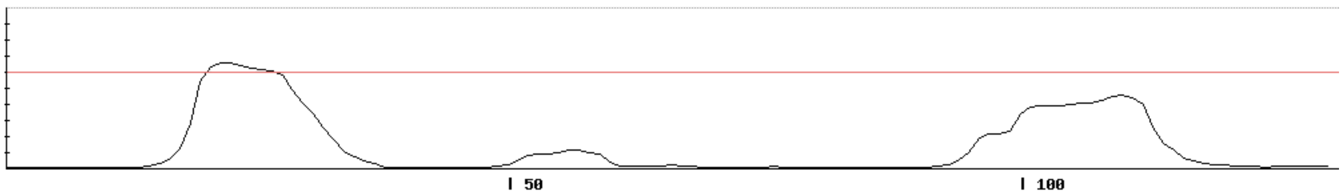

Predictions for CmsSPL5

Viterbi
Path

Viterbi
Path

Posterior
@ 0.6

Posterior
@ 0.6

Posterior
Graphic.
Threshold
Marked
@ 0.6

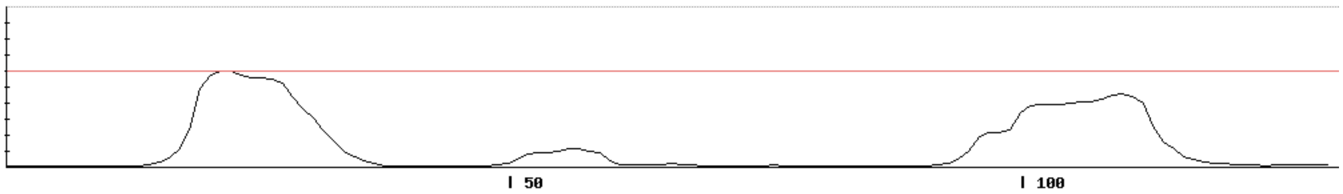

Predictions for CicSPL5

Viterbi
Path

Viterbi
Path

Posterior
@ 0.6

Posterior
@ 0.6

Posterior
Graphic.
Threshold
Marked
@ 0.6

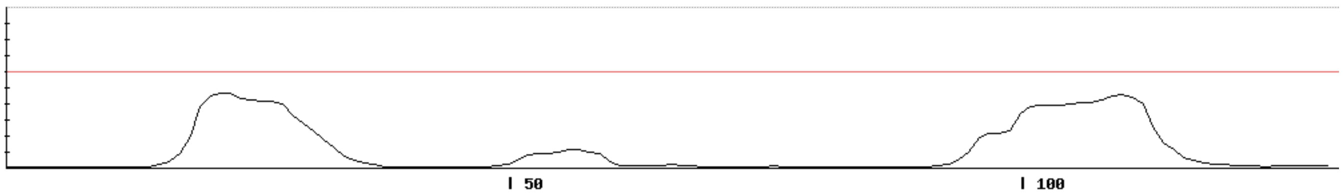

Predictions for CsSPL5

Viterbi
Path

Viterbi
Path

Posterior @ 0.6

Posterior @ 0.6

Posterior Graphic. Threshold Marked @ 0.6

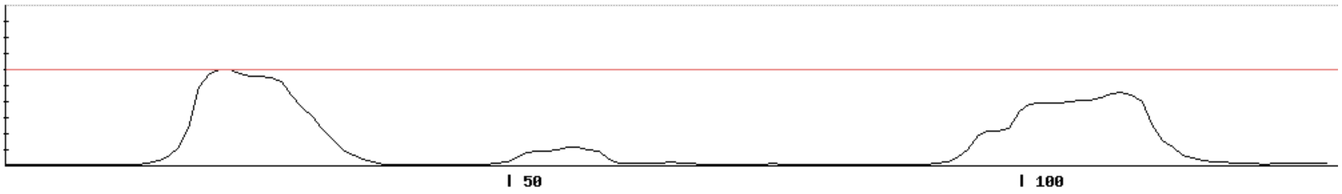

Predictions for CrSPL5

Viterbi Path

Viterbi Path

Posterior @ 0.6

Posterior @ 0.6

Posterior Graphic. Threshold Marked @ 0.6

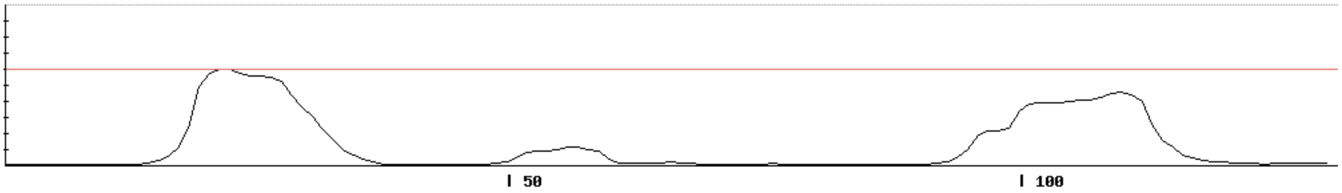

Predictions for ChSPL5

Viterbi Path

Viterbi Path

Posterior @ 0.6

Posterior @ 0.6

Posterior Graphic. Threshold Marked @ 0.6

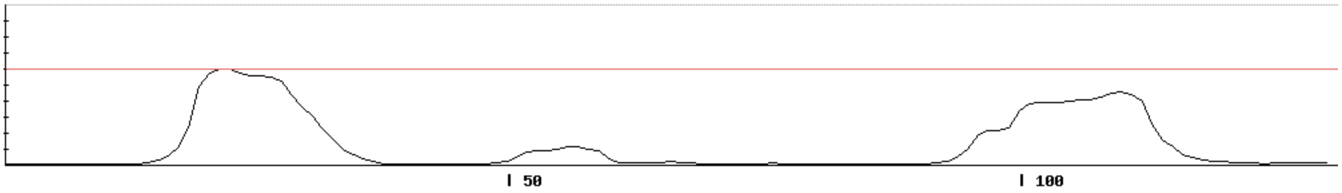

Predictions for CmjSPL5

Viterbi Path

Viterbi Path

Posterior @ 0.6

Posterior @ 0.6

Posterior Graphic. Threshold Marked @ 0.6

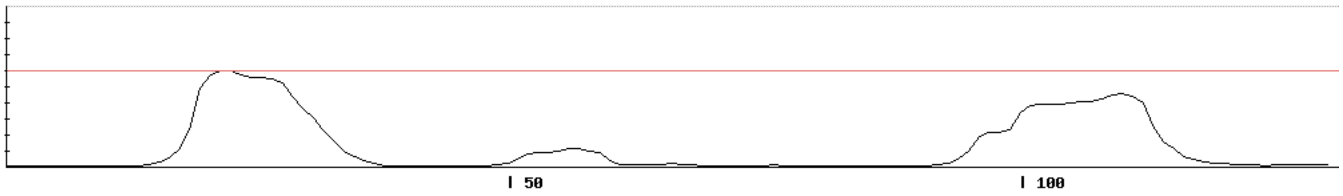

Predictions for CzpSPL5

Viterbi Path

Viterbi Path

Posterior @ 0.6

Posterior @ 0.6

Posterior Graphic. Threshold Marked @ 0.6

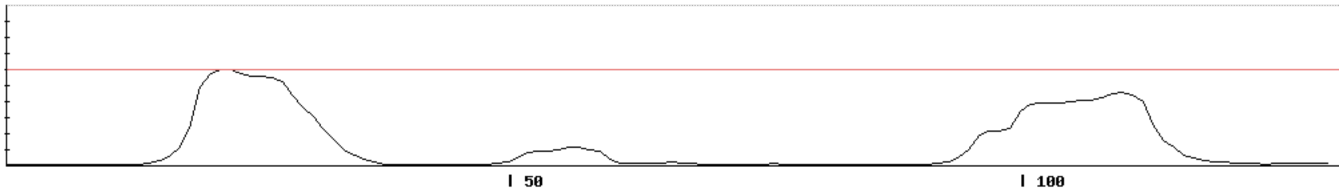

Predictions for CmSPL5

Viterbi Path

Viterbi Path

Posterior @ 0.6

22 - KK - 23

Posterior @ 0.6

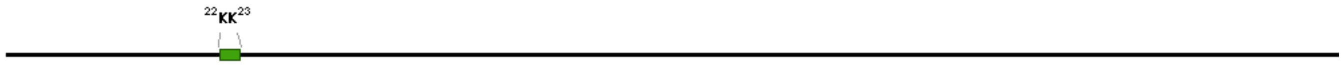

Posterior Graphic. Threshold

Marked
@ 0.6

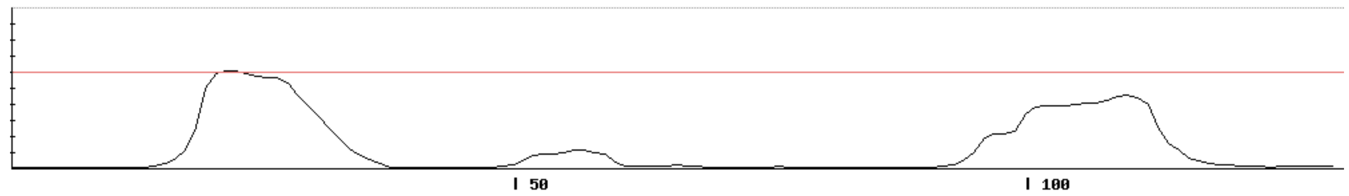

Predictions for AmSPL4

Viterbi
Path

Viterbi
Path

Posterior
@ 0.6

Posterior
@ 0.6

Posterior
Graphic.
Threshold
Marked
@ 0.6

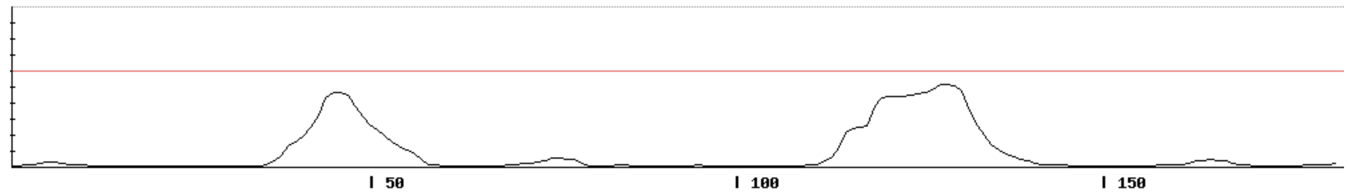

Predictions for AbSPL4

Viterbi
Path

Viterbi
Path

Posterior
@ 0.6

Posterior
@ 0.6

Posterior
Graphic.
Threshold
Marked
@ 0.6

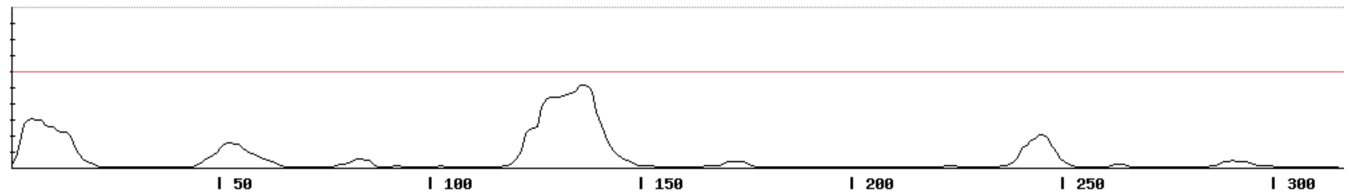

Predictions for ClSPL4

Viterbi
Path

Viterbi
Path

Posterior
@ 0.6

Posterior
@ 0.6

Posterior
Graphic.
Threshold
Marked
@ 0.6

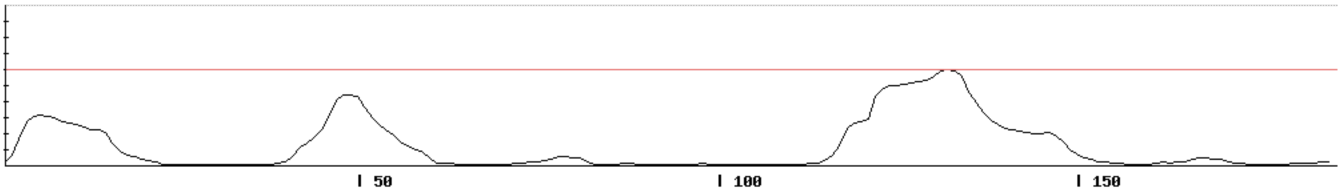

Predictions for CitSPL4

Viterbi
Path

Viterbi
Path

Posterior
@ 0.6

Posterior
@ 0.6

Posterior
Graphic.
Threshold
Marked
@ 0.6

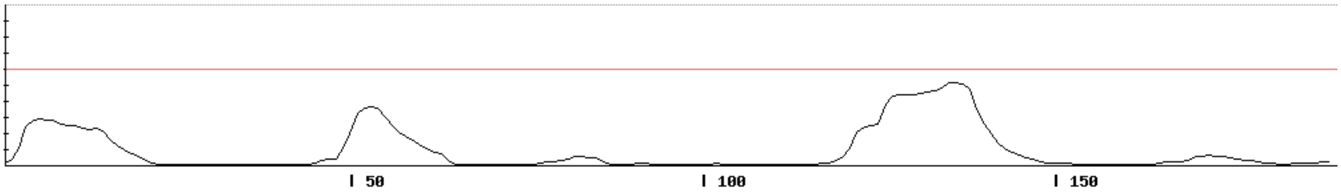

Predictions for PtSPL4

Viterbi
Path

Viterbi
Path

Posterior
@ 0.6

Posterior
@ 0.6

Posterior
Graphic.
Threshold
Marked
@ 0.6

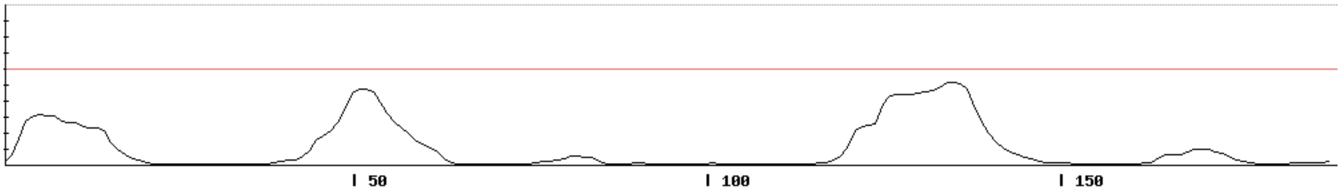

Predictions for FhSPL4

Viterbi Path

Viterbi Path

Posterior @ 0.6

Posterior @ 0.6

Posterior Graphic. Threshold Marked @ 0.6

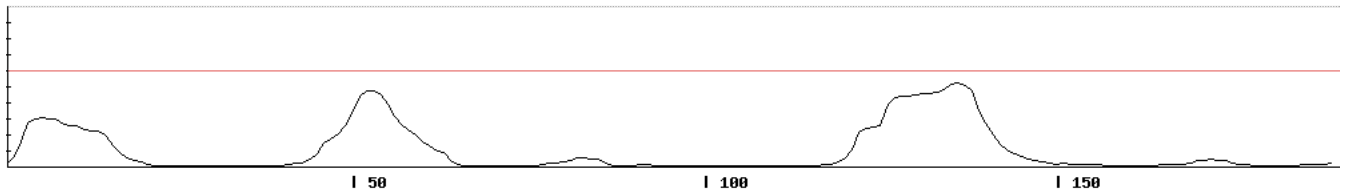

Predictions for CmsSPL4

Viterbi Path

Viterbi Path

Posterior @ 0.6

Posterior @ 0.6

Posterior Graphic. Threshold Marked @ 0.6

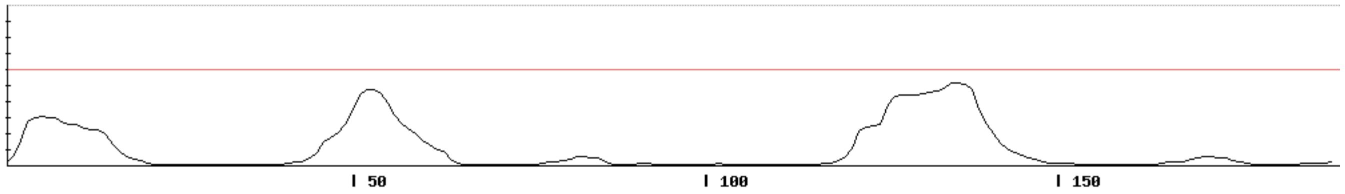

Predictions for CicSPL4

Viterbi Path

Viterbi Path

Posterior @ 0.6

Posterior @ 0.6

Posterior Graphic. Threshold

Marked
@ 0.6

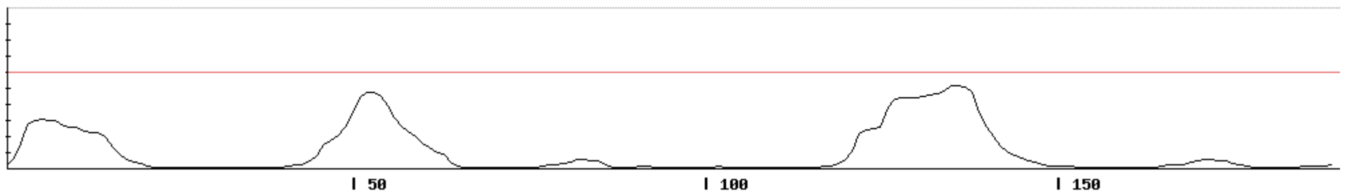

Predictions for CsSPL4

Viterbi
Path

Viterbi
Path

Posterior
@ 0.6

Posterior
@ 0.6

Posterior
Graphic.
Threshold
Marked
@ 0.6

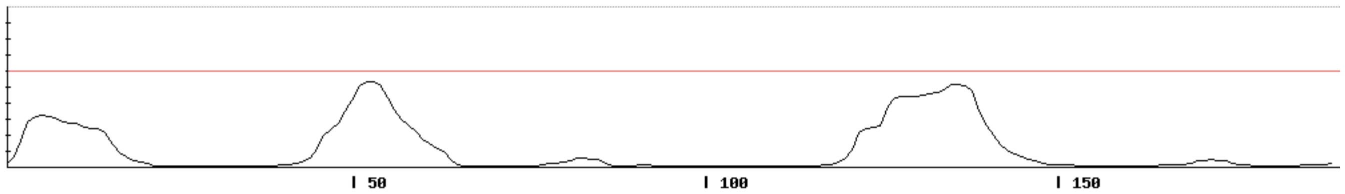

Predictions for CrSPL4

Viterbi
Path

Viterbi
Path

Posterior
@ 0.6

Posterior
@ 0.6

Posterior
Graphic.
Threshold
Marked
@ 0.6

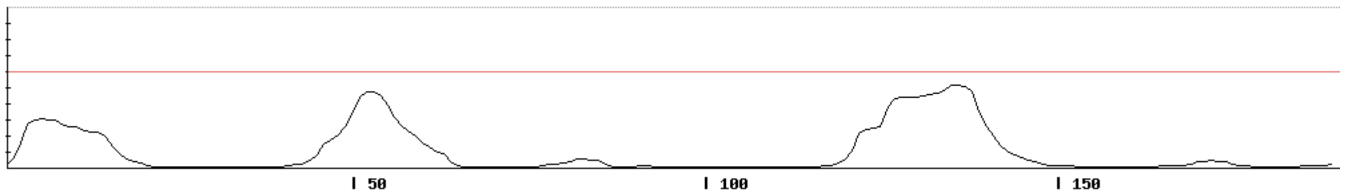

Predictions for ChSPL4

Viterbi
Path

Viterbi
Path

Posterior @ 0.6

Posterior @ 0.6

Posterior Graphic. Threshold Marked @ 0.6

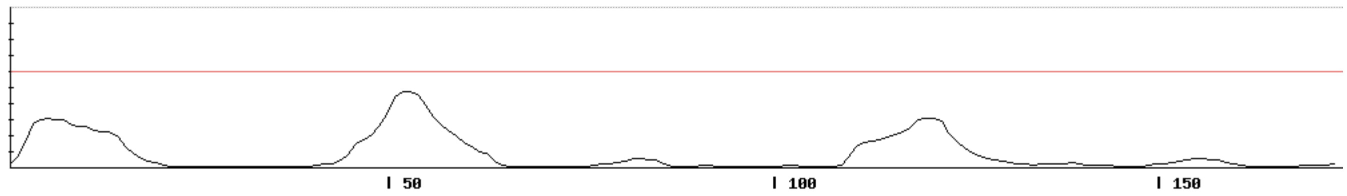

Predictions for CmjSPL4

Viterbi Path

Viterbi Path

Posterior @ 0.6

Posterior @ 0.6

Posterior Graphic. Threshold Marked @ 0.6

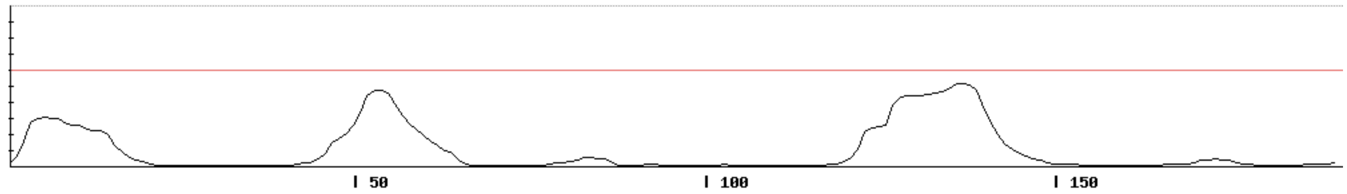

Predictions for CzpSPL4

Viterbi Path

Viterbi Path

Posterior @ 0.6

Posterior @ 0.6

Posterior Graphic. Threshold Marked @ 0.6

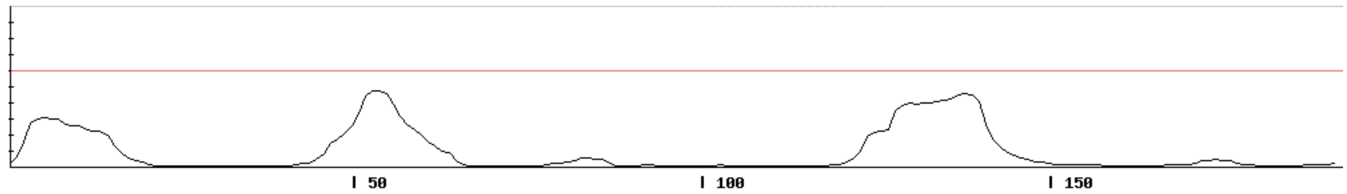

Predictions for CmSPL4

Viterbi Path

Viterbi Path

Posterior @ 0.6

Posterior @ 0.6

Posterior Graphic. Threshold Marked @ 0.6

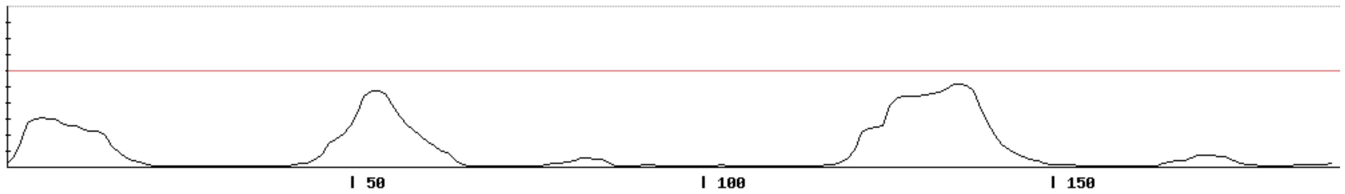

Predictions for AtSPL1

Viterbi Path

Viterbi Path

Posterior @ 0.6

167 - RRRLAGHNKRRRK - 179

Posterior @ 0.6

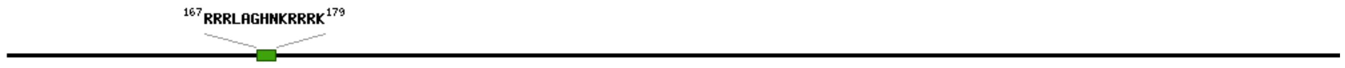

Posterior Graphic. Threshold Marked @ 0.6

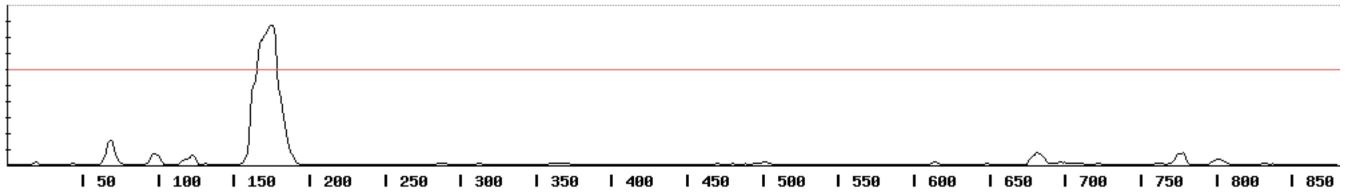

Predictions for AmSPL1

Viterbi Path

Viterbi Path

Posterior @ 0.6

Posterior @ 0.6

Posterior Graphic. Threshold

Marked
@ 0.6

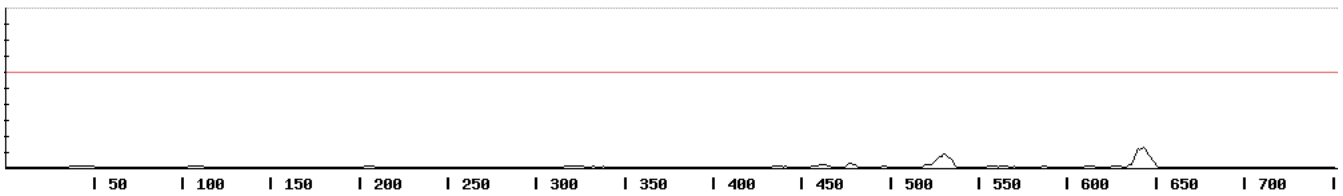

Predictions for MuSPL1

Viterbi Path

Viterbi Path

Posterior @ 0.6

157 - RRRLAGHNKRRRK - 169

Posterior @ 0.6

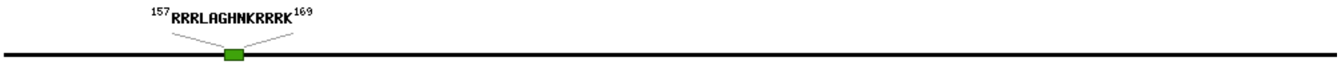

Posterior Graphic. Threshold Marked @ 0.6

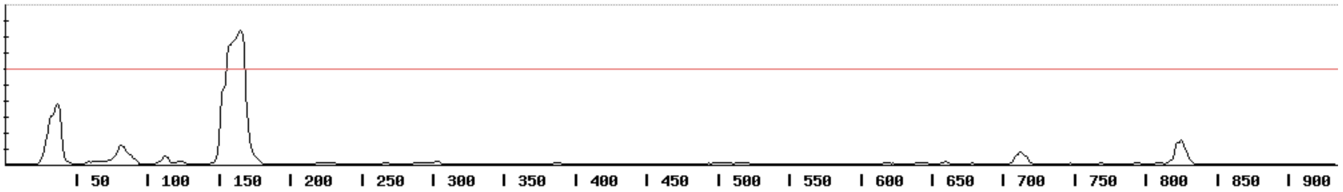

Predictions for AbSPL1

Viterbi Path

Viterbi Path

Posterior @ 0.6

211 - RRRLAGHNKRRRK - 223

Posterior @ 0.6

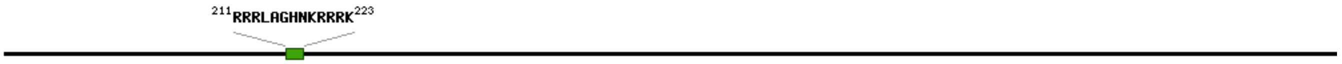

Posterior Graphic. Threshold Marked @ 0.6

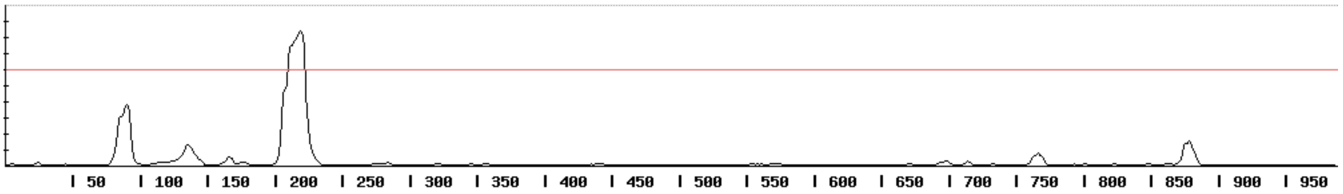

Predictions for ClSPL1

Viterbi Path

Viterbi Path

Posterior @ 0.6	211 - RRRLAGHNKRRRK - 223
Posterior @ 0.6	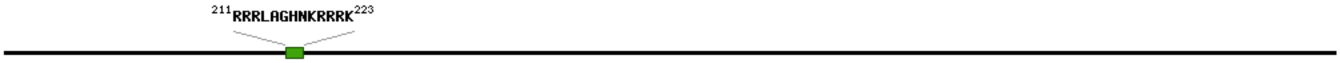
Posterior Graphic. Threshold Marked @ 0.6	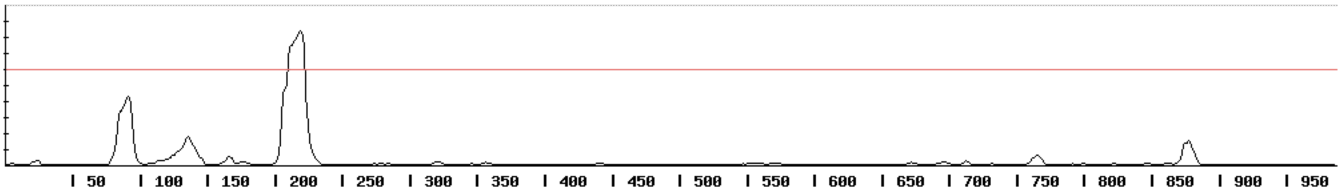

Predictions for CitSPL1

Viterbi Path	
Viterbi Path	
Posterior @ 0.6	211 - RRRLAGHNKRRRK - 223
Posterior @ 0.6	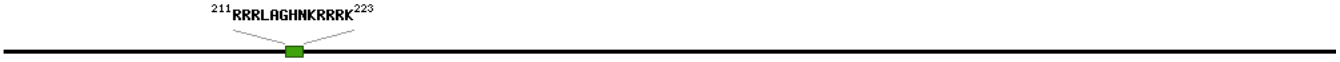
Posterior Graphic. Threshold Marked @ 0.6	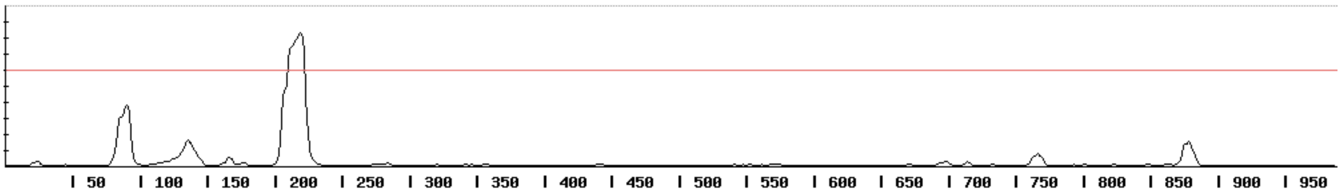

Predictions for PtSPL1

Viterbi Path	
Viterbi Path	
Posterior @ 0.6	211 - RRRLAGHNKRRRK - 223
Posterior @ 0.6	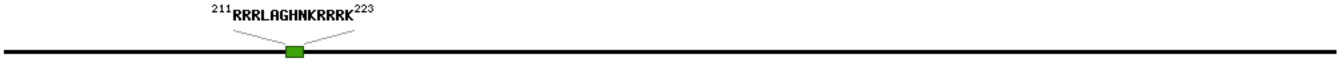
Posterior Graphic. Threshold Marked @ 0.6	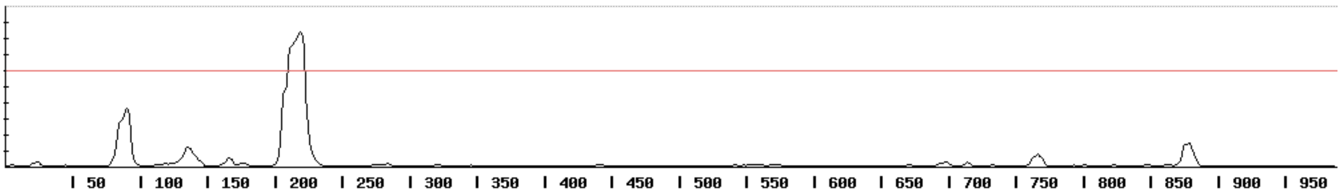

Predictions for FhSPL1

Viterbi Path

Viterbi Path

Posterior @ 0.6 | 211 - RRRLAGHNKRRRK - 223

Posterior @ 0.6

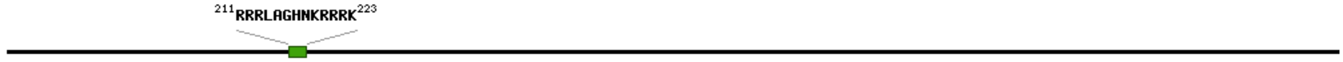

Posterior Graphic. Threshold Marked @ 0.6

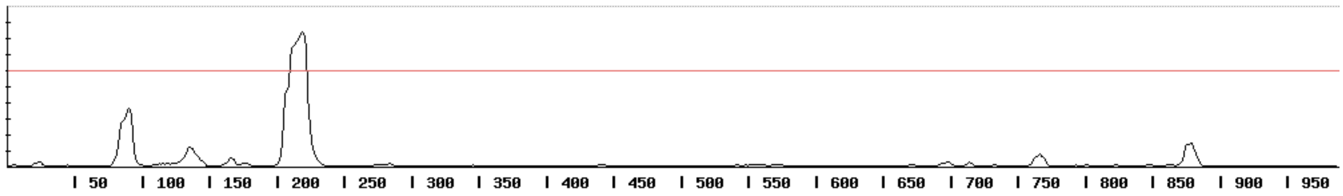

Predictions for CmsSPL1

Viterbi Path

Viterbi Path

Posterior @ 0.6 | 211 - RRRLAGHNKRRRK - 223

Posterior @ 0.6

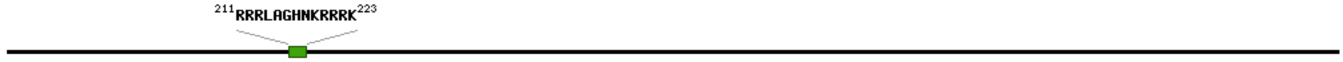

Posterior Graphic. Threshold Marked @ 0.6

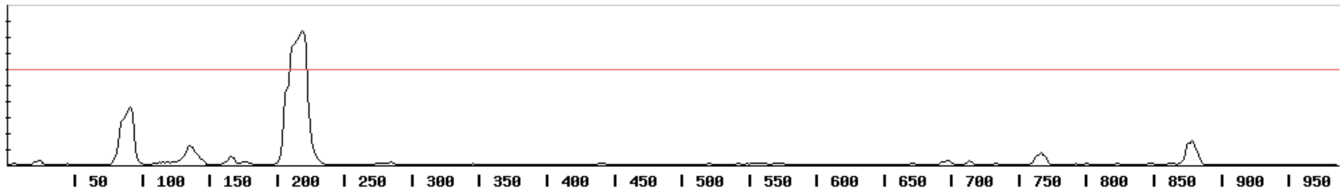

Predictions for CicSPL1

Viterbi Path

Viterbi Path

Posterior @ 0.6 | 211 - RRRLAGHNKRRRK - 223

Posterior @ 0.6

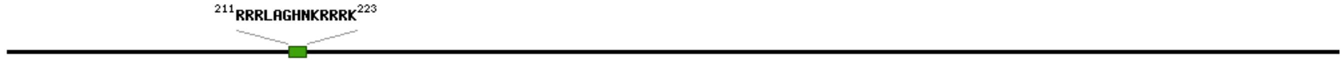

Posterior Graphic. Threshold

Marked @ 0.6

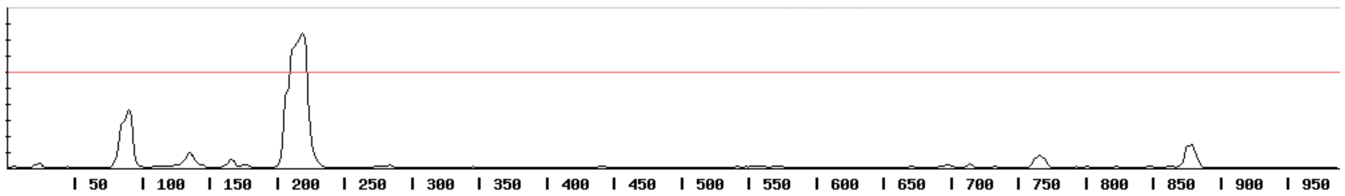

Predictions for CsSPL1

Viterbi Path

Viterbi Path

Posterior @ 0.6

211 - RRRLAGHNKRRRK - 223

Posterior @ 0.6

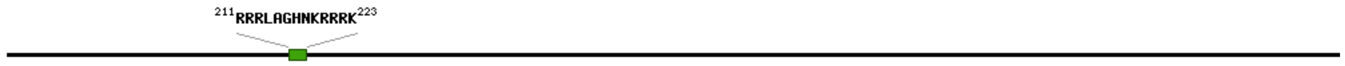

Posterior Graphic. Threshold Marked @ 0.6

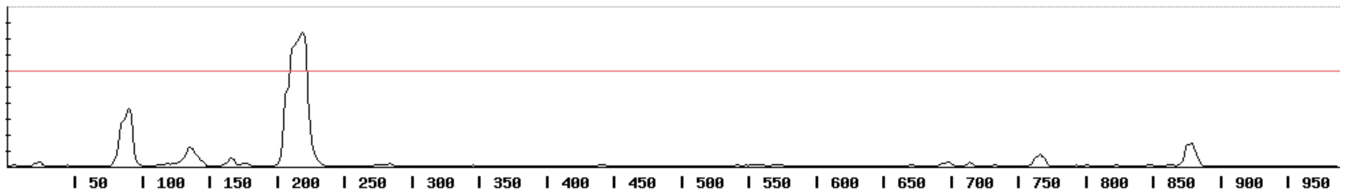

Predictions for CrSPL1

Viterbi Path

Viterbi Path

Posterior @ 0.6

211 - RRRLAGHNKRRRK - 223

Posterior @ 0.6

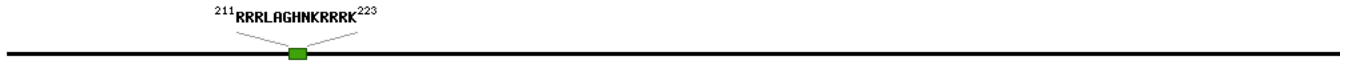

Posterior Graphic. Threshold Marked @ 0.6

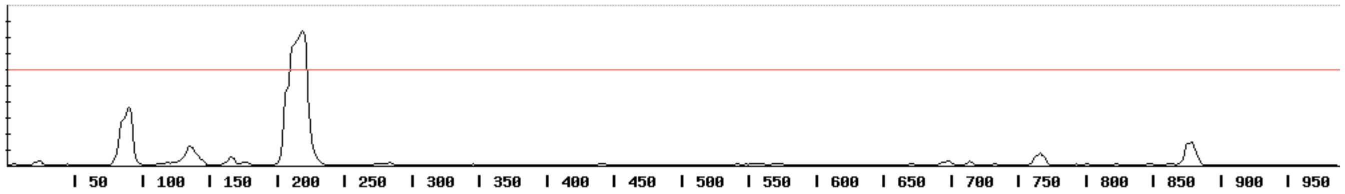

Predictions for ChSPL1

Viterbi Path

Viterbi Path

Posterior @ 0.6 — 211 - RRRLAGHNKRRRK - 223

Posterior @ 0.6

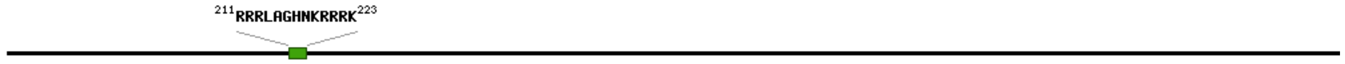

Posterior Graphic. Threshold Marked @ 0.6

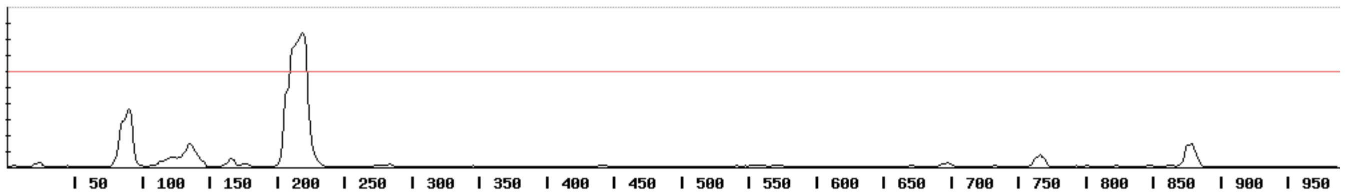

Predictions for CmjSPL1

Viterbi Path

Viterbi Path

Posterior @ 0.6 — 211 - RRRLAGHNKRRRK - 223

Posterior @ 0.6

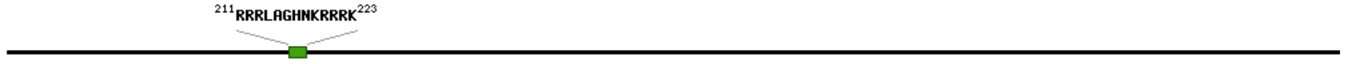

Posterior Graphic. Threshold Marked @ 0.6

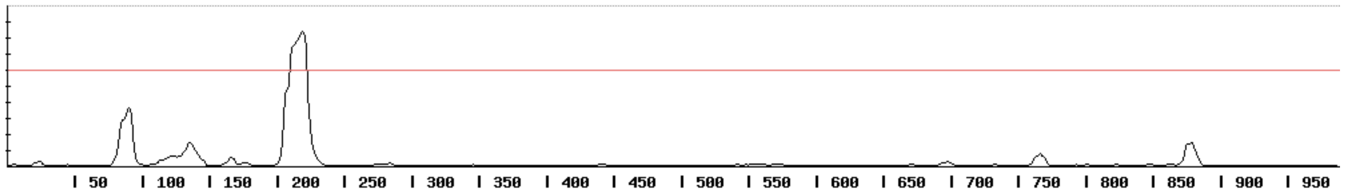

Predictions for CzpSPL1

Viterbi Path

Viterbi Path

Posterior @ 0.6 — 211 - RRRLAGHNKRRRK - 223

Posterior @ 0.6

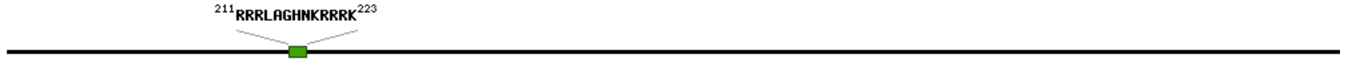

Posterior Graphic. Threshold Marked @ 0.6

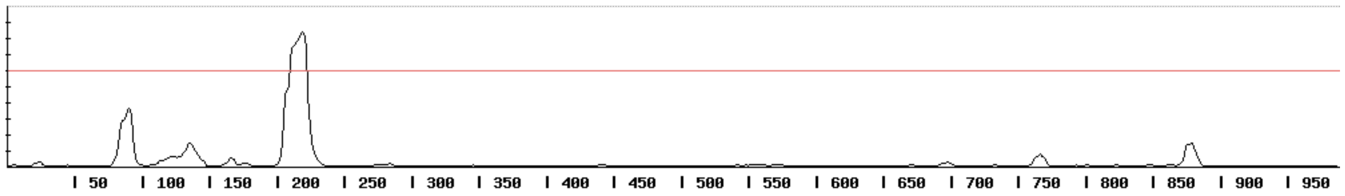

Predictions for CmSPL1

Viterbi Path

Viterbi Path

Posterior @ 0.6

211 - RRRLAGHNKRRRK - 223

Posterior @ 0.6

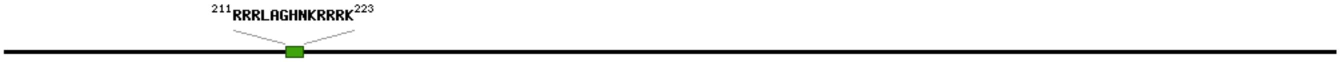

Posterior Graphic. Threshold Marked @ 0.6

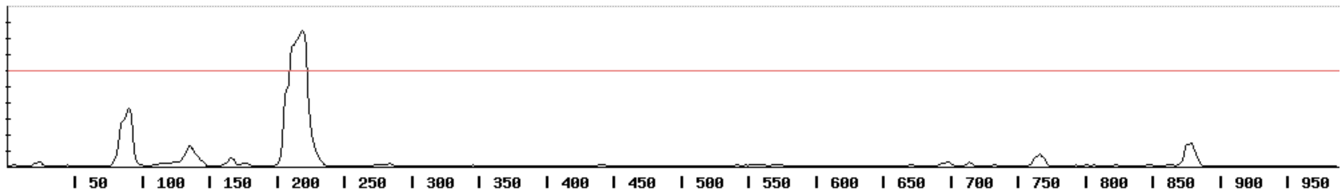

Predictions for AtSPL12

Viterbi Path

Viterbi Path

Posterior @ 0.6

66 - RRIEMEKKKKRR - 77

188 - RRRLAGHNKRRRK - 200

Posterior @ 0.6

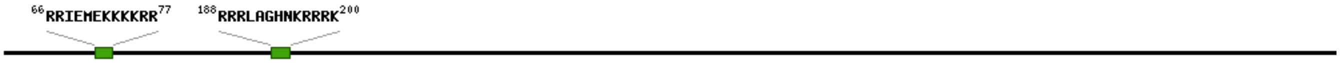

Posterior Graphic. Threshold Marked @ 0.6

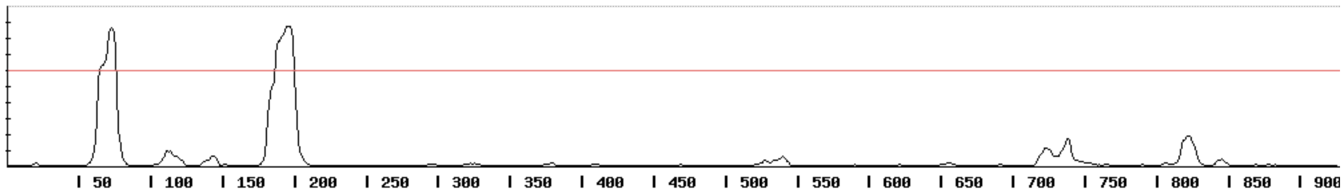

Predictions for AmSPL12

Viterbi Path

Viterbi Path

Posterior @ 0.6

211 - RRRLAGHNKRRRK - 223

Posterior @ 0.6

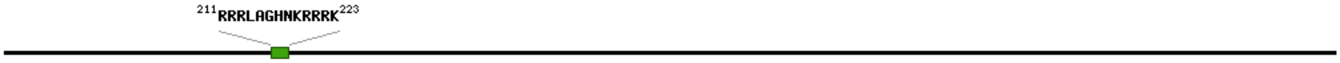

Posterior Graphic.

Threshold Marked @ 0.6

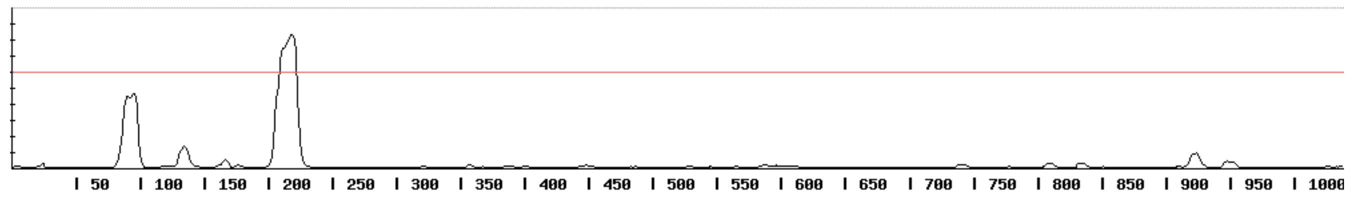

Predictions for MuSPL12

Viterbi Path	
Viterbi Path	
Posterior @ 0.6	45 - RRRLAGHNKRRRK - 57
Posterior @ 0.6	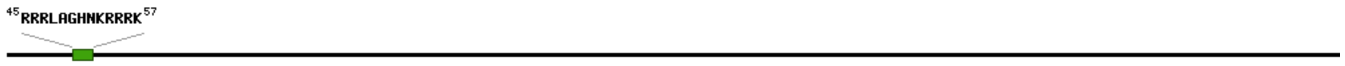
Posterior Graphic. Threshold Marked @ 0.6	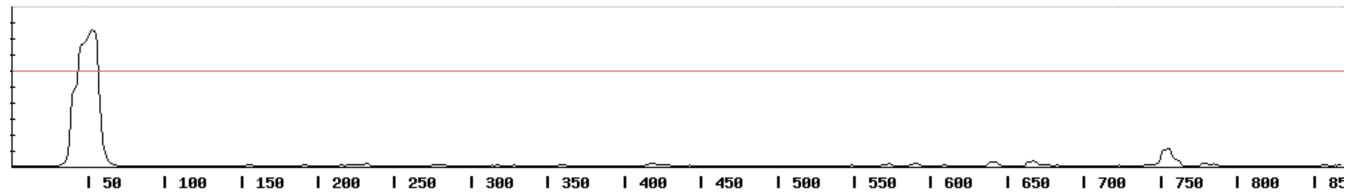

Predictions for AbSPL12

Viterbi Path	
Viterbi Path	
Posterior @ 0.6	
Posterior @ 0.6	
Posterior Graphic. Threshold Marked @ 0.6	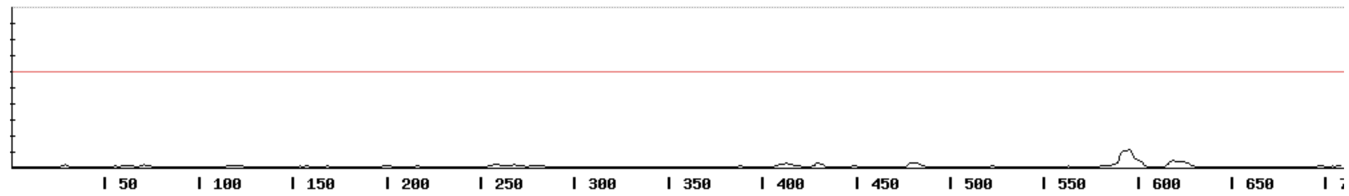

Predictions for ClSPL12

Viterbi Path	
Viterbi Path	

Posterior @ 0.6	211 - RRRLAGHNKRRRK - 223
Posterior @ 0.6	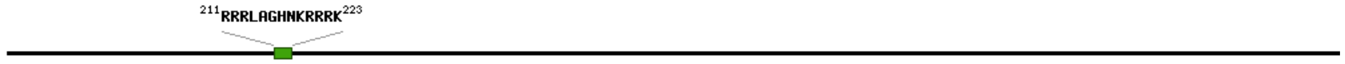
Posterior Graphic. Threshold Marked @ 0.6	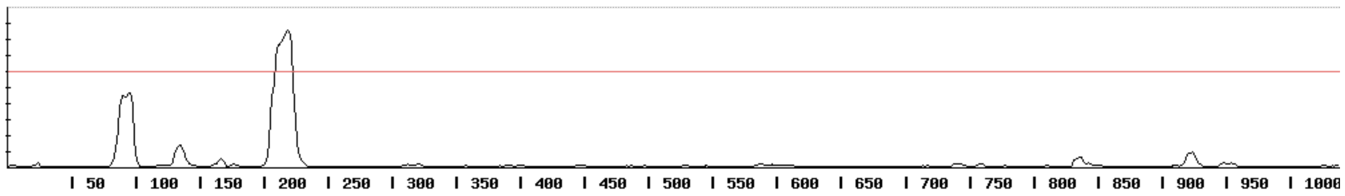

Predictions for CitSPL12

Viterbi Path	
Viterbi Path	
Posterior @ 0.6	212 - RRRLAGHNKRRRK - 224
Posterior @ 0.6	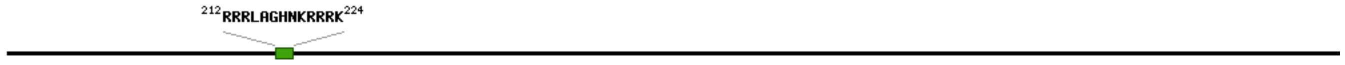
Posterior Graphic. Threshold Marked @ 0.6	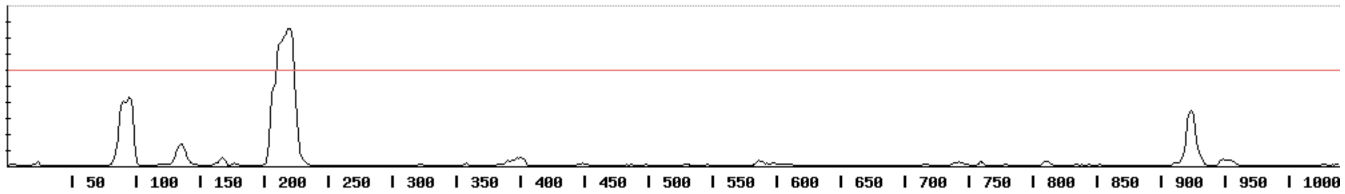

Predictions for PtSPL12

Viterbi Path	
Viterbi Path	
Posterior @ 0.6	211 - RRRLAGHNKRRRK - 223
Posterior @ 0.6	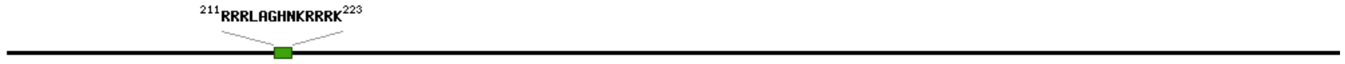
Posterior Graphic. Threshold Marked @ 0.6	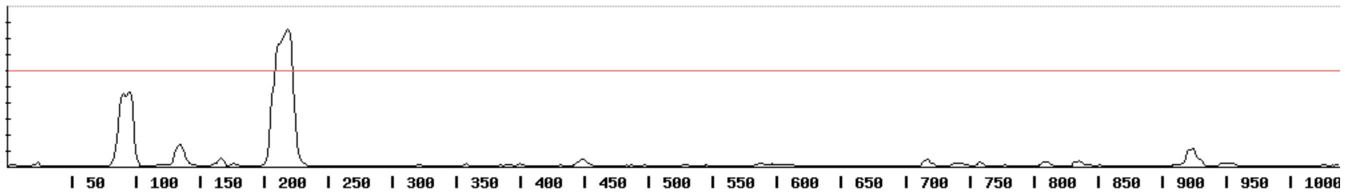

Predictions for FhSPL12

Viterbi Path

Viterbi Path

Posterior @ 0.6

211 - RRRLAGHNKRRRK - 223

Posterior @ 0.6

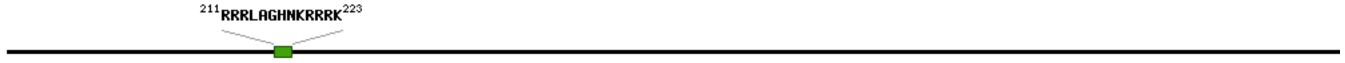

Posterior Graphic. Threshold Marked @ 0.6

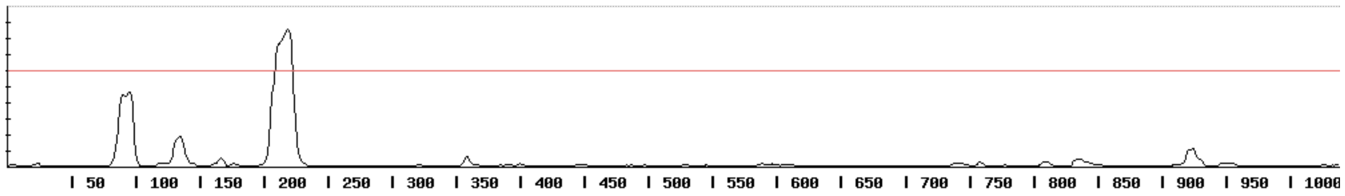

Predictions for CmsSPL12

Viterbi Path

Viterbi Path

Posterior @ 0.6

211 - RRRLAGHNKRRRK - 223

Posterior @ 0.6

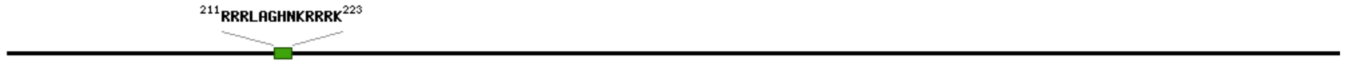

Posterior Graphic. Threshold Marked @ 0.6

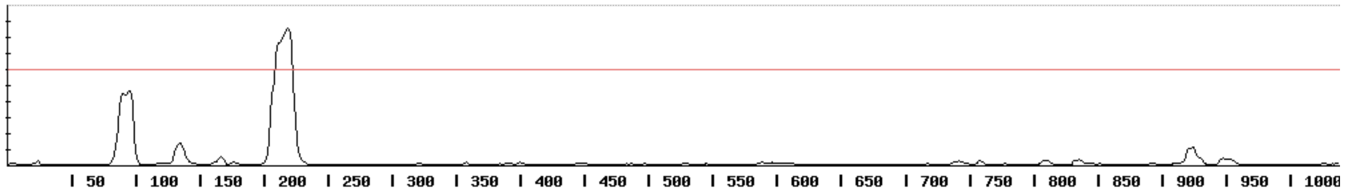

Predictions for CicSPL12

Viterbi Path

Viterbi Path

Posterior @ 0.6

211 - RRRLAGHNKRRRK - 223

Posterior @ 0.6

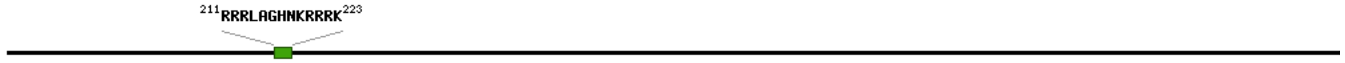

Posterior Graphic. Threshold

Marked @ 0.6

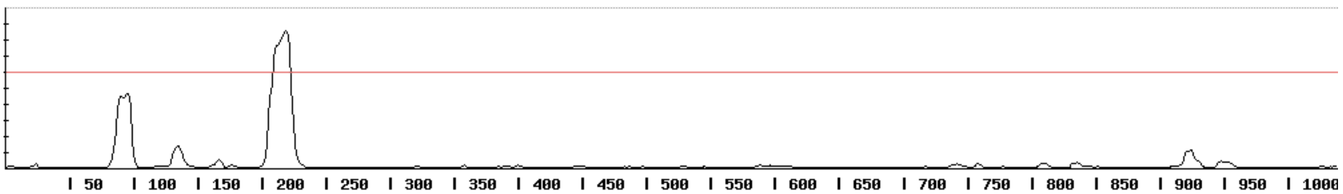

Predictions for CsSPL12

Viterbi Path

Viterbi Path

Posterior @ 0.6

45 - RRRLAGHNKRRRK - 57

Posterior @ 0.6

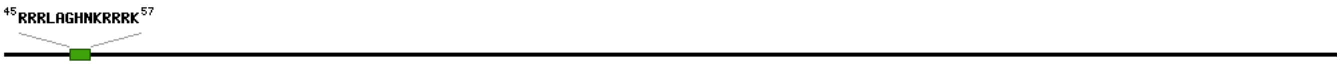

Posterior Graphic. Threshold Marked @ 0.6

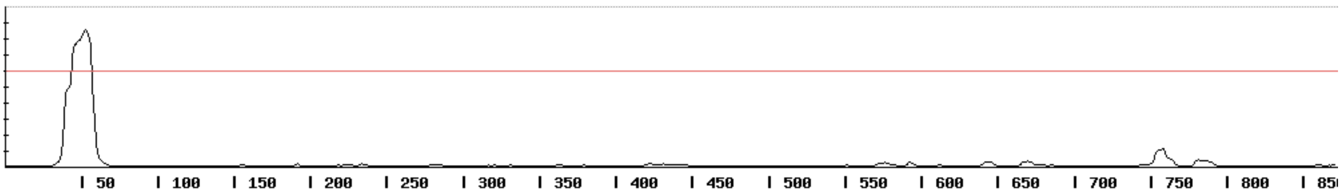

Predictions for CrSPL12

Viterbi Path

Viterbi Path

Posterior @ 0.6

211 - RRRLAGHNKRRRK - 223

Posterior @ 0.6

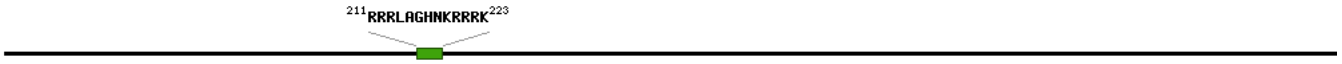

Posterior Graphic. Threshold Marked @ 0.6

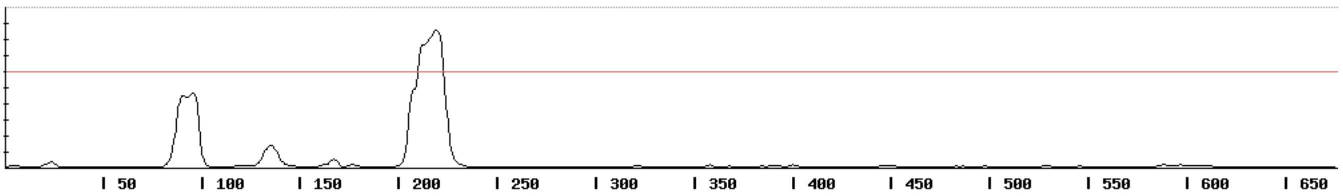

Predictions for ChSPL12

Viterbi Path

Viterbi Path

Posterior @ 0.6	211 - RRRLAGHNKRRRK - 223
Posterior @ 0.6	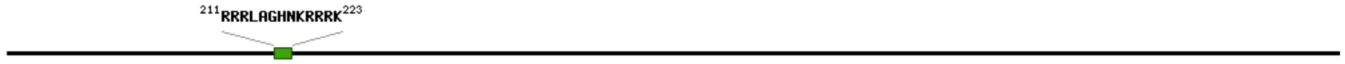
Posterior Graphic. Threshold Marked @ 0.6	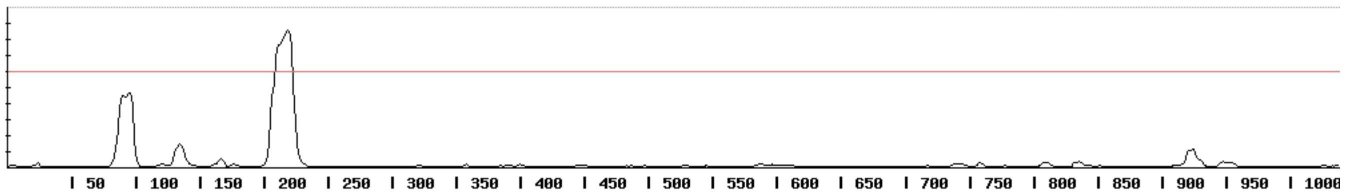

Predictions for CmjSPL12

Viterbi Path	
Viterbi Path	
Posterior @ 0.6	211 - RRRLAGHNKRRRK - 223
Posterior @ 0.6	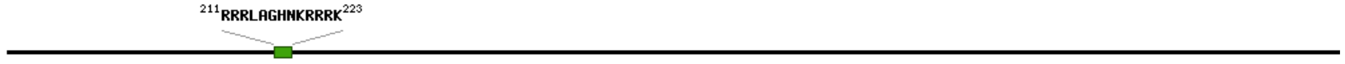
Posterior Graphic. Threshold Marked @ 0.6	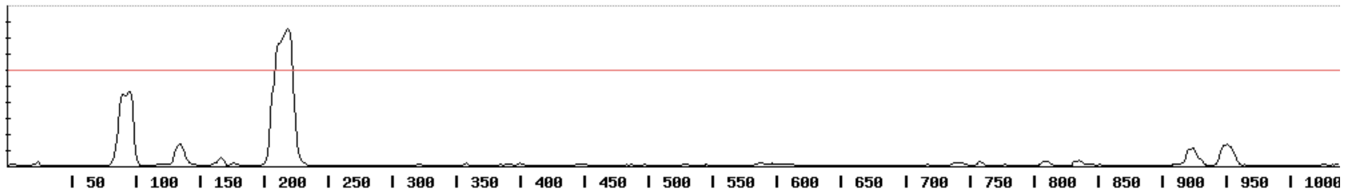

Predictions for CzpSPL12

Viterbi Path	
Viterbi Path	
Posterior @ 0.6	211 - RRRLAGHNKRRRK - 223
Posterior @ 0.6	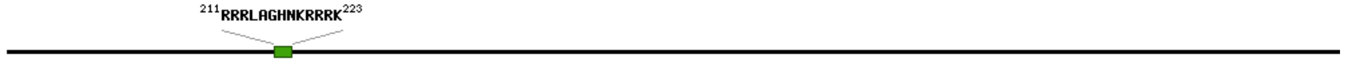
Posterior Graphic. Threshold Marked @ 0.6	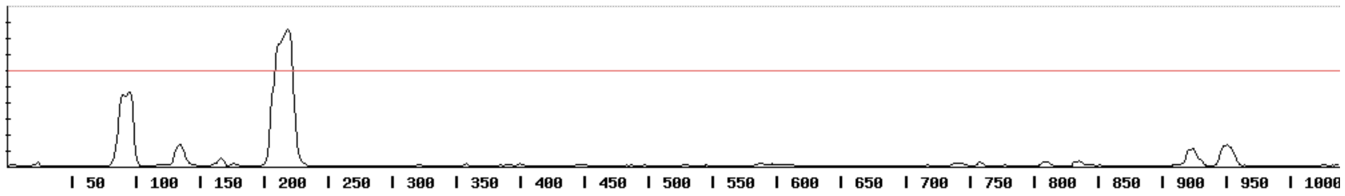

Predictions for CmSPL12

Viterbi Path

Viterbi Path

Posterior @ 0.6: 211 - RRRLAGHNKRRRK - 223

Posterior @ 0.6

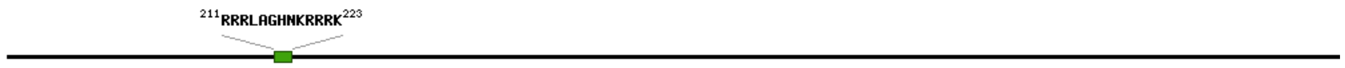

Posterior Graphic. Threshold Marked @ 0.6

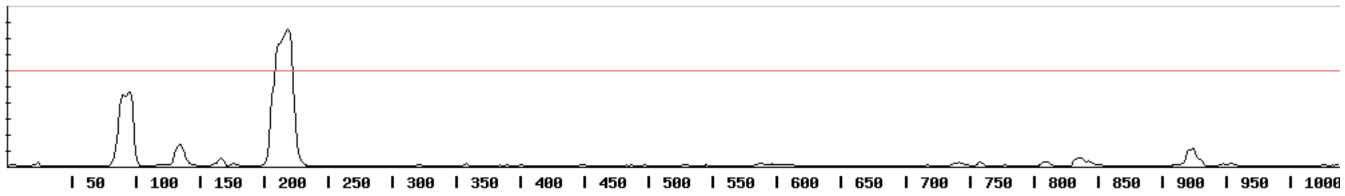

Predictions for AtSPL16

Viterbi Path

Viterbi Path

Posterior @ 0.6: 182 - NRRRR - 186

Posterior @ 0.6

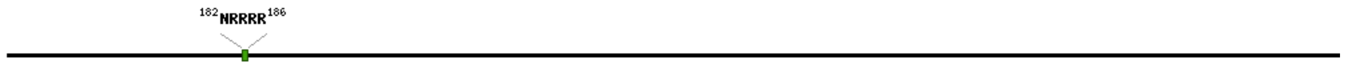

Posterior Graphic. Threshold Marked @ 0.6

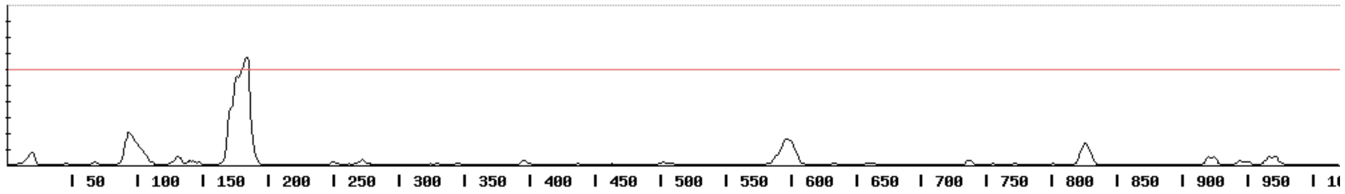

Predictions for AtSPL14

Viterbi Path

Viterbi Path

Posterior @ 0.6: 181 - RRRLAGHNRRRRK - 193

Posterior @ 0.6

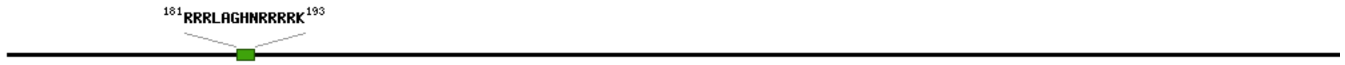

Posterior Graphic. Threshold

Marked @ 0.6

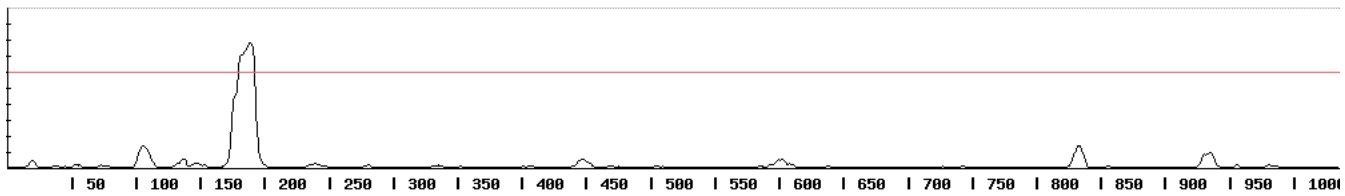

Predictions for AmSPL14

Viterbi Path

Viterbi Path

Posterior @ 0.6

207 - RRRLAGHNRRRRK - 219

Posterior @ 0.6

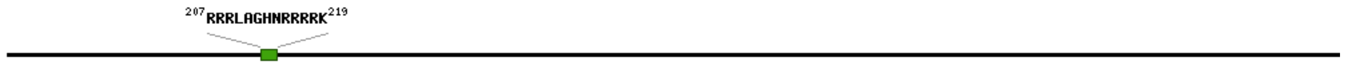

Posterior Graphic. Threshold Marked @ 0.6

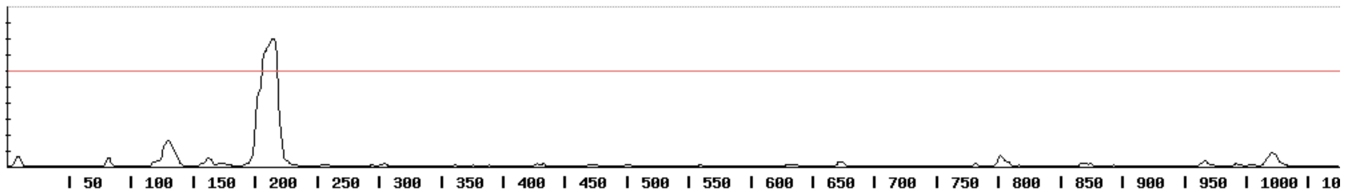

Predictions for MuSPL14

Viterbi Path

Viterbi Path

Posterior @ 0.6

234 - RRRLAGHNRRRRK - 246

Posterior @ 0.6

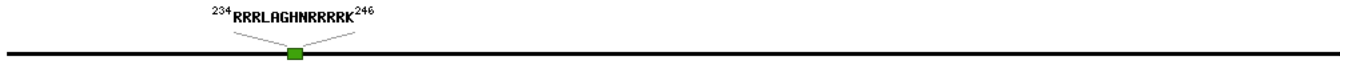

Posterior Graphic. Threshold Marked @ 0.6

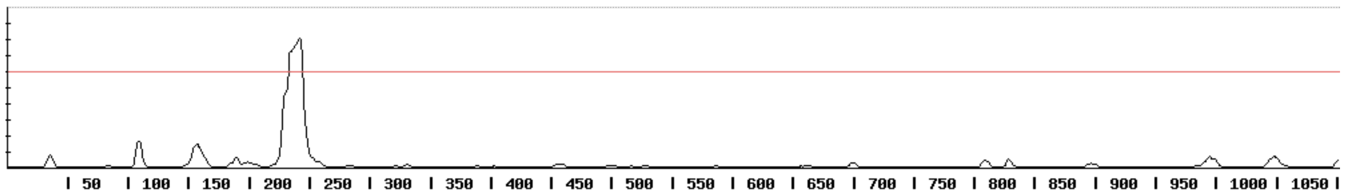

Predictions for AbSPL14

Viterbi Path

Viterbi Path

Posterior @ 0.6 — 207 - RRRLAGHNRRRRK - 219

Posterior @ 0.6

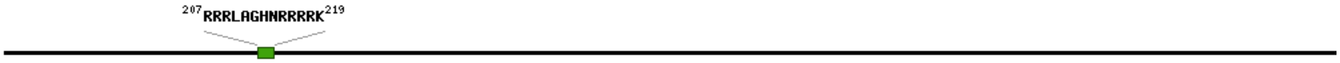

Posterior Graphic. Threshold Marked @ 0.6

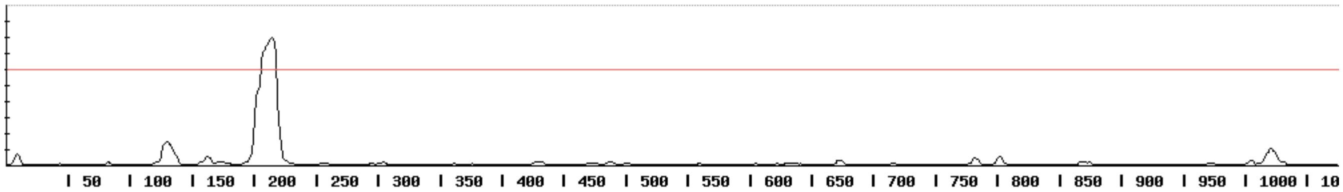

Predictions for ClSPL14

Viterbi Path

Viterbi Path

Posterior @ 0.6 — 208 - RRRLAGHNRRRRK - 220

Posterior @ 0.6

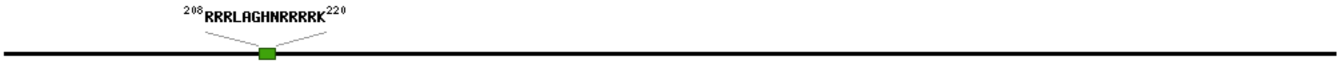

Posterior Graphic. Threshold Marked @ 0.6

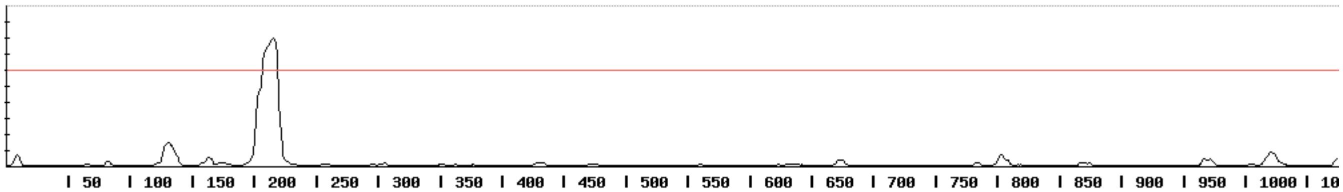

Predictions for CitSPL14

Viterbi Path

Viterbi Path

Posterior @ 0.6 — 234 - RRRLAGHNRRRRK - 246

Posterior @ 0.6

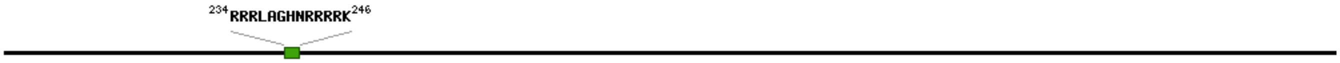

Posterior Graphic. Threshold Marked @ 0.6

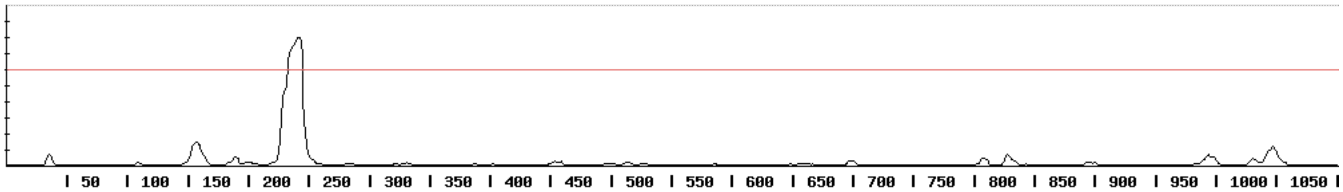

Predictions for PtSPL14

Viterbi Path

Viterbi Path

Posterior @ 0.6

234 - RRRLAGHNRRRRK - 246

Posterior @ 0.6

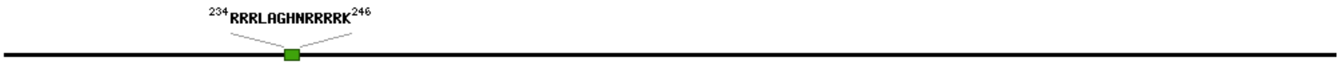

Posterior Graphic. Threshold Marked @ 0.6

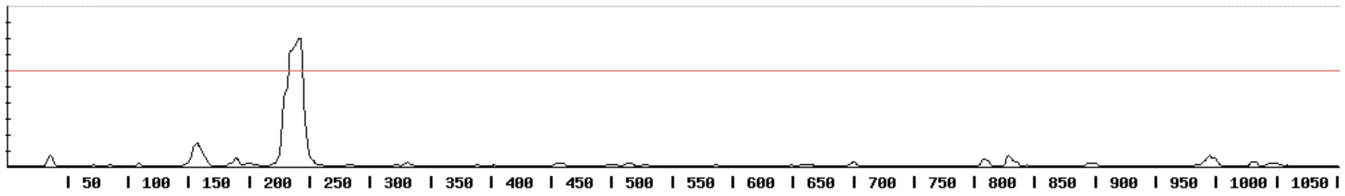

Predictions for FhSPL14

Viterbi Path

Viterbi Path

Posterior @ 0.6

234 - RRRLAGHNRRRRK - 246

Posterior @ 0.6

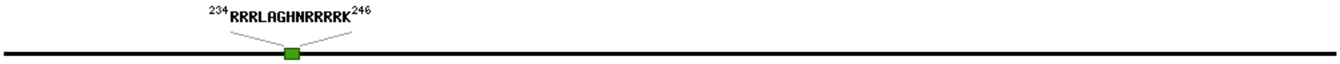

Posterior Graphic. Threshold Marked @ 0.6

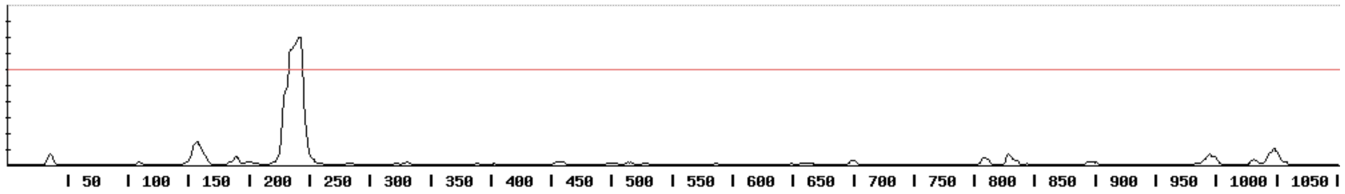

Predictions for CmsSPL14

Viterbi Path

Viterbi Path

Posterior @ 0.6

234 - RRRLAGHNRRRRK - 246

Posterior @ 0.6

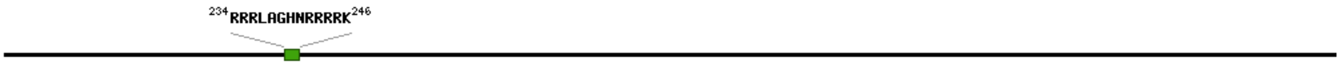

Posterior Graphic. Threshold

Marked
@ 0.6

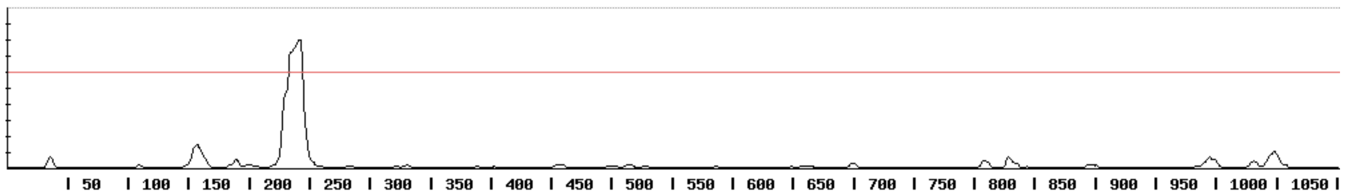

Predictions for CicSPL14

Viterbi Path

Viterbi Path

Posterior @ 0.6

234 - RRRLAGHNRRRRK - 246

Posterior @ 0.6

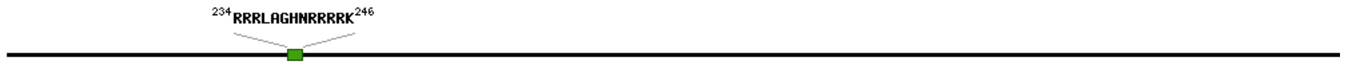

Posterior Graphic. Threshold Marked @ 0.6

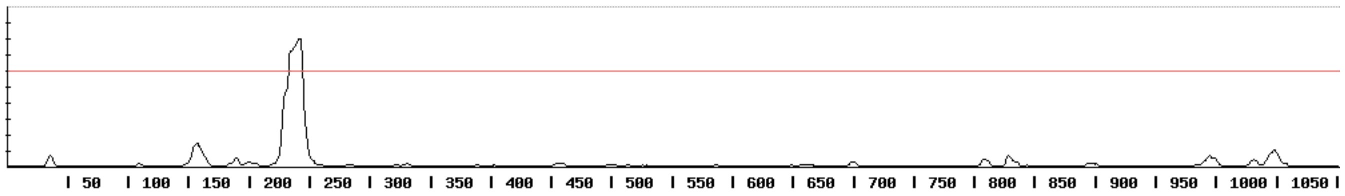

Predictions for CsSPL14

Viterbi Path

Viterbi Path

Posterior @ 0.6

207 - RRRLAGHNRRRRK - 219

Posterior @ 0.6

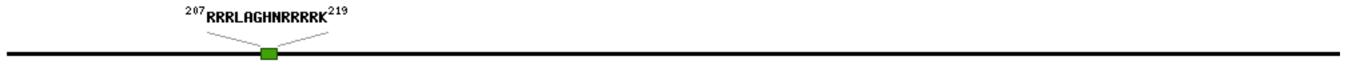

Posterior Graphic. Threshold Marked @ 0.6

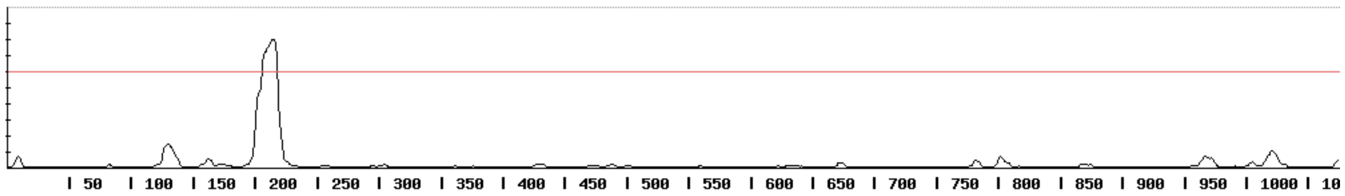

Predictions for CrSPL14

Viterbi Path

Viterbi Path

Posterior @ 0.6	234 - RRRLAGHNRRRRK - 246
Posterior @ 0.6	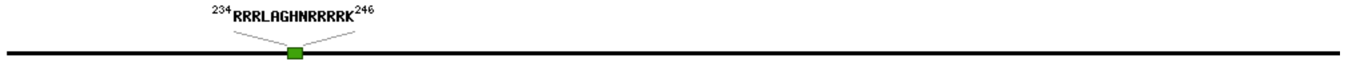
Posterior Graphic. Threshold Marked @ 0.6	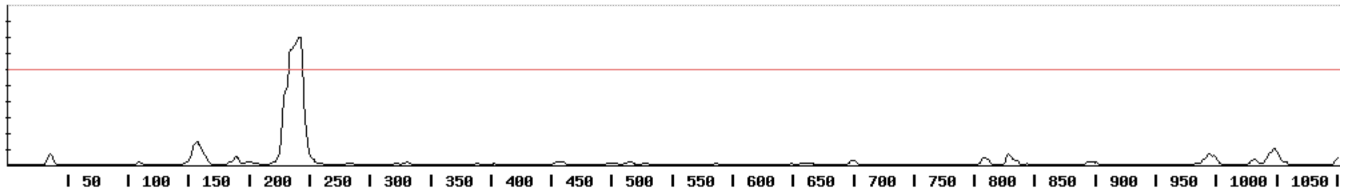

Predictions for ChSPL14

Viterbi Path	
Viterbi Path	
Posterior @ 0.6	234 - RRRLAGHNRRRRK - 246
Posterior @ 0.6	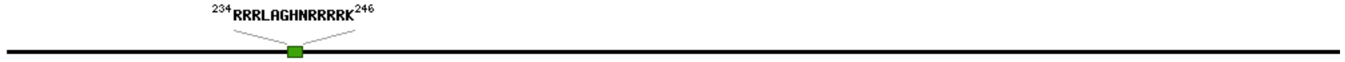
Posterior Graphic. Threshold Marked @ 0.6	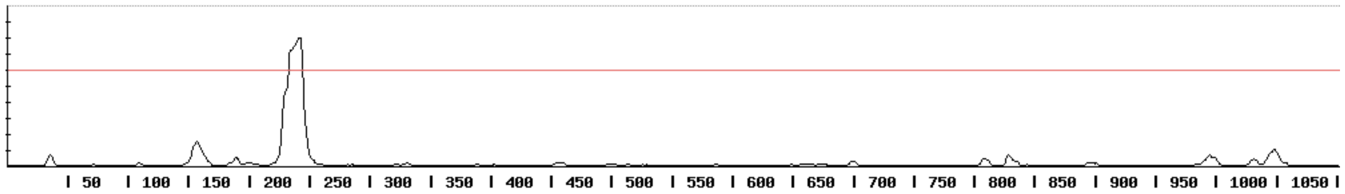

Predictions for CmjSPL14

Viterbi Path	
Viterbi Path	
Posterior @ 0.6	234 - RRRLAGHNRRRRK - 246
Posterior @ 0.6	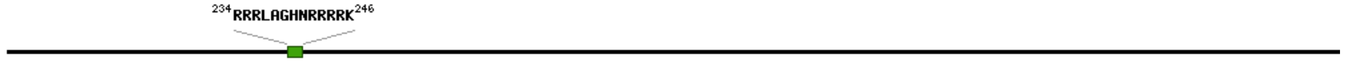
Posterior Graphic. Threshold Marked @ 0.6	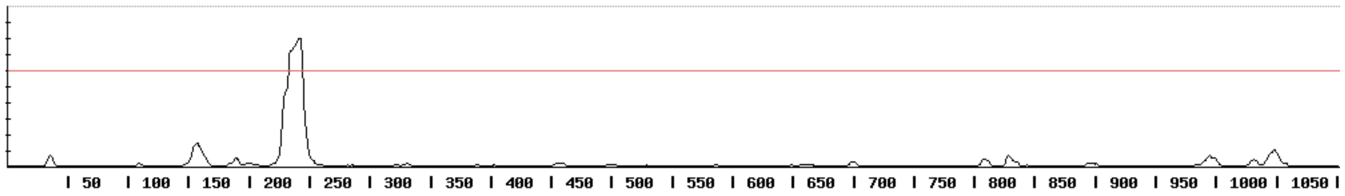

Predictions for CzpSPL14

Viterbi Path	
Viterbi Path	
Posterior @ 0.6	207 - RRRLAGHNRRRRK - 219
Posterior @ 0.6	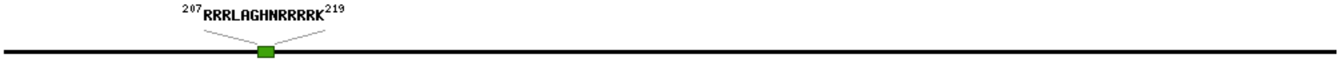
Posterior Graphic. Threshold Marked @ 0.6	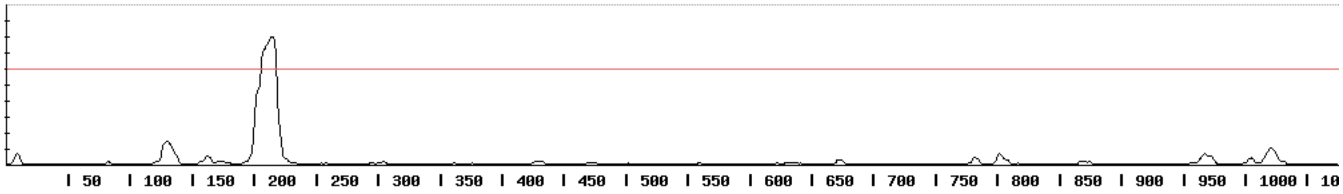

Predictions for CmSPL14

Viterbi Path	
Viterbi Path	
Posterior @ 0.6	234 - RRRLAGHNRRRRK - 246
Posterior @ 0.6	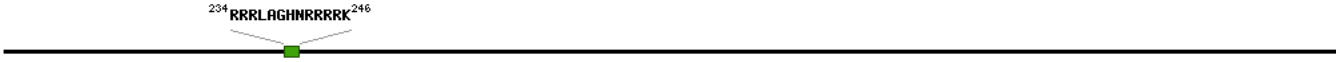
Posterior Graphic. Threshold Marked @ 0.6	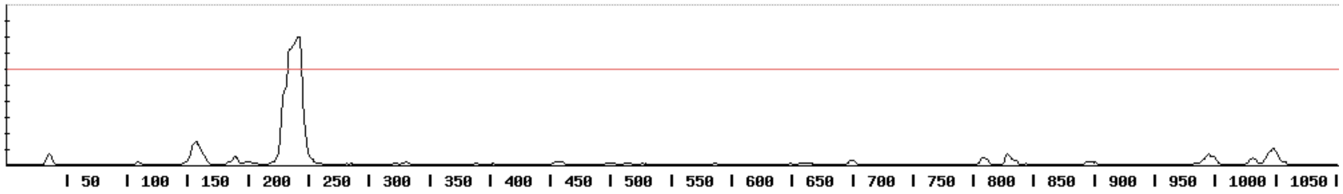

Predictions for AtSPL13

Viterbi Path	
Viterbi Path	
Posterior @ 0.6	162 - RKRLDGHNRRRRKPQ - 176
Posterior @ 0.6	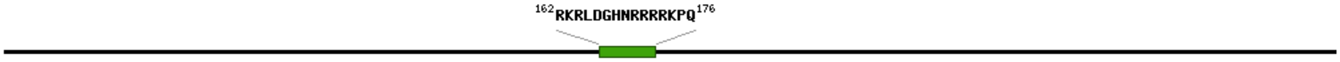
Posterior Graphic. Threshold	

Marked @ 0.6

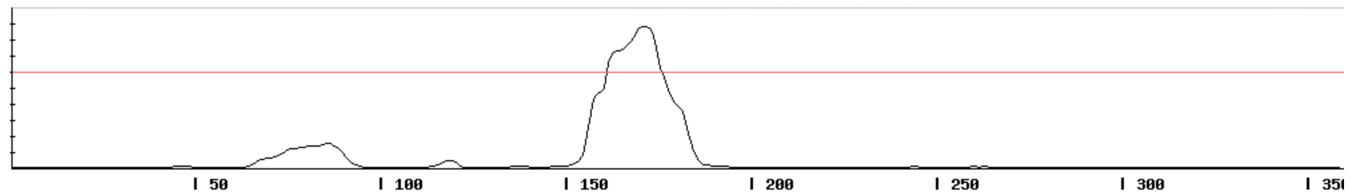

Predictions for AmSPL13A

Viterbi Path

Viterbi Path

Posterior @ 0.6 — 38 - RKRLDGHNRRRRK - 50

Posterior @ 0.6

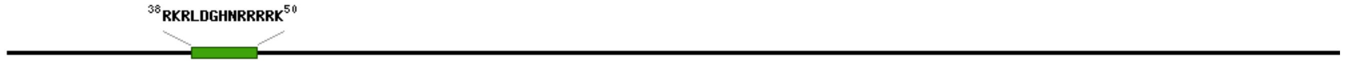

Posterior Graphic. Threshold Marked @ 0.6

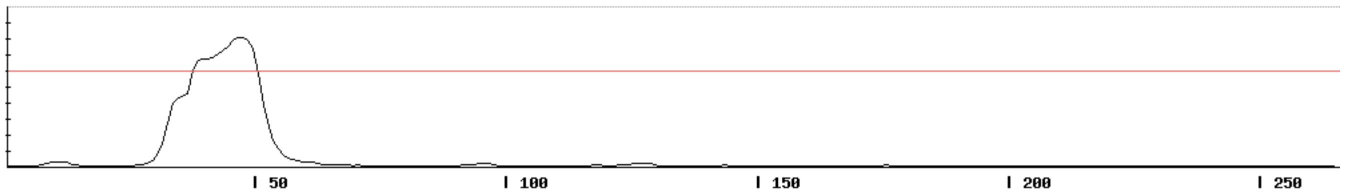

Predictions for AmSPL13B

Viterbi Path

Viterbi Path

Posterior @ 0.6 — 149 - RKRLDGHNRRRRKP - 162

Posterior @ 0.6

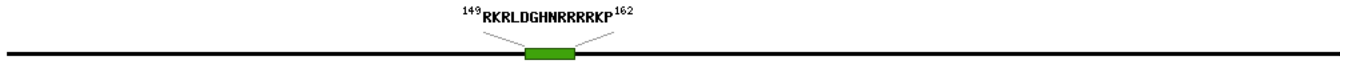

Posterior Graphic. Threshold Marked @ 0.6

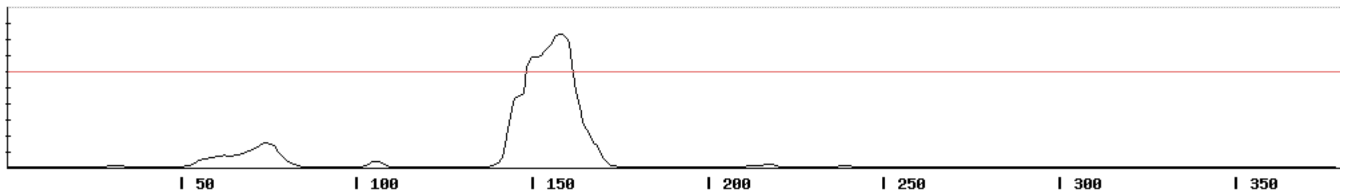

Predictions for MuSPL13A

Viterbi Path

Viterbi Path

Posterior @ 0.6	546 - RKRLDGHNRRRRK - 558
Posterior @ 0.6	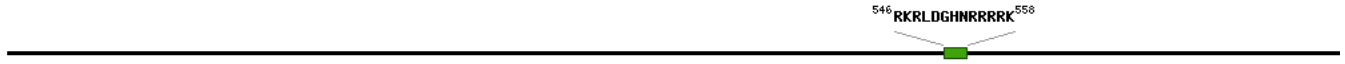
Posterior Graphic. Threshold Marked @ 0.6	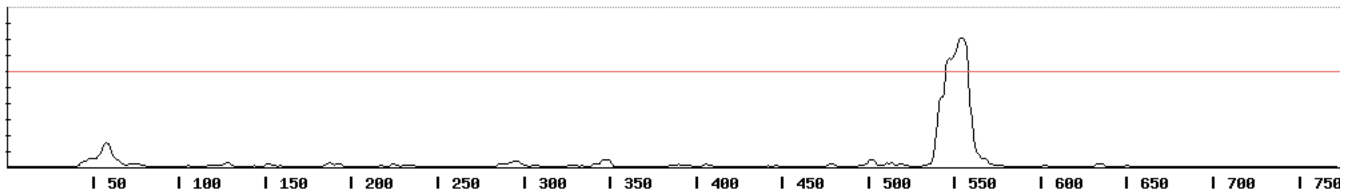

Predictions for MuSPL13B

Viterbi Path	
Viterbi Path	
Posterior @ 0.6	149 - RKRLDGHNRRRRKP - 162
Posterior @ 0.6	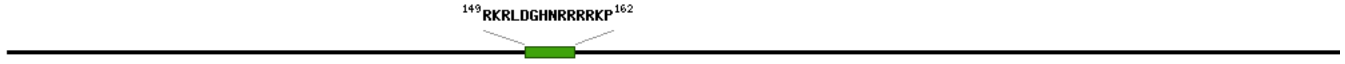
Posterior Graphic. Threshold Marked @ 0.6	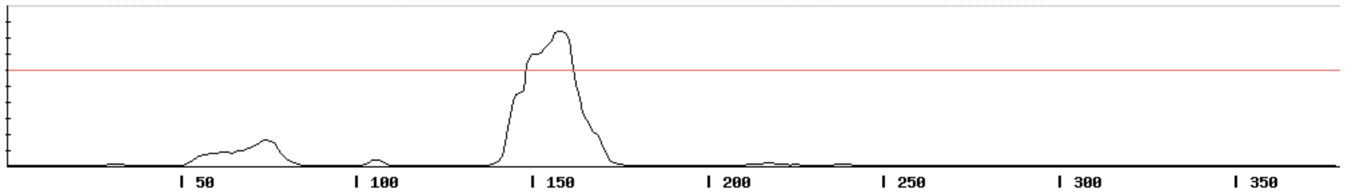

Predictions for AbSPL13A

Viterbi Path	
Viterbi Path	
Posterior @ 0.6	85 - RKRLDGHNRRRRK - 97
Posterior @ 0.6	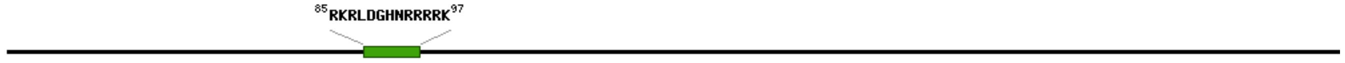
Posterior Graphic. Threshold Marked @ 0.6	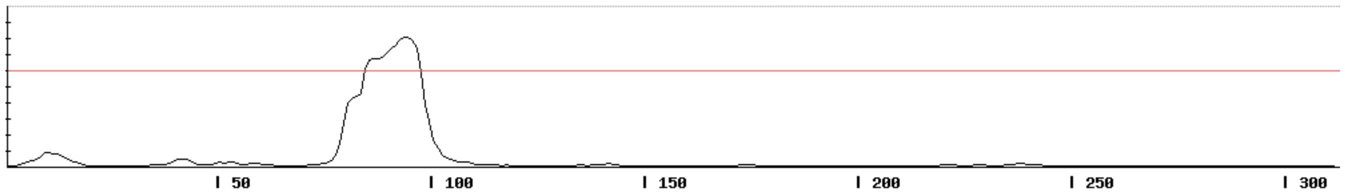

Predictions for AbSPL13B

Viterbi Path	
Viterbi Path	
Posterior @ 0.6	149 - RKRLDGHNRRRRKP - 162
Posterior @ 0.6	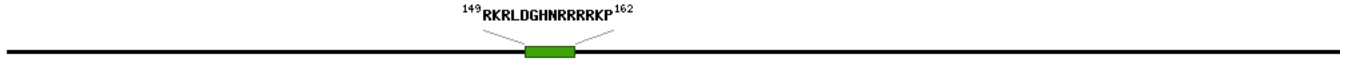
Posterior Graphic. Threshold Marked @ 0.6	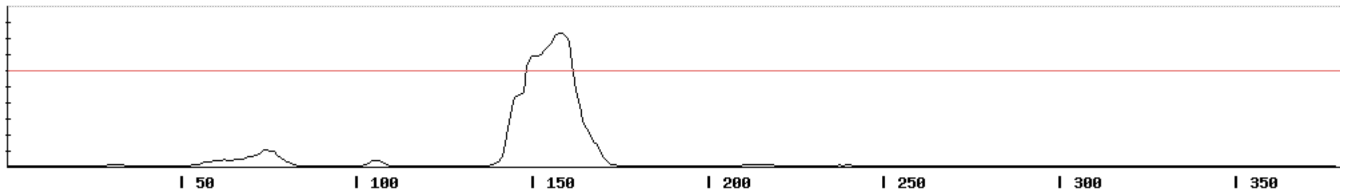

Predictions for ClSPL13A

Viterbi Path	
Viterbi Path	
Posterior @ 0.6	85 - RKRLDGHNRRRRK - 97
Posterior @ 0.6	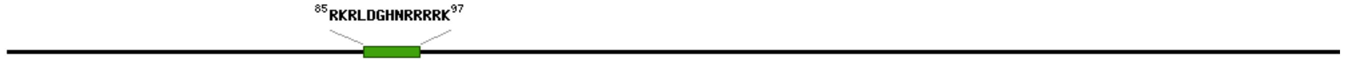
Posterior Graphic. Threshold Marked @ 0.6	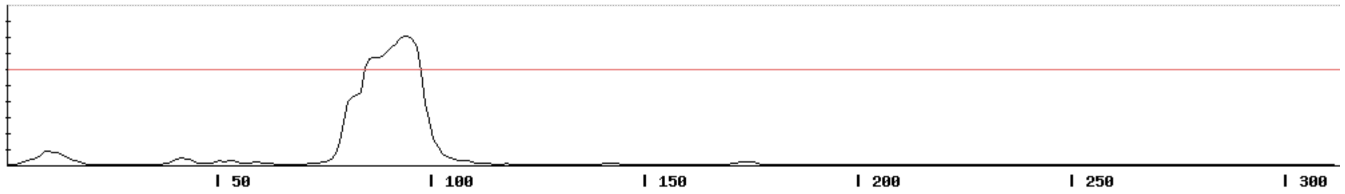

Predictions for ClSPL13B

Viterbi Path	
Viterbi Path	
Posterior @ 0.6	149 - RKRLDGHNRRRRKP - 162
Posterior @ 0.6	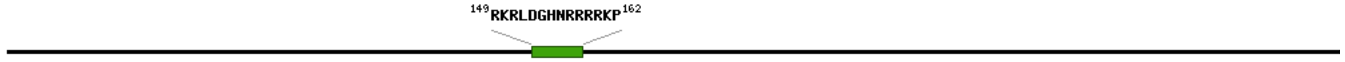
Posterior Graphic. Threshold	

Marked
@ 0.6

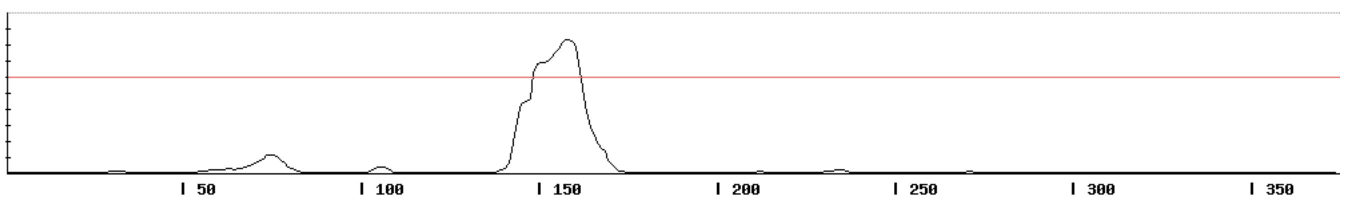

Predictions for CitSPL13A

Viterbi Path

Viterbi Path

Posterior @ 0.6 — 85 - RKRLDGHNRRRRK - 97

Posterior @ 0.6

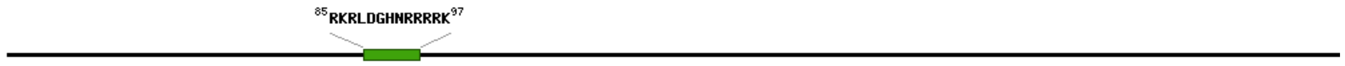

Posterior Graphic. Threshold Marked @ 0.6

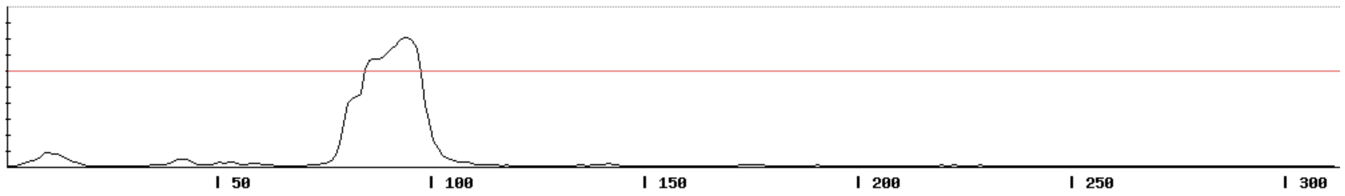

Predictions for CitSPL13B

Viterbi Path

Viterbi Path

Posterior @ 0.6 — 149 - RKRLDGHNRRRRK - 161

Posterior @ 0.6

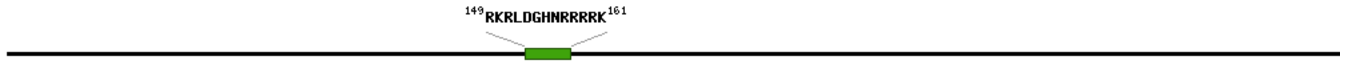

Posterior Graphic. Threshold Marked @ 0.6

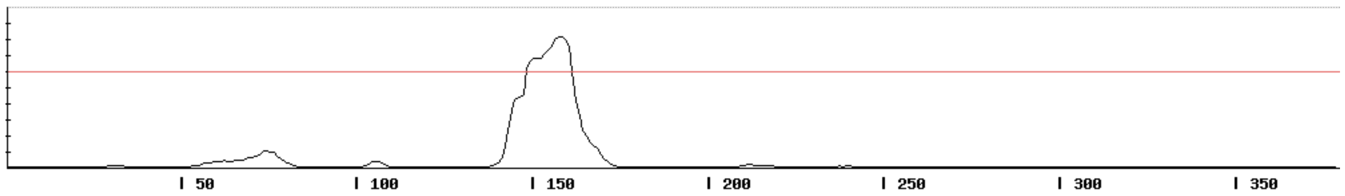

Predictions for PtSPL13A

Viterbi Path

Viterbi Path

Posterior @ 0.6 | 85 - RKRLDGHNRRRRK - 97

Posterior @ 0.6

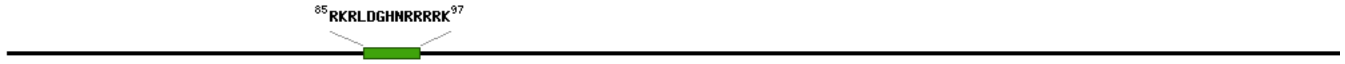

Posterior Graphic. Threshold Marked @ 0.6

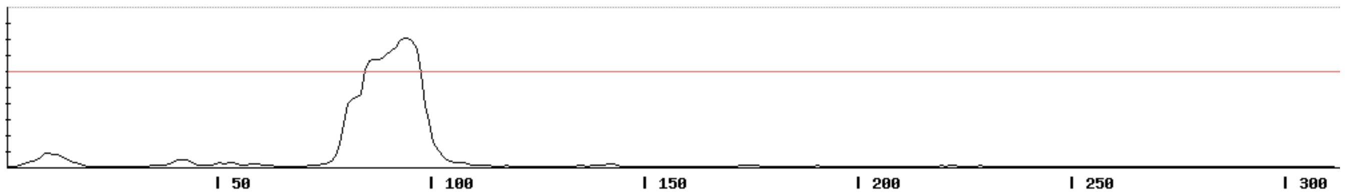

Predictions for PtSPL13B

Viterbi Path

Viterbi Path

Posterior @ 0.6 | 149 - RKRLDGHNRRRRKP - 162

Posterior @ 0.6

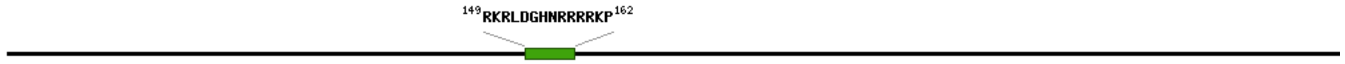

Posterior Graphic. Threshold Marked @ 0.6

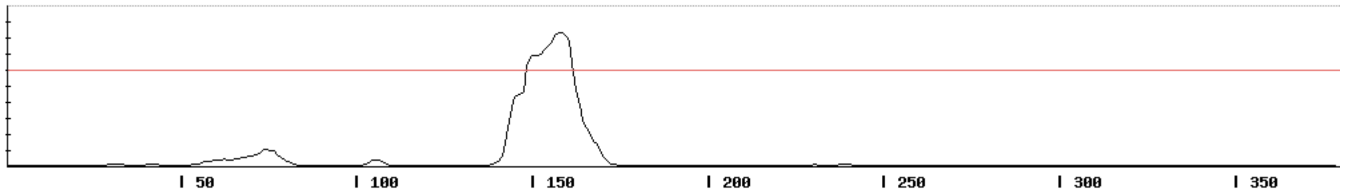

Predictions for FhSPL13B

Viterbi Path

Viterbi Path

Posterior @ 0.6 | 149 - RKRLDGHNRRRRKP - 162

Posterior @ 0.6

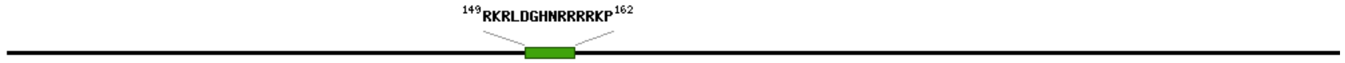

Posterior Graphic. Threshold Marked @ 0.6

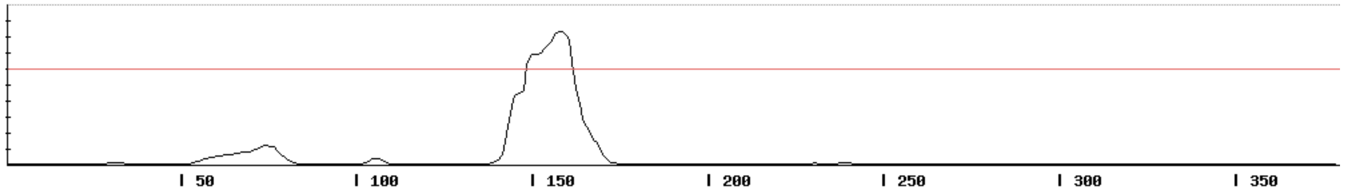

Predictions for FhSPL13A

Viterbi Path

Viterbi Path

Posterior @ 0.6

85 - RKRLDGHNRRRRK - 97

Posterior @ 0.6

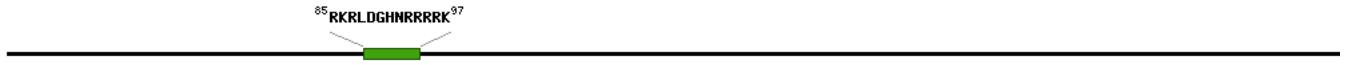

Posterior Graphic. Threshold Marked @ 0.6

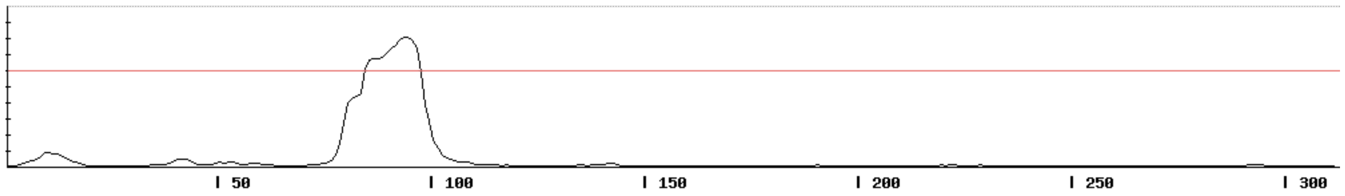

Predictions for CmsSPL13A

Viterbi Path

Viterbi Path

Posterior @ 0.6

74 - RKRLDGHNRRRRK - 86

Posterior @ 0.6

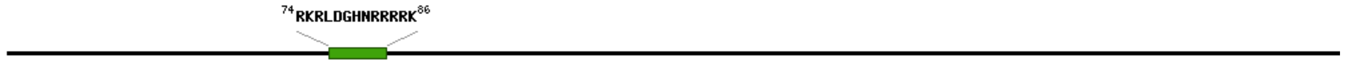

Posterior Graphic. Threshold Marked @ 0.6

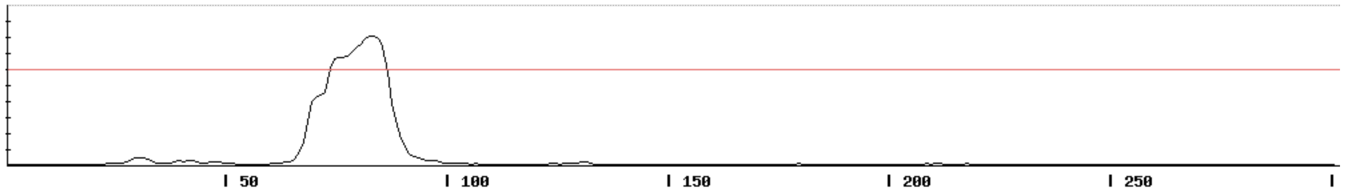

Predictions for CmsSPL13B

Viterbi Path

Viterbi Path

Posterior @ 0.6

149 - RKRLDGHNRRRRKP - 162

Posterior @ 0.6

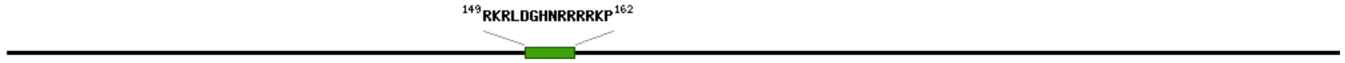

Posterior Graphic. Threshold

Marked
@ 0.6

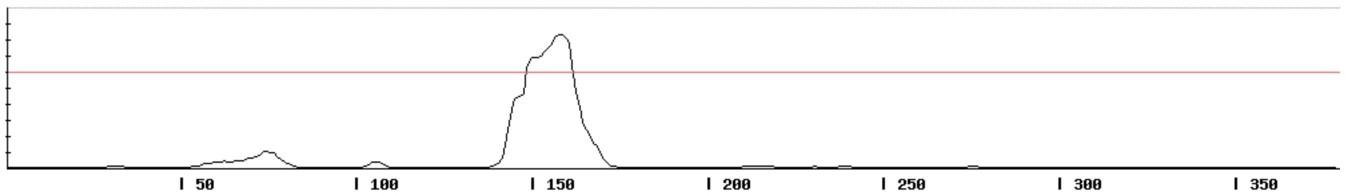

Predictions for CicSPL13A

Viterbi Path	
Viterbi Path	
Posterior @ 0.6	22 - RKRLDGHNRRRRK - 34
Posterior @ 0.6	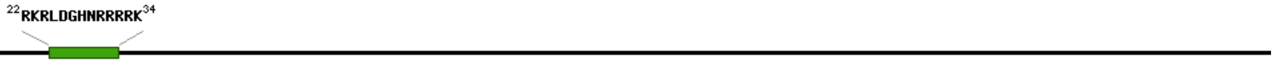
Posterior Graphic. Threshold Marked @ 0.6	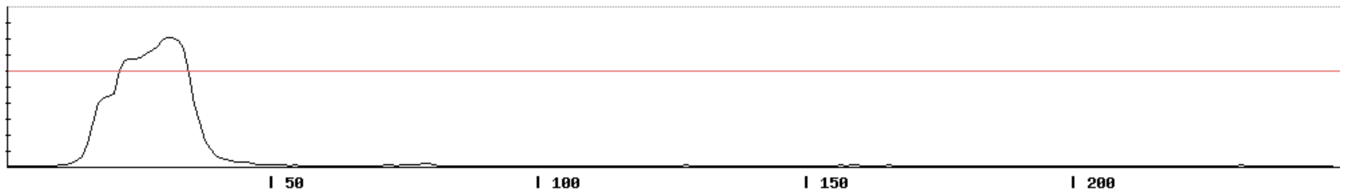

Predictions for CicSPL13B

Viterbi Path	
Viterbi Path	
Posterior @ 0.6	149 - RKRLDGHNRRRRKP - 162
Posterior @ 0.6	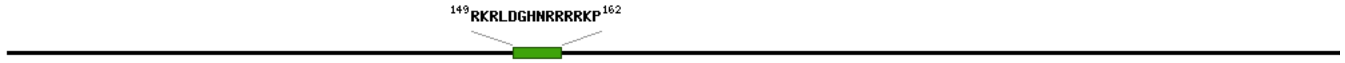
Posterior Graphic. Threshold Marked @ 0.6	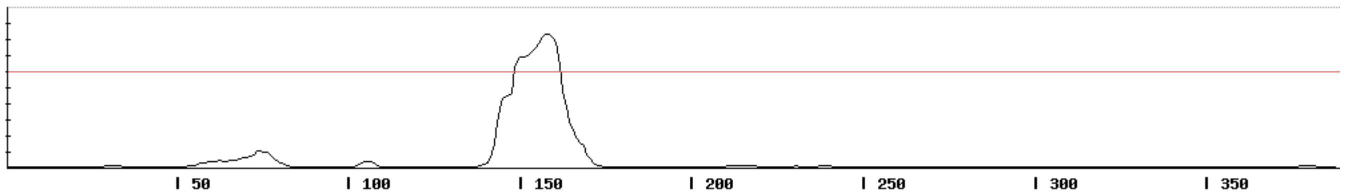

Predictions for CsSPL13A

Viterbi Path	
Viterbi Path	

Posterior @ 0.6	85 - RKRLDGHNRRRRK - 97
Posterior @ 0.6	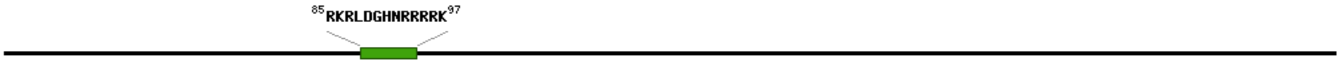
Posterior Graphic. Threshold Marked @ 0.6	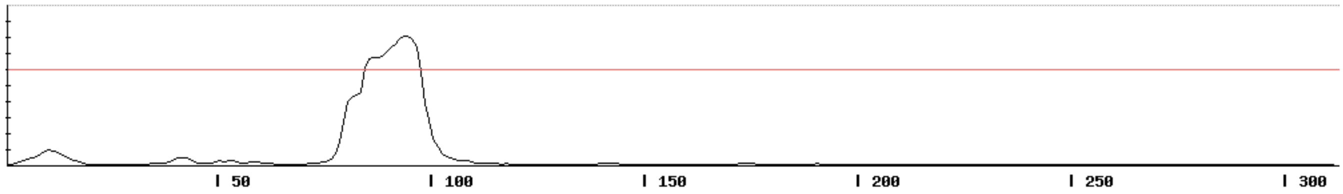

Predictions for CsSPL13B

Viterbi Path	
Viterbi Path	
Posterior @ 0.6	149 - RKRLDGHNRRRRK - 161
Posterior @ 0.6	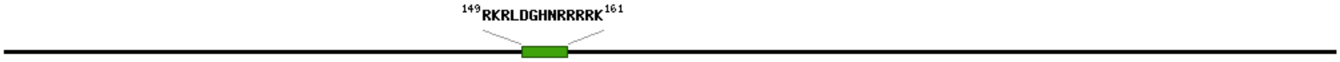
Posterior Graphic. Threshold Marked @ 0.6	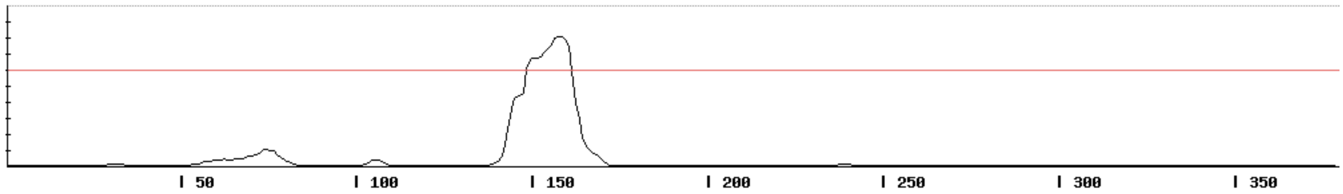

Predictions for CrSPL13A

Viterbi Path	
Viterbi Path	
Posterior @ 0.6	85 - RKRLDGHNRRRRK - 97
Posterior @ 0.6	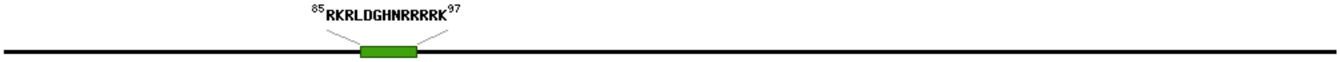
Posterior Graphic. Threshold Marked @ 0.6	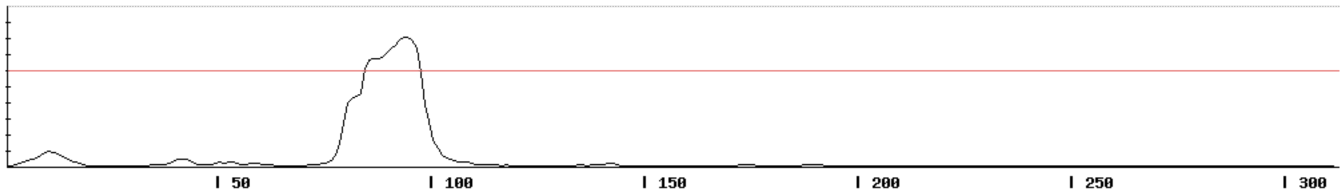

Predictions for CrSPL13B

Viterbi Path

Viterbi Path

Posterior @ 0.6

149 - RKRLDGHNRRRRK - 161

Posterior @ 0.6

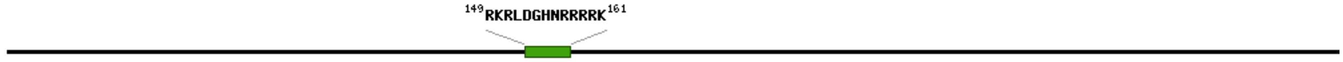

Posterior Graphic. Threshold Marked @ 0.6

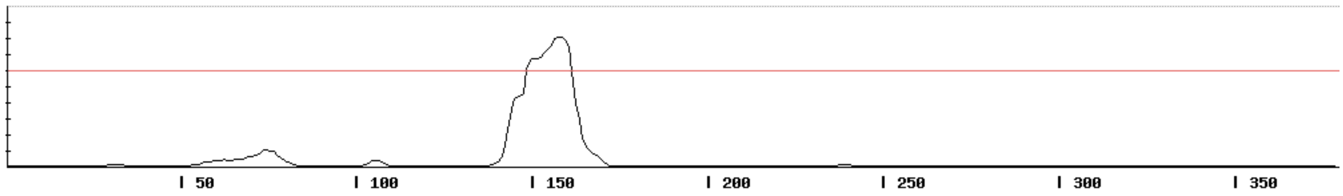

Predictions for ChSPL13A

Viterbi Path

Viterbi Path

Posterior @ 0.6

21 - RKRLDGHNRRRRK - 33

Posterior @ 0.6

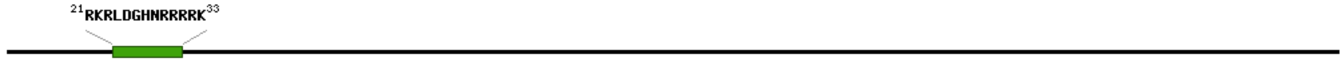

Posterior Graphic. Threshold Marked @ 0.6

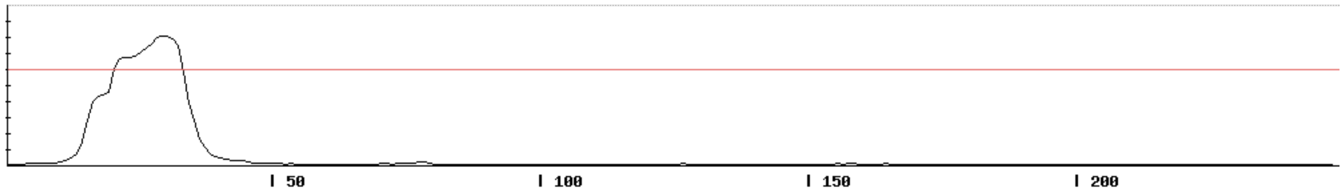

Predictions for ChSPL13B

Viterbi Path

Viterbi Path

Posterior @ 0.6

149 - RKRLDGHNRRRRKP - 162

Posterior @ 0.6

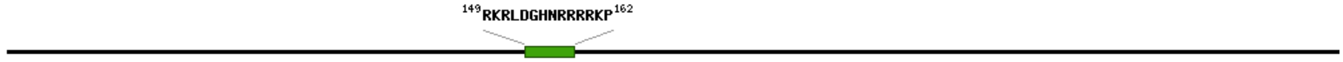

Posterior Graphic. Threshold

Marked
@ 0.6

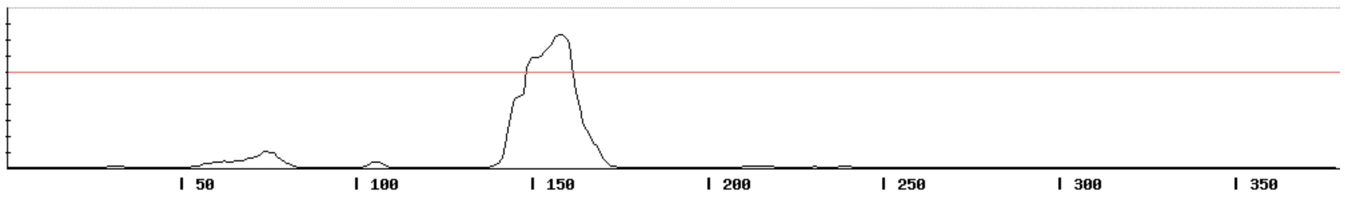

Predictions for CmjSPL13A

Viterbi Path

Viterbi Path

Posterior @ 0.6 — 21 - RKRLDGHNRRRRK - 33

Posterior @ 0.6

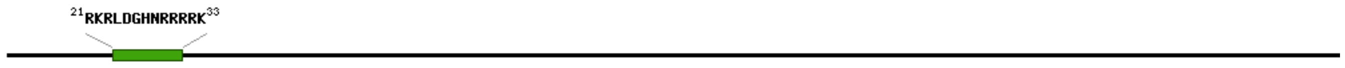

Posterior Graphic. Threshold Marked @ 0.6

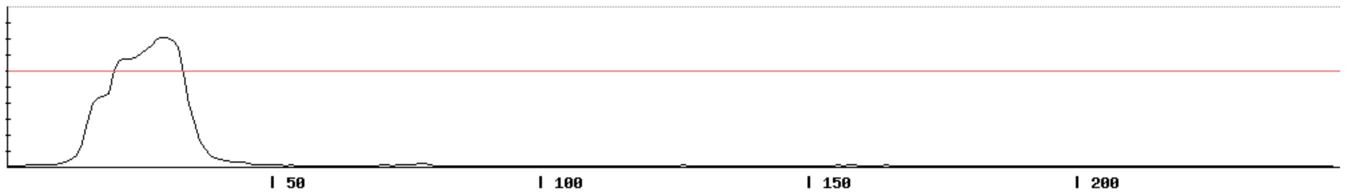

Predictions for CmjSPL13B

Viterbi Path

Viterbi Path

Posterior @ 0.6 — 149 - RKRLDGHNRRRRKP - 162

Posterior @ 0.6

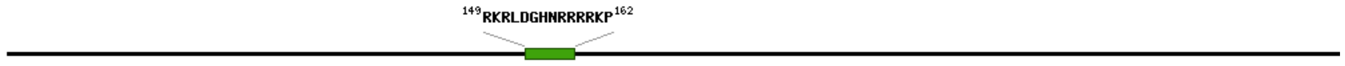

Posterior Graphic. Threshold Marked @ 0.6

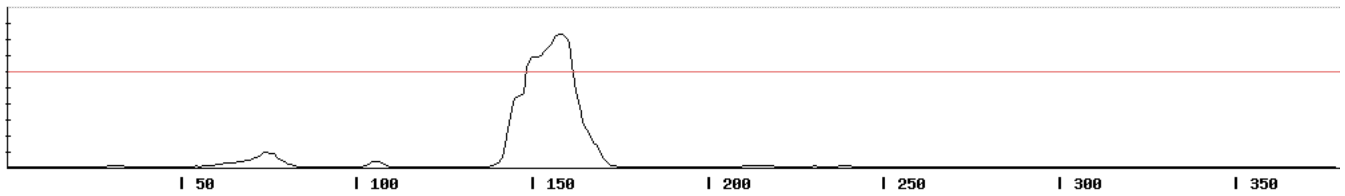

Predictions for CzpSPL13B

Viterbi Path

Viterbi Path

Posterior @ 0.6

149 - RKRLDGHNRRRRKP - 162

Posterior @ 0.6

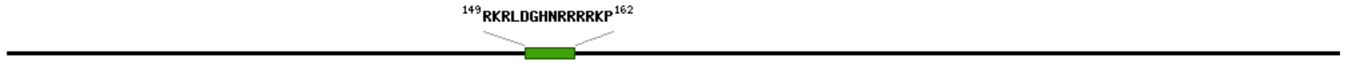

Posterior Graphic. Threshold Marked @ 0.6

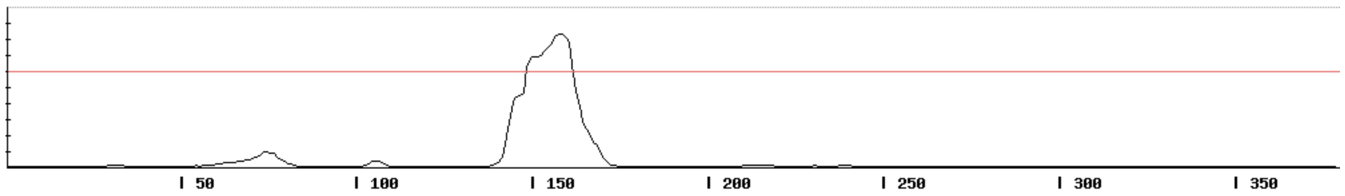

Predictions for CmSPL13B

Viterbi Path

Viterbi Path

Posterior @ 0.6

149 - RKRLDGHNRRRRKP - 162

Posterior @ 0.6

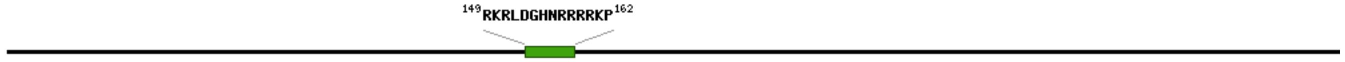

Posterior Graphic. Threshold Marked @ 0.6

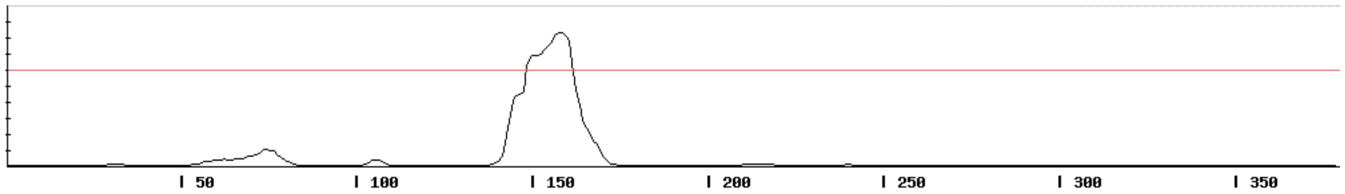

Predictions for CmSPL13A

Viterbi Path

Viterbi Path

Posterior @ 0.6

85 - RKRLDGHNRRRRK - 97

Posterior @ 0.6

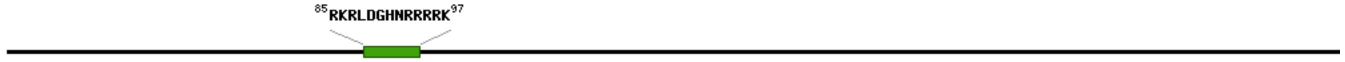

Posterior Graphic. Threshold Marked @ 0.6

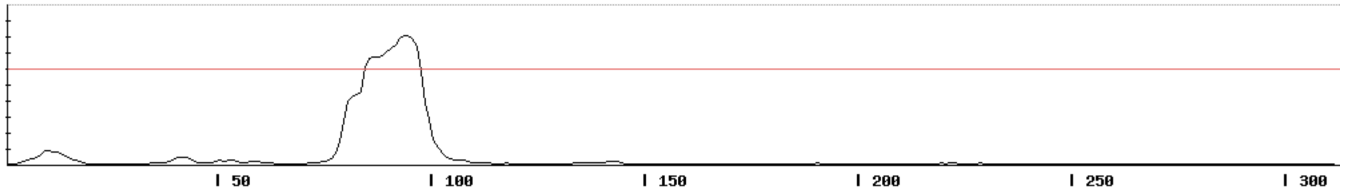

Predictions for AtSPL9

Viterbi Path	
Viterbi Path	
Posterior @ 0.6	144 - RRR - 146
Posterior @ 0.6	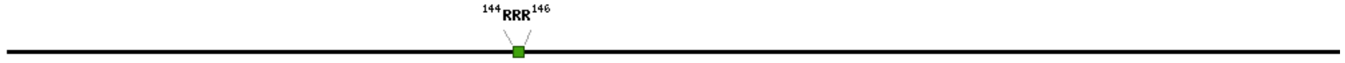
Posterior Graphic. Threshold Marked @ 0.6	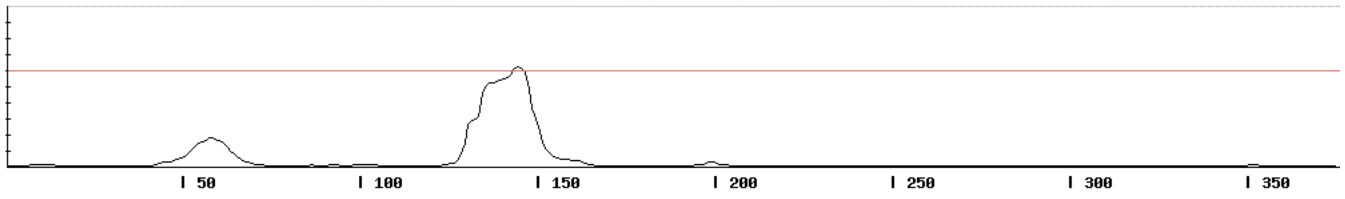

Predictions for AmSPL9

Viterbi Path	
Viterbi Path	
Posterior @ 0.6	158 - RRR - 160
Posterior @ 0.6	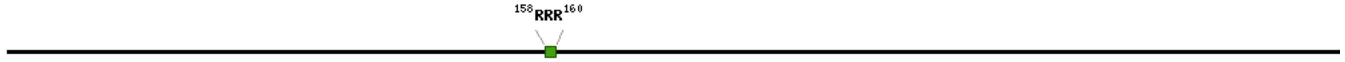
Posterior Graphic. Threshold Marked @ 0.6	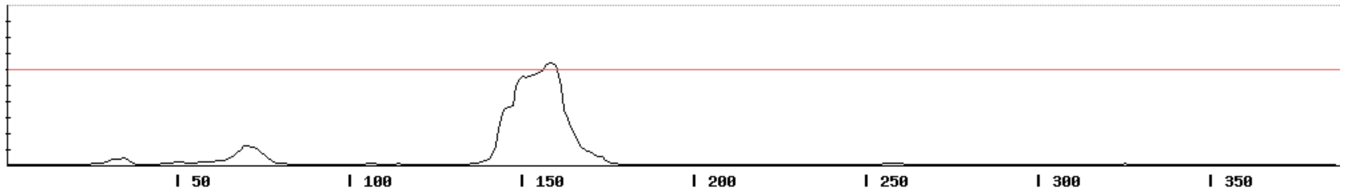

Predictions for MuSPL9

Viterbi Path	
Viterbi Path	
Posterior @ 0.6	160 - RRR - 162
Posterior @ 0.6	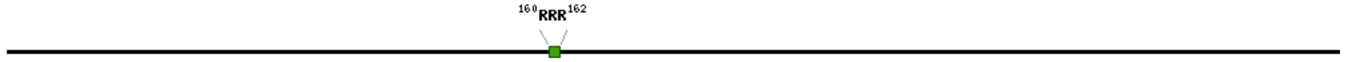
Posterior Graphic. Threshold	

Marked
@ 0.6

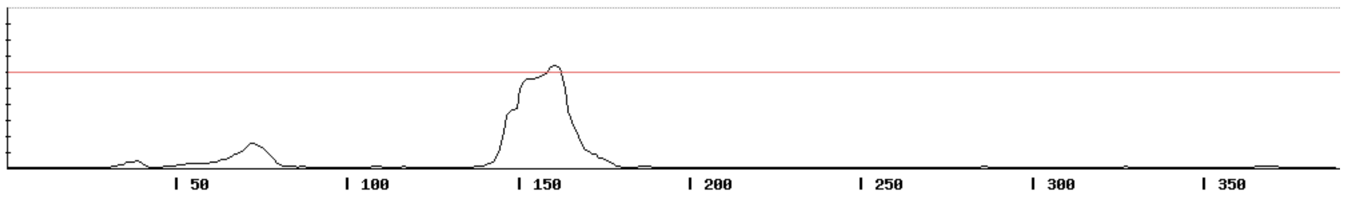

Predictions for AbSPL9

Viterbi Path	
Viterbi Path	
Posterior @ 0.6	160 - RRR - 162

Posterior
@ 0.6

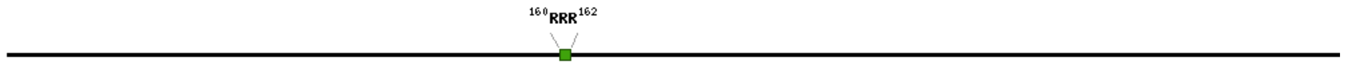

Posterior
Graphic.
Threshold
Marked
@ 0.6

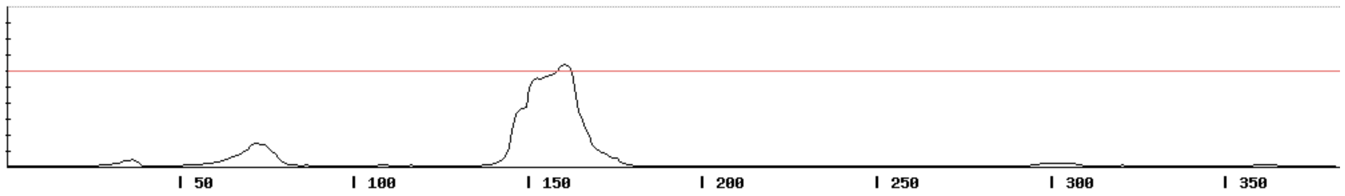

Predictions for ClSPL9

Viterbi Path	
Viterbi Path	
Posterior @ 0.6	152 - RRR - 154

Posterior
@ 0.6

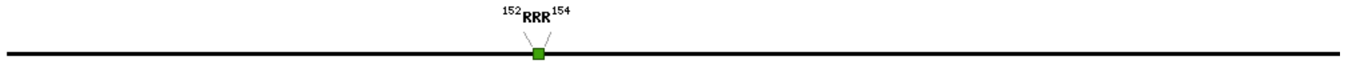

Posterior
Graphic.
Threshold
Marked
@ 0.6

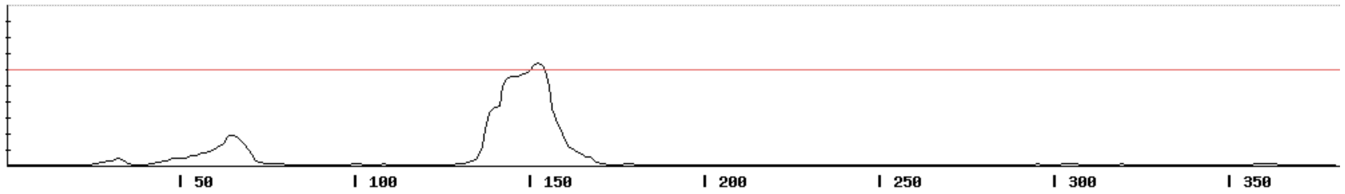

Predictions for CitSPL9

Viterbi
Path

Viterbi
Path

Posterior @ 0.6

155 - RRR - 157

Posterior @ 0.6

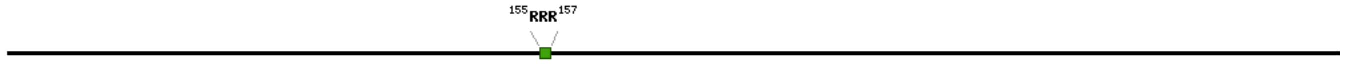

Posterior Graphic. Threshold Marked @ 0.6

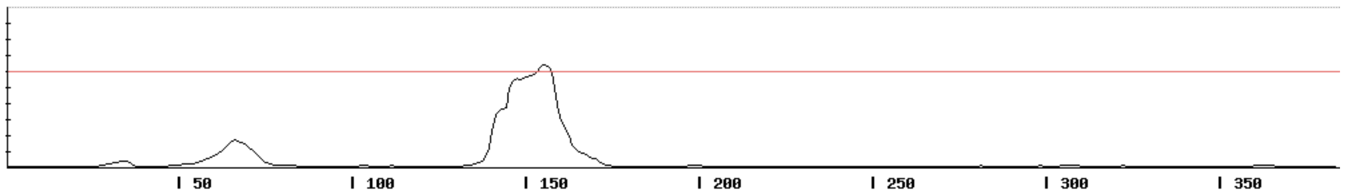

Predictions for PtSPL9

Viterbi Path

Viterbi Path

Posterior @ 0.6

159 - ERRRK - 163

Posterior @ 0.6

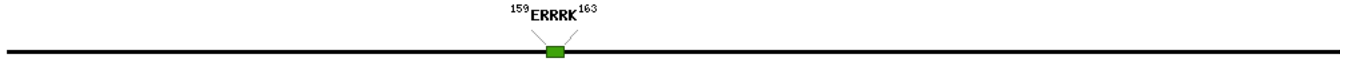

Posterior Graphic. Threshold Marked @ 0.6

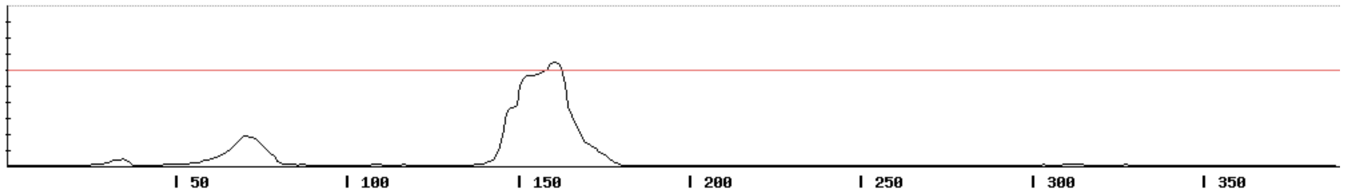

Predictions for FhSPL9

Viterbi Path

Viterbi Path

Posterior @ 0.6

159 - RRR - 161

Posterior @ 0.6

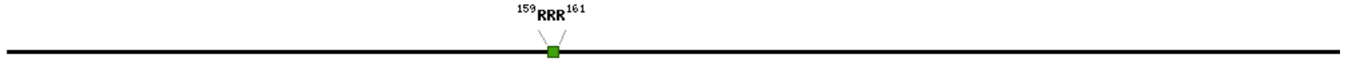

Posterior Graphic. Threshold Marked @ 0.6

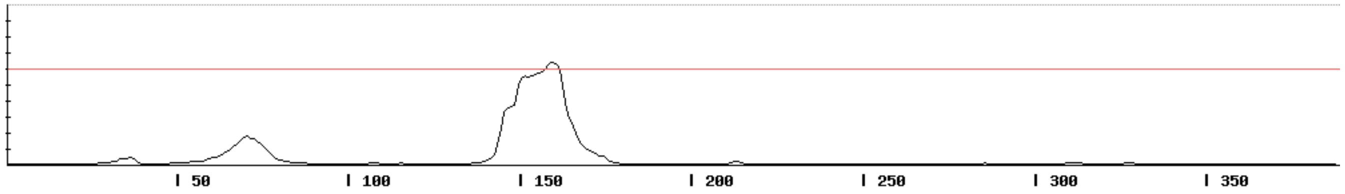

Predictions for CmsSPL9

Viterbi Path

Viterbi Path

Posterior @ 0.6 | 165 - RRR - 167

Posterior @ 0.6

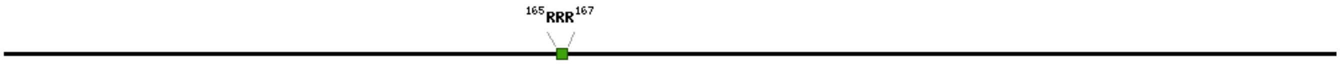

Posterior Graphic. Threshold Marked @ 0.6

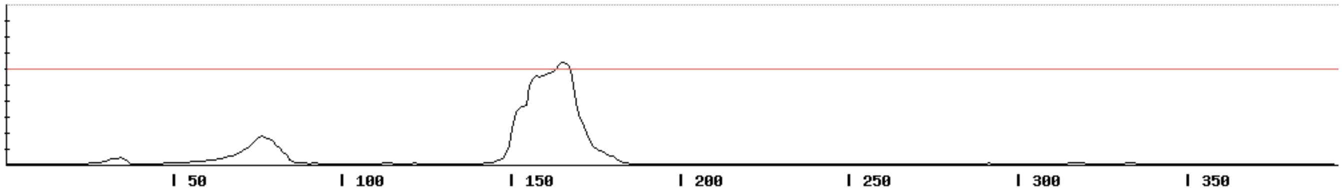

Predictions for CicSPL9

Viterbi Path

Viterbi Path

Posterior @ 0.6 | 155 - RRR - 157

Posterior @ 0.6

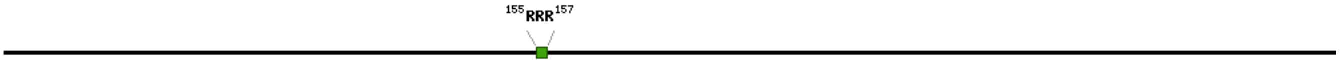

Posterior Graphic. Threshold Marked @ 0.6

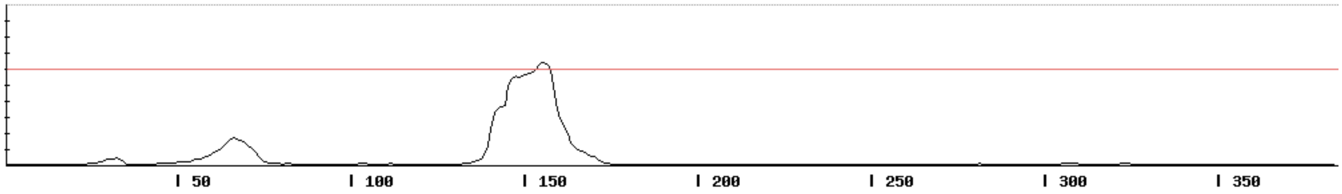

Predictions for CsSPL9

Viterbi Path

Viterbi Path

Posterior @ 0.6 | 157 - RRR - 159

Posterior @ 0.6

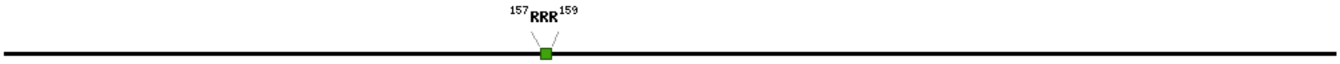

Posterior Graphic. Threshold

Marked
@ 0.6

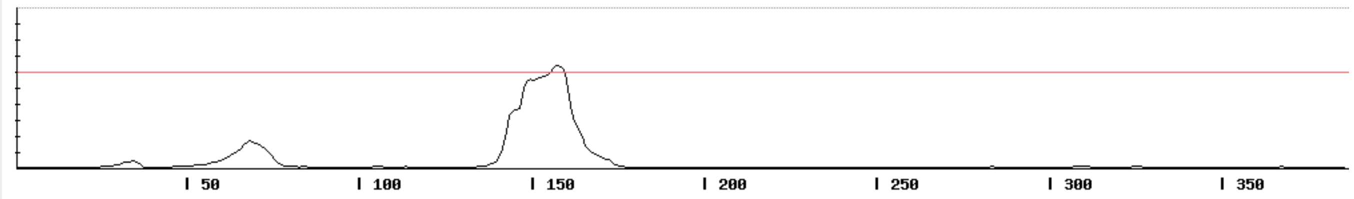

Predictions for CrSPL9

Viterbi Path

Viterbi Path

Posterior @ 0.6

157 - RRR - 159

Posterior @ 0.6

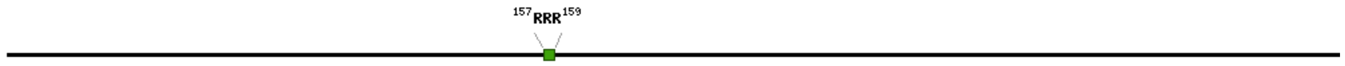

Posterior Graphic. Threshold Marked @ 0.6

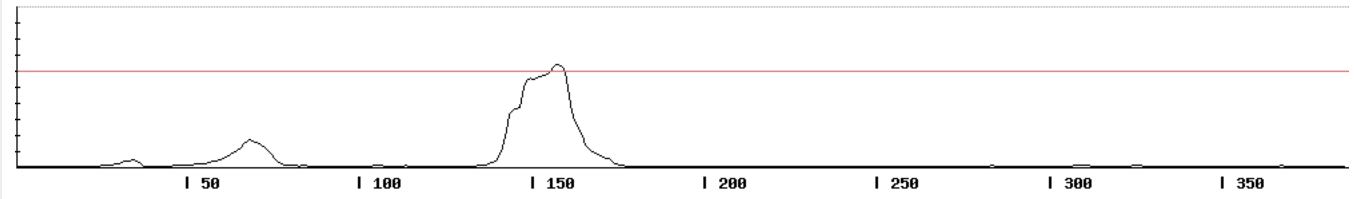

Predictions for ChSPL9

Viterbi Path

Viterbi Path

Posterior @ 0.6

159 - RRR - 161

Posterior @ 0.6

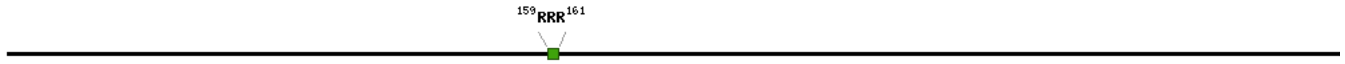

Posterior Graphic. Threshold Marked @ 0.6

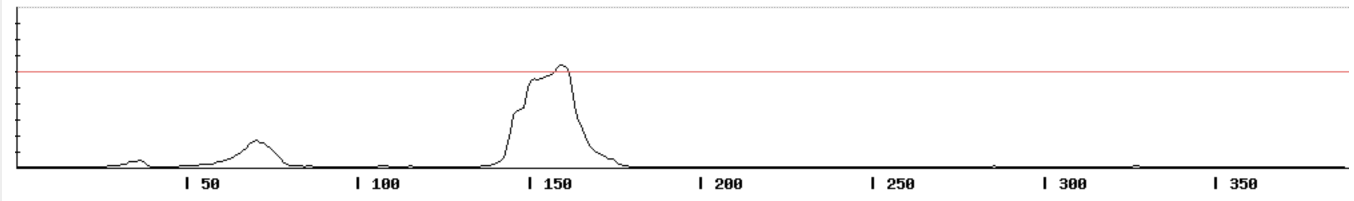

Predictions for CmjSPL9

Viterbi Path

Viterbi Path

Posterior @ 0.6	159 - RRR - 161
Posterior @ 0.6	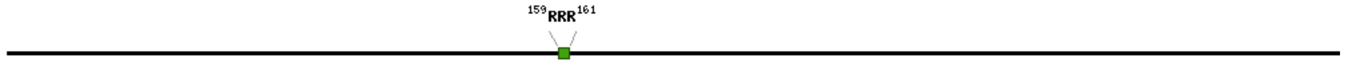
Posterior Graphic. Threshold Marked @ 0.6	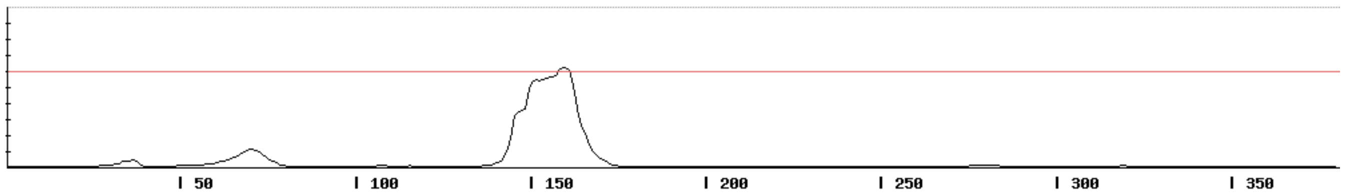

Predictions for CzpSPL9

Viterbi Path	
Viterbi Path	
Posterior @ 0.6	161 - RRR - 163
Posterior @ 0.6	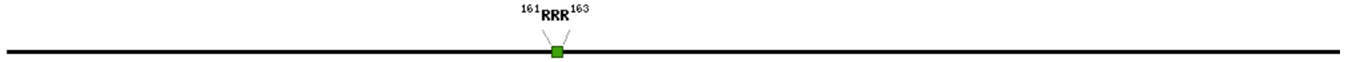
Posterior Graphic. Threshold Marked @ 0.6	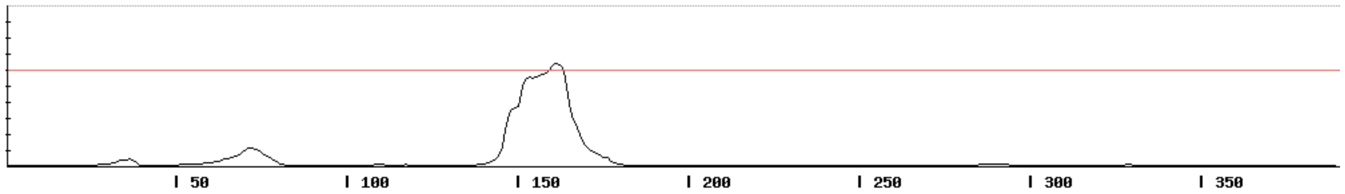

Predictions for CmSPL9

Viterbi Path	
Viterbi Path	
Posterior @ 0.6	155 - RRR - 157
Posterior @ 0.6	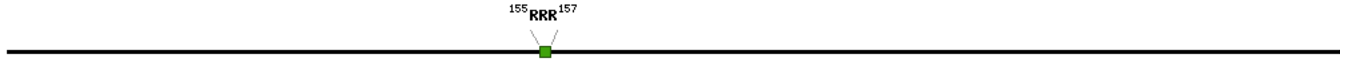
Posterior Graphic. Threshold Marked @ 0.6	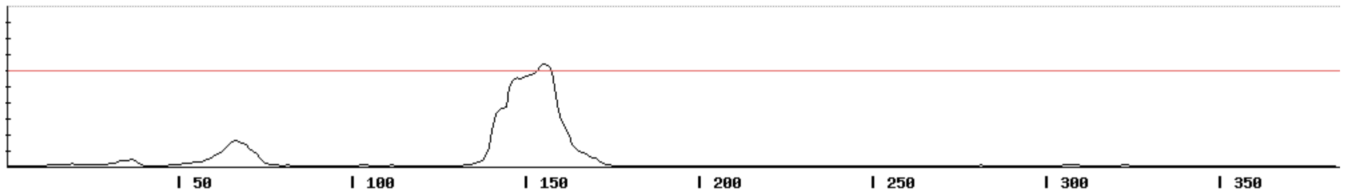

Predictions for AmSPL10

Viterbi Path

Viterbi Path

Posterior @ 0.6

Posterior @ 0.6

Posterior Graphic. Threshold Marked @ 0.6

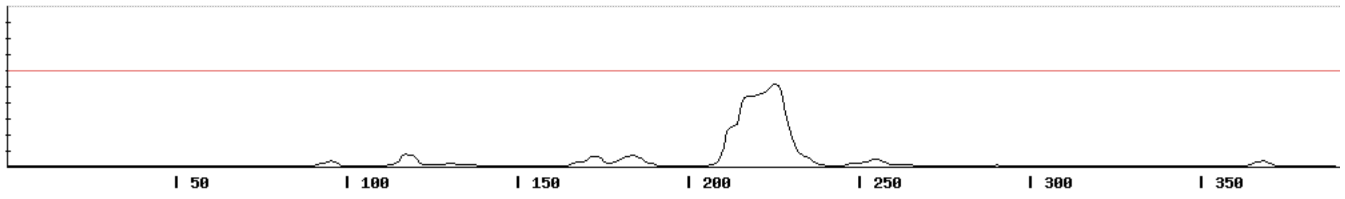

Predictions for MuSPL10

Viterbi Path

Viterbi Path

Posterior @ 0.6

Posterior @ 0.6

Posterior Graphic. Threshold Marked @ 0.6

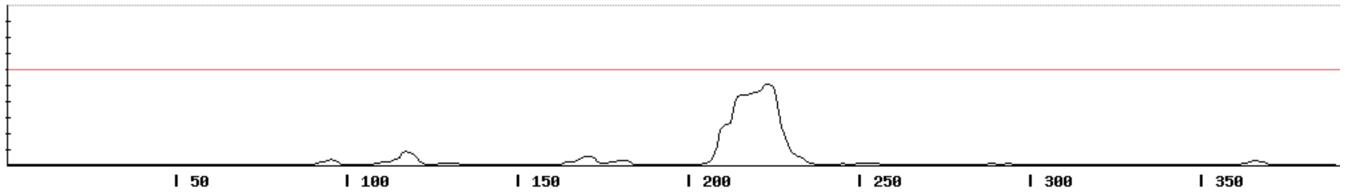

Predictions for AbSPL10

Viterbi Path

Viterbi Path

Posterior @ 0.6

Posterior @ 0.6

Posterior Graphic. Threshold

Marked
@ 0.6

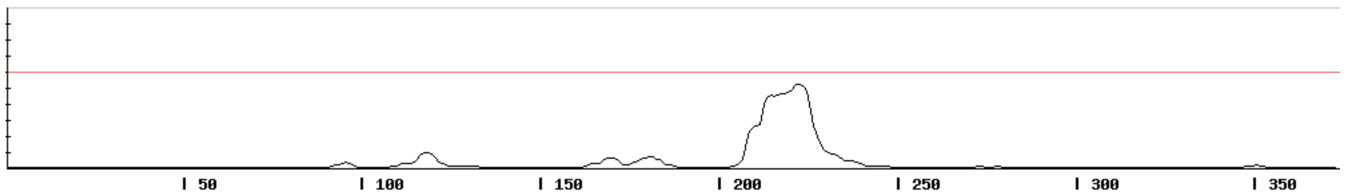

Predictions for ClSPL10

Viterbi Path

Viterbi Path

Posterior @ 0.6

Posterior @ 0.6

Posterior Graphic. Threshold Marked @ 0.6

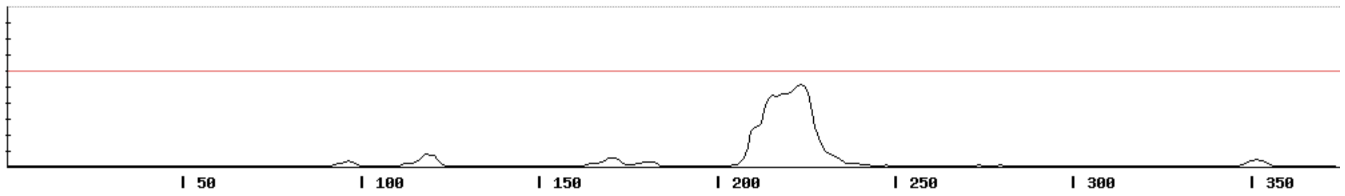

Predictions for CitSPL10

Viterbi Path

Viterbi Path

Posterior @ 0.6

Posterior @ 0.6

Posterior Graphic. Threshold Marked @ 0.6

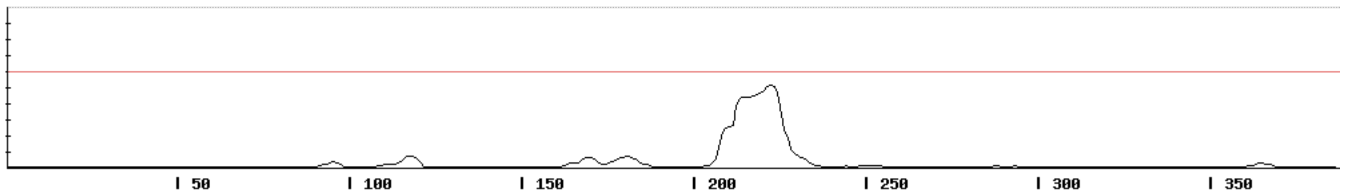

Predictions for PtSPL10

Viterbi Path

Viterbi Path

Posterior
@ 0.6

Posterior
@ 0.6

Posterior
Graphic.
Threshold
Marked
@ 0.6

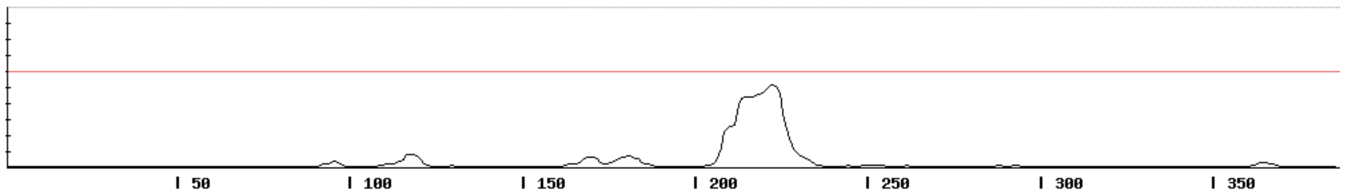

Predictions for FhSPL10

Viterbi
Path

Viterbi
Path

Posterior
@ 0.6

Posterior
@ 0.6

Posterior
Graphic.
Threshold
Marked
@ 0.6

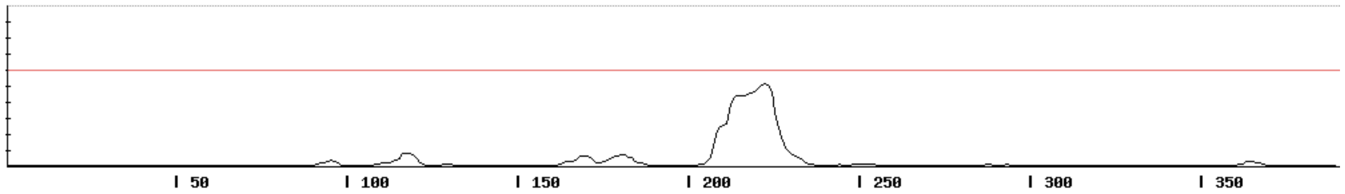

Predictions for CmsSPL10

Viterbi
Path

Viterbi
Path

Posterior
@ 0.6

Posterior
@ 0.6

Posterior
Graphic.
Threshold
Marked
@ 0.6

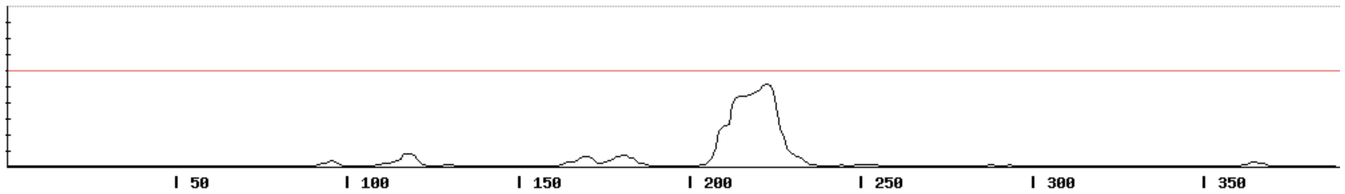

Predictions for CicSPL10

Viterbi Path

Viterbi Path

Posterior @ 0.6

Posterior @ 0.6

Posterior Graphic. Threshold Marked @ 0.6

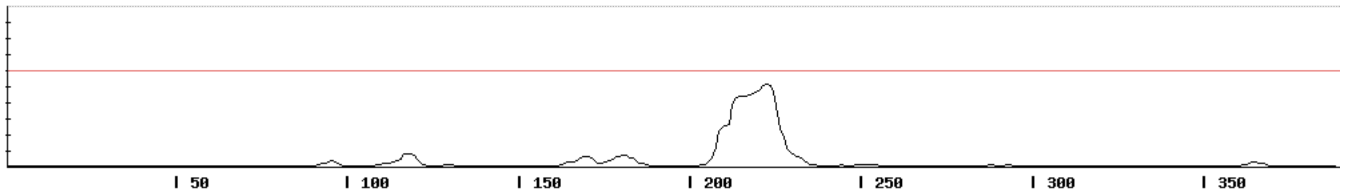

Predictions for CsSPL10

Viterbi Path

Viterbi Path

Posterior @ 0.6

Posterior @ 0.6

Posterior Graphic. Threshold Marked @ 0.6

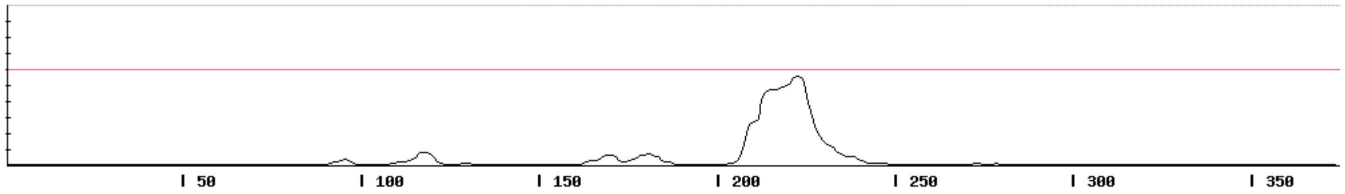

Predictions for CrSPL10

Viterbi Path

Viterbi Path

Posterior @ 0.6

Posterior @ 0.6

Posterior Graphic. Threshold

Marked
@ 0.6

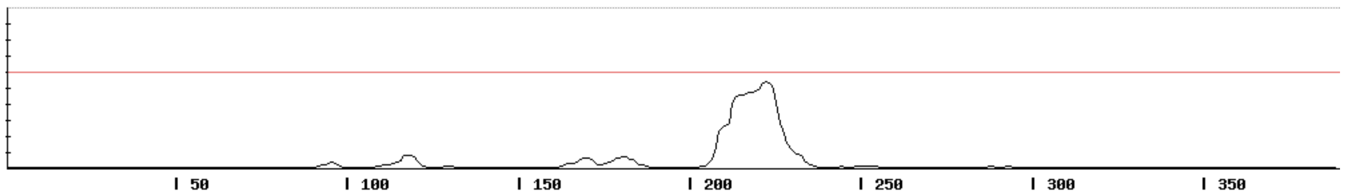

Predictions for ChSPL10

Viterbi
Path

Viterbi
Path

Posterior
@ 0.6

Posterior
@ 0.6

Posterior
Graphic.
Threshold
Marked
@ 0.6

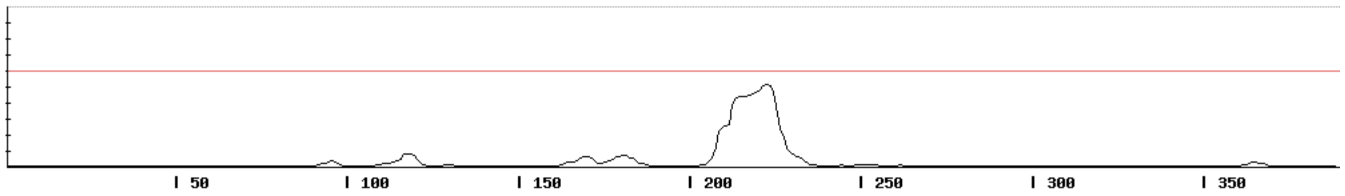

Predictions for CmjSPL10

Viterbi
Path

Viterbi
Path

Posterior
@ 0.6

Posterior
@ 0.6

Posterior
Graphic.
Threshold
Marked
@ 0.6

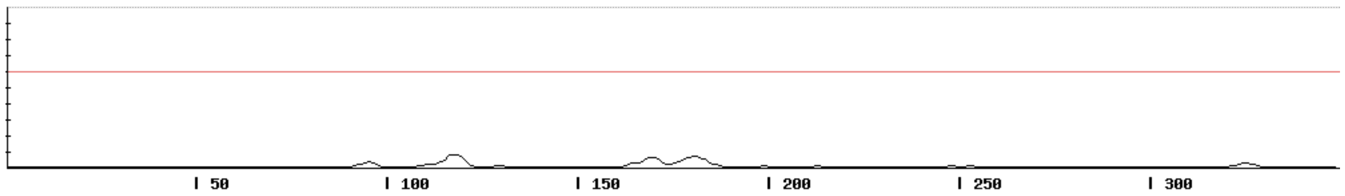

Predictions for CzpSPL10

Viterbi
Path

Viterbi
Path

Posterior @ 0.6

Posterior @ 0.6

Posterior Graphic. Threshold Marked @ 0.6

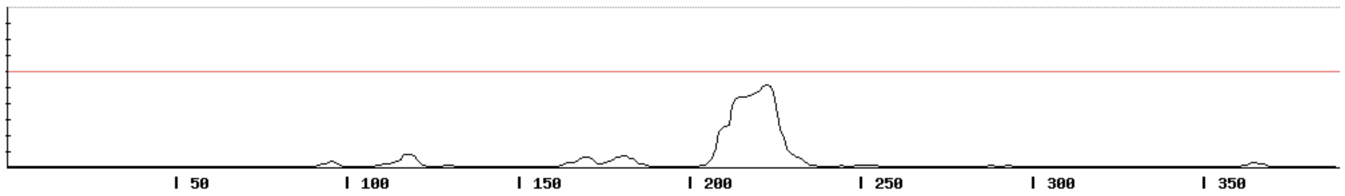

Predictions for CmSPL10

Viterbi Path

Viterbi Path

Posterior @ 0.6

Posterior @ 0.6

Posterior Graphic. Threshold Marked @ 0.6

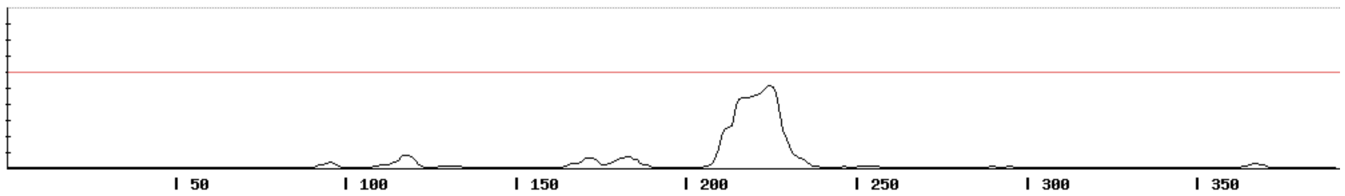

Predictions for AtSPL10

Viterbi Path

Viterbi Path

Posterior @ 0.6

236 - CRKRLSHHNARRRK - 249

Posterior @ 0.6

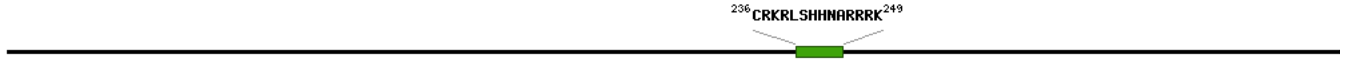

Posterior Graphic. Threshold Marked @ 0.6

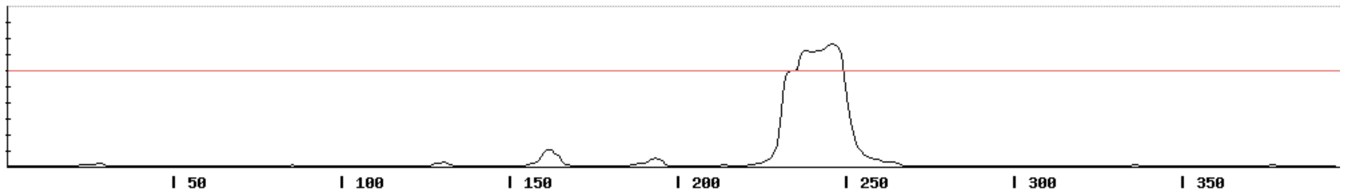

Predictions for AtSPL11

Viterbi Path

Viterbi Path

Posterior @ 0.6

236 - RKRLSHHNARRRK - 248

Posterior @ 0.6

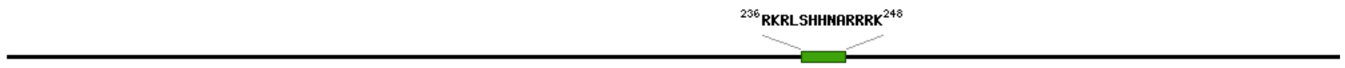

Posterior Graphic. Threshold Marked @ 0.6

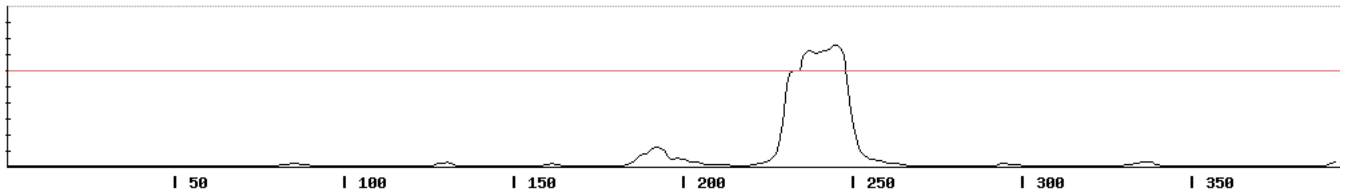

Predictions for AmSPL11

Viterbi Path

Viterbi Path

Posterior @ 0.6

255 - ARRRK - 259

Posterior @ 0.6

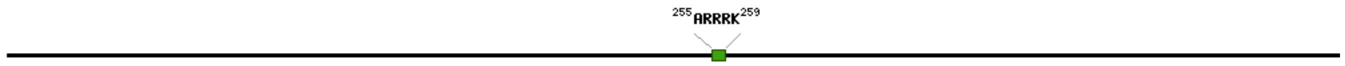

Posterior Graphic. Threshold Marked @ 0.6

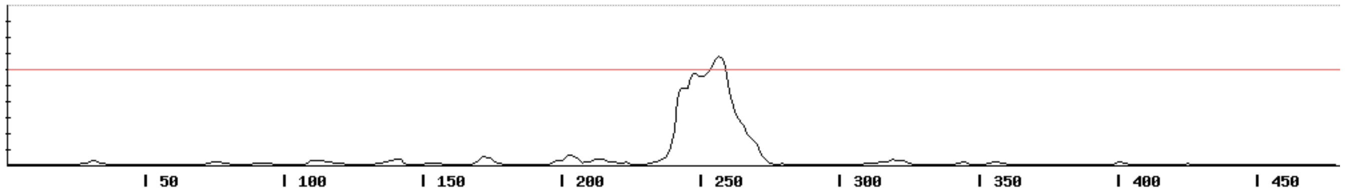

Predictions for MuSPL11

Viterbi Path

Viterbi Path

Posterior @ 0.6

Posterior @ 0.6

Posterior Graphic. Threshold

Marked
@ 0.6

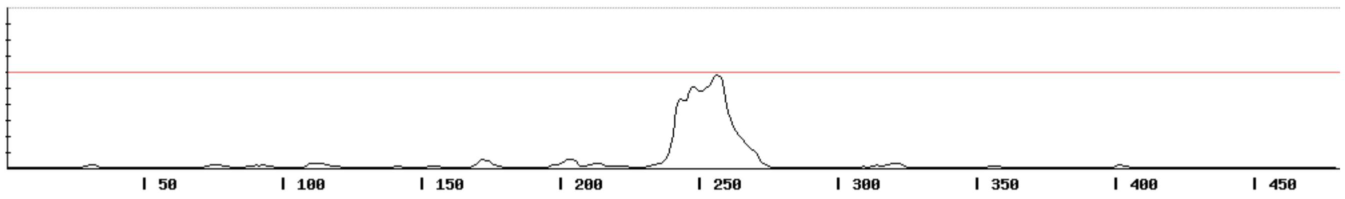

Predictions for AbSPL11

Viterbi
Path

Viterbi
Path

Posterior
@ 0.6

Posterior
@ 0.6

Posterior
Graphic.
Threshold
Marked
@ 0.6

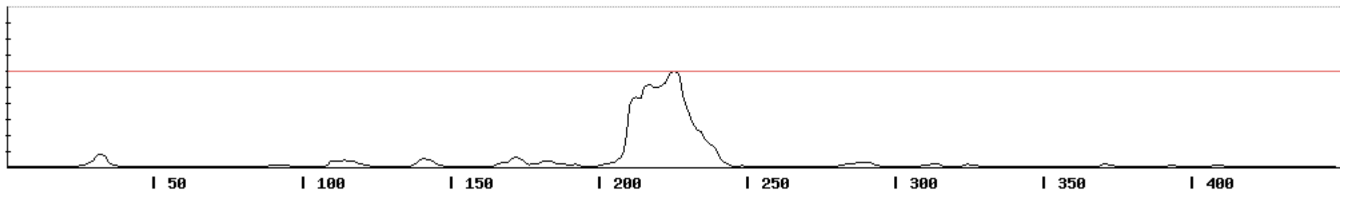

Predictions for ClSPL11

Viterbi
Path

Viterbi
Path

Posterior
@ 0.6

Posterior
@ 0.6

Posterior
Graphic.
Threshold
Marked
@ 0.6

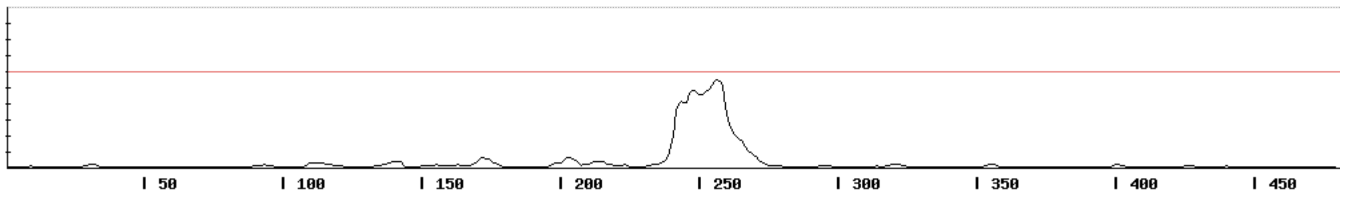

Predictions for CitSPL11

Viterbi
Path

Viterbi
Path

Posterior
@ 0.6

Posterior
@ 0.6

Posterior
Graphic.
Threshold
Marked
@ 0.6

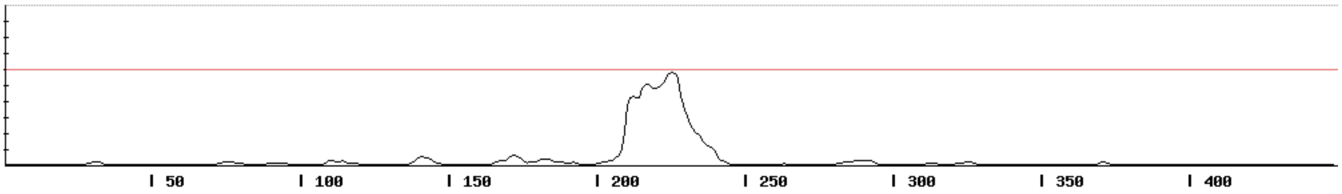

Predictions for PtSPL11

Viterbi
Path

Viterbi
Path

Posterior
@ 0.6

Posterior
@ 0.6

Posterior
Graphic.
Threshold
Marked
@ 0.6

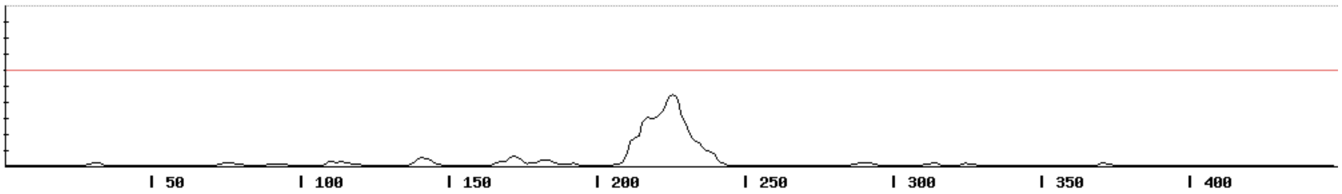

Predictions for FhSPL11

Viterbi
Path

Viterbi
Path

Posterior
@ 0.6

Posterior
@ 0.6

Posterior
Graphic.
Threshold
Marked
@ 0.6

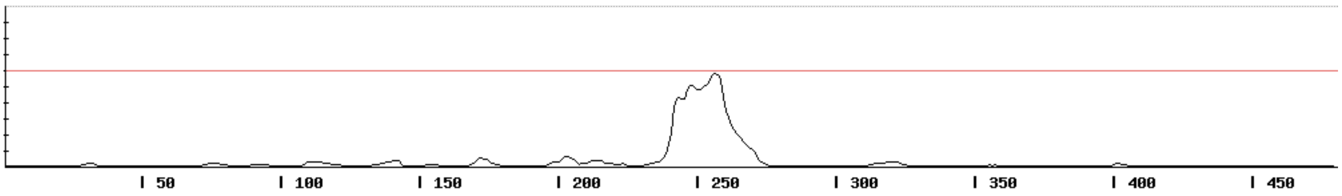

Predictions for CmsSPL11

Viterbi Path

Viterbi Path

Posterior @ 0.6

Posterior @ 0.6

Posterior Graphic. Threshold Marked @ 0.6

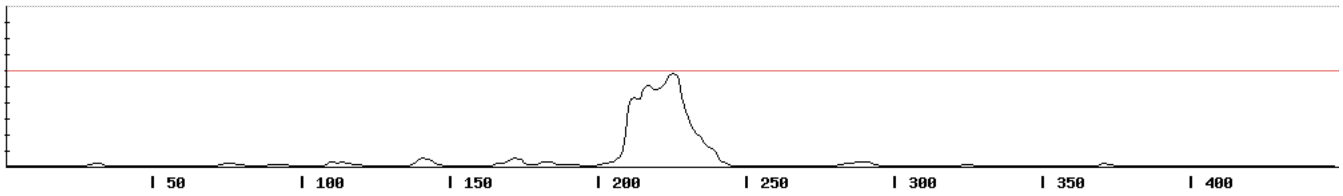

Predictions for CicSPL11

Viterbi Path

Viterbi Path

Posterior @ 0.6

Posterior @ 0.6

Posterior Graphic. Threshold Marked @ 0.6

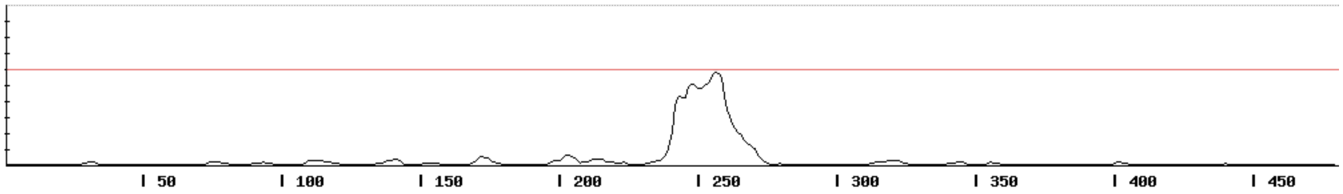

Predictions for CsSPL11

Viterbi Path

Viterbi Path

Posterior @ 0.6

Posterior @ 0.6

Posterior Graphic. Threshold

Marked
@ 0.6

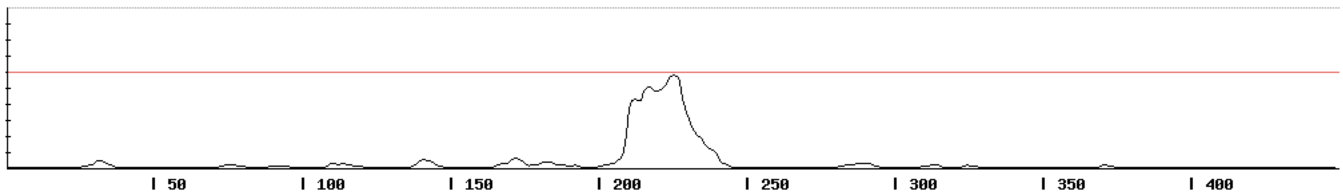

Predictions for CrSPL11

Viterbi
Path

Viterbi
Path

Posterior
@ 0.6

Posterior
@ 0.6

Posterior
Graphic.
Threshold
Marked
@ 0.6

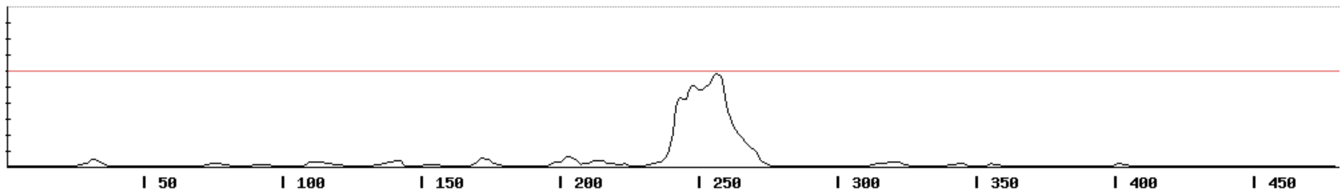

Predictions for ChSPL11

Viterbi
Path

Viterbi
Path

Posterior
@ 0.6

Posterior
@ 0.6

Posterior
Graphic.
Threshold
Marked
@ 0.6

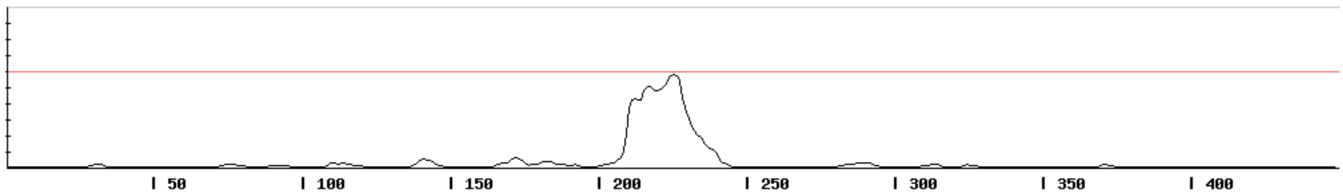

Predictions for CmjSPL11

Viterbi
Path

Viterbi
Path

Posterior @ 0.6

Posterior @ 0.6

Posterior Graphic. Threshold Marked @ 0.6

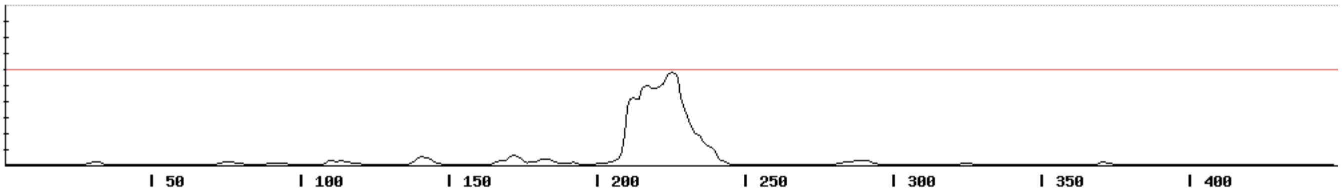

Predictions for CmSPL11

Viterbi Path

Viterbi Path

Posterior @ 0.6

Posterior @ 0.6

Posterior Graphic. Threshold Marked @ 0.6

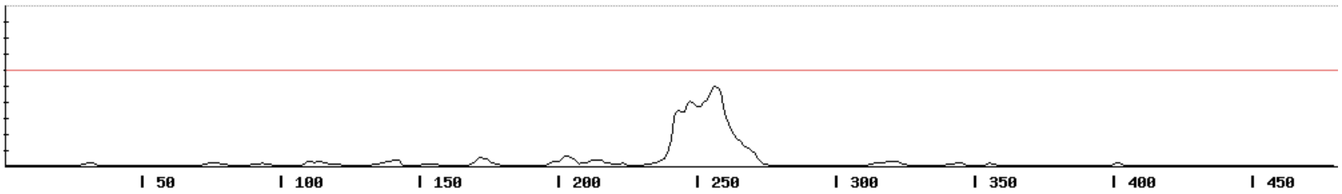

Predictions for AtSPL6

Viterbi Path

Viterbi Path

Posterior @ 0.6

186 - RRLAGHNERRRKP - 198

Posterior @ 0.6

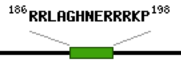

Posterior Graphic. Threshold Marked @ 0.6

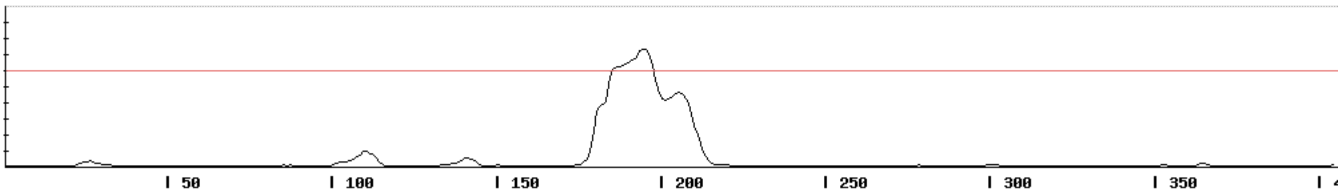

Predictions for AmSPL6A

Viterbi Path

Viterbi Path

Posterior @ 0.6

Posterior @ 0.6

Posterior Graphic. Threshold Marked @ 0.6

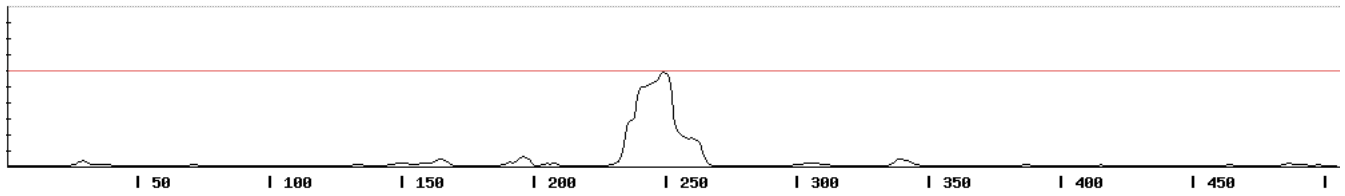

Predictions for AmSPL6B

Viterbi Path

Viterbi Path

Posterior @ 0.6

193 - RKRLAGHNERRRKPRVGIQHGR - 214

Posterior @ 0.6

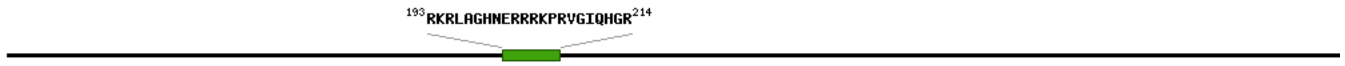

Posterior Graphic. Threshold Marked @ 0.6

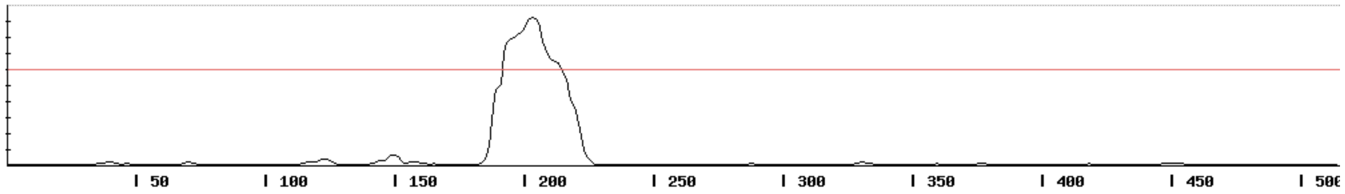

Predictions for MuSPL6A

Viterbi Path

Viterbi Path

Posterior @ 0.6

Posterior @ 0.6

Posterior Graphic. Threshold

Marked
@ 0.6

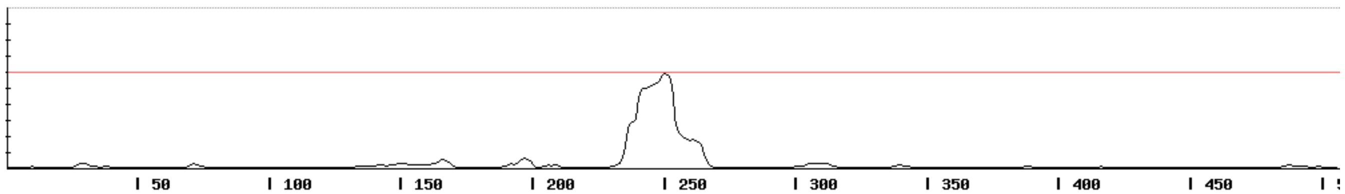

Predictions for MuSPL6B

Viterbi Path	
Viterbi Path	
Posterior @ 0.6	242 - RRR - 244
Posterior @ 0.6	
Posterior Graphic. Threshold Marked @ 0.6	

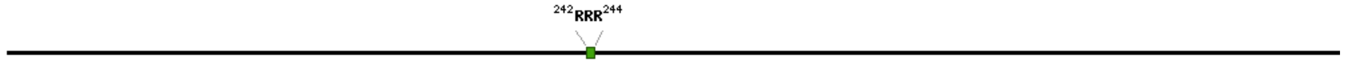

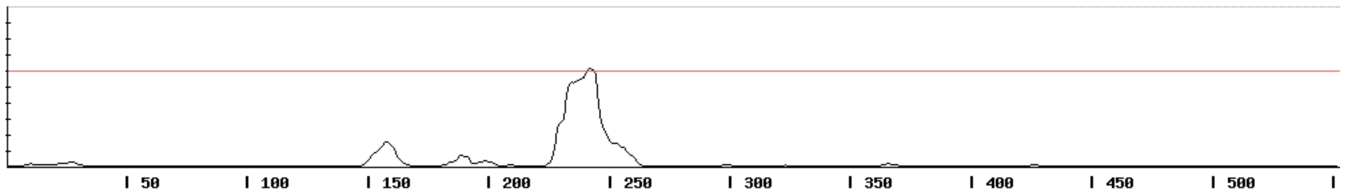

Predictions for AbSPL6A

Viterbi Path	
Viterbi Path	
Posterior @ 0.6	
Posterior @ 0.6	
Posterior Graphic. Threshold Marked @ 0.6	

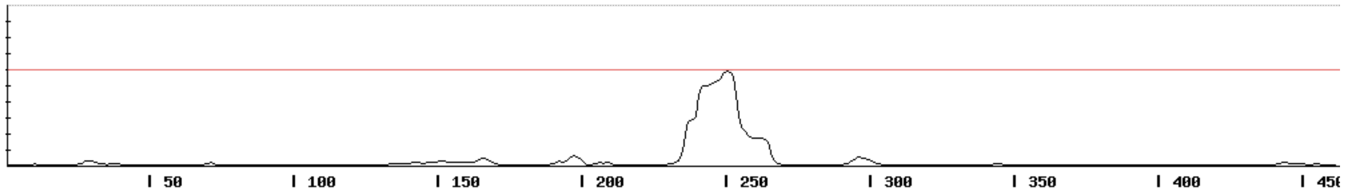

Predictions for AbSPL6B

Viterbi Path	
Viterbi Path	

Posterior @ 0.6	240 - NERRRK - 245
Posterior @ 0.6	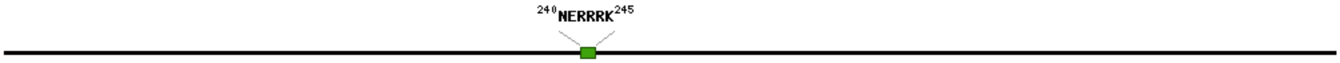
Posterior Graphic. Threshold Marked @ 0.6	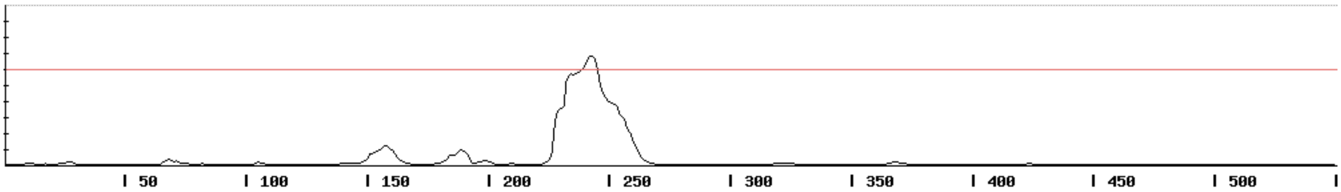

Predictions for ClSPL6A

Viterbi Path	
Viterbi Path	
Posterior @ 0.6	
Posterior @ 0.6	
Posterior Graphic. Threshold Marked @ 0.6	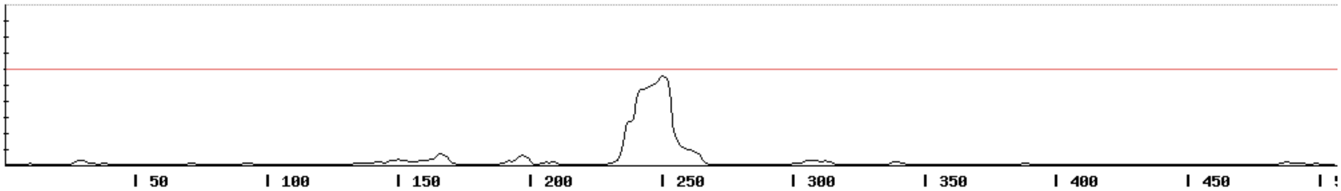

Predictions for ClSPL6B

Viterbi Path	
Viterbi Path	
Posterior @ 0.6	233 - RKRLAGHNERRRKPQGGIHP - 252
Posterior @ 0.6	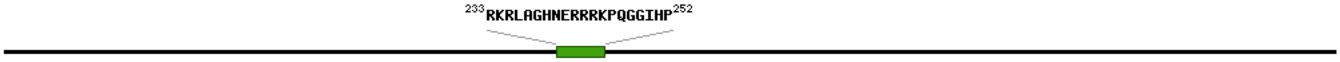
Posterior Graphic. Threshold Marked @ 0.6	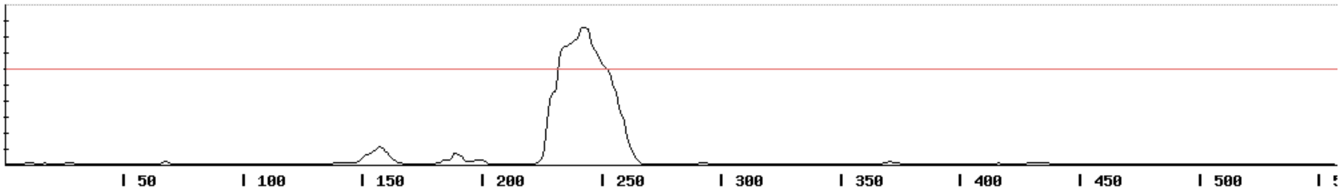

Predictions for CitSPL6A

Viterbi Path

Viterbi Path

Posterior @ 0.6

Posterior @ 0.6

Posterior Graphic. Threshold Marked @ 0.6

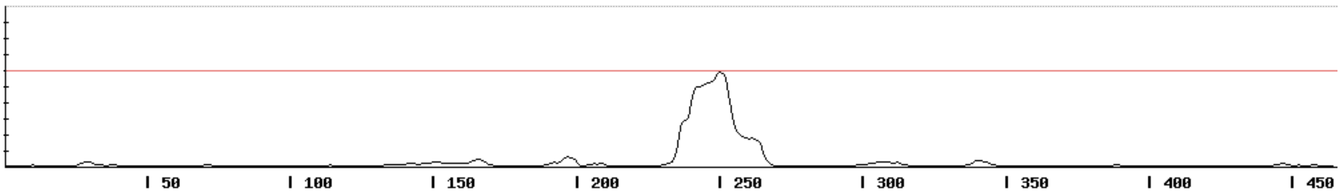

Predictions for CitSPL6B

Viterbi Path

Viterbi Path

Posterior @ 0.6

233 - RKRLAGHNERRRKPQVGI - 250

Posterior @ 0.6

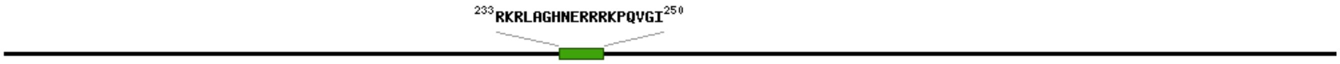

Posterior Graphic. Threshold Marked @ 0.6

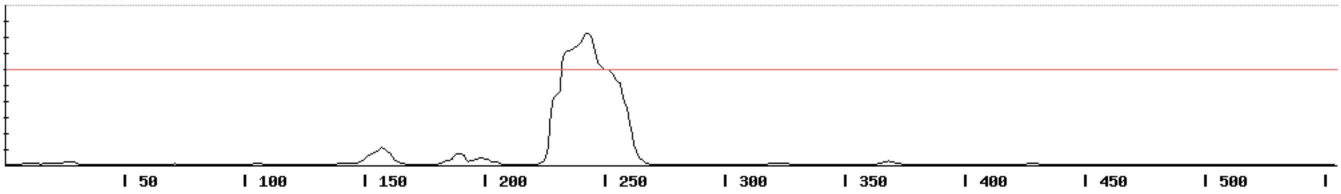

Predictions for PtSPL6A

Viterbi Path

Viterbi Path

Posterior @ 0.6

Posterior @ 0.6

Posterior Graphic. Threshold

Marked
@ 0.6

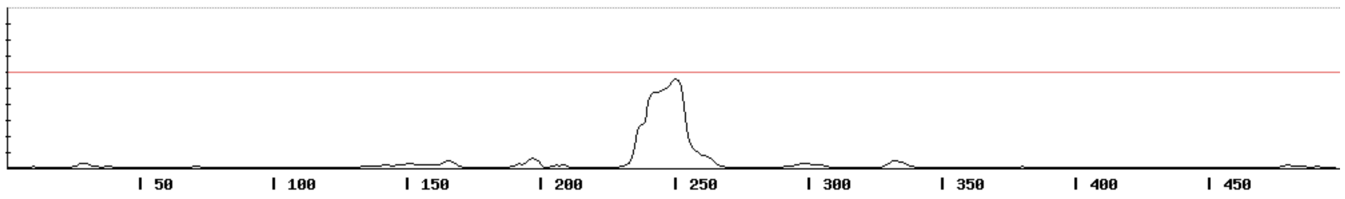

Predictions for PtSPL6B

Viterbi
Path

Viterbi
Path

Posterior
@ 0.6

234 - KRLAGHNERRRKP - 246

Posterior
@ 0.6

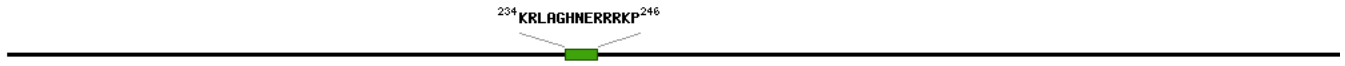

Posterior
Graphic.
Threshold
Marked
@ 0.6

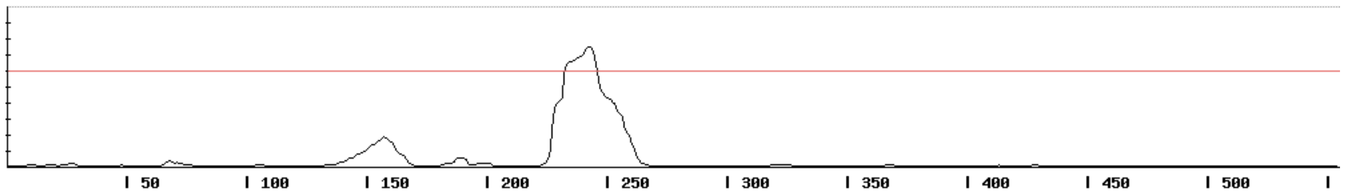

Predictions for FhSPL6A

Viterbi
Path

Viterbi
Path

Posterior
@ 0.6

130 - KKK - 132

149 - GRRKRR - 154

Posterior
@ 0.6

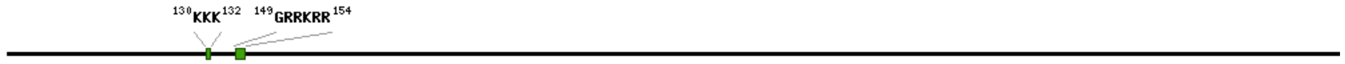

Posterior
Graphic.
Threshold
Marked
@ 0.6

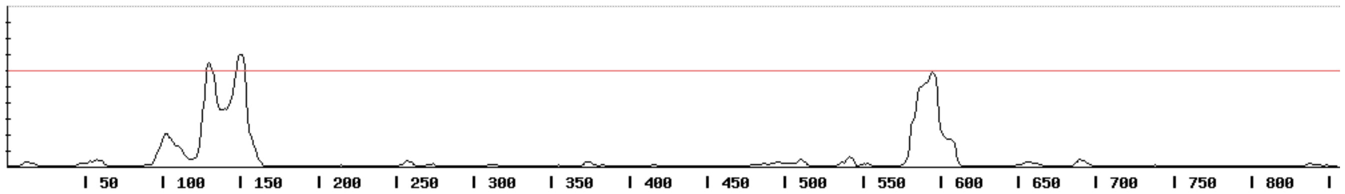

Predictions for FhSPL6B

Viterbi
Path

Viterbi
Path

Posterior @ 0.6

234 - KRLAGHNERRRKP - 246

Posterior @ 0.6

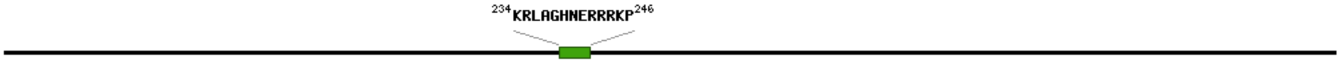

Posterior Graphic. Threshold Marked @ 0.6

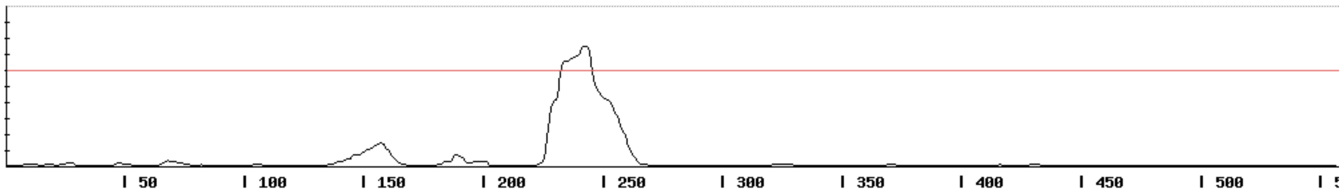

Predictions for CmsSPL6A

Viterbi Path

Viterbi Path

Posterior @ 0.6

Posterior @ 0.6

Posterior Graphic. Threshold Marked @ 0.6

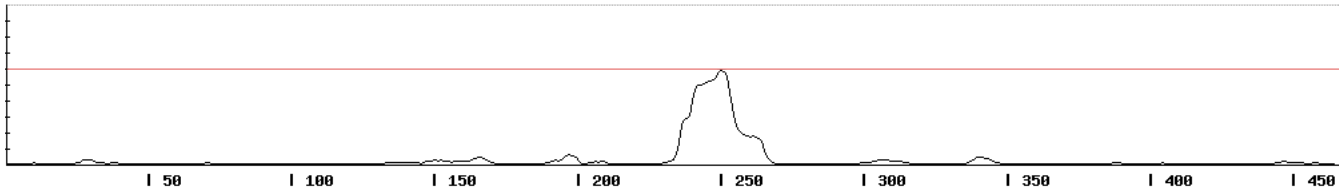

Predictions for CmsSPL6B

Viterbi Path

Viterbi Path

Posterior @ 0.6

234 - KRLAGHNERRRKP - 246

Posterior @ 0.6

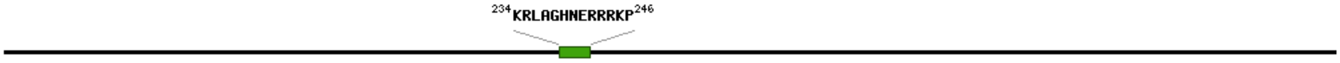

Posterior Graphic. Threshold Marked @ 0.6

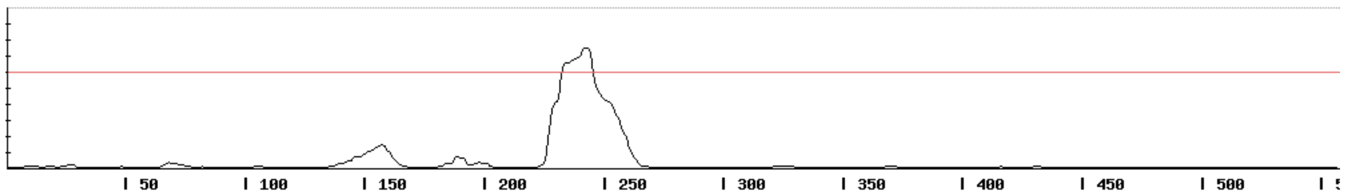

Predictions for CicSPL6A

Viterbi Path

Viterbi Path

Posterior @ 0.6

Posterior @ 0.6

Posterior Graphic. Threshold Marked @ 0.6

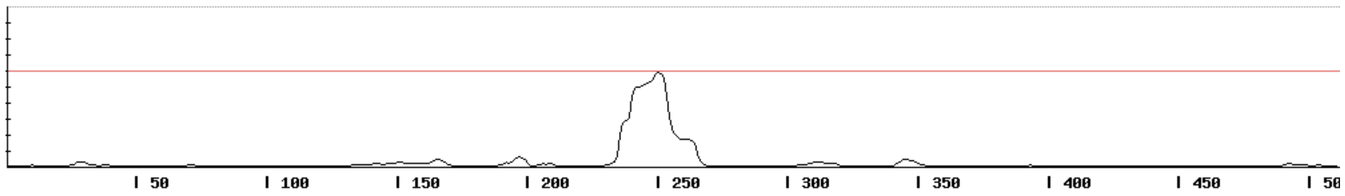

Predictions for CicSPL6B

Viterbi Path

Viterbi Path

Posterior @ 0.6

234 - KRLAGHNERRRKP - 246

Posterior @ 0.6

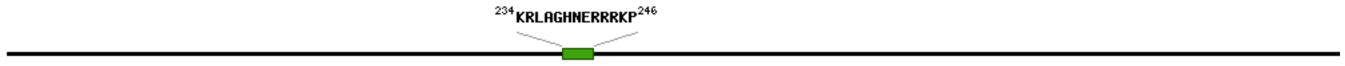

Posterior Graphic. Threshold Marked @ 0.6

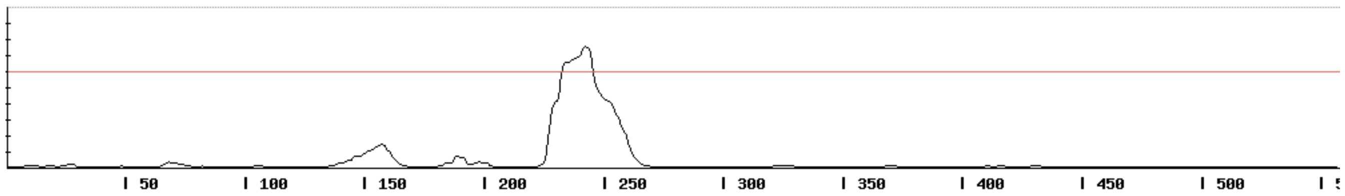

Predictions for CsSPL6A

Viterbi Path

Viterbi Path

Posterior @ 0.6	
Posterior @ 0.6	
Posterior Graphic. Threshold Marked @ 0.6	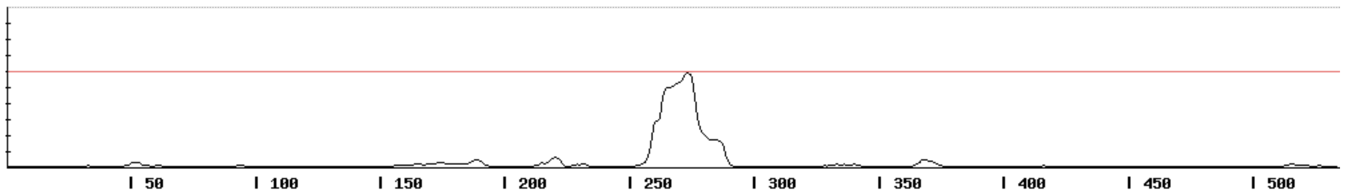

Predictions for CsSPL6B

Viterbi Path	
Viterbi Path	
Posterior @ 0.6	234 - KRLAGHNERRRKP - 246
Posterior @ 0.6	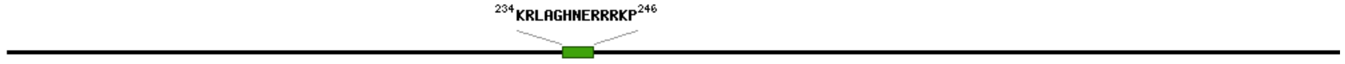
Posterior Graphic. Threshold Marked @ 0.6	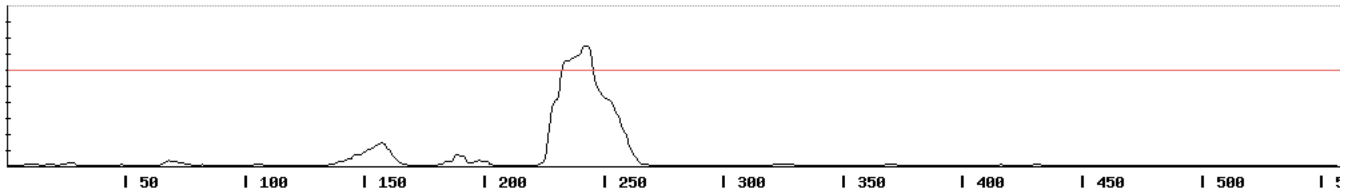

Predictions for CrSPL6A

Viterbi Path	
Viterbi Path	
Posterior @ 0.6	
Posterior @ 0.6	
Posterior Graphic. Threshold Marked @ 0.6	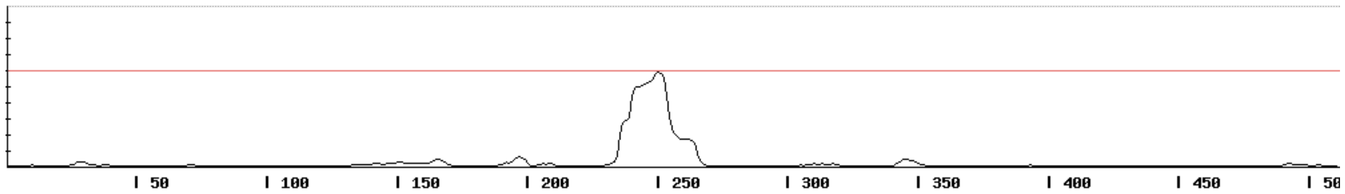

Predictions for CrSPL6B

Viterbi Path

Viterbi Path

Posterior @ 0.6

143 - KRLAGHNERRRKP - 155

Posterior @ 0.6

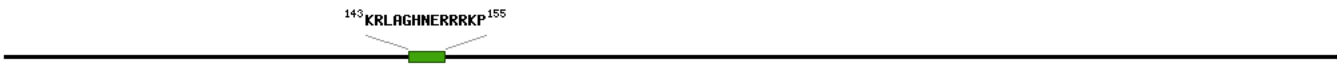

Posterior Graphic. Threshold Marked @ 0.6

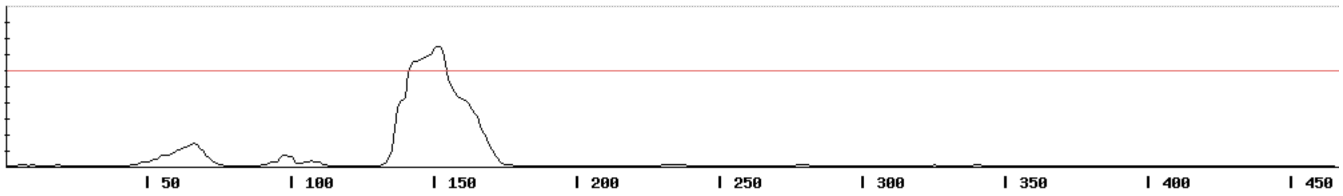

Predictions for ChSPL6A

Viterbi Path

Viterbi Path

Posterior @ 0.6

Posterior @ 0.6

Posterior Graphic. Threshold Marked @ 0.6

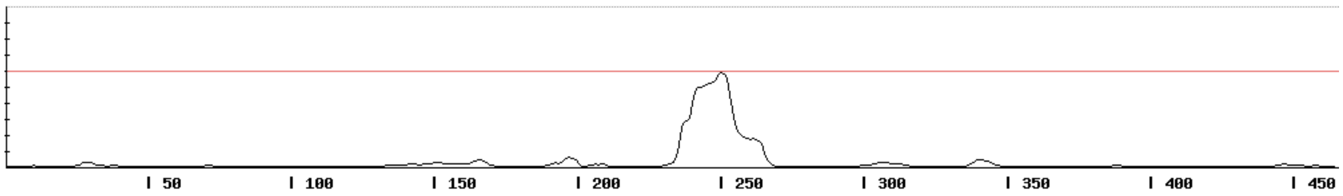

Predictions for ChSPL6B

Viterbi Path

Viterbi Path

Posterior @ 0.6

234 - KRLAGHNERRRKP - 246

Posterior @ 0.6

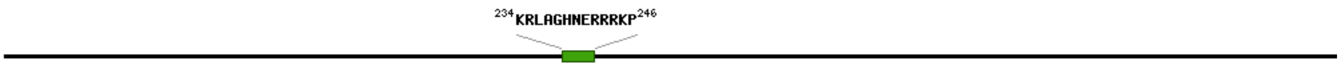

Posterior Graphic. Threshold

Marked
@ 0.6

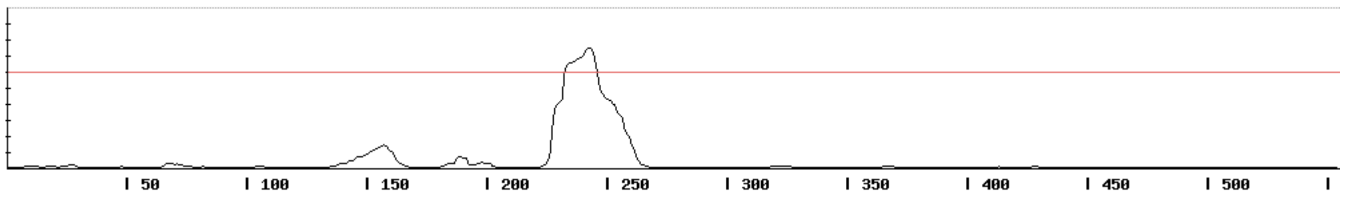

Predictions for CmjSPL6A

Viterbi Path

Viterbi Path

Posterior @ 0.6

Posterior @ 0.6

Posterior Graphic. Threshold Marked @ 0.6

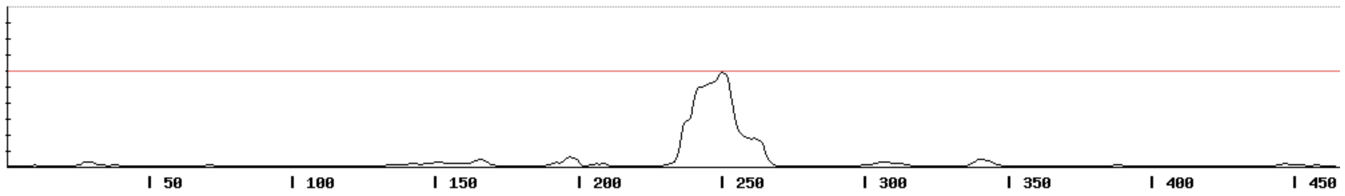

Predictions for CmjSPL6B

Viterbi Path

Viterbi Path

Posterior @ 0.6

234 - KRLAGHNERRRKP - 246

Posterior @ 0.6

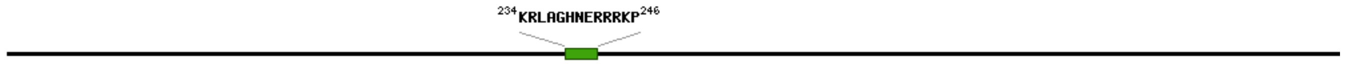

Posterior Graphic. Threshold Marked @ 0.6

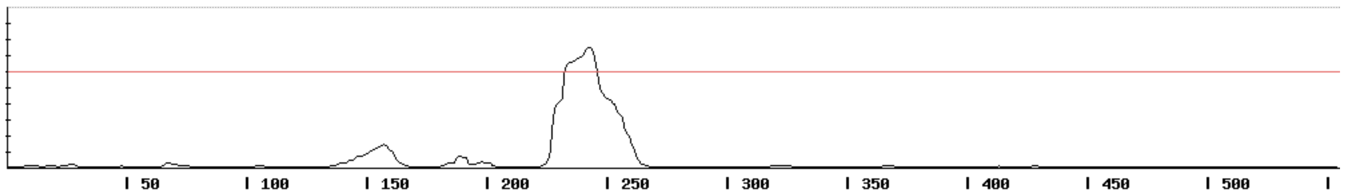

Predictions for CzpSPL6B

Viterbi Path

Viterbi Path

Posterior @ 0.6: 234 - KRLAGHNERRRKP - 246

Posterior @ 0.6

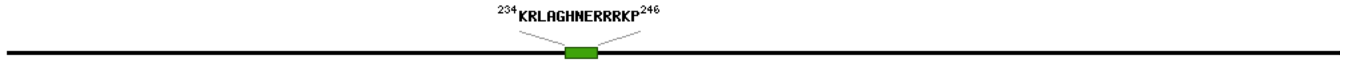

Posterior Graphic. Threshold Marked @ 0.6

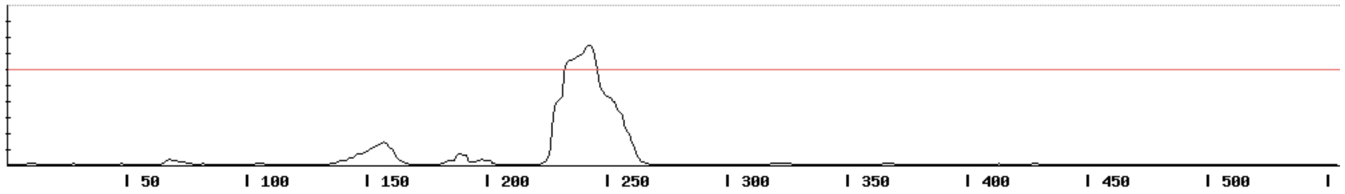

Predictions for CmSPL6A

Viterbi Path

Viterbi Path

Posterior @ 0.6

Posterior @ 0.6

Posterior Graphic. Threshold Marked @ 0.6

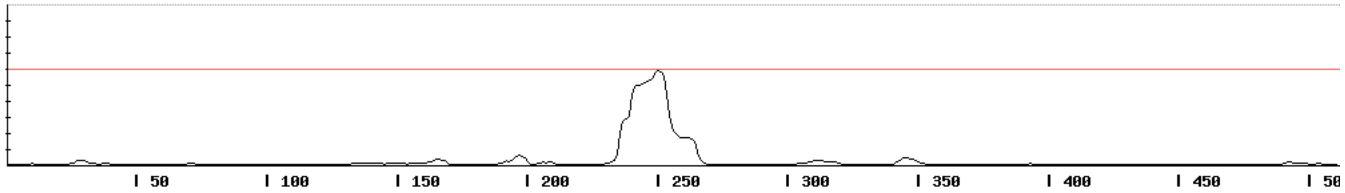

Predictions for CmSPL6B

Viterbi Path

Viterbi Path

Posterior @ 0.6: 234 - KRLAGHNERRRKP - 246

Posterior @ 0.6

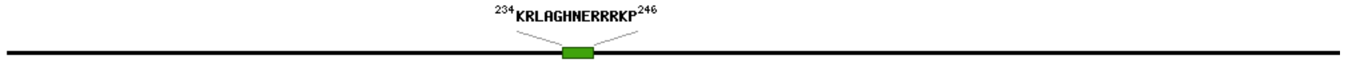

Posterior Graphic. Threshold Marked @ 0.6

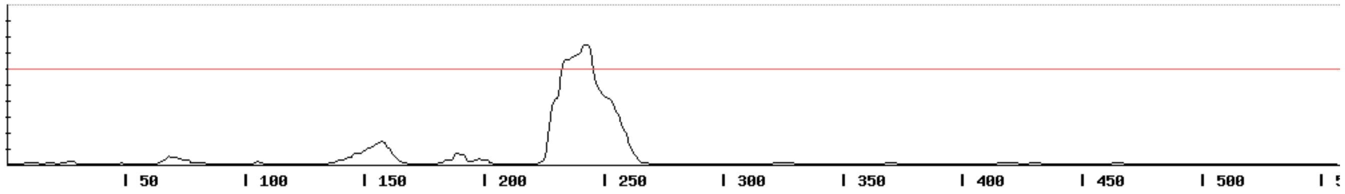

alex.nguyenba@utoronto.ca
