# Supplementary material for: Evolutionary assessment of SQUAMOSA PROMOTER BINDING PROTEIN-LIKE genes in citrus relatives with a specific focus on flowering
Source: Mol Hortic. 2023 Jul 20;3:13. doi: 10.1186/s43897-023-00061-4 (PMC10515035; doi:10.1186/s43897-023-00061-4)
Supplement: Supplementary file 1 — Additional file 1: The online version contains supplementary material available at (web address will be provided by the publisher). Supplementary Fig. S1. Prediction of target sites for miR156 in SPL. Supplementary Fig. S2. SPL conserved domain sequence alignment. Supplementary Fig. S3. Gene structure. Supplementary Fig. S4. SPL-motifs prediction. Supplementary Fig. S5. Nuclear localization prediction. Supplementary Fig. S6. Cis-acting element pred by PlantCARE + TBTOOLS. Supplementary Fig. S7. FhSPL9 and FhSPL11 Mutation Sites. Supplementary Table S1. The characteristics of identified SPL genes in Citrus. Supplementary Table S2. Table S2. Prediction of miR156 and SPL target sequences. Supplementary Table S3. Prediction of miR156 target genes in Fortunella hindsii. Supplementary Table S4. Vector construction and quantitative primers for gene expression detection. Supplementary Table S5. Quantitative PCR primers for SPL genes of Fortunella hindsii, Citrus sinensis, Citrus reticulata ‘Pokan’ and Citrus maxima‘Majia’. [file 43897_2023_61_MOESM1_ESM.zip › Figure S6 Cis-acting element pred by PlantCARE + TBTOOLS.pdf]

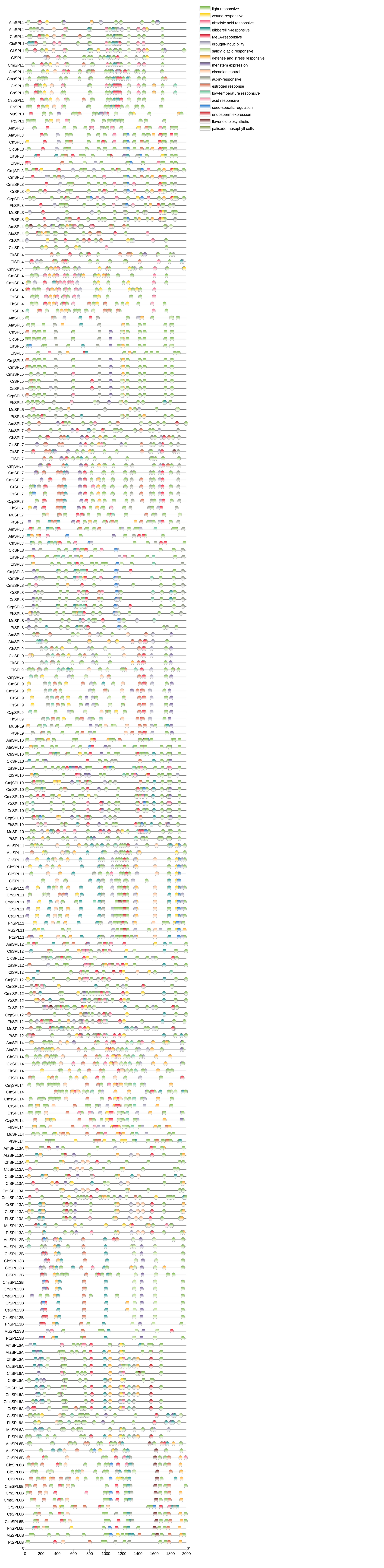
light responsive
wound-responsive
abscisic acid responsive
gibberellin-responsive
MeJA-responsive
drought-inducibility
salicylic acid responsive
defense and stress responsive
meristem expression
circadian control
auxin-responsive
estrogen response
low-temperature responsive
acid responsive
seed-specific regulation
endosperm expression
flavonoid biosynthetic
palisade mesophyll cells
0
200
400
600
800
1000
1200
1400
1600
1800
2000
